# The moderating role of informatization between country risks and international tourism: A cross-country panel analysis

**DOI:** 10.1371/journal.pone.0278518

**Published:** 2022-12-16

**Authors:** Zhaoming Deng, Meijing Zhou, Qiong Xu

**Affiliations:** 1 Institute of Geographic Sciences and Natural Resources Research, Chinese Academy of Sciences, Beijing, China; 2 State Key Laboratory of Resources and Environmental Information System, Chinese Academy of Sciences, Beijing, China; 3 College of Humanities and Social Sciences, Beijing Institute of Petrochemical Technology, Beijing, China; 4 Beijing Academy of Safety Engineering and Technology, Beijing, China; 5 Business School, Central South University, Changsha, China; University of Almeria: Universidad de Almeria, SPAIN

## Abstract

Informatization plays an increasingly important role in the tourism industry, while its effectiveness in alleviating tourism risks remains to be verified. This research aims to explore the effects of country risks on the international tourism and the moderating role of informatization between the two. This study firstly measures country risks based on the ICRG database, quantifies international tourism by tourism revenue, tourism expenditure, and tourist arrival, and calculates informatization level from informatization facilities, informatization applications, and informatization skills. A dynamic SYS-GMM model is then adopted to verify the research hypotheses based on the panel data of 138 countries from 2000 to 2019. The research results show that the composite country risk, political risk, economic risk, and financial risk all show a negative impact on the international tourism indicators regardless of different time periods, regions, or income levels. However, the effects are more obvious before the global financial crisis in 2008 and regions and countries with lower income levels. In addition, informatization is found to positively mitigate the adverse impacts of country risks on international tourism, especially for economic and financial risks. The research findings indicate the risk hedge potential of informatization in the tourism industry, which provides a profound reference for destination risk management.

## Introduction

Tourism has become a pillar industry of the world economy [1]. The global tourism revenue contributed 6.6–6.9% to the world’s GDP from 2016 to 2019 [2]. However, tourism is quite vulnerable to external risks, such as terrorist attacks, natural disasters, climate change, financial and economic crises [3], and pandemics. The outbreak of SARS in 2003 made the tourism industry in China, Hong Kong, Singapore, and Vietnam lose 3 million jobs and more than $20 billion, and the rest of Asia saw visitor numbers drop by 70% or more [4]. International tourist arrival dropped by 4% in 2009 due to the global financial crisis [5]. In addition, affected by COVID-19, the share of global tourism revenue in the world’s GDP declined to 3.7% and 3.8% in 2020 and 2021, respectively [2], and international tourist arrival decreased by 71%, 71%, and 54% in 2020, 2021 and 2022 compared to 2019, separately [6].

As a long-distance and cross-cultural travel, international tourism is greatly influenced by risks, mainly including terrorism, war and political instability, health issues, and crime [7]. After the outbreak of major financial crises, such as the Asian financial crisis in 1997, the impacts of economic and financial risks on tourism started to receive attention from academics [8]. Generally, scholars have qualitatively analyzed the adverse impacts of these risks on tourism development [9–12], or quantitatively testified the relationship between these risks and tourism growth indicators through surveys [11], case studies [13], and econometric modeling [14–17]. Country risk (CR), a comprehensive expression of political, economic, and financial risks of a country, has become a new channel to explore the impacts of risks on tourism. In particular, research has been facilitated by the availability of CR data covering time series from multiple countries [18–20]. Among all the studies, most of them have explored the effects of CRs on tourism in a certain country or region, and analysis at a global scale is relatively lacking in the research field. In addition, the impacts of different risk types, such as political risk (PR), economic risk (ER), and financial risk (FR), on various tourism growth indicators, such as tourism revenue (TR), tourism expenditure (TE) and tourist arrival (TA), have been ignored in previous research. Most scholars have only considered the unilateral impacts of risks on TA or TE, ignoring the overall measurement of the risk effects [21].

Meanwhile, risk management at destinations has been a hot topic given the severe results of tourism risks, including risk management principles and procedures [22], financial hedging strategies towards climate risk [23], authoritative guide for managing crises and disasters [24], management model for specifying risk sources [25], management mechanism and techniques for disaster risk [26, 27], and risk management strategies for adventure tourism [28, 29], etc. Thanks to the high integration of informatization and tourism, the comprehensive benefits of tourism have been significantly improved. In recent years, “smart tourism” and “Internet + tourism” have become new models of tourism development [30, 31]. It is well-documented that the development of informatization has brought new opportunities for tourism [32, 33]. On the one hand, informatization has improved tourism efficiency by enriching the tourist experience, activating the potential tourism market, and promoting the rapid growth of tourist numbers and tourism consumption levels. On the other hand, informatization has expanded the scale of publicity in the tourism industry, which contributes to the construction of a comprehensive and intelligent tourism service system, thereby strengthening the tourism industry’s ability to withstand risks. Informatization plays a significant role in supporting and safeguarding tourism industry against risks, as well as enables the tourism industry to turn risks into opportunities [34]. The positive role of informatization in tourism risk management has already been verified [35, 36]. But few scholars have explored the risk mitigation effects of informatization in the tourism industry.

To address the above research gaps, this study adopts an international perspective to examine and compare the impacts of different risk types on various tourism indicators in different countries and regions, thereby revealing the temporal and spatial differences in impact. Additionally, this study introduces informatization as a risk hedge measure to analyze the mitigation mechanism between external risks and tourism growth, thus providing practical implications for destination risk management. The framework of this paper is as follows. Section 2 proposes research hypotheses based on existing theories and literature review. Section 3 describes the research data and methods. Section 4 presents the empirical analysis results of the impacts of CRs on international tourism. Section 5 illustrates the moderating effect results. And section 6 discusses and summarizes the research findings.

## Theoretical foundation and research hypotheses

### Theory of planned behavior

**Fig 1:**
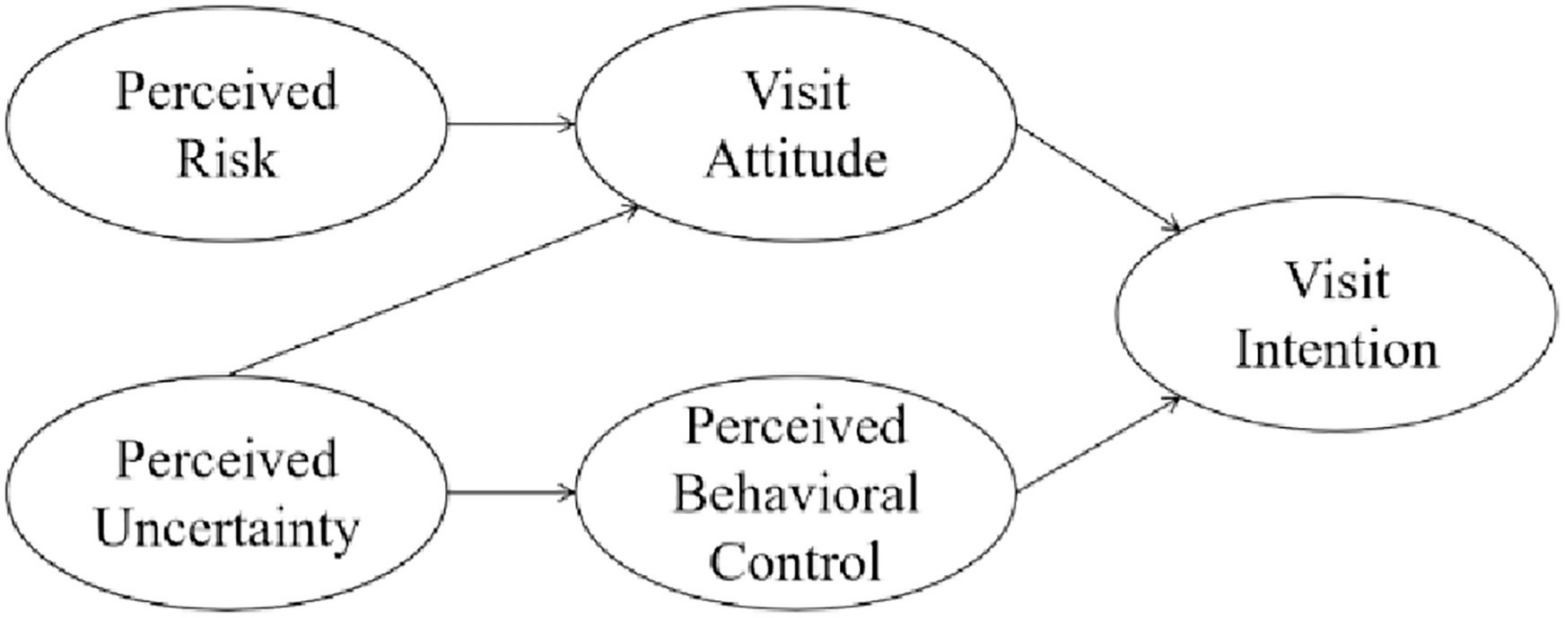
The relationships between perceived risk, perceived uncertainty, and visit intention based on TPB. Source: Modified from Quintal, Lee [40].

According to the theory of planned behavior (TPB), people’s behavioral intentions and actual behaviors are influenced by three kinds of beliefs, including the beliefs about the possible results of an action (behavioral beliefs, BB), beliefs about the normative expectations of others (normative beliefs, NB), and beliefs about the factors that may promote or impede the behavioral performance (control beliefs, CB) [37]. Further, BB generates people’s attitudes towards an action, NB produces people’s perceived social pressure about whether to perform an action, and CB leads to people’s perceived ability to perform a given behavior [38]. Generally, the greater the three beliefs, the stronger people’s intentions to perform the behavior, and the more likely the planned behavior to happen [37]. TPB has a wide range of applications in tourism, leisure, and hospitality management, and risk management is one of the most important research contexts [39]. Within the TPB framework, perceived risk or uncertainty negatively impacts travel intentions by changing people’s travel attitude and perceived behavioral control [40]. In this sense, CRs, consisting of PR, ER, and FR, will significantly reduce people’s travel frequency. When a destination encounters CR, people will perceive personal risk or economic loss before making the travel decision and probably cancel the travel plan. Meanwhile, they will be uncertain about whether they can ensure their personal safety and property safety during the journey. The relationships between perceived risk, perceived uncertainty, and visit intention based on TPB are illustrated in Fig 1.

### Risk perception attitude framework

**Fig 2:**
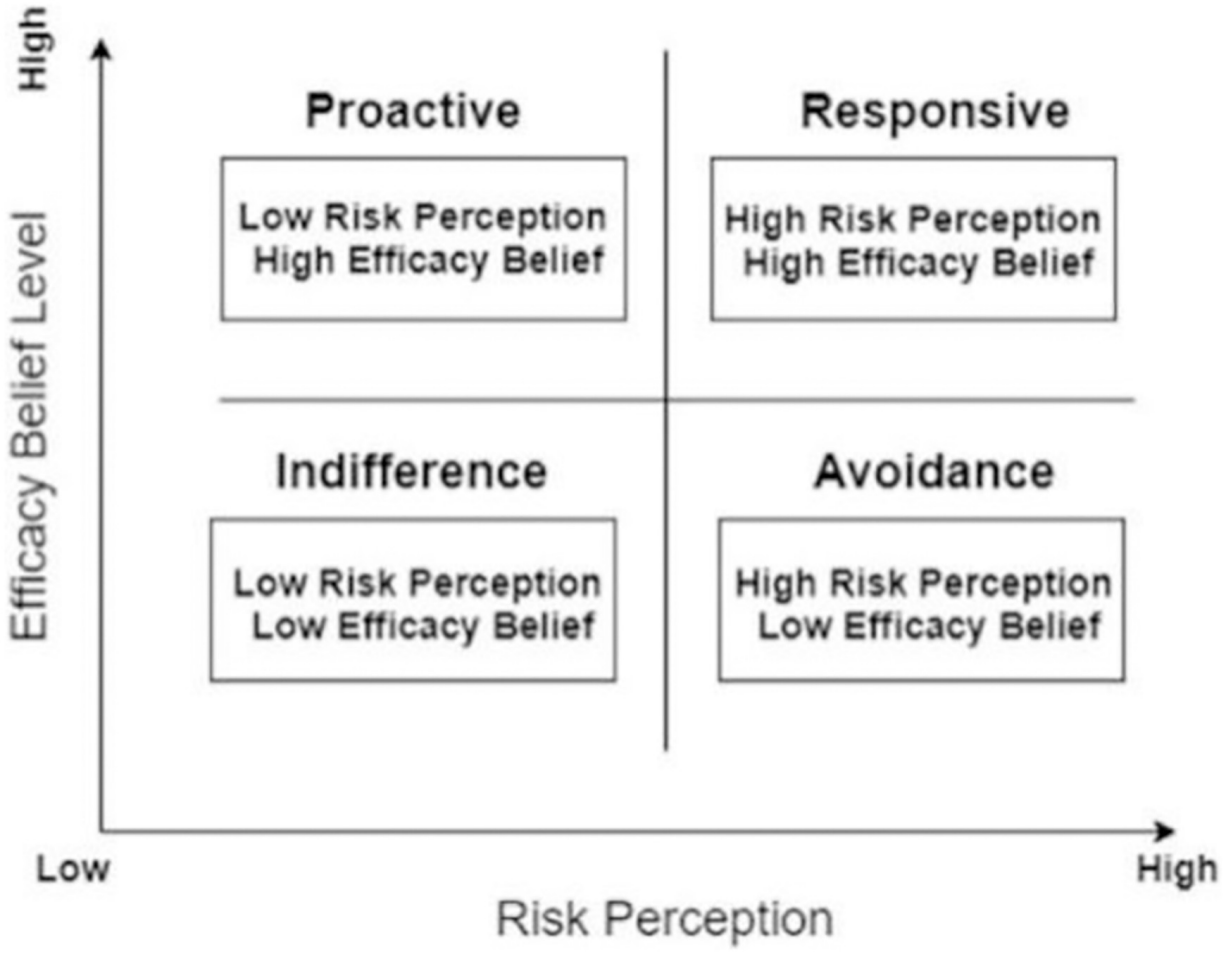
Risk perception attitude framework. Source: Chisty, Islam [44].

The risk perception attitude framework (RPAF) explains people’s reactions to risks based on their perceived risk level and efficacy belief level (Fig 2). According to the framework, efficacy belief and risk perception level significantly affect individuals’ visit intentions [41]. Therefore, destinations need to reduce tourists’ risk perceptions and improve their efficacy beliefs to increase TA. In the internet information era, destinations’ informatization level greatly contributes to managing tourists’ risk perception and efficacy belief [41]. On the one hand, a high informatization level helps spread related information, which increases the transparency of risk events, thereby facilitating tourists’ decision- making before visits and crisis management during visits [42]. On the other hand, information searching is one of the important ways for tourists to prevent risks and reduce uncertainties [43]. Destinations’ high informatization level enables tourists to obtain valid and timely information, thus improving their self-efficacy in dealing with risks or uncertainties. In this way, informatization plays an important moderating role between risks and visiting behaviors.

### Hypotheses formulation

Based on TPB, RPAF, and previous literature, this study proposes the following hypotheses.

PR directly threatens the personal safety of tourists, leading to a reduction in TA and travel time [21, 45]. The destructive impacts of political events (e.g., terrorist attacks and conflicts) on tourism have been widely verified [11, 31, 46]. Frequent PR can damage the image of tourist destinations and generate negative publicity [47, 48]. Conflicts adversely determine the perceptions of international tourists about a destination [49] and decreases tourist visits [50]. Generally, tourists change their destination choices or even abandon their travel plans under risky conditions [51]. Meanwhile, a turbulent political environment could cause a large number of service providers and operators in tourism industry to suspend business activities [52], thereby hindering the tourism development [53, 54]. Generally, tourism growth and development are largely dependent on a risk-free and politically stable environment [55]. Therefore, destinations that are politically unstable and/or directly involved in terrorist activities generally see a decline in visitor numbers and tourism receipts [41].

Based on the above literature, the following research hypotheses are proposed.

H1: PR has a negative impact on TR.
H2: PR has a negative impact on TE.
H3: PR has a negative impact on TA.

Tourism is a comprehensive industry that integrates restaurants, hotels, transportation, traveling, shopping, and entertainment, which is strongly dependent on the external economic environment. Therefore, an increase in ER brings uncertainty to the supporting environment of tourism, which reduces tourist numbers and TE [56]. ER is believed to be part of tourist destinations, which is negatively correlated with TE [57]. In addition, ER increases the transaction cost and internal management fees of tourism operators, which indirectly reduces TR [58]. From the tourist perspective, daily consumption depends on disposable income and expectations of future changes in risky economic conditions [17]. Since tourism belongs to non-necessary consumer goods, individuals prefer to cut tourism expenses or choose domestic travel instead of international travel in the face of an economic crisis [59, 60]. As a result, international TA, TE, and revenue are negatively affected by economic crises [16]. The adverse impacts of the economic crisis on tourism development have been extensively confirmed by existing studies [61–65].

Based on the above research, this paper proposes the following hypotheses.

H4: ER has a negative impact on TR.
H5: ER has a negative impact on TE.
H6: FR has a negative impact on TA.

Tourism is an international trade activity. The occurrence of FR increases the uncertainty of a country’s trading environment and production cost, which reduces tourism operators’ confidence to invest, thus negatively affecting the scale of tourism operations and tourism development level [17]. For tourists, affordability is one of the prerequisites for traveling. Similar to the economic crisis, when faced with FR, tourists tend to reduce travel frequency and control TE [66]. Chan [67] verifies that the financial crisis seriously threatens Macau’s tourism industry. Sio-Chong and So [8] conclude that FR poses a greater threat on tourism than non-financial risks. The negative association between the financial crisis and tourism development has been verified by copious literature [67–71].

Based on the above studies, the following research hypotheses are made in the current paper.

H7: FR has a negative impact on TR.
H8: FR has a negative impact on TE.
H9: FR has a negative impact on TA.

The penetration of information and communication technology (ICT) has improved the level of tourism informatization, which provides more opportunities for the tourism industry to resist CR. On the one hand, informatization accelerates the dissemination of risk information, which enables tourists to cope with risks in advance or in time, thereby reducing the loss of tourist numbers. In addition, the government can scientifically configure tourism flow based on enough information, thereby extending the buffer time of risk mitigation and lowering tourism economic losses. Brown [72] found that broadcasting and media affect tourists’ emotions and enhance tourists’ awareness of negative events at destinations. On the other hand, the tourism industry can use ICT to transform risks into unique advantageous tourism resources, thereby innovating tourism business models (e.g., smart tour guides and online visits) and increasing the added value of risks [35]. Mandal and Dubey [36] confirmed the positive role of tourism IT adoption in promoting tourism supply chain resilience. concluded that the development of ICTs promoted international tourism.

Based on the above analyses, this article proposes the following hypotheses.

H10: Informatization mitigates the negative impact of CRs on TR.
H11: Informatization mitigates the negative impact of CRs on TE.
H12: Informatization mitigates the negative impact of CRs on TA.

## Data and methods

### Explained variables

Tourism is the sum of various economic activities and economic phenomena caused by tourism activities [18]. TR, TE, and TA are often recognized as the three major tourism indicators [18, 73]. TR is the total monetary income that a tourist destination obtains through the sale of tourism goods and services, which reflects the overall scale and development level of the tourism industry [74, 75]. TE is a currency form that reflects tourists’ consumption level for tourism goods and services, which is reflected by TA and unit tourist expenditure [76, 77]. TA refers to the tourist flow in a tourism destination, which is calculated by the number of tourists and the number of trips per capita [78, 79]. TR, TE, and TA focus on the development level, consumption level, and flow scale of the tourism industry, respectively. This paper uses TR, TE, and TA to characterize a country’s tourism. The data mainly come from the World Bank.

### Independent variables

CR indicates the possibility that a sovereign state or a borrower in a particular country fails to repay foreign lenders, and/or investors, and CR assessment aims primarily to predict payment problems for sovereign borrowers [19]. Among all the evaluation databases, ICRG is the most widely used one for measuring CRs, which contains monthly indexes of 146 countries from 1984 to 2020. ICRG comprehensively evaluates CRs from 22 indicators in three dimensions (i.e., PR, ER, and FR). PR is measured by 12 indicators involving both political and social aspects, including government stability, socioeconomic conditions, investment profile, internal conflict, external conflict, corruption, military in politics, religious tensions, law and order, ethnic tensions, democratic accountability, and bureaucracy quality; ER reflects a country’s economic status by investigating its GDP per head, real GDP growth, annual inflation rate, budget balance as a percentage of GDP, and current account as a percentage of GDP; FR estimates a country’s financing ability to pay its debt officially, commercially and in trade by calculating its foreign debt as a percentage of GDP, foreign debt service as a percentage of exports of goods and services, current account as a percentage of exports of goods and services, net international liquidity as months of import cover, and exchange rate stability [80]. In terms of evaluation scores, the composite CR ranges from 0 to 100, totaling 50% of PR (0–100), ER (0–50), and FR (0–50). The higher the score, the lower the risk.

### Moderate variable

Following the studies of Gretzel [35], Bris, Pawlak [81], and Hong, Thakuriah [82], this paper evaluates a country’s informatization level (INF) from three dimensions of informatization facilities, informatization applications, and informatization skills (Table 1). Among the three dimensions, fixed telephone ownership rate, mobile phone ownership rate, computer ownership rate, and user internet average broadband are used to characterize information facilities. The data mainly come from the International Telecommunication Union. Informatization applications are evaluated by internet penetration rate, fixed Internet penetration rate, mobile informatization fixed broadband users, and the population using the Internet. The data mainly come from the World Bank. The adult literacy rate, junior high school enrollment rate, and high school enrollment rate that measure the informatization skills mainly come from the World Bank and Bloomberg database. The weight of each index is obtained based on the fuzzy analytic hierarchy process and the importance of each index. First, all the indicators are standardized to reduce the dimensional difference of the original data as formula (1).


$$X_{i}^{\text{'}}=\frac{X_{i}-\overset{\ldots }{X_{i}}}{S_{i}},X_{i}=\frac{1}{n}\sum_{j=1}^{n}X_{ij},S_{i}=\sqrt{\frac{1}{n-1}}\sum_{j=1}^{n}{\left(X_{ij}-\bar{X_{i}}\right)}^{2}$$ (1)

**Table 1:** Informatization index system.

Variable	Category	Indicators
**Informatization**	Information facilities (40.6%)^a^	Information of the world (7.0%)^a^
		Mobile phone ownership (10.7%)^a^
		Computer ownership (10.8%)^a^
		Average international internet broadband of users (12.1%)^a^
		Number of internet users per 100 residents (13.1%)^a^
	Information application (40.1%)^a^	Number of fixed internet users per 100 residents (8.1%)^a^
		Number of mobile internet users per 100 residents (10.9%)^a^
		Internet development speed (8.0%)^a^
	Information skills (19.3%)^a^	Adult literacy (5.4%)^a^
		Number of junior high school enrollment (7.4%)^a^
		Number of high school enrollment (6.5%)^a^

^a^the values in the brackets are the weight of the indicators.

Secondly, the informatization of each country was calculated according to the entropy method as formula (2).

$$INF=\sum_{i-1}^{n}Y_{i}\left(\sum_{j=1}^{m}Y_{i}X_{ij}\right)$$ (2)

Where *X*_*ij*_ is the standardized value of the *j* indicator of the *i* category, *Y*_*ij*_ is the weight of the *j* indicator of the *i* category, *Y*_*i*_ is the weight of the *i* category, *n* is the number of categories, and *m* is the number of indicators under each category.

### Control variables

Opening-up level (OPL). The improvement of the opening degree to the outside world not only provides more convenient and preferential travel conditions for tourists, but also broadens trade channels for the transnational investment of tourism enterprises. Therefore, there is a positive correlation between OPL and tourism [58]. OPL is evaluated by the proportion of total imports and exports of goods in GDP, with data from the World Bank.

Tourism resource endowment (TRE). Tourism resources are the attraction base of tourist destinations and important sources of TR. In general, the richer the tourism resources, the easier for a destination to attract tourists, which is conducive to increasing TR. Therefore, TRE shows a positive impact on tourism development [83]. This article uses the number of world heritage sites in a country to represent TRE, and the data mainly come from the World Heritage Center of UNESCO.

Exchange rate (EX). The fluctuation of EX directly affects the demand for tourism. Various countries use exchange rate to adjust the supply and demand of tourism. When the exchange rate of a tourist destination rises, the currency value of the tourist source country depreciates, and the purchasing ability of tourists decreases, thereby reducing the tourism demand. On the contrary, when the exchange rate of a tourist destination drops, the currency value of the tourist source country appreciates, and the purchasing ability of tourists increases, which stimulates the tourism demand. Therefore, the exchange rate is negatively correlated with tourism [84]. In this paper, EX is calculated by the convertible local currency of one USD, where real price processing refers to [85], and the influence of relative price is excluded. The data are mainly from the World Bank.

Economic development level (EDL). EDL is an important foundation for a country’s tourism development. From the demand side, tourism belongs to a high-level demand, which only occurs when economic development reaches a certain level. From the supply side, a higher economic development level helps provide better supporting facilities and services for tourism [83]. Therefore, EDL is a positive predictor of tourism. This article uses GDP per capita (PGDP) to characterize EDL, which considers the relative price of both a tourist destination and a tourist source. The data come from the World Bank.

By matching various index databases, the paper selects the intersection of each database, eliminates the outliers, and fills the missing data to construct a panel data for 138 countries from 2000 to 2019. The countries cover the five continents (i.e., Asia, Europe, Africa, America, and Oceania), and include high-income, middle-income, and low-income countries. In these countries, the population and GDP account for 95% and 97% of the world’s total, respectively; the tourist number is 94% of the world’s total population, and the TR accounts for 96% of the world’s total. The descriptive statistics of the variables are shown in Table 2. The results show that among the three international tourism indicators, the average values of TR and TE are close, but TR fluctuates more wildly; TA has both the least average value and fluctuation. Among the four risk indexes, FR has the highest mean value and standard deviation, and the composite CR has the smallest average value. Informatization has a moderate mean value and volatility. Among all the control variables, the economic development level has the largest mean value and the second greatest standard deviation; tourism resource endowment has the smallest average value and the second smallest standard deviation; the opening-up level has the minimum fluctuations and the second largest mean value.

**Table 2:** Descriptive statistical results of the variables.

Variables	Unit	Obs	Mean	Std. Dev	Max	Min
**Tourism revenue (lnTR)** ^a^	$	2760	21.110	2.136	26.194	11.513
**Tourism expenditure (lnTE)** ^a^	$	2760	21.010	1.978	25.929	15.425
**Tourist arrival (lnTA)** ^a^	person	2760	14.445	1.821	18.928	8.430
**Country risk (lnCR)** ^a^	--	2760	3.352	0.590	4.174	2.031
**Political risk (lnPR)** ^a^	--	2760	3.423	0.453	4.233	1.365
**Economic risk (lnER)** ^a^	--	2760	4.159	0.090	4.537	3.601
**Financial risk (lnFR)** ^a^	--	2760	8.569	1.564	11.685	1.556
**Informatization (lnINF)** ^a^	--	2760	3.022	1.307	4.612	0.001
**Opening-up level (lnOPL)** ^a^	--	2760	3.606	0.615	5.438	0.095
**Tourism resource endowment (TRE)**	--	2760	1.899	0.740	4.127	0.693
**Exchange rate (ER)**	--	2760	3.132	2.551	10.789	0.000
**Economic development Level (ln PGDP)** ^a^	$	2760	8.571	1.556	11.685	4.718

^a^ln denotes the data of the variable are natural logarithm.

## Methods

### System GMM model

Compared with other general linear methods, the generalized method of moment (GMM) not only deals with the endogenous problem, but also considers the hysteresis effect with good robustness [86]. Specifically, GMM takes the lag term of the core independent variable as an explanatory variable, thereby eliminating fixed effects. The difference GMM (DIF-GMM) and system GMM (SYS-GMM) models are both designed for panel data with “large N, small T”, which means much more individuals than time periods [86]. Compared with DIF-GMM, SYS-GMM overcomes the weak instrumental variable problem, thereby solving the endogeneity problem better, and improving the efficiency of estimation [87–89]. Because of these superiorities in dealing with dynamic panel data, SYS-GMM has been widely used in economic modeling analyses [90–94]. The dataset in this study covers 138 countries and 20 years, which is the typical “large N, small T” panel data. In addition, the impacts of external uncertainty on tourism are profound and lasting, leading to a lag effect. Tiwari, Das [95] believe that both PR and ER have significant lagging effects on tourists. Therefore, this paper employs the SYS-GMM model to conduct empirical analyses.

The formulas of the SYS-GMM model in this paper are seen in formulas (3) to (6).

$$\begin{array}{ll} lnTR_{it}/lnTE_{it}/lnTA_{it}= & \alpha_{0}+\alpha_{1}lnTR_{it-1}/lnTE_{it-1}/lnTA_{it-1}+\alpha_{2}lnCR_{it}+\alpha_{3}lnOPL_{it}\\ & +\alpha_{4}lnTRE_{it}+\alpha_{5}EX_{it}+\alpha_{6}PGDP_{it}+\lambda_{i}+\mu_{t}+\;\varepsilon_{it} \end{array}$$ (3)

$$\begin{array}{ll} lnTR_{it}/lnTE_{it}/lnTA_{it}= & \alpha_{0}+\alpha_{1}lnTR_{it-1}/lnTE_{it-1}/lnTA_{it-1}+\alpha_{2}lnPR_{it}+\alpha_{3}lnOPL_{it}\\ & +\alpha_{4}lnTRE_{it}+\alpha_{5}EX_{it}+\alpha_{6}PGDP_{it}+\lambda_{i}+\mu_{t}+\varepsilon_{it} \end{array}$$ (4)

$$\begin{array}{ll} lnTR_{it}/lnTE_{it}/lnTA_{it}= & \alpha_{0}+\alpha_{1}lnTR_{it-1}/lnTE_{it-1}/lnTA_{it-1}+\alpha_{2}lnER_{it}+\alpha_{3}lnOPL_{it}\\ & +\alpha_{4}lnTRE_{it}+\alpha_{5}EX_{it}+\alpha_{6}PGDP_{it}+\lambda_{i}+\mu_{t}+\varepsilon_{it} \end{array}$$ (5)

$$\begin{array}{ll} lnTR_{it}/lnTE_{it}/lnTA_{it}= & \alpha_{0}+\alpha_{1}lnTR_{it-1}/lnTE_{it-1}/lnTA_{it-1}+\alpha_{2}lnFR_{it}+\alpha_{3}lnOPL_{it}\\ & +\alpha_{4}lnTRE_{it}+\alpha_{5}EX_{it}+\alpha_{6}PGDP_{it}+\lambda_{i}+\mu_{t}+\varepsilon_{it} \end{array}$$ (6)

Where ln denotes natural logarithm; it means region i and year t, it-1 refers to the first order lag; α0, α1, α2, α3, α4, α5, and α6 are the estimated coefficients; TR, TE, and TA represent tourism revenue, TE and TA, respectively; CR, PR, ER, and FR are the composite country risk, PR, ER, and FR, respectively; OPL, TRE, EX, and PGDP denote opening-up level, the endowment of tourism resources, exchange rate, and the economic development level, respectively; λi and μt are fixed effects for time and region, respectively; and εit indicates the random error term. The same symbols below have the same meaning.

### Moderating effect model

This paper introduces the moderating variable (ln*INF*_*it*_), the cross-terms of the moderating variable, and independent variables (*lnCR*_*it*_ × *lnINF*_*it*_, *lnPR*_*it*_ × *lnINF*_*it*_, *lnER*_*it*_ × *lnINF*_*it*_, *lnFR*_*it*_ × *lnINF*_*it*_) into the direct effect model, thereby estimating the mitigation effects of informatization between the dependent variables (TR, TE, and TA) and independent variables (CR, PR, ER, and FR). If the independent variables, moderating variable, and the cross-terms all significantly impact the dependent variables in the same direction, it indicates that the moderating variable plays a significant positive moderating role; if they show significant but inconsistent influence, it means the moderating variable plays a negative moderating effect; if one of the impacts is not significant, it means the moderating variable is not functioning. The formulas for testing moderating effects are seen in formulas (7) to (10).

$$\begin{array}{l} \begin{array}{ll} lnTR_{it}/lnTE_{it}/lnTA_{it}= & \alpha_{0}+\alpha_{1}lnTR_{it}/lnTE_{it}/lnTA_{it}+\alpha_{2}lnCR_{it}+\alpha_{3}lnINF_{it}+\;\alpha_{4}lnCR_{it}\times \\ & lnINF_{it}+\alpha_{5}lnOPL_{it}+\alpha_{6}TRE+\alpha_{7}EX+\alpha_{8}lnPGDP+\lambda_{i}+\mu_{t}+\varepsilon_{it} \end{array} \end{array}$$ (7)

$$\begin{array}{ll} lnTR_{it}/lnTE_{it}/lnTA_{it}= & \alpha_{0}+\alpha_{1}lnTR_{it}/lnTE_{it}/lnTA_{it}+\alpha_{2}lnPR_{it}+\alpha_{3}lnINF_{it}+\alpha_{4}lnPR_{it}\times \\ & lnINF_{it}+\alpha_{5}lnOPL_{it}+\alpha_{6}TRE+\alpha_{7}EX+\alpha_{8}lnPGDP+\lambda_{i}+\mu_{t}+\varepsilon_{it} \end{array}$$ (8)

$$\begin{array}{ll} lnTR_{it}/lnTE_{it}/lnTA_{it}= & \alpha_{0}+\alpha_{1}lnTR_{it}/lnTE_{it}/lnTA_{it}+\alpha_{2}lnER_{it}+\alpha_{3}lnINF_{it}+\alpha_{4}lnER_{it}\times \\ & lnINF_{it}+\alpha_{5}lnOPL_{it}+\alpha_{6}TRE+\alpha_{7}EX+\alpha_{8}lnPGDP+\lambda_{i}+\mu_{t}+\varepsilon_{it} \end{array}$$ (9)

$$\begin{array}{ll} lnTR_{it}/lnTE_{it}/lnTA_{it}= & \alpha_{0}+\alpha_{1}lnTR_{it}/lnTE_{it}/lnTA_{it}+\alpha_{2}lnFR_{it}+\alpha_{3}lnINF_{it}+\alpha_{4}lnER_{it}\times \\ & lnINF_{it}+\alpha_{5}lnOPL_{it}+\alpha_{6}TRE+\alpha_{7}EX+\alpha_{8}lnPGDP+\lambda_{i}+\mu_{t}+\varepsilon_{it} \end{array}$$ (10)

Where INF denotes informatization; the other variables have the same meaning as those in the formulas (3) to (6).

## Empirical analysis

### Unit root test

Unit root test was carried out to test the stationarity of the variables. The IPS test [96], Fisher-ADF test [97], and Fisher-PP test [98] were employed to perform the unit root tests. Table 3 shows that all the null hypotheses are rejected at 1% or 5% significance level for the three tests, which means that there is no unit root in the variables, i.e., all the variables are stationary.

**Table 3:** Unit root test.

Variables	IPS	ADF	PP
**lnTR**	-1.093***^a^	346.928***^a^	890.018***^a^
**lnTE**	-1.302**^b^	406.029***^a^	719.987***^a^
**lnTA**	-1.009***^a^	455.011***^a^	710.009***^a^
**lnCR**	-0.193***^a^	393.913***^a^	655.201***^a^
**lnPR**	-0.201***^a^	319.610**^b^	575.199***^a^
**lnER**	-0.329***^a^	293.512**^b^	534.791**^b^
**lnFR**	-0.274**^b^	264.193***^a^	614.501***^a^
**lnINF**	-0.231***^a^	313.193***^a^	514.917***^a^
**lnOPL**	-0.194***^a^	293.903***^a^	554.181***^a^
**TRE**	-0.189**^b^	319.877**^b^	524.019***^a^
**EX**	-0.174***^a^	293.128**^b^	394.337**^b^
**lnPGDP**	-0.199***^a^	337.219**^b^	345.193***^a^

^a^*** indicates the rejection of null hypotheses at 1% significance level, the same below.

^b^** indicates the rejection of null hypotheses at 5% significance level, the same below.

### Panel cointegration test

Cointegration test was used to avoid pseudo regression. This study tests the overall panel cointegration between CRs and international tourism based on the methods proposed by Kao [99] and Pedroni [100]. Table 4 shows that for the Pedroni test, the null hypotheses are rejected at 5% or 1% significance level for Panel-v, Panel-PP, Panel-ADF, Group-PP, and Group-ADF, indicating that there is a cointegration relationship in the panel data. For the Kao test, the null hypothesis is rejected for the ADF statistic at 1% significance level. The panel cointegration test results show that CRs have a long-term impact on international tourism. Combining the unit root test and panel cointegration test, the panel data are stationary and cointegrated, which is suitable for the following dynamic panel analysis.

**Table 4:** Panel cointegration test.

Method	Statistic	Panel
**Pedroni residual cointegration**	Panel v statistic	5.192**
	Panel rho-statistic	-2.469
	Panel PP statistic	-39.284***
	Panel ADF statistic	-32.914***
	Group rho statistic	7.910
	Group PP statistic	-30.109***
	Group ADF statistic	-17.193***
**Kao residual cointegration test**	ADF statistic	9.392***

### The impact of different risk types on international tourism

The dynamic SYS-GMM method was used to estimate the impact of CRs on international tourism, and the results are seen in Tables 5–10. AR (1) and AR (2) represent the first and second difference of the error term, respectively. The results show that AR (1) passes the Wald test at 5% or 1% significance level, and AR (2) fails to pass the Wald test, indicating that the first-order difference of the error term is correlated, and the second-order difference is uncorrelated. In addition, the results demonstrate that the null hypothesis of “all instrumental variables are valid” in Hansen’s overidentification test is accepted, indicating the validity of the selection of instrumental variables, and the authenticity and credibility of the estimation results.

**Table 5:** The impact of different types of CRs on TR.

Variable	lnCR	lnPR	lnER	lnFR
**L.lnTR** ^a^	0.965***	0.885***	0.954***	0.917***
	(0.004) ^b^	(0.003) ^b^	(0.002) ^b^	(0.004) ^b^
**lnCR**	-0.307***			
	(0.032) ^b^			
**lnPR**		-0.291***		
		(0.011) ^b^		
**lnER**			-0.352***	
			(0.028) ^b^	
**lnFR**				-0.568***
				(0.031) ^b^
**lnOPL**	0.086***	0.068	0.188***^b^	0.102***
	(0.012) ^b^	(0.122) ^b^	(0.022) ^b^	(0.019) ^b^
**TRE**	0.207***	0.058***	0.015	0.068***
	(0.018) ^b^	(0.014) ^b^	(0.012) ^b^	(0.006) ^b^
**EX**	-0.030	-0.765***	-0.030**	-0.058**
	(0.048) ^b^	(0.019) ^b^	(0.012) ^b^	(0.027) ^b^
**lnPGDP**	0.080***	0.218***	0.108***	0.229***
	(0.007) ^b^	(0.004) ^b^	(0.005) ^b^	(0.009) ^b^
**Observations**	2620	2620	2620	2620
**AR (1)**	0.021	0.026	0.023	0.039
**AR (2)**	0.274	0.302	0.264	0.301
**Controls**	Yes	Yes	Yes	Yes
**Hansen test**	131.00	132.30	116.26	131.74
**P-value of Hansen test**	0.899	0.997	0.872	0.998

^a^L. denotes the first order lag of the variable, the same below.

^b^the values in the brackets are the standard errors of the coefficients, the same below.

**Table 6:** The impact of different types of CRs on TE.

Variable	lnCR	lnPR	LnER	lnFR
**L.lnTE**	0.988***	0.978***	1.007***	0.969***
	(0.002)	(0.013)	(0.021)	(0.029)
**lnCR**	-0.222***			
	(0.027)			
**lnPR**		-0.780***		
		(0.170)		
**lnER**			-0.999***	
			(0.306)	
**lnFR**				-0.953***
				(0.225)
**lnOPL**	0.232***	0.240	0.437**	0.225*^a^
	(0.007)	(0.146)	(0.175)	(0.128)
**TRE**	0.020*^a^	0.051***	0.015	0.596
	(0.012)	(0.017)	(0.027)	(1.515)
**EX**	-0.468***	-0.139	-0.054	-0.195
	(0.048)	(0.181)	(0.204)	(1.089)
**lnPGDP**	0.073***	0.131***	0.145***	0.111***
	(0.004)	(0.035)	(0.044)	(0.033)
**Observations**	2620	2620	2620	2620
**AR (1)**	0.002	0.002	0.001	0.003
**AR (2)**	0.501	0.675	0.432	0.548
**Controls**	Yes	Yes	Yes	Yes
**Hansen test**	118.89	124.42	127.26	126.03
**P-value of Hansen**	0.998	0.979	0.999	0.891

^a^* denotes the rejection of null hypotheses at 10% significance level, the same below.

**Table 7:** The impact of different types of CRs on TA.

Variable	lnCR	lnPR	LnER	lnFR
**L.lnTA**	0.887***	0.933***	0.883***	0.907***
	(0.042)	(0.032)	(0.006)	(0.049)
**lnCR**	-0.631**			
	(0.303)			
**lnPR**		-0.726***		
		(0.035)		
**lnER**			-0.448***	
			(0.037)	
**lnFR**				-0.699**
				(0.299)
**lnOPL**	0.236	0.786**	0.391***	0.021
	(0.389)	(0.374)	(0.055)	(0.628)
**TRE**	0.707	0.493	0.929***^b^	0.442
	(0.565)	(0.368)	(0.199)	(0.863)
**EX**	-0.283	-0.453	-0.468	-0.777**
	(0.321)	(0.810)	(0.302)	(0.382)
**lnPGDP**	0.327*	0.352	0.632***	0.626***
	(0.168)	(0.284)	(0.015)	(0.144)
**Observations**	2620	2620	2620	2620
**AR (1)**	0.002	0.002	0.001	0.001
**AR (2)**	0.330	0.134	0.448	0.329
**Controls**	Yes	Yes	Yes	Yes
**Hansen test**	124.97	126.50	133.93	129.09
**P-value of Hansen**	0.929	0.999	0.997	0.988

**Table 8:** The impact of CR on international tourism in different time periods.

Variable	2000–2007			2008–2019		
	lnTR	lnTE	lnTA	lnTR	lnTE	lnTA
**L.lnTR**	1.026***			0.982***		
	(0.035)			(0.186)		
**L.lnTE**		0.898***			1.010***	
		(0.080)			(0.021)	
**L.lnTA**			1.023***			0.781***
			(0.045)			(0.108)
**lnCR**	-0.481***	-0.337***	-0.302***	-0.442**	-0.577*	-0.169**
	(0.014)	(0.108)	(0.050)	(0.209)	(0.307)	(0.075)
**lnOPL**	0.397	0.552*	0.309**	0.040	0.159*	0.365*
	(0.398)	(0.308)	(0.121)	(0.083)	(0.091)	(0.193)
**TRE**	0.292	0.051	0.186	0.019	0.001	0.794
	(0.261)	(0.515)	(0.260)	(0.312)	(0.294)	(0.529)
**EX**	-0.656	-0.123	-0.079	-0.153	-0.184	-0.070
	(1.129)	(1.465)	(0.346)	(0.560)	(0.487)	(0.698)
**lnPGDP**	0.819***	0.683***	0.346**	0.044	0.070	0.291**
	(0.182)	(0.254)	(0.140)	(0.038)	(0.046)	(0.146)
**Observations**	854	854	854	1479	1479	1479
**AR (1)**	0.001	0.001	0.002	0.003	0.002	0.008
**AR (2)**	0.381	0.259	0.974	0.284	0.200	0.216
**Controls**	Yes	Yes	Yes	Yes	Yes	Yes
**Hansen test**	15.20	25.04	14.16	70.07	87.44	46.39
**P-value of Hansen**	0.413	0.245	0.863	0.153	0.809	0.884

**Table 9:** The impact of CR on tourism in different regions.

Variable	Asia-Pacific			Europe			Africa			America		
	lnTR	lnTE	lnTA	lnTR	lnTE	lnTA	lnTR	lnTE	lnTA	lnTR	lnTE	lnTA
**L.lnTR**	0.897***			0.896***			0.951***			1.167***		
	(0.046)			(0.121)			(0.071)			(0.136)		
**L.lnTE**		0.964***			1.006***			0.954***			1.109***	
		(0.055)			(0.098)			(0.138)			(0.135)	
**L.lnTA**			0.101***			0.973***			0.877***			0.926***
			(0.016)			(0.062)			(0.101)			(0.071)
**lnCR**	-0.420***	-0.398***	-0.461***	-0.304***	-0.395**	-0.291***	-0.753**	-0.498*	-0.132*	-0.403*	-0.471***	-0.599***
	(0.095)	(0.106)	(0.097)	(0.110)	(0.162)	(0.101)	(0.342)	(0.279)	(0.074)	(0.206)	(0.148)	(0.177)
**lnOPL**	0.039***	0.025**	0.342***	0.037*	0.054*	0.022	0.122	0.203***	0.115	0.136	0.064	0.544***
	(0.011)	(0.011)	(0.037)	(0.021)	(0.029)	(0.024)	(0.175)	(0.075)	(0.166)	(0.106)	(0.143)	(0.085)
**TRE**	0.067	0.036***	0.039	0.330	0.364	0.299***	0.048	0.047	0.172	0.060	0.267	0.561*
	(0.073)	(0.007)	(0.057)	(0.204)	(0.247)	(0.093)	(0.104)	(0.080)	(0.380)	(0.051)	(0.252)	(0.328)
**EX**	-0.058***	-0.390***	-0.182***	-0.358***	-0.207*	-0.033	-0.489	-0.077	-0.119*	-0.155	-0.042	-0.107
	(0.011)	(0.053)	(0.015)	(0.117)	(0.124)	(0.089)	(0.708)	(0.253)	(0.063)	(0.180)	(0.452)	(0.102)
**lnPGDP**	0.247***	0.246***	0.113**	0.312***	0.368**	0.038	0.116	0.054	0.442***	0.137	0.424*	0.526***
	(0.037)	(0.049)	(0.050)	(0.087)	(0.182)	(0.035)	(0.114)	(0.149)	(0.149)	(0.120)	(0.253)	(0.056)
**Observations**	660	660	660	722	722	722	646	646	646	513	513	513
**AR (1)**	0.005	0.002	0.003	0.001	0.001	0.002	0.003	0.001	0.002	0.004	0.003	0.002
**AR (2)**	0.436	0.491	0.939	0.584	0.265	0.643	0.392	0.491	0.524	0.819	0.319	0.671
**Controls**	Yes	Yes	Yes	Yes	Yes	Yes	Yes	Yes	Yes	Yes	Yes	Yes
**Hansen test**	31.16	29.83	30.89	24.24	31.74	31.17	42.98	40.91	29.91	30.92	32.01	33.01
**P-value of Hansen**	0.989	0.911	0.838	0.789	0.893	0.921	0.991	0.891	0.939	0.918	0.819	0.817

**Table 10:** The impact of CR on tourism in countries with different income levels.

Variable	High-income countries			Middle-income countries			Low-income countries		
	lnTR	lnTE	lnTA	lnTR	lnTE	lnTA	lnTR	lnTE	lnTA
**L.lnTR**	1.017***			0.939***			0.926***		
	(0.015)			(0.014)			(0.071)		
**L.lnTE**		0.999***			0.975***			0.883***	
		(0.022)			(0.052)			(0.224)	
**L.lnTA**			0.909***			0.972***			0.943*
			(0.026)			(0.010)			(0.492)
**lnCR**	-0.261***	-0.291***	-0.195***	-0.302***	-0.340**	-0.229***	-0.599***	-0.653***	-0.704*
	(0.078)	(0.062)	(0.061)	(0.059)	(0.170)	(0.005)	(0.177)	(0.178)	(0.395)
**lnOPL**	0.010	0.007	0.091***	0.021	0.165**	0.154***	0.544***	0.103	0.168
	(0.012)	(0.010)	(0.027)	(0.030)	(0.078)	(0.008)	(0.085)	(0.370)	(0.741)
**TRE**	0.135*	0.049	0.037	0.461***	0.154	0.092**	0.561*	0.009	0.786
	(0.080)	(0.112)	(0.045)	(0.063)	(0.229)	(0.045)	(0.328)	(0.647)	(1.110)
**EX**	-0.002	-0.054	-0.336***	-0.003	-0.006	-0.035	-0.107	-0.187	-0.500
	(0.047)	(0.056)	(0.069)	(0.009)	(0.034)	(0.023)	(0.182)	(0.824)	(0.398)
**lnPGDP**	0.006	0.124***	0.195***	0.099***	0.169**	0.001	0.516***	0.029	0.351
	(0.039)	(0.041)	(0.029)	(0.027)	(0.071)	(0.010)	(0.056)	(0.077)	(0.221)
**Observations**	988	988	988	1207	1207	1207	332	332	332
**AR (1)**	0.006	0.005	0.003	0.002	0.002	0.025	0.012	0.004	0.038
**AR (2)**	0.198	0.541	0.330	0.532	0.294	0.364	0.664	0.588	0.128
**Controls**	Yes	Yes	Yes	Yes	Yes	Yes	Yes	Yes	Yes
**Hansen test**	43.67	40.91	45.03	59.30	59.71	55.66	3.33	3.94	2.07
**P-value of Hansen**	0.998	0.340	0.997	0.917	0.978	0.910	0.998	0.999	0.998

### The impact of different risk types on TR

Table 5 estimates the impact of CR and different risk types (i.e., PR, ER, and FR) on TR. Table 5 shows that the impact of lnCR on lnTR is significant at 1% significance level, with an impact coefficient of -0.307, indicating that CR has a significant negative impact on TR, and 1% increase in lnCR leads to 0.307% reduction in lnTR. This result is consistent with the conclusion of Lee et al., (2020). In addition, lnPR, lnER and lnFR all have a significant negative impact on TR, with lnFR having the greatest Influence coefficient (-0.568), followed by lnER (-0.352) and lnPR (-0.291).

With the deepening of economic globalization, FR presents an increasingly deep and continuous influence on the global economy, including TR [8]. In comparison, PR and ER have a smaller impact on tourism [95]. In addition, L.lnTR shows a significant influence, indicating that TR has a significant lag effect, which means that CR has a long-term and lasting impact on TR. This is also consistent with the tourism destination management theory, which believes that any adverse changes in the tourism destination have a long-term impact on tourism [101].

### The impact of different risk types on TE

Table 6 estimates the impact of different risk types (i.e., CR, PR, ER, and FR) on TE. The results in Table 6 show that there is a significant negative correlation between lnCR and lnTE, and every 1% increase in lnCR leads to a 0.222% decrease in lnTE, which indicates that CR lowers TE (Bernini et al., 2015). Furthermore, lnPR, lnER and lnFR all show a significant negative influence on lnTE, with lnFR (-0.953), and lnER (-0.999) having a greater impact than lnPR (-0.780). This finding echoes the study result of Alrawadieh, Alrawadieh [57], which contends that more residents choose to save money during financial and economic risks, thereby reducing travel frequency and TE. Additionally, L.lnTE presents a significant impact, indicating that TE also has an obvious time lag effect. Therefore, the impact of CRs on TE is long-term, which also conforms to the sustainability of tourism influence [102].

### The impact of different risk types on TA

Table 7 demonstrates the impact of CR, PR, ER, and FR on TA. The results show that lnCR has a significant negative impact on lnTA, with every 1% increase in lnCR reducing lnTA by 0.631%. Different risk types are also significantly negatively correlated with TA, with lnPR showing the greatest influence (-0.726), followed by lnFR (-0.699) and lnER (-0.448). Travels are not the basic need of residents, which are relatively flexible and vulnerable to external risks. Therefore, potential tourists will give up travel plans when facing risks. In addition, TA presents a significant time lag effect, which confirms that risks have a long-term impact on TA and influence tourists’ travel decisions through their perception [103].

### The impact of CR on tourism in different time periods

The outbreak of the financial crisis in 2008 had a great impact on the global economy and increased the uncertainty of international tourism. Therefore, this paper uses 2008 as a breakpoint to estimate the impact of CRs on tourism during 2000–2007 and 2008–2019. The results are shown in Table 8. It can be seen that before the financial crisis (2000–2007), the impact of lnCR on lnTR, lnTE, and lnTA were all significantly negative, with the largest impact on lnTR (-0.481), followed by lnTE (-0.337), and lnTA (-0.302), indicating that TR is most sensitive to CR before 2008. After the financial crisis (2008–2019), lnCR also presented a significant negative impact on lnTR, lnTE, and lnTA, with lnTE being mostly affected by lnCR (-0.577), followed by lnTR (-0.442) and lnTA (-0.169), demonstrating that TE was more fragile to CR after 2008. In addition, there are time lag effects in lnTR, lnTE, and lnTA in different time periods. Regarding the impact coefficients, CR has a greater impact on TR and TA before 2008 and exerts a larger influence on TE after 2008.

### The impact of CR on tourism in different regions

Different regions face different CRs and have different capacities to resist them. This paper explores the impact of CR on tourism in different regions, and the results are shown in Table 9. It can be seen that the effects of lnCR on lnTR, lnTE, and lnTA are all significantly negative and vary across different regions, which confirms the adverse impact of CR on international tourism. Specifically, in the Asia-Pacific region, lnCR has the greatest impact on lnTA (-0.461), followed by lnTR (-0.420) and lnTE (-0.398). In European region, lnCR has the greatest impact on lnTE (-0.395), followed by lnTR (-0.304) and lnTA (-0.291). In African countries, lnCR has the greatest impact on lnTR (-0.753), followed by lnTE (-0.498) and lnTA (-0.132). For American countries, lnCR has the greatest impact on lnTA (-0.599), followed by lnTE (-0.471) and lnTR (-0.403). From the impact coefficients, CR displays a larger effect on international tourism in African American and Asia-Pacific regions and the smallest influence on the European region, suggesting that CR’s influence tends to be correlated with the overall development level of a region. In addition, CR has the greatest impact on TA in the American and Asia-Pacific countries, TE, and TR in the African region.

### The impact of CR on tourism in countries with different income levels

Table 10 presents the impact of CR on tourism in countries with different income levels. The results show that in high-income countries, lnCR has a greater impact on lnTE (-0.291) and lnTR (-0.261), and has a smaller impact on lnTA (-0.195). In middle-income countries, the impact coefficients of lnCR on lnTR, lnTE, and lnTA are -0.302, -0.340, and -0.229, respectively. In low-income countries, lnCR has the largest influence on lnTA (-0.704), followed by lnTE (-0.653) and lnTR (-0.599). Within the income groups, CR has a greater impact on TR and TE in high-income countries and middle-income countries, but have a greater impact on TA in low-income countries. Among different income groups, CR shows the greatest impact on the tourism indicators of low-income countries, followed by the tourism indicators of middle-income countries and high-income countries, demonstrating that the effects of CR on international tourism decrease with the increase in income.

### Robustness test

This paper examines the robustness of estimation results through different indicators, data samples, and estimation methods. Firstly, three dependent variables (i.e., TR, TE, TA) and four independent variables (i.e., CR, ER, PR, FR) are employed to confirm the impact of CRs on international tourism. By replacing the core variables, the robustness of the results is enhanced. Secondly, this study explores the heterogeneity of the relationship between variables in different time periods, different regions, and countries with different income levels, which enriches the research perspectives and enhances the robustness of the estimation results.

To further verify the robustness of the estimated results, generalized least squares (GLS) and difference generalized moment estimation (DIF-GMM) are utilized to test the influence of CR on TR, TE, and TA (seen in Table 11). The results show that the models fit the data well, and CR significantly negatively affects TR, TE, and TA in the GLS model and the DIF-GMM model, which is consistent with the estimation results of SYS-GMM model.

**Table 11:** The impact of CR on international tourism using different methods.

Variable	TR		TE		TA	
	GLS^a^	DIF-GMM	GLS^a^	DIF-GMM	GLS^a^	DIF-GMM
**lnTR**		0.687***				
		(0.183)				
**lnTE**				0.691***		
				(0.059)		
**lnTA**						0.675***
						(0.137)
**lnCR**	-0.314***	-0.450***	-0.357**	-0.745**	-0.961**	-0.555**
	(0.112)	(0.168)	(0.159)	(0.304)	(0.441)	(0.220)
**lnOPL**	0.341	0.434	0.031	0.259	0.167	0.566**
	(0.553)	(0.378)	(0.393)	(0.507)	(0.798)	(0.225)
**TRE**	0.353	0.156**	0.050	0.112**	0.180	0.006
	(0.314)	(0.065)	(0.361)	(0.053)	(0.225)	(0.996)
**lnEX**	-0.028	-0.025	-0.492*	-0.060	-0.493	-0.763
	(0.425)	(0.043)	(0.271)	(0.065)	(0.555)	(0.503)
**lnPGDP**	0.045***	0.038**	0.078***	0.023	0.432***	0.492***
	(0.014)	(0.016)	(0.018)	(0.018)	(0.124)	(0.148)
**Observations**	2760	2320	2760	2320	2760	2360
**R2**	0.329		0.331		0.530	
**AR (1)**		0.079		0.001		0.002
**AR (2)**		0.333		0.717		0.235
**Controls**	Yes	Yes	Yes	Yes	Yes	Yes
**Hansen test**		128.38		133.17		130.57
**P-value of Hansen**		0.984		0.967		0.978

^a^ Fixed effects model was used to conduct the generalized least square analysis (GLS).

### Moderating effects

The regression results of the moderating effect of informatization between CR and international tourism are seen in Table 12. In the table, columns (1) to (3) present the impact of CR and INF on TR, TE, and TA, (4) to (6) exhibit the influence of PR and INF on TR, TE, and TA, (7) to (9) show the effects of ER and INF on TR, TE and TA, and (10) to (12) display the impact of FR and INF on TR, TE, and TA. The results demonstrate that the effects of lnCR, lnPR, lnER, and lnFR on lnTR, lnTE, and lnTA are all negative, while lnINF, lnCR*INF, lnPR*INF, lnER*INF, and lnFR*INF all show a positive influence, indicating that informatization not only promotes international tourism directly, but also alleviate the adverse impact of CR on international tourism.

**Table 12:** The moderating effects of informatization.

Variable	CR			PR			ER			FR		
	(1)	(2)	(3)	(4)	(5)	(6)	(7)	(8)	(9)	(10)	(11)	(12)
	lnTR	lnTE	lnTA	lnTR	lnTE	lnTA	lnTR	lnTE	lnTA	lnTR	lnTE	lnTA
**L. lnTR**	0.965***			0.958***			0.973***			0.972***		
	(0.021)			(0.015)			(0.167)			(0.019)		
**L. lnTE**		0.981***			0.949***			0.976***			0.968***	
		(0.024)			(0.023)			(0.020)			(0.017)	
**L. lnTA**			0.993***			0.832***			0.893***			0.948***
			(0.029)			(0.054)			(0.049)			(0.013)
**lnCR/lnPR/lnER/lnFR**	-0.237*	-0.210**	-0.533***	-0.272**	-0.488***	-0.395***	-0.242*	-0.184***	-0.407*	-0.568**	-0.943**	-0.528**
	(0.130)	(0.095)	(0.197)	(0.103)	(0.169)	(0.018)	(0.127)	(0.052)	(0.227)	(0.231)	(0.385)	(0.239)
**lnINF**	0.480***	0.799***	0.579**	0.480**	0.481***	0.022**	0.777**	0.081***	0.048***	0.133*	0.106**	0.092*
	(0.148)	(0.125)	(0.279)	(0.206)	(0.165)	(0.011)	(0.381)	(0.023)	(0.013)	(0.079)	(0.049)	(0.050)
lnCR*INF	0.085*	0.090**	0.079**									
	(0.047)	(0.037)	(0.033)									
lnPR*INF				0.184**	0.119***	0.077**						
				(0.088)	(0.008)	(0.035)						
lnER*INF							0.035*	0.197***	0.154**			
							(0.019)	(0.057)	(0.068)			
lnFR*INF										0.515***	0.132*	0.075**
										(0.186)	(0.071)	(0.035)
**lnOPL**	0.016	0.062***	0.092***	0.024	0.069	0.138	0.602	0.029*	0.061	0.010	0.001	0.012
	(0.020)	(0.023)	(0.027)	(0.047)	(0.055)	(0.216)	(0.411)	(0.015)	(0.140)	(0.021)	(0.017)	(0.102)
**TRE**	0.773*	0.079	0.051	0.519***	0.496**	0.057	0.380**	0.010	0.456	0.322	0.342	0.410
	(0.426)	(0.197)	(0.102)	(0.190)	(0.217)	(0.209)	(0.187)	(0.221)	(0.345)	(0.305)	(0.273)	(0.315)
**EX**	-0.019	-0.001	-0.068	-0.018*	-0.008	-0.205*	-0.654*	-0.605	-0.310***	-0.673	-0.418	-0.098
	(0.033)	(0.017)	(0.101)	(0.010)	(0.014)	(0.108)	(0.370)	(0.444)	(0.098)	(0.545)	(0.368)	(0.378)
**lnPGDP**	0.198***	0.221**	0.201***	0.646***	0.677***	0.442**	0.301***	0.043	0.354	0.059	0.544**	0.071***
	(0.023)	(0.011)	(0.019)	(0.119)	(0.098)	(0.185)	(0.075)	(0.245)	(0.320)	(0.119)	(0.224)	(0.011)
**Observations**	2618	2618	2618	2618	2618	2618	2618	2618	2618	2618	2618	2618
**AR (1)**	0.003	0.001	0.001	0.003	0.002	0.003	0.003	0.001	0.001	0.002	0.004	0.001
**AR (2)**	0.22	0.467	0.228	0.222	0.488	0.421	0.479	0.301	0.319	0.329	0.417	0.313
**Controls**	Yes	Yes	Yes	Yes	Yes	Yes	Yes	Yes	Yes	Yes	Yes	Yes
**Hansen test**	132.94	132.99	124.15	124.12	126.39	125.52	129.78	133.92	127.18	27.21	31.19	17.01
**P-value of Hansen**	0.907	0.998	0.999	0.998	0.999	0.997	0.997	0.819	0.999	0.998	0.919	0.897

In terms of the impact coefficients, INF moderates the influence of CR on TR, TE, and TA by 0.085, 0.090, and 0.079, which suggests that for every 1% negative effects of CR, INF can decrease 0.085% for TR, 0.090% for TE, and 0.079% for TA, indicating that informatization is more effective in alleviating risks for TR and TE than TA. Among the three different dimensions of CR, INF has the greatest moderation effect between FR and TR (0.515), followed by ER and TE (0.197), and PR and TR (0.184), demonstrating that INF can alleviate more than 50% of the adverse impacts of FR on TR, and nearly 20% of the negative influence of ER on TE and PR on TR.

## Conclusions and discussions

Based on the panel data of 138 countries from 2000 to 2019, this paper estimates the impacts of CRs on international tourism, and the moderating role of informatization using the dynamic SYS-GMM model. In the models, CRs include an aggregate indicator of CR, and three dimensions of PR, ER, and FR, and international tourism is proxied by three variables of TR, TE, and TA. In addition, the empirical tests are examined in different time periods, countries in different regions, and countries with different income levels. The main research conclusions are as follows.

CRs show a significant negative impact on international tourism regardless of the time periods, regions, or income levels, indicating the negative relationship between the two variables, which is consistent with the conclusion of Lee and Chen [18]. In terms of impact intensity, the influence of CRs on international tourism is heterogenous across different risk indicators, time periods, regions, and countries.

Firstly, the composite CR presents the greatest impact on TA, followed by TR and TE, indicating that the number of tourists is more affected by CR than tourism receipts and spending. Since TA is the starting point of traveling, it is the easiest to be affected by external risks. CR usually affects TA by adversely impacting destination image [47–49], thereby changing tourists’ attitude towards or perceived control about a planned travel.

Secondly, FR and ER have the greatest impact on TR and TE, while PR affects TA the most. As indicators closely related to money, TR and TE are more affected by financial and economic factors, which has also been confirmed by Chan [67] by studying the effects of global financial markets on the entertainment TR of Macao, and Eugenio-Martin and Campos-Soria [17] through examining the TE cutback decision during the economic crisis. However, PR involves factors such as wars, terrorism, and conflicts, etc., that threaten the lives of tourists, thus having a greater impact on TA. Tiwari, Das [95] have also testified that geopolitical risks have stronger and longer impacts on TA than economic uncertainties. Moreover, the negative impacts of PR on tourist flow have been recognized by previous studies [52, 104].

Thirdly, TR and TA were more sensitive to CR before 2008, while TE was the most vulnerable to CR after 2008. With the increasing awareness of tourism risk management since 2008, destinations’ ability to resist risks has been enhanced. In addition, the popularity of tourists’ personalized psychology in recent years has made some high-risk tourist destinations attractive to tourists, satisfying tourists’ pursuit of rare and new experiences [45]. Therefore, the effect of CR on TR and TA decreased after 2008. However, in the face of crisis, tourist spending seems to have become more conservative after the financial crisis, which is consistent with the conclusion of Eugenio-Martin and Campos-Soria [17].

Fourthly, CR shows a larger impact on international tourism in African, American, and Asia-Pacific regions, while exerting the smallest effect on international tourism in Europe. Furthermore, international tourism in high-income countries is least affected by CR, followed by middle-income countries, while international tourism in low-income countries is most affected by CR. These results indicate that the influence of CR on international tourism is negatively related to the development level of a region or country. A higher development level or income usually indicates stronger support for tourism development, and more mature tourism markets. Therefore, higher-income countries can adopt more diverse measures to recover the tourism industry during risky periods. On the contrary, the tourism industry in low-income countries is restricted by various factors, such as high cost and low availability of capital [105]. As a result, international tourism is more vulnerable to CR in less developed regions or countries. Karabulut, Bilgin [106] also pointed out that the negative impact of the pandemic on TA only lasts in low-income economies because they have weaker abilities to resist crisis.

In addition to the direct relationship between CRs and international tourism, this paper further reveals the significant moderating role of informatization between the two variables. Specifically, informatization exhibits a positive impact on international tourism indicators, uncovering a direct improvement effect of informatization on tourism, which has been supported by Hong, Thakuriah [82], who confirmed that people using the internet travel further, and Wang [107], who asserted that modern information technology facilitates tourism hotel management. Besides, the interaction terms of informatization and CRs display an obvious promoting effect on international indicators, demonstrating a significant moderation function of informatization between CRs and international tourism. Although no research has directly confirmed the positive effect of informatization on resolving the tourism crises, quite a few scholars have pointed out the potential of informatization in this regard by enhancing tourists’ crisis awareness [72] and innovating tourism crisis management models [35, 108]. Among different indicators, informatization presents the greatest moderation effect between CR and TE, followed by CR and TR, and CR and TA. By providing more diversified local-based tourism services and products, as well as more channels for tourism advertising, recommendation, and booking, information technology plays an increasingly important role in promoting the tourism economy [109]. Lorente-Bayona, Gras-Gil [110] has concluded that the wide use of the internet raises international TE. However, as stated above, when facing PR, TA is more related to the personal safety of tourists, which is difficult to be alleviated through informatization.

## Policy and managerial implications

Based on the above research findings, this study proposes the following policy and managerial suggestions.

Firstly, given the widespread negative impacts of CRs on international tourism, risk management measures should be taken by all countries. From the supply side, all the destinations should establish normalized risk early warning mechanisms to prevent or reduce the risks in advance, such as the cooperation between the tourism industry and the emergency management department. In addition, considering the spillover effects of CRs across countries, inter-country collaboration and timely communication are necessary for the whole process of traveling. From the demand side, since tourists’ visit intention is influenced by their risk perception and perceived self-efficacy, techniques targeting tourist perception management could be adopted by destinations, such as publicizing destination security and safety precautions on tourism promotion meetings and official websites.

Secondly, in view of the spatial and temporal heterogeneity of the impacts of CRs on international tourism, different destinations should take targeted measures for different risk types, tourism indicators, and time periods. Specifically, measures to increase TA should be the priority during PR, while policies and movements to stimulate TE and TR are worth the greatest attention during ER and FR. In particular, TE needs to be prevented from contracting significantly after the wake of the economic and financial crisis. Therefore, destinations should adopt sales promotion, consumption vouchers, and other marketing means in time to boost consumer confidence. Furthermore, low-income countries should pay more attention to the precaution and alleviation of CRs since they generally have weaker resistance to external risks. UNWTO and other international tourism organizations should also give more preference to low-income countries regarding policy and financial support during tourism risks.

Lastly, based on the significant moderation effects of informatization between CRs and international tourism, modern information technology can be an effective tool for risk prevention and alleviation. By utilizing the internet and mobile communication technologies, destinations can release risk-related information anytime and anywhere. Therefore, tourism destinations should first build a sound information platform for CR warning and management, including official websites, official Weibo accounts, APPs, WeChat mini programs, and short video accounts, etc. In addition, timely risk warnings and relevant information releases are essential to minimize risk losses, which relies on information technologies to construct smooth information communication channels and orderly information release procedures. Last but not least, destination image restoration after risk needs a wide range of marketing campaigns to reverse tourists’ perception of CRs, highlighting the advantages of modern information technologies.

## Research limitations and future research directions

Overall, this research confirms the negative impact of CRs on international tourism by considering four types of risks and three tourism indicators, reveals the heterogeneous influence in different time periods, regions, and countries, and explores the moderation effects of informatization between the two variables. Although we have explored the relationship between CRs, informatization, and international tourism from multiple dimensions and perspectives, there is still space for further refinement in future research. First, there are five subdimensions of ER and FR, respectively, and 12 subdimensions of PR. Therefore, a deeper exploration into these 22 indicators will provide more detailed insights into the impact of various CR sources on tourism. Second, informatization consists of three subcomponents of informatization infrastructure, informatization applications, and informatization skills. It will be interesting to compare the moderation intensity of different informatization indicators between CRs and tourism. Besides, the current study focuses on the influence of CRs and informatization on international tourism, and other risk types, e.g., geopolitical risks, economic uncertainties, climate change, and pandemics, and other possible moderation mechanisms, e.g., specific risk mitigation measures, have been ignored, which worth further research.

## Supporting information

S1 Table Abbreviations. (DOCX)

S2 Table Data. (DOCX)
