# Supplementary material for: The moderating role of informatization between country risks and international tourism: A cross-country panel analysis
Source: PLoS One. 2022 Dec 16;17(12):e0278518. doi: 10.1371/journal.pone.0278518 (PMC9757600; doi:10.1371/journal.pone.0278518)
Supplement: S1 Table — (DOCX) [file pone.0278518.s001.docx]

**S1 Table. Abbreviations**

| **Number** | **Abbreviations** | **Full name** |
| --- | --- | --- |
| 1 | SYS-GMM | System generalized method of moments |
| 2 | DIF-GMM | Difference generalized method of moments |
| 3 | GLS | Generalized least squares |
| 4 | CR | Country risk |
| 5 | PR | Political risk |
| 6 | ER | Economic risk |
| 7 | FR | Financial risk |
| 8 | TPB | Theory of planned behavior |
| 9 | RPAF | Risk perception attitude framework |
| 10 | BB | Behavioral beliefs |
| 11 | NB | Normative beliefs |
| 12 | CB | Control beliefs |
| 13 | ICT | Information and communication technology |
| 14 | TR | Tourism revenue |
| 15 | TE | Tourism expenditure |
| 16 | TA | Tourist arrival |
| 17 | OPL | Opening-up level |
| 18 | TRE | Tourism resource endowment |
| 19 | EX | Exchange rate |
| 20 | EDL | Economic development level |
| 21 | PGDP | GDP per capita |
| 22 | INF | Informatization |
