# Supplementary material for: The moderating role of informatization between country risks and international tourism: A cross-country panel analysis
Source: PLoS One. 2022 Dec 16;17(12):e0278518. doi: 10.1371/journal.pone.0278518 (PMC9757600; doi:10.1371/journal.pone.0278518)
Supplement: S2 Table — (DOCX) [file pone.0278518.s002.docx]

**S2 Table. Data**

| ID | YEAR | CR | PR | FR | ER | TR | TE | TA | PGDP | OPL | TRE | EX | INF |
| --- | --- | --- | --- | --- | --- | --- | --- | --- | --- | --- | --- | --- | --- |
| 1 | 2000 | 37.65 | 40.58 | 67.54 | 67.17 | 400000000 | 290000000 | 97000 | 1126.68 | 20.12 | 4.00 | 143.71 | 0.11 |
| 1 | 2001 | 37.08 | 39.21 | 67.46 | 67.50 | 450000000 | 270000000 | 117000 | 1281.66 | 21.33 | 4.00 | 143.49 | 0.49 |
| 1 | 2002 | 35.83 | 37.42 | 66.50 | 67.75 | 490000000 | 390000000 | 137000 | 1425.12 | 20.95 | 4.00 | 140.16 | 7.04 |
| 1 | 2003 | 33.19 | 31.96 | 66.29 | 68.13 | 540000000 | 510000000 | 322000 | 1846.12 | 20.85 | 4.00 | 121.86 | 23.63 |
| 1 | 2004 | 34.58 | 34.92 | 66.46 | 67.79 | 760000000 | 670000000 | 507000 | 2373.58 | 22.22 | 4.00 | 102.78 | 3.52 |
| 1 | 2005 | 31.83 | 33.83 | 64.83 | 65.00 | 880000000 | 810000000 | 692000 | 2673.79 | 23.01 | 5.00 | 99.87 | 0.15 |
| 1 | 2006 | 30.54 | 31.96 | 64.63 | 64.50 | 1100000000 | 990000000 | 877000 | 2972.74 | 25.21 | 5.00 | 98.10 | 0.64 |
| 1 | 2007 | 29.1 | 32.33 | 60.50 | 65.38 | 1500000000 | 1300000000 | 1100000 | 3595.04 | 28.25 | 6.00 | 90.43 | 0.02 |
| 1 | 2008 | 27.6 | 31.42 | 59.33 | 64.46 | 1900000000 | 1600000000 | 1200000 | 4370.54 | 25.30 | 7.00 | 83.89 | 17.85 |
| 1 | 2009 | 30.98 | 31.33 | 62.46 | 68.17 | 2000000000 | 1700000000 | 1700000 | 4114.14 | 25.24 | 7.00 | 94.98 | 28.58 |
| 1 | 2010 | 32.25 | 32.79 | 62.21 | 69.50 | 1800000000 | 1500000000 | 2200000 | 4094.35 | 27.98 | 7.00 | 103.94 | 0.11 |
| 1 | 2011 | 33.44 | 34.79 | 62.25 | 69.83 | 1800000000 | 1700000000 | 2500000 | 4437.14 | 29.24 | 8.00 | 100.90 | 33.73 |
| 1 | 2012 | 32.65 | 35.38 | 62.42 | 67.50 | 1600000000 | 1400000000 | 3200000 | 4247.63 | 28.94 | 8.00 | 108.18 | 46.76 |
| 1 | 2013 | 32.87 | 34.33 | 63.79 | 67.63 | 1700000000 | 1600000000 | 2900000 | 4413.06 | 28.92 | 8.00 | 105.67 | 0.84 |
| 1 | 2014 | 32.44 | 32.71 | 64.67 | 67.50 | 1800000000 | 1700000000 | 3300000 | 4578.63 | 28.21 | 8.00 | 105.48 | 8.00 |
| 1 | 2015 | 32.67 | 33.88 | 63.96 | 67.50 | 1600000000 | 1300000000 | 3800000 | 3952.80 | 27.27 | 8.00 | 125.96 | 0.06 |
| 1 | 2016 | 31.12 | 33.29 | 62.83 | 66.13 | 1800000000 | 1300000000 | 4100000 | 4124.06 | 28.98 | 8.00 | 124.14 | 0.75 |
| 1 | 2017 | 31.25 | 32.29 | 64.21 | 66.00 | 2100000000 | 1500000000 | 4600000 | 4531.02 | 31.57 | 9.00 | 119.10 | 6.15 |
| 1 | 2018 | 28.4 | 32.50 | 58.46 | 65.83 | 2300000000 | 1800000000 | 5300000 | 5284.38 | 31.54 | 9.00 | 107.99 | 6.55 |
| 1 | 2019 | 29.83 | 33.67 | 61.17 | 64.83 | 2500000000 | 1900000000 | 6400000 | 5353.24 | 31.56 | 9.00 | 96.88 | 2.87 |
| 2 | 2000 | 43.98 | 55.75 | 66.79 | 65.42 | 270000000 | 500000000 | 866000 | 1764.97 | 42.07 | 7.00 | 75.26 | 1.86 |
| 2 | 2001 | 37.91 | 54.88 | 60.75 | 60.21 | 310000000 | 520000000 | 901000 | 1740.64 | 36.69 | 7.00 | 77.22 | 5.37 |
| 2 | 2002 | 38.21 | 53.58 | 61.29 | 61.54 | 350000000 | 540000000 | 988000 | 1781.84 | 35.50 | 7.00 | 79.68 | 29.43 |
| 2 | 2003 | 34.5 | 54.92 | 57.42 | 56.67 | 390000000 | 500000000 | 1200000 | 2103.41 | 38.25 | 7.00 | 77.40 | 44.47 |
| 2 | 2004 | 28.62 | 46.29 | 55.08 | 55.88 | 440000000 | 580000000 | 1200000 | 2609.95 | 40.05 | 7.00 | 72.06 | 7.29 |
| 2 | 2005 | 22.81 | 36.79 | 53.38 | 55.46 | 480000000 | 660000000 | 1400000 | 3113.10 | 47.20 | 7.00 | 73.28 | 1.08 |
| 2 | 2006 | 22.08 | 36.71 | 52.54 | 54.92 | 390000000 | 410000000 | 1600000 | 3478.65 | 48.81 | 7.00 | 72.65 | 2.90 |
| 2 | 2007 | 21.96 | 37.58 | 51.25 | 55.08 | 330000000 | 500000000 | 1700000 | 3950.52 | 47.07 | 7.00 | 69.29 | 0.08 |
| 2 | 2008 | 22.75 | 38.29 | 51.00 | 56.21 | 470000000 | 610000000 | 1800000 | 4923.63 | 47.97 | 7.00 | 64.58 | 39.17 |
| 2 | 2009 | 26.6 | 39.33 | 52.71 | 61.17 | 360000000 | 570000000 | 1900000 | 3883.26 | 35.37 | 7.00 | 72.65 | 30.22 |
| 2 | 2010 | 28.1 | 39.04 | 52.42 | 64.75 | 320000000 | 720000000 | 2100000 | 4480.80 | 38.44 | 7.00 | 74.39 | 0.80 |
| 2 | 2011 | 28.1 | 43.21 | 51.25 | 61.75 | 300000000 | 600000000 | 2400000 | 5455.85 | 38.79 | 7.00 | 72.94 | 3.70 |
| 2 | 2012 | 28.29 | 44.38 | 51.38 | 60.83 | 300000000 | 600000000 | 2600000 | 5592.26 | 36.89 | 7.00 | 77.54 | 1.98 |
| 2 | 2013 | 28.17 | 44.08 | 51.46 | 60.79 | 330000000 | 530000000 | 2700000 | 5499.58 | 33.21 | 7.00 | 79.37 | 1.46 |
| 2 | 2014 | 28.98 | 42.88 | 52.83 | 62.25 | 320000000 | 680000000 | 2300000 | 5493.03 | 30.22 | 7.00 | 80.58 | 14.31 |
| 2 | 2015 | 34.52 | 42.33 | 58.88 | 67.83 | 350000000 | 760000000 | 1700000 | 4177.89 | 23.17 | 7.00 | 100.69 | 1.98 |
| 2 | 2016 | 38.23 | 43.79 | 59.25 | 73.42 | 250000000 | 560000000 | 2000000 | 3946.44 | 20.87 | 7.00 | 109.44 | 37.25 |
| 2 | 2017 | 38.21 | 44.63 | 58.88 | 72.92 | 170000000 | 630000000 | 2500000 | 4044.28 | 22.66 | 7.00 | 110.97 | 0.92 |
| 2 | 2018 | 36.48 | 44.79 | 57.54 | 70.63 | 200000000 | 540000000 | 2700000 | 4114.71 | 25.62 | 7.00 | 116.59 | 0.03 |
| 2 | 2019 | 36.6 | 46.83 | 56.13 | 70.25 | 140000000 | 670000000 | 2400000 | 3973.96 | 22.79 | 7.00 | 122.22 | 0.01 |
| 3 | 2000 | 30.15 | 27.42 | 71.38 | 61.50 | 3200000000 | 5500000000 | 2900000 | 7708.10 | 10.99 | 8.00 | 1.00 | 2.21 |
| 3 | 2001 | 31.31 | 27.29 | 71.21 | 64.13 | 2800000000 | 4900000000 | 2600000 | 7208.37 | 11.58 | 8.00 | 1.00 | 5.80 |
| 3 | 2002 | 48.06 | 40.83 | 82.29 | 73.00 | 1700000000 | 2700000000 | 2800000 | 2593.40 | 28.38 | 8.00 | 3.06 | 0.54 |
| 3 | 2003 | 41.02 | 39.21 | 76.08 | 66.75 | 2300000000 | 3000000000 | 3000000 | 3349.81 | 25.93 | 9.00 | 2.90 | 6.61 |
| 3 | 2004 | 32.6 | 35.08 | 70.58 | 59.54 | 2700000000 | 3200000000 | 3500000 | 4277.72 | 23.85 | 9.00 | 2.92 | 0.67 |
| 3 | 2005 | 29.92 | 31.04 | 67.88 | 60.92 | 3200000000 | 3600000000 | 3800000 | 5109.85 | 23.25 | 9.00 | 2.90 | 0.23 |
| 3 | 2006 | 25.4 | 29.00 | 62.42 | 59.38 | 3900000000 | 4000000000 | 4200000 | 5919.01 | 23.03 | 9.00 | 3.05 | 44.70 |
| 3 | 2007 | 25.31 | 29.33 | 62.29 | 59.00 | 5000000000 | 5100000000 | 4600000 | 7245.45 | 22.66 | 9.00 | 3.10 | 43.98 |
| 3 | 2008 | 28.42 | 33.00 | 62.67 | 61.17 | 5300000000 | 6000000000 | 4700000 | 9020.87 | 22.06 | 9.00 | 3.14 | 18.35 |
| 3 | 2009 | 33.6 | 35.13 | 65.33 | 66.75 | 4500000000 | 5800000000 | 4300000 | 8225.14 | 19.56 | 9.00 | 3.71 | 1.20 |
| 3 | 2010 | 29.94 | 36.00 | 60.88 | 63.00 | 5600000000 | 6400000000 | 6800000 | 10386.00 | 18.93 | 9.00 | 3.90 | 0.10 |
| 3 | 2011 | 26.12 | 33.54 | 58.83 | 59.88 | 6100000000 | 7600000000 | 6700000 | 12848.90 | 18.45 | 9.00 | 4.11 | 0.23 |
| 3 | 2012 | 29 | 34.79 | 59.83 | 63.38 | 5700000000 | 8500000000 | 6500000 | 13082.70 | 16.24 | 9.00 | 4.54 | 51.30 |
| 3 | 2013 | 32.46 | 38.08 | 62.17 | 64.67 | 5200000000 | 8400000000 | 6500000 | 13080.30 | 14.62 | 9.00 | 5.46 | 0.15 |
| 3 | 2014 | 36.33 | 37.92 | 65.92 | 68.83 | 5600000000 | 7800000000 | 7200000 | 12334.80 | 14.41 | 10.00 | 8.08 | 1.22 |
| 3 | 2015 | 33.85 | 35.83 | 61.79 | 70.08 | 5400000000 | 9300000000 | 6800000 | 13789.10 | 10.71 | 10.00 | 9.23 | 9.78 |
| 3 | 2016 | 36.12 | 33.21 | 67.38 | 71.67 | 5500000000 | 12000000000 | 6700000 | 12790.20 | 12.53 | 11.00 | 14.76 | 0.40 |
| 3 | 2017 | 31.69 | 32.29 | 61.13 | 69.96 | 5800000000 | 14000000000 | 6700000 | 14591.90 | 11.24 | 12.00 | 16.56 | 0.25 |
| 3 | 2018 | 36.35 | 34.38 | 68.50 | 69.83 | 6000000000 | 13000000000 | 6900000 | 11683.90 | 14.28 | 12.00 | 28.10 | 4.86 |
| 3 | 2019 | 38.6 | 32.88 | 69.29 | 75.04 | 5700000000 | 9800000000 | 7400000 | 9912.28 | 17.44 | 12.00 | 39.63 | 0.23 |
| 4 | 2000 | 20.92 | 28.00 | 57.79 | 56.04 | 1100000000 | 3000000000 | 3900000 | 33291.40 | 48.82 | 0.00 | 3.67 | 6.73 |
| 4 | 2001 | 16.37 | 23.25 | 55.00 | 54.50 | 1200000000 | 3300000000 | 4100000 | 31280.80 | 49.16 | 0.00 | 3.67 | 6.64 |
| 4 | 2002 | 18.12 | 23.50 | 56.79 | 55.96 | 1300000000 | 3700000000 | 5400000 | 31567.50 | 49.50 | 0.00 | 3.67 | 0.32 |
| 4 | 2003 | 16.23 | 22.58 | 55.67 | 54.21 | 1400000000 | 4000000000 | 5900000 | 33499.10 | 55.92 | 0.00 | 3.67 | 6.32 |
| 4 | 2004 | 16.19 | 22.42 | 55.92 | 54.04 | 1600000000 | 4500000000 | 6200000 | 36333.30 | 63.57 | 0.00 | 3.67 | 7.95 |
| 4 | 2005 | 15.67 | 21.04 | 55.83 | 54.46 | 3200000000 | 6200000000 | 7100000 | 39365.40 | 67.59 | 0.00 | 3.67 | 6.43 |
| 4 | 2006 | 14.62 | 21.50 | 55.08 | 52.67 | 5000000000 | 8800000000 | 8100000 | 41907.40 | 68.63 | 0.00 | 3.67 | 0.19 |
| 4 | 2007 | 15.33 | 20.67 | 57.33 | 52.67 | 6100000000 | 11000000000 | 9000000 | 41809.50 | 72.38 | 0.00 | 3.67 | 22.89 |
| 4 | 2008 | 16.79 | 20.92 | 58.79 | 53.88 | 7200000000 | 13000000000 | 9900000 | 44498.90 | 78.87 | 1.00 | 3.67 | 3.61 |
| 4 | 2009 | 21.15 | 21.29 | 60.17 | 60.83 | 7400000000 | 10000000000 | 11000000 | 32024.20 | 79.65 | 1.00 | 3.67 | 0.20 |
| 4 | 2010 | 20.17 | 20.67 | 61.17 | 58.50 | 8600000000 | 12000000000 | 12000000 | 33893.30 | 82.24 | 2.00 | 3.67 | 13.11 |
| 4 | 2011 | 17.62 | 22.25 | 59.50 | 53.50 | 9200000000 | 13000000000 | 13000000 | 39194.70 | 90.08 | 2.00 | 3.67 | 0.13 |
| 4 | 2012 | 17.44 | 22.13 | 60.25 | 52.50 | 11000000000 | 15000000000 | 14000000 | 40976.50 | 100.25 | 2.00 | 3.67 | 21.38 |
| 4 | 2013 | 17.25 | 22.29 | 59.75 | 52.46 | 12000000000 | 16000000000 | 15000000 | 42412.60 | 100.55 | 2.00 | 3.67 | 0.14 |
| 4 | 2014 | 17.02 | 22.25 | 59.96 | 51.83 | 15000000000 | 16000000000 | 16000000 | 43751.80 | 99.56 | 3.00 | 3.67 | 43.08 |
| 4 | 2015 | 20.92 | 23.42 | 61.13 | 57.29 | 17000000000 | 17000000000 | 17000000 | 38663.40 | 100.87 | 4.00 | 3.67 | 1.26 |
| 4 | 2016 | 22.06 | 24.08 | 61.04 | 59.00 | 19000000000 | 17000000000 | 19000000 | 38141.80 | 101.00 | 4.00 | 3.67 | 0.07 |
| 4 | 2017 | 21.42 | 24.50 | 60.58 | 57.75 | 21000000000 | 18000000000 | 20000000 | 40644.80 | 99.59 | 4.00 | 3.67 | 3.08 |
| 4 | 2018 | 21.5 | 24.50 | 61.00 | 57.50 | 21000000000 | 18000000000 | 21000000 | 43839.40 | 93.05 | 5.00 | 3.67 | 0.00 |
| 4 | 2019 | 20 | 24.50 | 60.25 | 55.25 | 38000000000 | 33000000000 | 22000000 | 43103.30 | 92.46 | 5.00 | 3.67 | 1.28 |
| 5 | 2000 | 21.75 | 24.00 | 60.00 | 59.50 | 380000000 | 630000000 | 416000 | 8601.25 | 53.69 | 3.00 | 0.38 | 0.69 |
| 5 | 2001 | 19.25 | 24.04 | 57.83 | 56.63 | 540000000 | 700000000 | 491000 | 8475.96 | 51.70 | 3.00 | 0.38 | 0.11 |
| 5 | 2002 | 21.96 | 25.25 | 58.75 | 59.92 | 540000000 | 700000000 | 566000 | 8626.98 | 49.10 | 3.00 | 0.38 | 5.08 |
| 5 | 2003 | 19.73 | 24.33 | 57.38 | 57.75 | 550000000 | 800000000 | 641000 | 9066.35 | 49.76 | 3.00 | 0.38 | 1.64 |
| 5 | 2004 | 20.04 | 24.25 | 57.25 | 58.58 | 600000000 | 820000000 | 716000 | 10126.10 | 51.38 | 3.00 | 0.38 | 5.35 |
| 5 | 2005 | 17.37 | 23.42 | 55.96 | 55.38 | 630000000 | 860000000 | 891000 | 12377.10 | 58.28 | 3.00 | 0.38 | 0.98 |
| 5 | 2006 | 17.25 | 23.50 | 56.50 | 54.50 | 750000000 | 890000000 | 1100000 | 14420.50 | 56.17 | 4.00 | 0.38 | 0.04 |
| 5 | 2007 | 16 | 23.00 | 55.50 | 53.50 | 910000000 | 950000000 | 1200000 | 15838.50 | 56.48 | 4.00 | 0.38 | 0.06 |
| 5 | 2008 | 17.12 | 23.75 | 56.00 | 54.50 | 1100000000 | 1200000000 | 1400000 | 22139.60 | 58.48 | 4.00 | 0.38 | 52.00 |
| 5 | 2009 | 19.29 | 25.50 | 53.92 | 59.17 | 1100000000 | 1300000000 | 1500000 | 16823.80 | 50.64 | 4.00 | 0.38 | 16.43 |
| 5 | 2010 | 18.81 | 26.00 | 54.38 | 57.25 | 1100000000 | 1200000000 | 1400000 | 18757.00 | 67.49 | 4.00 | 0.38 | 29.99 |
| 5 | 2011 | 20.75 | 31.00 | 54.50 | 56.00 | 1500000000 | 1500000000 | 1000000 | 20921.10 | 72.83 | 4.00 | 0.38 | 45.69 |
| 5 | 2012 | 17.04 | 27.79 | 53.00 | 53.29 | 1700000000 | 1600000000 | 1200000 | 21902.60 | 71.56 | 4.00 | 0.38 | 47.10 |
| 5 | 2013 | 17.5 | 27.50 | 53.75 | 53.75 | 1900000000 | 1800000000 | 1400000 | 20926.60 | 75.38 | 4.00 | 0.38 | 1.18 |
| 5 | 2014 | 18.85 | 27.88 | 54.50 | 55.33 | 2000000000 | 2100000000 | 1600000 | 20131.90 | 70.25 | 4.00 | 0.38 | 12.30 |
| 5 | 2015 | 22.46 | 27.50 | 55.79 | 61.63 | 2200000000 | 2200000000 | 1900000 | 16028.80 | 57.14 | 4.00 | 0.38 | 0.12 |
| 5 | 2016 | 28.65 | 27.96 | 58.75 | 70.58 | 2400000000 | 2600000000 | 2300000 | 14618.90 | 47.42 | 4.00 | 0.38 | 0.40 |
| 5 | 2017 | 30.62 | 29.96 | 59.42 | 71.88 | 2700000000 | 2900000000 | 2300000 | 15130.90 | 52.36 | 4.00 | 0.38 | 15.26 |
| 5 | 2018 | 28.6 | 30.92 | 59.17 | 67.13 | 3000000000 | 3200000000 | 2300000 | 16414.90 | 58.26 | 5.00 | 0.38 | 2.21 |
| 5 | 2019 | 28.12 | 30.92 | 61.00 | 64.33 | 3200000000 | 3400000000 | 3500000 | 15343.10 | 60.51 | 5.00 | 0.38 | 0.65 |
| 6 | 2000 | 41.21 | 43.00 | 68.42 | 71.00 | 68000000 | 140000000 | 292000 | 655.10 | 40.17 | 1.00 | 0.89 | 9.43 |
| 6 | 2001 | 35.17 | 41.50 | 61.75 | 67.08 | 57000000 | 120000000 | 384000 | 703.68 | 41.50 | 1.00 | 0.93 | 15.11 |
| 6 | 2002 | 32.48 | 40.71 | 59.92 | 64.33 | 63000000 | 110000000 | 576000 | 763.08 | 42.77 | 1.00 | 0.97 | 0.03 |
| 6 | 2003 | 31.52 | 37.33 | 60.88 | 64.83 | 70000000 | 120000000 | 768000 | 883.64 | 42.01 | 1.00 | 0.98 | 2.51 |
| 6 | 2004 | 31.83 | 35.92 | 62.29 | 65.46 | 79000000 | 140000000 | 989000 | 1045.01 | 48.79 | 1.00 | 0.98 | 3.69 |
| 6 | 2005 | 28.96 | 34.25 | 60.67 | 63.00 | 100000000 | 190000000 | 693000 | 1578.40 | 62.94 | 1.00 | 0.95 | 0.12 |
| 6 | 2006 | 28.73 | 35.33 | 60.83 | 61.29 | 200000000 | 260000000 | 682000 | 2473.08 | 66.50 | 1.00 | 0.89 | 7.72 |
| 6 | 2007 | 26.75 | 34.75 | 58.79 | 59.96 | 320000000 | 380000000 | 732000 | 3851.44 | 68.13 | 2.00 | 0.86 | 2.75 |
| 6 | 2008 | 26.79 | 36.00 | 54.00 | 63.58 | 380000000 | 460000000 | 1000000 | 5574.60 | 65.78 | 2.00 | 0.82 | 3.76 |
| 6 | 2009 | 25.81 | 35.21 | 52.92 | 63.50 | 550000000 | 490000000 | 1000000 | 4950.29 | 51.64 | 2.00 | 0.80 | 0.71 |
| 6 | 2010 | 23.46 | 35.08 | 51.50 | 60.33 | 790000000 | 860000000 | 1300000 | 5842.81 | 54.30 | 2.00 | 0.80 | 3.36 |
| 6 | 2011 | 26.31 | 36.13 | 51.17 | 65.33 | 1500000000 | 1800000000 | 1600000 | 7189.69 | 56.43 | 2.00 | 0.79 | 9.00 |
| 6 | 2012 | 28.62 | 38.13 | 51.79 | 67.33 | 2600000000 | 2600000000 | 2000000 | 7496.29 | 52.97 | 2.00 | 0.79 | 0.16 |
| 6 | 2013 | 26.94 | 39.46 | 51.71 | 62.71 | 2600000000 | 3000000000 | 2100000 | 7875.76 | 48.42 | 2.00 | 0.78 | 0.72 |
| 6 | 2014 | 24.31 | 39.58 | 51.58 | 57.46 | 2700000000 | 3200000000 | 2200000 | 7891.31 | 43.27 | 2.00 | 0.78 | 10.54 |
| 6 | 2015 | 30.87 | 43.46 | 57.96 | 60.33 | 2500000000 | 2700000000 | 1900000 | 5500.31 | 37.79 | 2.00 | 1.02 | 13.62 |
| 6 | 2016 | 37.96 | 43.83 | 62.67 | 69.42 | 2900000000 | 2600000000 | 2000000 | 3880.74 | 46.43 | 2.00 | 1.60 | 9.14 |
| 6 | 2017 | 35.15 | 45.33 | 57.83 | 67.13 | 3200000000 | 2800000000 | 2500000 | 4147.09 | 48.55 | 2.00 | 1.72 | 27.83 |
| 6 | 2018 | 30.96 | 43.00 | 56.38 | 62.54 | 2800000000 | 2500000000 | 2600000 | 4739.84 | 54.09 | 2.00 | 1.70 | 36.00 |
| 6 | 2019 | 28.29 | 40.50 | 55.46 | 60.63 | 2000000000 | 1800000000 | 3200000 | 4793.59 | 49.18 | 2.00 | 1.68 | 13.94 |
| 7 | 2000 | 29.81 | 35.00 | 61.00 | 63.63 | 4700000000 | 1200000000 | 5100000 | 1450.48 | 16.20 | 5.00 | 3.47 | 47.38 |
| 7 | 2001 | 30.71 | 34.33 | 62.58 | 64.50 | 4100000000 | 1200000000 | 4400000 | 1391.71 | 17.48 | 5.00 | 3.97 | 7.00 |
| 7 | 2002 | 32.58 | 36.50 | 62.54 | 66.13 | 4100000000 | 1300000000 | 4900000 | 1228.94 | 18.32 | 6.00 | 4.50 | 0.18 |
| 7 | 2003 | 33.65 | 36.25 | 64.92 | 66.13 | 4700000000 | 1500000000 | 5700000 | 1138.66 | 21.80 | 6.00 | 5.85 | 3.12 |
| 7 | 2004 | 30.65 | 35.75 | 61.17 | 64.38 | 6300000000 | 1500000000 | 7800000 | 1063.00 | 28.23 | 6.00 | 6.20 | 1.30 |
| 7 | 2005 | 31.1 | 36.96 | 59.83 | 65.42 | 7200000000 | 1900000000 | 8200000 | 1187.52 | 30.34 | 7.00 | 5.78 | 0.08 |
| 7 | 2006 | 31.23 | 37.33 | 59.00 | 66.13 | 8100000000 | 2200000000 | 8600000 | 1398.19 | 29.95 | 7.00 | 5.73 | 0.10 |
| 7 | 2007 | 31.08 | 38.50 | 58.58 | 65.08 | 10000000000 | 2900000000 | 11000000 | 1667.84 | 30.25 | 7.00 | 5.64 | 0.93 |
| 7 | 2008 | 33.46 | 40.58 | 59.21 | 67.13 | 12000000000 | 3400000000 | 12000000 | 2044.53 | 33.04 | 7.00 | 5.43 | 20.87 |
| 7 | 2009 | 33.37 | 41.13 | 57.38 | 68.25 | 12000000000 | 2900000000 | 12000000 | 2329.24 | 24.96 | 7.00 | 5.54 | 23.11 |
| 7 | 2010 | 33.71 | 41.88 | 57.63 | 67.92 | 14000000000 | 2700000000 | 14000000 | 2644.82 | 21.35 | 7.00 | 5.62 | 0.53 |
| 7 | 2011 | 39.56 | 48.71 | 58.25 | 72.17 | 9300000000 | 2600000000 | 9500000 | 2791.96 | 20.57 | 7.00 | 5.93 | 0.93 |
| 7 | 2012 | 39.67 | 49.83 | 57.25 | 72.25 | 11000000000 | 3000000000 | 11000000 | 3232.65 | 16.40 | 7.00 | 6.06 | 26.82 |
| 7 | 2013 | 42.87 | 53.25 | 60.50 | 72.00 | 6700000000 | 3300000000 | 9200000 | 3264.38 | 17.02 | 7.00 | 6.87 | 2.62 |
| 7 | 2014 | 41.69 | 50.96 | 60.50 | 71.92 | 8000000000 | 3500000000 | 9600000 | 3378.83 | 14.24 | 7.00 | 7.08 | 0.25 |
| 7 | 2015 | 40.6 | 49.38 | 61.33 | 70.50 | 6900000000 | 3600000000 | 9100000 | 3598.97 | 13.18 | 7.00 | 7.69 | 0.19 |
| 7 | 2016 | 41.73 | 48.38 | 64.83 | 70.25 | 3300000000 | 4400000000 | 5300000 | 3525.02 | 10.35 | 7.00 | 10.03 | 16.60 |
| 7 | 2017 | 44.94 | 46.04 | 70.83 | 73.00 | 8600000000 | 2400000000 | 8200000 | 2440.51 | 15.82 | 7.00 | 17.78 | 1.78 |
| 7 | 2018 | 38.06 | 42.50 | 62.88 | 70.75 | 13000000000 | 2900000000 | 11000000 | 2549.13 | 18.91 | 7.00 | 17.77 | 0.33 |
| 7 | 2019 | 35.17 | 41.04 | 60.04 | 69.25 | 14000000000 | 3700000000 | 13000000 | 3019.21 | 17.50 | 7.00 | 17.75 | 0.65 |
| 8 | 2000 | 40.77 | 38.67 | 73.88 | 69.00 | 210000000 | 80000000 | 136000 | 124.46 | 48.80 | 7.00 | 8.22 | 9.78 |
| 8 | 2001 | 39.58 | 42.54 | 69.50 | 67.13 | 220000000 | 50000000 | 148000 | 120.77 | 45.88 | 7.00 | 8.46 | 26.27 |
| 8 | 2002 | 40.69 | 45.63 | 68.25 | 67.50 | 260000000 | 55000000 | 156000 | 111.93 | 42.96 | 7.00 | 8.57 | 5.89 |
| 8 | 2003 | 41.37 | 47.25 | 68.50 | 67.00 | 340000000 | 63000000 | 180000 | 119.49 | 40.04 | 7.00 | 8.60 | 0.31 |
| 8 | 2004 | 41.21 | 46.92 | 68.50 | 67.00 | 460000000 | 59000000 | 184000 | 136.47 | 37.12 | 7.00 | 8.64 | 0.84 |
| 8 | 2005 | 43.08 | 48.17 | 68.50 | 69.50 | 530000000 | 61000000 | 227000 | 162.43 | 34.20 | 7.00 | 8.67 | 0.04 |
| 8 | 2006 | 42.48 | 48.96 | 66.00 | 70.00 | 640000000 | 63000000 | 330000 | 194.69 | 31.28 | 8.00 | 8.70 | 23.14 |
| 8 | 2007 | 40.27 | 49.13 | 60.96 | 70.46 | 790000000 | 65000000 | 358000 | 244.29 | 28.37 | 8.00 | 8.97 | 31.53 |
| 8 | 2008 | 40.21 | 49.50 | 60.75 | 70.17 | 1200000000 | 67000000 | 383000 | 326.44 | 25.45 | 8.00 | 9.60 | 0.14 |
| 8 | 2009 | 41.58 | 49.21 | 63.96 | 70.00 | 1100000000 | 140000000 | 427000 | 380.57 | 22.53 | 8.00 | 11.78 | 39.19 |
| 8 | 2010 | 42.65 | 50.67 | 64.96 | 69.67 | 1400000000 | 100000000 | 468000 | 341.55 | 19.61 | 8.00 | 14.41 | 52.69 |
| 8 | 2011 | 40.83 | 51.08 | 65.33 | 65.25 | 2000000000 | 120000000 | 523000 | 354.48 | 16.69 | 9.00 | 16.90 | 0.90 |
| 8 | 2012 | 40.75 | 52.83 | 60.96 | 67.71 | 2000000000 | 140000000 | 597000 | 467.08 | 13.77 | 9.00 | 17.70 | 11.80 |
| 8 | 2013 | 40.9 | 53.13 | 60.67 | 68.00 | 2200000000 | 160000000 | 681000 | 499.53 | 12.48 | 9.00 | 18.63 | 1.32 |
| 8 | 2014 | 40.85 | 52.58 | 61.13 | 68.00 | 2100000000 | 180000000 | 770000 | 566.93 | 11.64 | 9.00 | 19.59 | 1.10 |
| 8 | 2015 | 40.17 | 50.50 | 62.67 | 67.17 | 2300000000 | 190000000 | 864000 | 640.54 | 9.36 | 9.00 | 20.58 | 15.04 |
| 8 | 2016 | 41.46 | 48.71 | 65.38 | 68.83 | 2100000000 | 210000000 | 871000 | 717.13 | 7.81 | 9.00 | 21.73 | 7.27 |
| 8 | 2017 | 42.31 | 50.33 | 66.13 | 68.17 | 2500000000 | 360000000 | 933000 | 768.52 | 7.63 | 9.00 | 23.87 | 4.53 |
| 8 | 2018 | 42.27 | 48.96 | 68.83 | 66.75 | 3500000000 | 610000000 | 849000 | 771.52 | 8.37 | 9.00 | 27.43 | 4.30 |
| 8 | 2019 | 38.96 | 44.13 | 65.79 | 68.00 | 4600000000 | 860000000 | 812000 | 855.76 | 7.94 | 9.00 | 30.99 | 7.61 |
| 9 | 2000 | 14.94 | 13.83 | 60.79 | 55.25 | 3500000000 | 2600000000 | 6600000 | 26241.40 | 94.49 | 2.00 | 0.66 | 31.29 |
| 9 | 2001 | 11.65 | 10.38 | 58.25 | 54.67 | 3800000000 | 3000000000 | 6400000 | 28227.70 | 95.33 | 2.00 | 0.69 | 49.39 |
| 9 | 2002 | 11.31 | 7.71 | 58.29 | 56.63 | 4200000000 | 3800000000 | 6500000 | 32541.10 | 90.48 | 2.00 | 0.67 | 9.90 |
| 9 | 2003 | 12.79 | 7.83 | 59.00 | 58.75 | 5200000000 | 4800000000 | 6800000 | 41106.90 | 80.85 | 2.00 | 0.61 | 1.20 |
| 9 | 2004 | 13.67 | 10.42 | 58.71 | 58.21 | 6100000000 | 5300000000 | 7000000 | 47631.00 | 80.54 | 2.00 | 0.55 | 3.43 |
| 9 | 2005 | 14.6 | 10.33 | 60.96 | 57.92 | 6800000000 | 6200000000 | 7300000 | 50878.20 | 79.58 | 2.00 | 0.55 | 0.16 |
| 9 | 2006 | 15.12 | 10.17 | 62.29 | 57.79 | 7700000000 | 7000000000 | 8000000 | 54306.40 | 79.00 | 2.00 | 0.54 | 42.96 |
| 9 | 2007 | 15.17 | 10.25 | 61.58 | 58.50 | 9300000000 | 8800000000 | 8300000 | 61359.70 | 80.78 | 2.00 | 0.50 | 31.65 |
| 9 | 2008 | 16.65 | 10.75 | 61.96 | 60.58 | 10000000000 | 11000000000 | 8000000 | 61262.10 | 84.12 | 2.00 | 0.54 | 0.90 |
| 9 | 2009 | 24.81 | 17.13 | 62.79 | 69.71 | 8500000000 | 7900000000 | 7200000 | 52105.20 | 93.24 | 2.00 | 0.64 | 4.43 |
| 9 | 2010 | 27.81 | 20.13 | 64.17 | 71.33 | 8200000000 | 7200000000 | 7100000 | 48715.20 | 103.02 | 2.00 | 0.65 | 2.94 |
| 9 | 2011 | 26.37 | 19.79 | 65.21 | 67.75 | 9500000000 | 6800000000 | 7600000 | 51848.90 | 103.79 | 2.00 | 0.62 | 2.67 |
| 9 | 2012 | 27.79 | 21.71 | 68.38 | 65.50 | 9300000000 | 6000000000 | 7600000 | 48917.90 | 104.51 | 2.00 | 0.63 | 26.33 |
| 9 | 2013 | 26.85 | 22.96 | 68.38 | 62.38 | 10000000000 | 6300000000 | 8300000 | 51590.20 | 103.66 | 2.00 | 0.64 | 2.52 |
| 9 | 2014 | 23.58 | 19.00 | 68.71 | 59.46 | 11000000000 | 6500000000 | 8800000 | 55493.00 | 110.03 | 2.00 | 0.61 | 43.11 |
| 9 | 2015 | 20.1 | 14.17 | 69.79 | 56.25 | 11000000000 | 5800000000 | 9500000 | 61995.40 | 121.97 | 2.00 | 0.65 | 1.34 |
| 9 | 2016 | 17.83 | 14.21 | 65.96 | 55.50 | 13000000000 | 6300000000 | 10000000 | 63197.10 | 120.82 | 2.00 | 0.74 | 0.03 |
| 9 | 2017 | 17.6 | 14.25 | 65.46 | 55.50 | 14000000000 | 6600000000 | 10000000 | 69822.30 | 121.04 | 2.00 | 0.78 | 0.01 |
| 9 | 2018 | 16.44 | 13.79 | 64.67 | 54.42 | 15000000000 | 7500000000 | 11000000 | 78621.20 | 122.33 | 2.00 | 0.75 | 2.85 |
| 9 | 2019 | 16.25 | 13.67 | 64.08 | 54.75 | 15000000000 | 8200000000 | 11000000 | 78661.00 | 126.80 | 2.00 | 0.72 | 9.56 |
| 10 | 2000 | 26.02 | 24.75 | 64.71 | 51.25 | 660000000 | 250000000 | 1200000 | 4075.97 | 61.55 | 1.00 | 16.97 | 1.08 |
| 10 | 2001 | 23.98 | 23.50 | 62.88 | 52.00 | 660000000 | 250000000 | 1300000 | 4509.18 | 61.21 | 1.00 | 17.48 | 13.21 |
| 10 | 2002 | 25.21 | 25.79 | 62.63 | 52.75 | 740000000 | 310000000 | 1400000 | 5344.88 | 57.92 | 1.00 | 16.61 | 1.01 |
| 10 | 2003 | 25.37 | 25.50 | 63.25 | 53.50 | 880000000 | 400000000 | 1500000 | 7206.17 | 57.14 | 1.00 | 13.86 | 0.34 |
| 10 | 2004 | 24.85 | 25.13 | 62.75 | 54.25 | 1100000000 | 490000000 | 1800000 | 8912.99 | 61.09 | 1.00 | 12.60 | 56.60 |
| 10 | 2005 | 24.42 | 25.13 | 62.71 | 55.00 | 1200000000 | 530000000 | 1900000 | 10406.40 | 65.47 | 2.00 | 12.58 | 49.37 |
| 10 | 2006 | 23.67 | 23.54 | 62.79 | 55.75 | 1400000000 | 710000000 | 1900000 | 12631.60 | 63.31 | 2.00 | 12.47 | 20.10 |
| 10 | 2007 | 25.92 | 22.83 | 67.17 | 56.50 | 1400000000 | 800000000 | 1900000 | 16741.90 | 62.61 | 2.00 | 11.43 | 1.42 |
| 10 | 2008 | 28.1 | 23.38 | 69.33 | 57.25 | 1600000000 | 940000000 | 2200000 | 18227.10 | 66.31 | 2.00 | 10.69 | 0.18 |
| 10 | 2009 | 32 | 26.21 | 70.29 | 58.00 | 1700000000 | 1100000000 | 2200000 | 14795.00 | 60.52 | 2.00 | 11.26 | 0.30 |
| 10 | 2010 | 34.77 | 28.08 | 72.58 | 58.75 | 1800000000 | 1200000000 | 2500000 | 14790.80 | 74.45 | 2.00 | 11.81 | 60.20 |
| 10 | 2011 | 27.92 | 26.63 | 69.71 | 59.50 | 1900000000 | 1300000000 | 2800000 | 17621.50 | 85.83 | 2.00 | 0.62 | 0.20 |
| 10 | 2012 | 30.81 | 28.75 | 72.63 | 60.25 | 2100000000 | 1500000000 | 3000000 | 17534.40 | 85.57 | 2.00 | 0.63 | 1.35 |
| 10 | 2013 | 30.62 | 28.25 | 72.71 | 60.29 | 2000000000 | 1200000000 | 3100000 | 19174.10 | 84.02 | 2.00 | 0.64 | 14.70 |
| 10 | 2014 | 30.6 | 27.25 | 73.63 | 60.33 | 2300000000 | 1300000000 | 3200000 | 20367.10 | 81.33 | 2.00 | 0.61 | 0.80 |
| 10 | 2015 | 30.81 | 24.83 | 76.21 | 60.58 | 1900000000 | 1200000000 | 3000000 | 17522.20 | 76.87 | 2.00 | 0.65 | 0.28 |
| 10 | 2016 | 25.77 | 25.00 | 65.04 | 61.50 | 1900000000 | 1300000000 | 3100000 | 18237.30 | 77.45 | 2.00 | 0.74 | 6.17 |
| 10 | 2017 | 25.85 | 24.50 | 67.17 | 60.04 | 2100000000 | 1400000000 | 3200000 | 20388.20 | 76.14 | 2.00 | 0.78 | 0.40 |
| 10 | 2018 | 24.54 | 25.25 | 65.04 | 58.79 | 2300000000 | 1700000000 | 3200000 | 23258.50 | 74.26 | 2.00 | 0.75 | 8.55 |
| 10 | 2019 | 24.65 | 26.25 | 64.33 | 58.71 | 2300000000 | 1800000000 | 6100000 | 23723.30 | 72.91 | 2.00 | 0.72 | 11.56 |
| 11 | 2000 | 53.46 | 54.75 | 75.88 | 76.29 | 34000000 | 150000000 | 51000 | 556.84 | 89.69 | 0.00 | 10.04 | 0.62 |
| 11 | 2001 | 48.68 | 49.46 | 75.46 | 72.46 | 35000000 | 80000000 | 67000 | 527.33 | 75.39 | 0.00 | 22.06 | 7.22 |
| 11 | 2002 | 47.85 | 46.08 | 77.63 | 72.00 | 51000000 | 52000000 | 91000 | 872.50 | 57.09 | 0.00 | 43.53 | 6.78 |
| 11 | 2003 | 45.12 | 42.96 | 74.79 | 72.50 | 63000000 | 49000000 | 107000 | 982.96 | 54.32 | 0.00 | 74.61 | 7.18 |
| 11 | 2004 | 42.27 | 42.54 | 67.58 | 74.42 | 82000000 | 86000000 | 194000 | 1255.56 | 58.38 | 0.00 | 83.54 | 0.37 |
| 11 | 2005 | 38.54 | 43.17 | 64.42 | 69.50 | 100000000 | 140000000 | 210000 | 1902.42 | 65.53 | 0.00 | 87.16 | 36.16 |
| 11 | 2006 | 31.54 | 43.08 | 58.88 | 61.13 | 91000000 | 390000000 | 121000 | 2599.57 | 63.47 | 0.00 | 80.37 | 4.54 |
| 11 | 2007 | 29.98 | 42.54 | 58.04 | 59.38 | 240000000 | 470000000 | 195000 | 3122.00 | 67.91 | 0.00 | 76.71 | 0.22 |
| 11 | 2008 | 28.35 | 42.33 | 56.38 | 58.00 | 290000000 | 450000000 | 294000 | 4080.94 | 72.48 | 0.00 | 75.03 | 17.88 |
| 11 | 2009 | 29 | 41.00 | 55.50 | 61.50 | 550000000 | 270000000 | 366000 | 3122.78 | 58.76 | 0.00 | 79.33 | 0.16 |
| 11 | 2010 | 30.52 | 41.25 | 56.92 | 62.88 | 730000000 | 280000000 | 425000 | 3587.88 | 61.54 | 0.00 | 91.91 | 26.70 |
| 11 | 2011 | 30.37 | 43.25 | 57.00 | 60.50 | 650000000 | 320000000 | 481000 | 4615.47 | 60.67 | 0.00 | 93.93 | 0.19 |
| 11 | 2012 | 28.71 | 44.54 | 54.63 | 58.25 | 710000000 | 290000000 | 528000 | 5100.10 | 55.94 | 0.00 | 95.47 | 49.08 |
| 11 | 2013 | 28.19 | 44.21 | 54.33 | 57.83 | 1200000000 | 320000000 | 650000 | 5254.88 | 50.75 | 0.00 | 96.52 | 1.65 |
| 11 | 2014 | 30.9 | 45.88 | 55.42 | 60.50 | 1600000000 | 510000000 | 595000 | 5408.41 | 44.70 | 0.00 | 98.30 | 0.13 |
| 11 | 2015 | 37.25 | 47.50 | 60.25 | 66.75 | 1200000000 | 390000000 | 592000 | 4166.98 | 29.75 | 0.00 | 120.06 | 7.58 |
| 11 | 2016 | 43.6 | 48.29 | 65.33 | 73.58 | 630000000 | 820000000 | 397000 | 3506.07 | 28.12 | 0.00 | 163.66 | 0.00 |
| 11 | 2017 | 41.9 | 48.54 | 61.33 | 73.92 | 880000000 | 1200000000 | 261000 | 4095.81 | 29.00 | 1.00 | 165.92 | 1.49 |
| 11 | 2018 | 43.75 | 46.71 | 68.46 | 72.33 | 560000000 | 760000000 | 218000 | 3289.65 | 40.84 | 1.00 | 252.86 | 1.37 |
| 11 | 2019 | 40.98 | 44.38 | 67.46 | 70.13 | 400000000 | 720000000 | 218000 | 2790.73 | 40.56 | 1.00 | 339.80 | 0.16 |
| 12 | 2000 | 17.81 | 17.42 | 58.71 | 59.50 | 11000000000 | 7000000000 | 18000000 | 24564.50 | 43.35 | 6.00 | 0.66 | 7.04 |
| 12 | 2001 | 14.1 | 11.29 | 57.21 | 59.71 | 12000000000 | 7400000000 | 18000000 | 24537.50 | 44.62 | 8.00 | 0.69 | 2.42 |
| 12 | 2002 | 13.92 | 10.54 | 56.83 | 60.46 | 12000000000 | 7700000000 | 19000000 | 26401.70 | 45.32 | 8.00 | 0.67 | 6.35 |
| 12 | 2003 | 13.85 | 9.63 | 57.92 | 60.17 | 15000000000 | 9800000000 | 19000000 | 32222.90 | 44.57 | 8.00 | 0.61 | 1.45 |
| 12 | 2004 | 14.04 | 11.42 | 57.38 | 59.29 | 17000000000 | 11000000000 | 19000000 | 36821.50 | 46.87 | 8.00 | 0.55 | 0.11 |
| 12 | 2005 | 15.4 | 14.08 | 57.71 | 59.00 | 18000000000 | 11000000000 | 20000000 | 38403.10 | 48.62 | 8.00 | 0.55 | 0.09 |
| 12 | 2006 | 16 | 14.63 | 58.71 | 58.67 | 16000000000 | 11000000000 | 20000000 | 40635.30 | 50.84 | 8.00 | 0.54 | 64.00 |
| 12 | 2007 | 16.92 | 13.25 | 63.92 | 56.67 | 16000000000 | 12000000000 | 21000000 | 46855.80 | 52.56 | 9.00 | 0.50 | 18.09 |
| 12 | 2008 | 16.48 | 12.67 | 63.46 | 56.83 | 17000000000 | 12000000000 | 22000000 | 51708.80 | 53.25 | 9.00 | 0.54 | 38.53 |
| 12 | 2009 | 17.62 | 10.46 | 62.54 | 62.25 | 17000000000 | 12000000000 | 21000000 | 47963.20 | 45.21 | 9.00 | 0.64 | 51.77 |
| 12 | 2010 | 18.1 | 11.13 | 62.08 | 63.00 | 18000000000 | 12000000000 | 22000000 | 46858.00 | 51.26 | 10.00 | 0.65 | 55.10 |
| 12 | 2011 | 17.52 | 12.50 | 61.63 | 60.92 | 18000000000 | 13000000000 | 23000000 | 51375.00 | 53.95 | 12.00 | 0.62 | 1.50 |
| 12 | 2012 | 21.73 | 16.71 | 63.63 | 63.13 | 19000000000 | 13000000000 | 24000000 | 48567.70 | 53.97 | 12.00 | 0.63 | 15.10 |
| 12 | 2013 | 21.56 | 17.96 | 62.83 | 62.33 | 19000000000 | 13000000000 | 25000000 | 50716.70 | 53.44 | 12.00 | 0.64 | 0.16 |
| 12 | 2014 | 19.65 | 17.04 | 62.71 | 59.54 | 20000000000 | 13000000000 | 25000000 | 51717.50 | 53.39 | 12.00 | 0.61 | 0.98 |
| 12 | 2015 | 21.79 | 17.25 | 65.46 | 60.88 | 20000000000 | 11000000000 | 27000000 | 44178.00 | 53.09 | 12.00 | 0.65 | 18.82 |
| 12 | 2016 | 20.81 | 19.67 | 63.00 | 58.96 | 21000000000 | 12000000000 | 28000000 | 45237.80 | 52.45 | 12.00 | 0.74 | 4.68 |
| 12 | 2017 | 19.81 | 18.04 | 63.08 | 58.50 | 22000000000 | 13000000000 | 29000000 | 47549.10 | 54.04 | 13.00 | 0.78 | 0.79 |
| 12 | 2018 | 18.96 | 17.75 | 62.79 | 57.38 | 25000000000 | 14000000000 | 31000000 | 51525.00 | 55.76 | 13.00 | 0.75 | 12.53 |
| 12 | 2019 | 19.77 | 18.58 | 62.75 | 58.21 | 25000000000 | 14000000000 | 32000000 | 50137.70 | 55.58 | 13.00 | 0.72 | 30.18 |
| 13 | 2000 | 17.69 | 11.17 | 65.96 | 58.25 | 12000000000 | 9100000000 | 4900000 | 21679.20 | 19.43 | 19.00 | 1.72 | 0.14 |
| 13 | 2001 | 19.39 | 12.67 | 65.79 | 60.33 | 11000000000 | 8200000000 | 4900000 | 19490.90 | 22.23 | 19.00 | 1.93 | 3.06 |
| 13 | 2002 | 17.46 | 11.96 | 63.83 | 59.13 | 12000000000 | 8600000000 | 4800000 | 20082.50 | 20.77 | 19.00 | 1.84 | 5.56 |
| 13 | 2003 | 18.62 | 14.08 | 64.17 | 59.00 | 15000000000 | 10000000000 | 4700000 | 23447.00 | 19.10 | 20.00 | 1.54 | 0.17 |
| 13 | 2004 | 17.75 | 12.33 | 64.67 | 58.50 | 18000000000 | 15000000000 | 5200000 | 30430.70 | 17.20 | 21.00 | 1.36 | 15.38 |
| 13 | 2005 | 17.62 | 11.67 | 63.83 | 59.75 | 20000000000 | 16000000000 | 5500000 | 33999.20 | 18.27 | 21.00 | 1.31 | 4.30 |
| 13 | 2006 | 17.75 | 13.04 | 64.08 | 58.38 | 20000000000 | 17000000000 | 5500000 | 36044.90 | 19.89 | 21.00 | 1.33 | 5.19 |
| 13 | 2007 | 18.19 | 13.50 | 64.46 | 58.42 | 25000000000 | 22000000000 | 5600000 | 40960.10 | 20.20 | 22.00 | 1.20 | 1.74 |
| 13 | 2008 | 19.52 | 12.83 | 66.63 | 59.58 | 27000000000 | 26000000000 | 5600000 | 49601.70 | 20.16 | 22.00 | 1.19 | 4.64 |
| 13 | 2009 | 22.19 | 12.21 | 69.04 | 63.13 | 27000000000 | 23000000000 | 5500000 | 42772.40 | 23.00 | 22.00 | 1.28 | 12.92 |
| 13 | 2010 | 21.48 | 13.92 | 67.04 | 62.00 | 31000000000 | 28000000000 | 5800000 | 52022.10 | 19.81 | 23.00 | 1.09 | 0.24 |
| 13 | 2011 | 23.15 | 17.88 | 66.42 | 62.00 | 34000000000 | 34000000000 | 5800000 | 62517.80 | 21.44 | 24.00 | 0.97 | 1.24 |
| 13 | 2012 | 22.17 | 18.08 | 65.88 | 60.38 | 34000000000 | 35000000000 | 6000000 | 68012.10 | 21.48 | 24.00 | 0.97 | 11.12 |
| 13 | 2013 | 21.6 | 16.46 | 66.58 | 60.17 | 33000000000 | 35000000000 | 6500000 | 68150.10 | 19.96 | 24.00 | 1.04 | 18.15 |
| 13 | 2014 | 21.54 | 16.29 | 66.25 | 60.54 | 36000000000 | 36000000000 | 6900000 | 62510.80 | 21.04 | 24.00 | 1.11 | 10.94 |
| 13 | 2015 | 23.27 | 16.38 | 69.33 | 60.83 | 36000000000 | 34000000000 | 7400000 | 56755.70 | 19.98 | 24.00 | 1.33 | 38.67 |
| 13 | 2016 | 21.58 | 15.46 | 67.21 | 60.50 | 39000000000 | 36000000000 | 8300000 | 49971.10 | 19.25 | 24.00 | 1.35 | 41.67 |
| 13 | 2017 | 21.87 | 16.58 | 66.67 | 60.50 | 44000000000 | 40000000000 | 8800000 | 54066.50 | 21.19 | 24.00 | 1.30 | 18.24 |
| 13 | 2018 | 21.81 | 16.75 | 66.58 | 60.29 | 47000000000 | 42000000000 | 9200000 | 57395.90 | 21.80 | 24.00 | 1.34 | 53.24 |
| 13 | 2019 | 20.52 | 15.58 | 66.21 | 59.25 | 48000000000 | 41000000000 | 9500000 | 55060.30 | 24.11 | 24.00 | 1.37 | 14.53 |
| 14 | 2000 | 35.21 | 38.17 | 64.92 | 67.33 | 5700000 | 46000000 | 58000 | 602.19 | 66.18 | 0.00 | 2.78 | 0.35 |
| 14 | 2001 | 35.54 | 42.21 | 64.38 | 64.50 | 5500000 | 49000000 | 54000 | 515.69 | 65.33 | 0.00 | 3.39 | 3.86 |
| 14 | 2002 | 37.73 | 43.46 | 63.17 | 68.83 | 6000000 | 60000000 | 54000 | 491.83 | 60.92 | 0.00 | 3.90 | 1.63 |
| 14 | 2003 | 39.15 | 45.58 | 63.58 | 69.13 | 4900000 | 52000000 | 56000 | 568.25 | 69.42 | 0.00 | 3.56 | 0.09 |
| 14 | 2004 | 38.9 | 45.46 | 65.04 | 67.29 | 7100000 | 72000000 | 59000 | 618.04 | 72.16 | 0.00 | 3.22 | 0.10 |
| 14 | 2005 | 33.08 | 42.67 | 60.67 | 62.83 | 9400000 | 56000000 | 69000 | 749.19 | 74.91 | 0.00 | 3.10 | 1.48 |
| 14 | 2006 | 30.4 | 40.96 | 59.00 | 60.83 | 3900000 | 43000000 | 78000 | 1256.98 | 77.66 | 0.00 | 3.06 | 17.38 |
| 14 | 2007 | 29.19 | 39.75 | 58.38 | 60.25 | 4500000 | 81000000 | 104000 | 1401.95 | 80.40 | 0.00 | 2.97 | 27.22 |
| 14 | 2008 | 28.1 | 37.63 | 58.33 | 60.25 | 3700000 | 75000000 | 114000 | 1672.95 | 83.15 | 1.00 | 2.70 | 0.66 |
| 14 | 2009 | 30.29 | 39.00 | 57.17 | 64.42 | 3100000 | 130000000 | 121000 | 1626.31 | 85.89 | 1.00 | 2.76 | 2.02 |
| 14 | 2010 | 32.81 | 40.54 | 58.92 | 66.17 | 2400000 | 140000000 | 140000 | 1949.35 | 88.64 | 1.00 | 2.72 | 33.48 |
| 14 | 2011 | 35.79 | 41.42 | 61.17 | 69.00 | 5100000 | 190000000 | 158000 | 2406.91 | 91.39 | 1.00 | 2.37 | 4.71 |
| 14 | 2012 | 36.1 | 40.92 | 60.33 | 70.96 | 4400000 | 200000000 | 168000 | 2790.68 | 94.13 | 1.00 | 2.08 | 1.27 |
| 14 | 2013 | 37.85 | 39.38 | 62.58 | 73.75 | 3700000 | 210000000 | 174000 | 2729.89 | 96.88 | 1.00 | 2.24 | 0.23 |
| 14 | 2014 | 35.6 | 41.00 | 63.38 | 66.83 | 2800000 | 170000000 | 182000 | 2920.78 | 99.62 | 1.00 | 2.46 | 19.10 |
| 14 | 2015 | 33.52 | 43.17 | 60.92 | 62.96 | 2200000 | 140000000 | 183000 | 2679.35 | 102.37 | 1.00 | 2.77 | 2.64 |
| 14 | 2016 | 36.1 | 42.75 | 64.38 | 65.08 | 1600000 | 130000000 | 179000 | 2509.63 | 105.12 | 1.00 | 3.13 | 0.39 |
| 14 | 2017 | 34.06 | 43.79 | 59.96 | 64.38 | 15000000 | 170000000 | 139000 | 2695.25 | 107.86 | 1.00 | 3.19 | 1.59 |
| 14 | 2018 | 32.25 | 44.71 | 57.92 | 61.88 | 4200000 | 130000000 | 140000 | 2720.39 | 110.61 | 1.00 | 3.29 | 10.88 |
| 14 | 2019 | 38.67 | 43.96 | 63.42 | 69.96 | 9700000 | 99000000 | 211000 | 2829.17 | 113.36 | 1.00 | 3.40 | 28.32 |
| 15 | 2000 | 26.4 | 18.00 | 69.00 | 65.79 | 1800000000 | 350000000 | 1500000 | 27097.60 | 38.05 | 0.00 | 1.00 | 6.87 |
| 15 | 2001 | 24.68 | 16.21 | 69.00 | 64.17 | 1700000000 | 340000000 | 1500000 | 27486.20 | 34.07 | 0.00 | 1.00 | 5.00 |
| 15 | 2002 | 24 | 15.50 | 69.00 | 63.50 | 1800000000 | 340000000 | 1500000 | 28866.80 | 36.18 | 0.00 | 1.00 | 2.72 |
| 15 | 2003 | 22.98 | 15.96 | 66.29 | 63.71 | 1800000000 | 400000000 | 1500000 | 28327.30 | 35.81 | 0.00 | 1.00 | 0.07 |
| 15 | 2004 | 19.5 | 14.54 | 62.21 | 62.25 | 1900000000 | 470000000 | 1600000 | 28395.90 | 38.22 | 0.00 | 1.00 | 25.85 |
| 15 | 2005 | 18.73 | 13.58 | 62.13 | 61.75 | 2100000000 | 530000000 | 1600000 | 30278.40 | 38.76 | 0.00 | 1.00 | 41.52 |
| 15 | 2006 | 19.75 | 14.50 | 62.50 | 62.50 | 2100000000 | 540000000 | 1600000 | 30713.70 | 38.31 | 0.00 | 1.00 | 0.27 |
| 15 | 2007 | 19.44 | 15.71 | 60.21 | 62.96 | 2200000000 | 540000000 | 1500000 | 31472.10 | 40.09 | 0.00 | 1.00 | 36.56 |
| 15 | 2008 | 20.25 | 16.00 | 63.00 | 61.50 | 2200000000 | 460000000 | 1500000 | 30627.20 | 39.49 | 0.00 | 1.00 | 54.00 |
| 15 | 2009 | 21.25 | 16.00 | 61.33 | 65.17 | 2000000000 | 390000000 | 1300000 | 28552.20 | 34.19 | 0.00 | 1.00 | 1.32 |
| 15 | 2010 | 24.04 | 17.83 | 63.25 | 67.00 | 2200000000 | 370000000 | 1400000 | 28443.40 | 34.95 | 0.00 | 1.00 | 18.00 |
| 15 | 2011 | 24.52 | 18.54 | 63.50 | 67.00 | 2200000000 | 350000000 | 1300000 | 28006.40 | 37.44 | 1.00 | 1.00 | 2.58 |
| 15 | 2012 | 24.96 | 20.29 | 61.63 | 68.00 | 2300000000 | 380000000 | 1400000 | 29485.60 | 38.13 | 1.00 | 1.00 | 1.79 |
| 15 | 2013 | 24.79 | 20.08 | 61.00 | 68.50 | 2300000000 | 360000000 | 1400000 | 28768.00 | 40.20 | 1.00 | 1.00 | 18.05 |
| 15 | 2014 | 24.52 | 19.96 | 62.25 | 66.83 | 2300000000 | 330000000 | 1400000 | 29445.00 | 38.02 | 1.00 | 1.00 | 8.52 |
| 15 | 2015 | 24.06 | 19.42 | 62.79 | 65.92 | 2600000000 | 350000000 | 1500000 | 31406.00 | 35.10 | 1.00 | 1.00 | 9.15 |
| 15 | 2016 | 23.94 | 19.67 | 62.71 | 65.50 | 3100000000 | 440000000 | 1500000 | 31588.80 | 35.27 | 1.00 | 1.00 | 8.95 |
| 15 | 2017 | 24.08 | 20.17 | 62.83 | 65.17 | 3000000000 | 550000000 | 1400000 | 31827.20 | 34.50 | 1.00 | 1.00 | 9.08 |
| 15 | 2018 | 24.08 | 20.75 | 62.13 | 65.29 | 3400000000 | 540000000 | 1600000 | 32217.90 | 36.08 | 1.00 | 1.00 | 46.33 |
| 15 | 2019 | 24.85 | 22.13 | 62.08 | 65.50 | 3600000000 | 510000000 | 7300000 | 34863.70 | 39.24 | 1.00 | 1.00 | 79.12 |
| 16 | 2000 | 46.1 | 55.58 | 68.83 | 67.79 | 550000000 | 570000000 | 557000 | 576.20 | 12.12 | 6.00 | 53.65 | 21.15 |
| 16 | 2001 | 44.2 | 51.50 | 69.58 | 67.33 | 530000000 | 560000000 | 500000 | 544.49 | 13.34 | 6.00 | 61.93 | 2.65 |
| 16 | 2002 | 42.58 | 51.29 | 66.88 | 67.00 | 560000000 | 490000000 | 498000 | 534.30 | 13.78 | 6.00 | 59.72 | 3.39 |
| 16 | 2003 | 38.48 | 51.21 | 62.25 | 63.50 | 620000000 | 1200000000 | 501000 | 599.38 | 15.17 | 6.00 | 57.75 | 0.20 |
| 16 | 2004 | 36.29 | 51.29 | 57.96 | 63.33 | 770000000 | 1600000000 | 648000 | 687.84 | 14.24 | 6.00 | 58.26 | 64.25 |
| 16 | 2005 | 40.98 | 55.29 | 61.17 | 65.50 | 830000000 | 1800000000 | 798000 | 748.92 | 14.31 | 6.00 | 59.51 | 48.82 |
| 16 | 2006 | 39.92 | 52.58 | 61.42 | 65.83 | 920000000 | 2000000000 | 898000 | 836.86 | 14.13 | 6.00 | 60.27 | 1.00 |
| 16 | 2007 | 41.12 | 54.33 | 61.42 | 66.50 | 910000000 | 2100000000 | 840000 | 908.10 | 13.21 | 6.00 | 60.74 | 6.82 |
| 16 | 2008 | 44.62 | 55.71 | 64.96 | 68.58 | 990000000 | 2200000000 | 823000 | 990.85 | 12.38 | 6.00 | 70.41 | 4.13 |
| 16 | 2009 | 44.69 | 54.29 | 63.79 | 71.29 | 950000000 | 1100000000 | 855000 | 958.00 | 12.40 | 6.00 | 81.71 | 4.26 |
| 16 | 2010 | 42.04 | 53.75 | 61.00 | 69.33 | 1000000000 | 1400000000 | 907000 | 987.41 | 13.52 | 6.00 | 85.19 | 30.18 |
| 16 | 2011 | 43.06 | 54.83 | 61.08 | 70.21 | 1100000000 | 1900000000 | 1200000 | 1164.98 | 13.97 | 6.00 | 86.34 | 4.33 |
| 16 | 2012 | 43.17 | 55.04 | 62.67 | 68.63 | 1000000000 | 1900000000 | 966000 | 1198.11 | 12.40 | 6.00 | 93.40 | 62.43 |
| 16 | 2013 | 41.6 | 51.21 | 63.63 | 68.38 | 940000000 | 1600000000 | 771000 | 1208.90 | 13.28 | 6.00 | 101.63 | 1.80 |
| 16 | 2014 | 40.42 | 49.92 | 62.04 | 68.88 | 970000000 | 2200000000 | 676000 | 1251.16 | 12.24 | 6.00 | 101.10 | 0.16 |
| 16 | 2015 | 39.87 | 51.42 | 62.00 | 66.33 | 920000000 | 2600000000 | 581000 | 1356.67 | 10.60 | 6.00 | 102.77 | 0.09 |
| 16 | 2016 | 37.6 | 49.46 | 60.38 | 65.38 | 790000000 | 2900000000 | 486000 | 1368.45 | 9.15 | 6.00 | 104.77 | 4.60 |
| 16 | 2017 | 37.35 | 48.63 | 60.13 | 65.96 | 870000000 | 3300000000 | 391000 | 1464.99 | 8.26 | 6.00 | 105.46 | 19.89 |
| 16 | 2018 | 40.29 | 49.04 | 64.67 | 66.88 | 850000000 | 3000000000 | 296000 | 1482.31 | 8.97 | 6.00 | 121.82 | 3.77 |
| 16 | 2019 | 43.37 | 50.50 | 67.33 | 68.92 | 950000000 | 3000000000 | 201000 | 1284.70 | 10.12 | 6.00 | 138.19 | 9.91 |
| 17 | 2000 | 34.81 | 42.25 | 60.00 | 67.38 | 88000000 | 150000000 | 289000 | 1663.61 | 43.33 | 1.00 | 3486.35 | 1.67 |
| 17 | 2001 | 34.22 | 37.71 | 62.08 | 68.67 | 91000000 | 130000000 | 279000 | 1565.05 | 40.64 | 1.00 | 4105.92 | 0.89 |
| 17 | 2002 | 38.87 | 43.08 | 64.42 | 70.25 | 76000000 | 120000000 | 250000 | 1300.85 | 47.48 | 1.00 | 5716.26 | 59.40 |
| 17 | 2003 | 39.62 | 44.13 | 64.04 | 71.08 | 81000000 | 120000000 | 268000 | 1365.42 | 47.14 | 1.00 | 6424.34 | 61.29 |
| 17 | 2004 | 34.71 | 42.54 | 60.92 | 65.96 | 87000000 | 120000000 | 309000 | 1679.50 | 45.69 | 1.00 | 5974.58 | 21.85 |
| 17 | 2005 | 33.06 | 43.54 | 59.54 | 63.04 | 96000000 | 130000000 | 341000 | 1843.63 | 47.59 | 1.00 | 6177.94 | 2.60 |
| 17 | 2006 | 35.06 | 44.00 | 61.46 | 64.67 | 110000000 | 140000000 | 388000 | 2271.09 | 46.29 | 1.00 | 5635.09 | 0.40 |
| 17 | 2007 | 35.29 | 44.38 | 62.63 | 63.58 | 120000000 | 180000000 | 416000 | 2976.83 | 43.37 | 1.00 | 5032.71 | 1.02 |
| 17 | 2008 | 34.25 | 43.67 | 61.21 | 63.63 | 130000000 | 210000000 | 428000 | 4041.58 | 39.86 | 1.00 | 4363.29 | 61.59 |
| 17 | 2009 | 35.37 | 43.88 | 60.42 | 66.46 | 230000000 | 230000000 | 439000 | 3624.57 | 36.24 | 1.00 | 4966.68 | 0.83 |
| 17 | 2010 | 33.6 | 44.96 | 57.46 | 64.79 | 240000000 | 260000000 | 465000 | 4355.94 | 40.43 | 1.00 | 4758.43 | 1.94 |
| 17 | 2011 | 33.67 | 45.00 | 58.13 | 64.21 | 260000000 | 310000000 | 524000 | 5322.96 | 39.21 | 1.00 | 4193.80 | 23.93 |
| 17 | 2012 | 35.75 | 45.50 | 58.75 | 67.25 | 290000000 | 360000000 | 579000 | 5183.08 | 36.88 | 1.00 | 4421.66 | 1.10 |
| 17 | 2013 | 32.44 | 43.88 | 57.13 | 63.88 | 300000000 | 430000000 | 610000 | 5926.83 | 37.17 | 1.00 | 4303.88 | 0.36 |
| 17 | 2014 | 30.79 | 40.75 | 57.58 | 63.25 | 310000000 | 450000000 | 649000 | 6102.94 | 34.81 | 1.00 | 4462.19 | 10.23 |
| 17 | 2015 | 31.46 | 40.42 | 59.33 | 63.17 | 350000000 | 490000000 | 1200000 | 5406.70 | 33.00 | 1.00 | 5204.92 | 0.50 |
| 17 | 2016 | 31.87 | 41.58 | 58.17 | 64.00 | 360000000 | 510000000 | 1300000 | 5319.41 | 35.38 | 1.00 | 5670.54 | 10.25 |
| 17 | 2017 | 30.37 | 40.96 | 56.83 | 62.96 | 400000000 | 550000000 | 1600000 | 5680.58 | 36.38 | 1.00 | 5618.93 | 17.76 |
| 17 | 2018 | 30.31 | 40.79 | 56.75 | 63.08 | 390000000 | 550000000 | 1200000 | 5805.68 | 35.92 | 1.00 | 5732.10 | 1.21 |
| 17 | 2019 | 31.9 | 40.33 | 59.96 | 63.50 | 410000000 | 550000000 | 4400000 | 5414.80 | 34.67 | 1.00 | 5845.28 | 21.94 |
| 18 | 2000 | 28.25 | 36.00 | 57.50 | 63.00 | 850000000 | 430000000 | 3900000 | 13636.40 | 79.18 | 0.00 | 0.38 | 7.00 |
| 18 | 2001 | 23.58 | 28.50 | 56.17 | 62.50 | 890000000 | 420000000 | 4400000 | 12868.30 | 73.61 | 0.00 | 0.38 | 17.69 |
| 18 | 2002 | 20.21 | 22.63 | 56.00 | 61.79 | 990000000 | 550000000 | 4800000 | 13049.80 | 72.51 | 0.00 | 0.38 | 2.24 |
| 18 | 2003 | 19.92 | 22.33 | 56.00 | 61.50 | 1200000000 | 490000000 | 4800000 | 14222.00 | 72.06 | 0.00 | 0.38 | 39.84 |
| 18 | 2004 | 20.52 | 23.54 | 56.00 | 61.50 | 1500000000 | 530000000 | 5700000 | 15846.50 | 78.61 | 0.00 | 0.38 | 6.58 |
| 18 | 2005 | 19.98 | 25.46 | 56.50 | 58.00 | 1600000000 | 570000000 | 6300000 | 17959.30 | 83.89 | 1.00 | 0.38 | 0.34 |
| 18 | 2006 | 18.06 | 26.13 | 56.50 | 53.50 | 1800000000 | 640000000 | 7300000 | 19307.60 | 84.64 | 1.00 | 0.38 | 28.92 |
| 18 | 2007 | 17.54 | 26.58 | 56.50 | 52.00 | 1900000000 | 670000000 | 7800000 | 20976.50 | 79.68 | 1.00 | 0.38 | 0.22 |
| 18 | 2008 | 17.87 | 27.25 | 57.75 | 50.75 | 1900000000 | 700000000 | 8600000 | 23066.50 | 82.58 | 1.00 | 0.38 | 32.34 |
| 18 | 2009 | 21.81 | 28.00 | 58.29 | 57.33 | 1900000000 | 600000000 | 8900000 | 19355.90 | 68.47 | 1.00 | 0.38 | 0.23 |
| 18 | 2010 | 21.6 | 27.92 | 58.29 | 57.00 | 2200000000 | 680000000 | 12000000 | 20722.10 | 69.54 | 1.00 | 0.38 | 58.79 |
| 18 | 2011 | 23.44 | 30.88 | 59.50 | 56.50 | 1800000000 | 900000000 | 6700000 | 22514.20 | 99.42 | 1.00 | 0.38 | 2.04 |
| 18 | 2012 | 27.15 | 33.04 | 61.38 | 59.88 | 1800000000 | 980000000 | 8100000 | 23654.40 | 102.15 | 2.00 | 0.38 | 0.14 |
| 18 | 2013 | 30.31 | 36.13 | 64.33 | 60.17 | 1900000000 | 1000000000 | 9200000 | 24744.30 | 104.81 | 2.00 | 0.38 | 8.97 |
| 18 | 2014 | 30.02 | 37.04 | 63.50 | 59.50 | 1900000000 | 760000000 | 10000000 | 24989.40 | 96.05 | 2.00 | 0.38 | 0.00 |
| 18 | 2015 | 30.54 | 36.58 | 61.17 | 63.33 | 2800000000 | 4900000000 | 9700000 | 22688.90 | 82.91 | 2.00 | 0.38 | 3.79 |
| 18 | 2016 | 33.52 | 38.17 | 60.13 | 68.75 | 4000000000 | 3300000000 | 10000000 | 22619.10 | 74.17 | 2.00 | 0.38 | 2.37 |
| 18 | 2017 | 34.48 | 38.96 | 62.00 | 68.00 | 4400000000 | 3200000000 | 11000000 | 23709.40 | 75.89 | 2.00 | 0.38 | 0.26 |
| 18 | 2018 | 33.19 | 39.58 | 63.54 | 63.25 | 3800000000 | 2500000000 | 12000000 | 23991.10 | 79.57 | 2.00 | 0.38 | 11.90 |
| 18 | 2019 | 33.83 | 38.67 | 64.33 | 64.67 | 3900000000 | 2600000000 | 11000000 | 23504.00 | 83.25 | 2.00 | 0.38 | 2.63 |
| 19 | 2000 | 26.79 | 24.25 | 66.25 | 63.08 | 630000000 | 240000000 | 484000 | 4060.32 | 62.01 | 5.00 | 1.00 | 6.71 |
| 19 | 2001 | 26.75 | 24.92 | 65.58 | 63.00 | 670000000 | 230000000 | 519000 | 4046.42 | 62.14 | 5.00 | 1.00 | 1.71 |
| 19 | 2002 | 28.71 | 27.33 | 65.50 | 64.58 | 710000000 | 250000000 | 534000 | 4126.24 | 57.64 | 5.00 | 1.00 | 0.13 |
| 19 | 2003 | 28.58 | 27.79 | 65.13 | 64.25 | 800000000 | 270000000 | 566000 | 4267.30 | 54.34 | 6.00 | 1.00 | 0.32 |
| 19 | 2004 | 26.77 | 26.50 | 64.79 | 62.25 | 900000000 | 290000000 | 621000 | 4592.15 | 57.77 | 6.00 | 1.00 | 72.84 |
| 19 | 2005 | 26.42 | 25.29 | 64.58 | 62.96 | 1100000000 | 390000000 | 702000 | 4916.92 | 64.50 | 7.00 | 1.00 | 19.37 |
| 19 | 2006 | 26.79 | 25.50 | 64.75 | 63.33 | 1400000000 | 400000000 | 843000 | 5348.90 | 66.17 | 7.00 | 1.00 | 46.59 |
| 19 | 2007 | 25.81 | 25.50 | 64.04 | 62.08 | 1800000000 | 460000000 | 1100000 | 6166.18 | 71.46 | 7.00 | 1.00 | 70.57 |
| 19 | 2008 | 25.23 | 25.79 | 63.75 | 60.92 | 2200000000 | 560000000 | 1200000 | 7154.27 | 78.09 | 7.00 | 1.00 | 61.40 |
| 19 | 2009 | 27.06 | 25.38 | 64.88 | 63.88 | 2300000000 | 500000000 | 1200000 | 7576.14 | 69.81 | 7.00 | 1.00 | 1.90 |
| 19 | 2010 | 25.75 | 24.38 | 63.38 | 63.75 | 2600000000 | 580000000 | 1300000 | 8082.03 | 70.04 | 7.00 | 1.00 | 17.90 |
| 19 | 2011 | 26.02 | 25.71 | 63.13 | 63.21 | 3600000000 | 680000000 | 1500000 | 9358.25 | 78.23 | 7.00 | 1.00 | 0.18 |
| 19 | 2012 | 27.19 | 29.63 | 61.50 | 63.25 | 4600000000 | 590000000 | 1600000 | 10722.30 | 75.19 | 7.00 | 1.00 | 1.01 |
| 19 | 2013 | 27.67 | 30.54 | 60.96 | 63.83 | 5400000000 | 1100000000 | 1700000 | 11889.10 | 65.16 | 7.00 | 1.00 | 28.32 |
| 19 | 2014 | 28.42 | 29.75 | 63.33 | 63.75 | 5700000000 | 1100000000 | 1700000 | 12796.10 | 55.36 | 7.00 | 1.00 | 6.38 |
| 19 | 2015 | 26.79 | 27.67 | 62.25 | 63.67 | 5800000000 | 1300000000 | 2100000 | 13630.30 | 47.69 | 7.00 | 1.00 | 1.05 |
| 19 | 2016 | 26.67 | 28.96 | 61.75 | 62.63 | 6300000000 | 1400000000 | 1900000 | 14344.00 | 41.97 | 7.00 | 1.00 | 40.14 |
| 19 | 2017 | 24.98 | 28.25 | 60.67 | 61.04 | 6800000000 | 1100000000 | 1800000 | 15150.30 | 41.78 | 7.00 | 1.00 | 27.84 |
| 19 | 2018 | 25.17 | 29.21 | 60.63 | 60.50 | 7100000000 | 1400000000 | 1800000 | 15592.60 | 42.54 | 7.00 | 1.00 | 0.44 |
| 19 | 2019 | 25.46 | 27.71 | 61.38 | 61.83 | 7100000000 | 1600000000 | 2500000 | 15731.00 | 39.69 | 7.00 | 1.00 | 4.16 |
| 20 | 2000 | 35.6 | 36.33 | 68.54 | 66.33 | 2000000000 | 4500000000 | 5300000 | 3749.75 | 10.19 | 15.00 | 1.83 | 7.53 |
| 20 | 2001 | 36.56 | 34.88 | 72.25 | 66.00 | 1800000000 | 3800000000 | 4800000 | 3156.80 | 12.37 | 18.00 | 2.35 | 0.22 |
| 20 | 2002 | 37.33 | 37.29 | 71.21 | 66.17 | 2100000000 | 2900000000 | 3800000 | 2829.28 | 14.23 | 18.00 | 2.92 | 22.00 |
| 20 | 2003 | 35.17 | 30.92 | 71.67 | 67.75 | 2700000000 | 2900000000 | 4100000 | 3070.91 | 15.18 | 19.00 | 3.08 | 5.25 |
| 20 | 2004 | 32.17 | 32.92 | 66.96 | 64.46 | 3400000000 | 3800000000 | 4800000 | 3637.46 | 16.55 | 19.00 | 2.93 | 11.38 |
| 20 | 2005 | 30.77 | 34.29 | 64.38 | 62.88 | 4200000000 | 5900000000 | 5400000 | 4790.44 | 15.24 | 19.00 | 2.43 | 3.39 |
| 20 | 2006 | 29.02 | 34.04 | 61.58 | 62.42 | 4600000000 | 7500000000 | 5000000 | 5886.46 | 14.37 | 19.00 | 2.18 | 4.91 |
| 20 | 2007 | 28.37 | 34.54 | 61.13 | 61.08 | 5300000000 | 10000000000 | 5000000 | 7348.03 | 13.33 | 19.00 | 1.95 | 15.33 |
| 20 | 2008 | 29.02 | 32.88 | 63.21 | 61.96 | 6100000000 | 13000000000 | 5100000 | 8831.02 | 13.53 | 19.00 | 1.83 | 0.38 |
| 20 | 2009 | 30.29 | 31.83 | 63.58 | 65.17 | 5600000000 | 13000000000 | 4800000 | 8597.92 | 10.85 | 19.00 | 2.00 | 1.87 |
| 20 | 2010 | 25.56 | 31.83 | 56.88 | 62.42 | 5500000000 | 19000000000 | 5200000 | 11286.20 | 10.87 | 20.00 | 1.76 | 11.42 |
| 20 | 2011 | 27.37 | 32.46 | 58.88 | 63.42 | 6400000000 | 25000000000 | 5400000 | 13245.60 | 11.58 | 20.00 | 1.67 | 20.39 |
| 20 | 2012 | 27.98 | 31.42 | 60.83 | 63.71 | 6600000000 | 26000000000 | 5700000 | 12370.00 | 11.88 | 21.00 | 1.95 | 14.67 |
| 20 | 2013 | 28.69 | 33.54 | 59.88 | 63.96 | 6800000000 | 29000000000 | 5800000 | 12300.30 | 11.74 | 21.00 | 2.16 | 43.08 |
| 20 | 2014 | 31 | 35.54 | 60.63 | 65.83 | 7400000000 | 30000000000 | 6400000 | 12112.60 | 11.01 | 21.00 | 2.35 | 47.00 |
| 20 | 2015 | 35.71 | 38.29 | 64.38 | 68.75 | 6300000000 | 20000000000 | 6300000 | 8814.00 | 12.90 | 21.00 | 3.33 | 22.39 |
| 20 | 2016 | 36.25 | 38.04 | 62.63 | 71.83 | 6600000000 | 17000000000 | 6500000 | 8710.10 | 12.47 | 22.00 | 3.49 | 59.08 |
| 20 | 2017 | 31.94 | 36.88 | 58.96 | 68.04 | 6200000000 | 23000000000 | 6600000 | 9925.39 | 12.52 | 23.00 | 3.19 | 16.67 |
| 20 | 2018 | 31.44 | 36.50 | 60.54 | 65.83 | 6300000000 | 22000000000 | 6600000 | 9001.23 | 14.89 | 23.00 | 3.65 | 2.09 |
| 20 | 2019 | 29.42 | 32.96 | 59.88 | 66.00 | 6100000000 | 21000000000 | 6400000 | 8717.19 | 14.32 | 23.00 | 4.12 | 6.10 |
| 21 | 2000 | 41.48 | 42.83 | 66.67 | 73.46 | 190000000 | 250000000 | 60000 | 1276.29 | 69.21 | 3.00 | 0.09 | 1.96 |
| 21 | 2001 | 39.48 | 40.29 | 67.29 | 71.38 | 270000000 | 490000000 | 61000 | 1244.37 | 66.75 | 3.00 | 0.14 | 0.52 |
| 21 | 2002 | 38.37 | 40.04 | 66.96 | 69.75 | 300000000 | 530000000 | 63000 | 1479.31 | 63.63 | 3.00 | 0.18 | 0.50 |
| 21 | 2003 | 35.46 | 38.17 | 63.13 | 69.63 | 340000000 | 440000000 | 64000 | 1819.77 | 65.16 | 3.00 | 0.21 | 4.63 |
| 21 | 2004 | 32.5 | 36.46 | 60.92 | 67.63 | 360000000 | 500000000 | 67000 | 2378.62 | 67.89 | 3.00 | 0.22 | 17.76 |
| 21 | 2005 | 31.62 | 38.75 | 59.50 | 65.00 | 350000000 | 520000000 | 91000 | 3125.81 | 59.80 | 5.00 | 0.22 | 28.04 |
| 21 | 2006 | 32.23 | 39.96 | 59.50 | 65.00 | 400000000 | 680000000 | 89000 | 3847.43 | 60.06 | 5.00 | 0.21 | 1.54 |
| 21 | 2007 | 32.25 | 40.00 | 59.50 | 65.00 | 480000000 | 720000000 | 105000 | 4735.66 | 60.94 | 5.00 | 0.21 | 2.13 |
| 21 | 2008 | 32.94 | 38.67 | 60.75 | 66.46 | 590000000 | 860000000 | 90500 | 6377.37 | 60.94 | 5.00 | 0.21 | 56.48 |
| 21 | 2009 | 37.69 | 36.71 | 67.67 | 71.00 | 560000000 | 750000000 | 95000 | 5351.36 | 48.88 | 5.00 | 0.28 | 6.03 |
| 21 | 2010 | 36.37 | 36.29 | 65.17 | 71.29 | 670000000 | 750000000 | 119300 | 6029.40 | 51.37 | 5.00 | 0.30 | 1.85 |
| 21 | 2011 | 41.77 | 43.50 | 71.42 | 68.63 | 750000000 | 730000000 | 116000 | 6519.23 | 78.47 | 5.00 | 0.50 | 0.48 |
| 21 | 2012 | 44.77 | 46.88 | 70.92 | 71.75 | 990000000 | 950000000 | 6100000 | 6940.16 | 78.78 | 5.00 | 0.83 | 22.10 |
| 21 | 2013 | 40.9 | 46.25 | 64.71 | 70.83 | 1200000000 | 1300000000 | 6200000 | 7978.87 | 58.33 | 5.00 | 0.89 | 4.60 |
| 21 | 2014 | 40.77 | 45.96 | 66.42 | 69.17 | 1200000000 | 1300000000 | 5400000 | 8318.51 | 54.94 | 6.00 | 1.02 | 0.97 |
| 21 | 2015 | 42.52 | 44.00 | 71.13 | 69.92 | 1000000000 | 1000000000 | 4400000 | 5949.11 | 58.01 | 6.00 | 1.59 | 2.20 |
| 21 | 2016 | 39.83 | 42.13 | 68.29 | 69.25 | 1000000000 | 890000000 | 11000000 | 5022.63 | 62.51 | 6.00 | 1.99 | 11.91 |
| 21 | 2017 | 37.48 | 42.25 | 64.13 | 68.58 | 1100000000 | 1100000000 | 11000000 | 5761.75 | 66.79 | 6.00 | 1.93 | 29.48 |
| 21 | 2018 | 34.87 | 39.13 | 63.29 | 67.33 | 1200000000 | 1200000000 | 12000000 | 6330.08 | 70.46 | 6.00 | 2.04 | 7.26 |
| 21 | 2019 | 34.54 | 39.13 | 62.96 | 67.00 | 1300000000 | 1200000000 | 12000000 | 6663.30 | 66.43 | 6.00 | 2.14 | 9.69 |
| 22 | 2000 | 30.79 | 29.33 | 68.04 | 64.21 | 1400000000 | 760000000 | 2800000 | 1621.24 | 36.22 | 9.00 | 2.12 | 4.04 |
| 22 | 2001 | 28.92 | 28.33 | 64.46 | 65.04 | 1300000000 | 840000000 | 3200000 | 1770.91 | 34.88 | 9.00 | 2.18 | 0.11 |
| 22 | 2002 | 28 | 26.08 | 64.13 | 65.79 | 1400000000 | 1000000000 | 3400000 | 2092.96 | 33.65 | 9.00 | 2.08 | 34.31 |
| 22 | 2003 | 28.46 | 27.83 | 64.17 | 64.92 | 2100000000 | 1500000000 | 4000000 | 2719.50 | 34.42 | 9.00 | 1.73 | 45.32 |
| 22 | 2004 | 29.04 | 30.79 | 62.75 | 64.54 | 2800000000 | 1900000000 | 4600000 | 3389.71 | 40.96 | 9.00 | 1.58 | 0.37 |
| 22 | 2005 | 28.33 | 28.96 | 62.92 | 64.79 | 3100000000 | 1900000000 | 4800000 | 3899.91 | 42.53 | 9.00 | 1.57 | 42.70 |
| 22 | 2006 | 27.83 | 27.83 | 63.75 | 64.08 | 3300000000 | 2100000000 | 5200000 | 4523.05 | 46.98 | 9.00 | 1.56 | 57.00 |
| 22 | 2007 | 28.06 | 26.96 | 64.75 | 64.42 | 4200000000 | 2100000000 | 5200000 | 5885.10 | 52.39 | 10.00 | 1.43 | 1.37 |
| 22 | 2008 | 33.12 | 28.50 | 68.58 | 69.17 | 4900000000 | 2600000000 | 5800000 | 7265.74 | 52.52 | 10.00 | 1.34 | 20.00 |
| 22 | 2009 | 36.77 | 31.75 | 70.46 | 71.33 | 4300000000 | 2000000000 | 5700000 | 6984.97 | 42.24 | 10.00 | 1.41 | 5.04 |
| 22 | 2010 | 34.12 | 31.50 | 68.50 | 68.25 | 3800000000 | 980000000 | 6000000 | 6809.90 | 50.42 | 11.00 | 1.48 | 2.11 |
| 22 | 2011 | 32.52 | 34.00 | 66.04 | 65.00 | 4300000000 | 1100000000 | 6300000 | 7806.35 | 59.13 | 12.00 | 1.41 | 21.55 |
| 22 | 2012 | 31.25 | 35.04 | 63.50 | 63.96 | 4000000000 | 1100000000 | 6500000 | 7393.19 | 60.67 | 12.00 | 1.52 | 9.99 |
| 22 | 2013 | 30.06 | 34.79 | 61.75 | 63.58 | 4400000000 | 1300000000 | 6900000 | 7651.82 | 64.84 | 12.00 | 1.47 | 13.21 |
| 22 | 2014 | 29.69 | 35.50 | 60.75 | 63.13 | 4500000000 | 1500000000 | 7300000 | 7874.26 | 64.83 | 12.00 | 1.47 | 5.70 |
| 22 | 2015 | 30.17 | 32.21 | 64.50 | 63.63 | 3600000000 | 1300000000 | 7100000 | 7053.60 | 63.99 | 12.00 | 1.76 | 12.04 |
| 22 | 2016 | 26.31 | 30.29 | 61.08 | 61.25 | 4200000000 | 1600000000 | 8300000 | 7545.79 | 64.07 | 12.00 | 1.77 | 49.97 |
| 22 | 2017 | 25.12 | 29.96 | 60.21 | 60.08 | 4700000000 | 2000000000 | 8900000 | 8331.06 | 67.30 | 13.00 | 1.74 | 83.14 |
| 22 | 2018 | 22.98 | 27.50 | 59.25 | 59.21 | 5100000000 | 2200000000 | 9300000 | 9423.56 | 65.86 | 13.00 | 1.66 | 24.87 |
| 22 | 2019 | 22.73 | 27.83 | 58.92 | 58.71 | 4800000000 | 2200000000 | 13000000 | 9828.15 | 64.19 | 13.00 | 1.58 | 3.97 |
| 23 | 2000 | 20.6 | 21.92 | 63.21 | 56.08 | 8000000000 | 10000000000 | 6500000 | 23041.50 | 72.55 | 8.00 | 0.66 | 3.35 |
| 23 | 2001 | 16.33 | 15.42 | 61.13 | 56.13 | 8300000000 | 11000000000 | 6500000 | 22995.20 | 71.61 | 8.00 | 0.69 | 0.37 |
| 23 | 2002 | 15.79 | 13.25 | 60.79 | 57.54 | 7600000000 | 11000000000 | 6700000 | 24887.60 | 70.83 | 8.00 | 0.67 | 76.26 |
| 23 | 2003 | 15.62 | 13.96 | 59.92 | 57.38 | 8800000000 | 13000000000 | 6700000 | 30587.70 | 69.04 | 8.00 | 0.61 | 55.90 |
| 23 | 2004 | 15.27 | 14.42 | 58.63 | 57.50 | 10000000000 | 15000000000 | 6700000 | 35364.40 | 70.91 | 8.00 | 0.55 | 1.20 |
| 23 | 2005 | 16.23 | 16.13 | 58.71 | 57.63 | 11000000000 | 17000000000 | 6700000 | 36796.00 | 74.28 | 9.00 | 0.55 | 7.90 |
| 23 | 2006 | 16.67 | 16.00 | 59.46 | 57.88 | 12000000000 | 18000000000 | 7000000 | 38672.70 | 76.81 | 9.00 | 0.54 | 8.30 |
| 23 | 2007 | 17.15 | 18.46 | 59.08 | 56.75 | 12000000000 | 19000000000 | 7000000 | 44262.90 | 78.30 | 10.00 | 0.50 | 4.46 |
| 23 | 2008 | 17.77 | 17.88 | 59.67 | 58.00 | 13000000000 | 20000000000 | 7200000 | 48106.90 | 80.88 | 10.00 | 0.54 | 36.14 |
| 23 | 2009 | 20.17 | 16.21 | 60.83 | 63.29 | 13000000000 | 21000000000 | 6800000 | 44583.50 | 68.83 | 11.00 | 0.64 | 4.86 |
| 23 | 2010 | 20.12 | 16.92 | 60.58 | 62.75 | 13000000000 | 21000000000 | 7200000 | 44141.90 | 75.85 | 11.00 | 0.65 | 69.22 |
| 23 | 2011 | 20.83 | 19.21 | 61.17 | 61.29 | 14000000000 | 23000000000 | 7500000 | 47348.50 | 80.70 | 12.00 | 0.62 | 2.44 |
| 23 | 2012 | 24.48 | 21.58 | 64.50 | 62.88 | 14000000000 | 22000000000 | 7600000 | 44673.10 | 80.40 | 13.00 | 0.63 | 0.46 |
| 23 | 2013 | 23.85 | 20.96 | 64.00 | 62.75 | 15000000000 | 25000000000 | 7700000 | 46744.70 | 79.32 | 13.00 | 0.64 | 0.13 |
| 23 | 2014 | 23.48 | 21.13 | 64.21 | 61.63 | 15000000000 | 26000000000 | 7900000 | 47700.50 | 79.80 | 13.00 | 0.61 | 7.39 |
| 23 | 2015 | 25.02 | 22.21 | 66.92 | 60.92 | 9000000000 | 16000000000 | 8400000 | 40991.80 | 77.81 | 13.00 | 0.65 | 20.33 |
| 23 | 2016 | 23.6 | 22.42 | 64.25 | 60.54 | 8800000000 | 17000000000 | 7500000 | 41998.30 | 79.40 | 14.00 | 0.74 | 5.24 |
| 23 | 2017 | 22.52 | 21.04 | 63.92 | 60.08 | 9600000000 | 18000000000 | 8400000 | 44288.50 | 82.39 | 15.00 | 0.78 | 11.56 |
| 23 | 2018 | 22.56 | 20.92 | 64.50 | 59.71 | 10000000000 | 21000000000 | 9100000 | 47491.30 | 82.63 | 15.00 | 0.75 | 2.00 |
| 23 | 2019 | 22.58 | 21.21 | 64.25 | 59.71 | 11000000000 | 21000000000 | 9300000 | 46420.70 | 81.84 | 15.00 | 0.72 | 1.65 |
| 24 | 2000 | 19.87 | 11.67 | 65.67 | 62.42 | 390000000 | 480000000 | 303000 | 32018.10 | 32.20 | 0.00 | 78.62 | 65.50 |
| 24 | 2001 | 21.5 | 8.50 | 70.21 | 64.29 | 380000000 | 500000000 | 301000 | 28794.00 | 37.06 | 0.00 | 97.42 | 64.35 |
| 24 | 2002 | 21 | 8.50 | 69.21 | 64.29 | 420000000 | 370000000 | 285000 | 32326.60 | 35.53 | 0.00 | 91.66 | 23.60 |
| 24 | 2003 | 20.35 | 9.42 | 69.04 | 62.25 | 490000000 | 520000000 | 317000 | 39423.90 | 32.66 | 0.00 | 76.71 | 4.80 |
| 24 | 2004 | 18.29 | 10.08 | 67.17 | 59.33 | 560000000 | 700000000 | 360000 | 47367.30 | 32.38 | 1.00 | 70.19 | 0.45 |
| 24 | 2005 | 15.67 | 9.00 | 65.33 | 57.00 | 640000000 | 990000000 | 374000 | 56658.60 | 30.40 | 1.00 | 62.98 | 1.35 |
| 24 | 2006 | 16.67 | 9.92 | 66.42 | 57.00 | 700000000 | 1100000000 | 422000 | 56673.60 | 30.82 | 1.00 | 70.18 | 64.20 |
| 24 | 2007 | 23.9 | 9.33 | 71.88 | 66.58 | 850000000 | 1300000000 | 485000 | 69054.30 | 33.02 | 1.00 | 64.06 | 1.19 |
| 24 | 2008 | 25.37 | 11.17 | 74.33 | 65.25 | 880000000 | 1600000000 | 502000 | 56409.80 | 40.63 | 2.00 | 87.95 | 2.66 |
| 24 | 2009 | 38.48 | 17.25 | 79.58 | 80.13 | 910000000 | 1800000000 | 494000 | 41333.40 | 48.65 | 2.00 | 123.64 | 34.30 |
| 24 | 2010 | 31.25 | 17.42 | 72.83 | 72.25 | 950000000 | 2100000000 | 489000 | 43024.90 | 51.98 | 2.00 | 122.24 | 1.80 |
| 24 | 2011 | 30.58 | 16.54 | 72.13 | 72.50 | 980000000 | 2300000000 | 566000 | 47516.90 | 54.76 | 2.00 | 115.95 | 0.59 |
| 24 | 2012 | 26.85 | 16.29 | 69.29 | 68.13 | 1000000000 | 2600000000 | 673000 | 45910.00 | 55.02 | 2.00 | 125.08 | 19.24 |
| 24 | 2013 | 21.04 | 15.75 | 64.00 | 62.33 | 1000000000 | 2800000000 | 807300 | 49522.20 | 53.49 | 2.00 | 122.18 | 0.76 |
| 24 | 2014 | 20.21 | 15.21 | 64.17 | 61.04 | 1100000000 | 3100000000 | 997000 | 54241.90 | 51.52 | 2.00 | 116.77 | 22.40 |
| 24 | 2015 | 19.04 | 14.21 | 66.63 | 57.25 | 1100000000 | 3400000000 | 1300000 | 52564.40 | 51.80 | 2.00 | 131.92 | 22.75 |
| 24 | 2016 | 18.73 | 14.38 | 66.25 | 56.83 | 1100000000 | 3600000000 | 1800000 | 61466.80 | 47.64 | 2.00 | 120.81 | 2.94 |
| 24 | 2017 | 18.44 | 13.75 | 66.08 | 57.04 | 1200000000 | 3900000000 | 2200000 | 71310.90 | 46.11 | 2.00 | 106.84 | 26.98 |
| 24 | 2018 | 16.19 | 14.67 | 64.21 | 53.50 | 1200000000 | 4100000000 | 2300000 | 72968.70 | 47.52 | 2.00 | 108.30 | 8.00 |
| 24 | 2019 | 17.5 | 14.96 | 62.92 | 57.13 | 1200000000 | 4400000000 | 2200000 | 66944.80 | 45.32 | 2.00 | 109.76 | 25.91 |
| 25 | 2000 | 25.92 | 24.75 | 62.46 | 64.63 | 6100000000 | 3400000000 | 17000000 | 4492.73 | 27.23 | 10.00 | 4.35 | 2.81 |
| 25 | 2001 | 24.54 | 22.04 | 61.79 | 65.25 | 5100000000 | 3600000000 | 15000000 | 4981.20 | 27.23 | 11.00 | 4.09 | 54.55 |
| 25 | 2002 | 24.06 | 21.75 | 60.75 | 65.63 | 5000000000 | 3400000000 | 14000000 | 5196.93 | 28.76 | 11.00 | 4.08 | 8.90 |
| 25 | 2003 | 24.4 | 23.42 | 60.75 | 64.63 | 4700000000 | 3300000000 | 14000000 | 5693.38 | 33.39 | 12.00 | 3.89 | 0.42 |
| 25 | 2004 | 25 | 25.79 | 61.33 | 62.88 | 6500000000 | 5100000000 | 14000000 | 6681.18 | 34.26 | 13.00 | 3.66 | 31.64 |
| 25 | 2005 | 25.1 | 25.00 | 62.46 | 62.75 | 7200000000 | 5900000000 | 15000000 | 8020.96 | 34.61 | 13.00 | 3.24 | 0.28 |
| 25 | 2006 | 23.08 | 24.38 | 61.00 | 60.79 | 8200000000 | 7600000000 | 16000000 | 9038.66 | 37.86 | 14.00 | 3.10 | 34.97 |
| 25 | 2007 | 23.96 | 25.29 | 61.79 | 60.83 | 12000000000 | 8300000000 | 15000000 | 11255.40 | 38.56 | 14.00 | 2.77 | 0.31 |
| 25 | 2008 | 23.5 | 20.42 | 64.21 | 62.38 | 13000000000 | 11000000000 | 13000000 | 14001.40 | 37.86 | 15.00 | 2.41 | 61.70 |
| 25 | 2009 | 26.73 | 19.38 | 67.92 | 66.17 | 9900000000 | 7900000000 | 12000000 | 11527.50 | 37.18 | 15.00 | 3.12 | 5.80 |
| 25 | 2010 | 24.6 | 20.04 | 65.00 | 64.17 | 9900000000 | 8900000000 | 12000000 | 12599.50 | 40.06 | 15.00 | 3.02 | 0.16 |
| 25 | 2011 | 26.1 | 21.21 | 66.21 | 64.79 | 11000000000 | 8700000000 | 13000000 | 13893.50 | 42.56 | 15.00 | 2.96 | 11.60 |
| 25 | 2012 | 26.33 | 23.46 | 64.46 | 64.75 | 12000000000 | 8900000000 | 15000000 | 13145.50 | 44.44 | 15.00 | 3.26 | 0.02 |
| 25 | 2013 | 25.67 | 25.58 | 61.67 | 64.08 | 12000000000 | 8900000000 | 16000000 | 13781.10 | 46.32 | 17.00 | 3.16 | 7.41 |
| 25 | 2014 | 24.17 | 24.50 | 61.58 | 62.25 | 13000000000 | 9200000000 | 16000000 | 14347.90 | 47.57 | 18.00 | 3.15 | 3.35 |
| 25 | 2015 | 24.06 | 22.13 | 64.13 | 61.88 | 11000000000 | 8300000000 | 17000000 | 12572.40 | 49.50 | 18.00 | 3.77 | 0.42 |
| 25 | 2016 | 21.23 | 21.04 | 61.29 | 60.13 | 12000000000 | 8500000000 | 17000000 | 12431.80 | 52.19 | 18.00 | 3.94 | 12.90 |
| 25 | 2017 | 22.48 | 22.63 | 61.42 | 60.92 | 14000000000 | 9400000000 | 18000000 | 13861.30 | 54.34 | 19.00 | 3.78 | 3.36 |
| 25 | 2018 | 22.85 | 23.58 | 61.33 | 60.79 | 16000000000 | 10000000000 | 20000000 | 15460.60 | 55.45 | 19.00 | 3.61 | 7.01 |
| 25 | 2019 | 21.92 | 22.38 | 60.92 | 60.54 | 16000000000 | 10000000000 | 89000000 | 15692.50 | 55.54 | 19.00 | 3.44 | 1.88 |
| 26 | 2000 | 31.81 | 31.75 | 65.42 | 66.46 | 100000000 | 120000000 | 319000 | 997.58 | 18.27 | 6.00 | 6.18 | 0.16 |
| 26 | 2001 | 32.08 | 31.13 | 65.25 | 67.79 | 120000000 | 110000000 | 316000 | 948.87 | 19.97 | 6.00 | 6.61 | 0.56 |
| 26 | 2002 | 33.48 | 33.83 | 65.63 | 67.50 | 140000000 | 110000000 | 334000 | 904.23 | 21.63 | 6.00 | 7.17 | 78.13 |
| 26 | 2003 | 33.35 | 37.13 | 63.21 | 66.38 | 240000000 | 200000000 | 427000 | 907.54 | 25.60 | 6.00 | 7.66 | 29.67 |
| 26 | 2004 | 34.12 | 38.13 | 62.58 | 67.54 | 280000000 | 230000000 | 480000 | 967.41 | 31.14 | 6.00 | 7.94 | 48.44 |
| 26 | 2005 | 34.1 | 42.50 | 60.88 | 64.83 | 350000000 | 260000000 | 524000 | 1034.31 | 35.55 | 6.00 | 8.07 | 79.13 |
| 26 | 2006 | 33.06 | 42.33 | 60.04 | 63.75 | 330000000 | 360000000 | 521000 | 1218.87 | 41.77 | 6.00 | 8.01 | 65.10 |
| 26 | 2007 | 33.04 | 42.88 | 59.79 | 63.42 | 330000000 | 390000000 | 573000 | 1372.63 | 41.80 | 6.00 | 7.85 | 2.50 |
| 26 | 2008 | 29.83 | 40.54 | 56.92 | 62.21 | 300000000 | 380000000 | 594000 | 1715.21 | 44.91 | 6.00 | 7.24 | 20.70 |
| 26 | 2009 | 30.02 | 39.25 | 56.04 | 64.75 | 310000000 | 390000000 | 599000 | 1754.21 | 35.72 | 6.00 | 7.02 | 0.19 |
| 26 | 2010 | 27.54 | 40.25 | 55.17 | 59.67 | 340000000 | 420000000 | 679000 | 1955.46 | 41.19 | 6.00 | 7.02 | 2.10 |
| 26 | 2011 | 29.6 | 42.75 | 55.00 | 61.46 | 500000000 | 410000000 | 711000 | 2346.34 | 44.09 | 6.00 | 6.94 | 30.09 |
| 26 | 2012 | 29.08 | 43.46 | 54.38 | 60.33 | 630000000 | 500000000 | 798000 | 2609.88 | 47.17 | 6.00 | 6.91 | 8.00 |
| 26 | 2013 | 28.71 | 42.25 | 54.33 | 60.83 | 640000000 | 660000000 | 798000 | 2908.20 | 44.09 | 6.00 | 6.91 | 1.46 |
| 26 | 2014 | 28.08 | 41.00 | 54.50 | 60.67 | 750000000 | 950000000 | 871000 | 3081.88 | 43.29 | 7.00 | 6.91 | 43.04 |
| 26 | 2015 | 28.56 | 38.83 | 54.92 | 63.38 | 810000000 | 850000000 | 882000 | 3035.97 | 30.87 | 7.00 | 6.91 | 31.85 |
| 26 | 2016 | 32.27 | 41.38 | 57.33 | 65.83 | 830000000 | 960000000 | 961000 | 3076.66 | 24.49 | 7.00 | 6.91 | 0.54 |
| 26 | 2017 | 32.52 | 41.29 | 57.83 | 65.92 | 930000000 | 1000000000 | 1100000 | 3351.12 | 24.90 | 7.00 | 6.91 | 4.72 |
| 26 | 2018 | 33.85 | 42.29 | 58.88 | 66.54 | 950000000 | 1000000000 | 1100000 | 3548.59 | 25.99 | 7.00 | 6.91 | 9.30 |
| 26 | 2019 | 34.06 | 42.33 | 59.21 | 66.58 | 940000000 | 1100000000 | 1200000 | 3552.07 | 24.97 | 7.00 | 6.91 | 0.68 |
| 27 | 2000 | 18 | 23.00 | 55.21 | 57.79 | 230000000 | 210000000 | 1100000 | 3522.31 | 51.83 | 0.00 | 5.10 | 25.97 |
| 27 | 2001 | 20.23 | 22.13 | 56.92 | 61.42 | 240000000 | 220000000 | 1200000 | 3278.01 | 48.66 | 1.00 | 5.84 | 6.49 |
| 27 | 2002 | 21 | 24.42 | 56.83 | 60.75 | 320000000 | 200000000 | 1300000 | 3190.62 | 52.16 | 1.00 | 6.33 | 12.33 |
| 27 | 2003 | 20.52 | 24.08 | 55.54 | 61.42 | 460000000 | 240000000 | 1400000 | 4330.94 | 48.83 | 1.00 | 4.95 | 4.55 |
| 27 | 2004 | 18.65 | 22.83 | 52.79 | 61.67 | 580000000 | 280000000 | 1500000 | 5073.54 | 49.61 | 1.00 | 4.69 | 7.50 |
| 27 | 2005 | 17.33 | 21.50 | 53.54 | 59.63 | 560000000 | 280000000 | 1500000 | 5520.12 | 52.92 | 1.00 | 5.12 | 19.60 |
| 27 | 2006 | 17.29 | 22.38 | 53.50 | 58.71 | 540000000 | 280000000 | 1400000 | 5516.04 | 52.25 | 1.00 | 5.83 | 0.46 |
| 27 | 2007 | 15.52 | 22.50 | 51.83 | 56.71 | 550000000 | 280000000 | 1700000 | 5832.73 | 54.52 | 1.00 | 6.14 | 3.15 |
| 27 | 2008 | 16.37 | 23.75 | 51.83 | 57.17 | 510000000 | 230000000 | 2100000 | 5713.54 | 45.67 | 1.00 | 6.83 | 15.94 |
| 27 | 2009 | 20.29 | 24.21 | 52.13 | 64.25 | 660000000 | 230000000 | 1700000 | 5255.77 | 34.80 | 1.00 | 7.16 | 39.93 |
| 27 | 2010 | 24.58 | 25.29 | 52.46 | 71.42 | 440000000 | 300000000 | 2000000 | 6434.82 | 43.64 | 1.00 | 6.79 | 17.80 |
| 27 | 2011 | 23.96 | 27.71 | 56.08 | 64.13 | 470000000 | 320000000 | 1800000 | 7617.33 | 49.95 | 1.00 | 6.84 | 52.20 |
| 27 | 2012 | 23.94 | 29.04 | 57.21 | 61.63 | 520000000 | 330000000 | 1600000 | 7070.38 | 49.25 | 1.00 | 7.64 | 53.84 |
| 27 | 2013 | 21.29 | 27.04 | 55.54 | 60.00 | 480000000 | 260000000 | 1500000 | 7224.97 | 61.52 | 1.00 | 8.40 | 26.41 |
| 27 | 2014 | 21.75 | 27.63 | 55.83 | 60.04 | 530000000 | 280000000 | 2000000 | 7780.64 | 58.47 | 2.00 | 8.98 | 60.96 |
| 27 | 2015 | 20.94 | 26.83 | 56.29 | 58.75 | 530000000 | 260000000 | 1500000 | 6799.88 | 49.82 | 2.00 | 10.13 | 21.63 |
| 27 | 2016 | 22.29 | 27.79 | 55.58 | 61.21 | 510000000 | 240000000 | 1600000 | 7243.85 | 52.56 | 2.00 | 10.90 | 3.40 |
| 27 | 2017 | 21.42 | 28.33 | 54.00 | 60.50 | 540000000 | 230000000 | 1600000 | 7893.21 | 39.67 | 2.00 | 10.35 | 7.80 |
| 27 | 2018 | 20.71 | 28.58 | 53.08 | 59.75 | 580000000 | 230000000 | 1700000 | 8279.60 | 40.34 | 2.00 | 10.20 | 4.58 |
| 27 | 2019 | 21.56 | 30.17 | 52.92 | 60.04 | 570000000 | 230000000 | 1700000 | 7961.33 | 33.59 | 2.00 | 10.05 | 0.60 |
| 28 | 2000 | 37.85 | 36.83 | 69.71 | 69.17 | 23000000 | 30000000 | 126000 | 254.72 | 8.84 | 1.00 | 710.21 | 0.60 |
| 28 | 2001 | 38.17 | 34.63 | 71.08 | 70.63 | 25000000 | 35000000 | 128000 | 266.86 | 9.04 | 1.00 | 732.40 | 6.93 |
| 28 | 2002 | 40.79 | 35.29 | 74.63 | 71.67 | 37000000 | 36000000 | 150000 | 293.28 | 8.87 | 1.00 | 693.71 | 19.59 |
| 28 | 2003 | 42.1 | 37.25 | 75.88 | 71.08 | 38000000 | 50000000 | 163000 | 373.79 | 9.22 | 1.00 | 579.90 | 29.04 |
| 28 | 2004 | 42.25 | 39.13 | 76.38 | 69.00 | 52000000 | 67000000 | 222000 | 417.63 | 10.88 | 1.00 | 527.34 | 1.69 |
| 28 | 2005 | 39.44 | 40.17 | 71.71 | 67.00 | 46000000 | 74000000 | 245000 | 457.75 | 9.84 | 1.00 | 527.26 | 2.39 |
| 28 | 2006 | 36.79 | 38.25 | 66.33 | 69.00 | 55000000 | 84000000 | 264000 | 473.03 | 10.96 | 1.00 | 522.43 | 64.82 |
| 28 | 2007 | 36.6 | 38.13 | 66.08 | 69.00 | 61000000 | 93000000 | 289000 | 534.36 | 10.35 | 1.00 | 478.63 | 8.47 |
| 28 | 2008 | 35.62 | 39.17 | 64.58 | 67.50 | 82000000 | 110000000 | 272000 | 640.81 | 10.15 | 1.00 | 446.00 | 3.78 |
| 28 | 2009 | 41.52 | 39.71 | 72.83 | 70.50 | 99000000 | 110000000 | 269000 | 621.67 | 13.79 | 2.00 | 470.29 | 0.98 |
| 28 | 2010 | 40.75 | 40.42 | 71.92 | 69.17 | 110000000 | 110000000 | 274000 | 647.84 | 20.52 | 2.00 | 494.79 | 25.47 |
| 28 | 2011 | 38.85 | 45.63 | 65.83 | 66.25 | 120000000 | 130000000 | 238000 | 751.17 | 26.20 | 2.00 | 471.25 | 6.20 |
| 28 | 2012 | 38.48 | 47.46 | 63.63 | 65.88 | 130000000 | 150000000 | 237000 | 758.00 | 26.60 | 2.00 | 510.56 | 2.42 |
| 28 | 2013 | 37.56 | 47.13 | 60.75 | 67.25 | 200000000 | 220000000 | 218000 | 787.47 | 27.08 | 2.00 | 493.90 | 4.63 |
| 28 | 2014 | 35.67 | 47.67 | 60.00 | 63.67 | 180000000 | 180000000 | 191000 | 792.85 | 26.93 | 2.00 | 493.76 | 16.04 |
| 28 | 2015 | 38.42 | 47.46 | 64.50 | 64.88 | 150000000 | 160000000 | 163000 | 653.33 | 26.11 | 2.00 | 591.21 | 30.13 |
| 28 | 2016 | 36.15 | 42.75 | 63.54 | 66.00 | 170000000 | 180000000 | 152000 | 687.67 | 25.94 | 2.00 | 592.61 | 6.76 |
| 28 | 2017 | 36.5 | 43.71 | 63.58 | 65.71 | 170000000 | 180000000 | 143000 | 738.27 | 26.32 | 3.00 | 580.66 | 8.86 |
| 28 | 2018 | 35.35 | 42.08 | 63.13 | 65.50 | 180000000 | 190000000 | 144000 | 820.16 | 27.85 | 3.00 | 555.45 | 11.92 |
| 28 | 2019 | 35.52 | 42.83 | 62.79 | 65.42 | 190000000 | 200000000 | 143000 | 786.90 | 25.37 | 3.00 | 530.24 | 0.16 |
| 29 | 2000 | 14.02 | 7.42 | 63.21 | 57.42 | 3700000000 | 4700000000 | 3500000 | 30743.50 | 44.85 | 3.00 | 8.08 | 36.99 |
| 29 | 2001 | 11.19 | 6.75 | 58.71 | 56.92 | 4000000000 | 4900000000 | 3700000 | 30751.70 | 45.55 | 3.00 | 8.32 | 53.20 |
| 29 | 2002 | 12.25 | 9.25 | 58.13 | 57.13 | 4800000000 | 5800000000 | 3400000 | 33228.70 | 45.70 | 3.00 | 7.89 | 0.46 |
| 29 | 2003 | 13.37 | 10.79 | 58.46 | 57.50 | 5300000000 | 6700000000 | 3500000 | 40458.80 | 43.84 | 3.00 | 6.59 | 54.28 |
| 29 | 2004 | 13.58 | 14.13 | 56.21 | 56.83 | 5700000000 | 7300000000 | 4400000 | 46511.60 | 43.93 | 4.00 | 5.99 | 60.00 |
| 29 | 2005 | 13.27 | 13.21 | 57.00 | 56.33 | 5300000000 | 6900000000 | 9600000 | 48799.80 | 47.45 | 4.00 | 6.00 | 1.51 |
| 29 | 2006 | 14.73 | 15.21 | 57.83 | 56.42 | 5600000000 | 7500000000 | 9600000 | 52027.00 | 50.73 | 4.00 | 5.95 | 22.00 |
| 29 | 2007 | 13.62 | 14.50 | 57.21 | 55.54 | 6000000000 | 8800000000 | 9800000 | 58487.10 | 51.48 | 4.00 | 5.44 | 6.16 |
| 29 | 2008 | 14.1 | 14.63 | 57.00 | 56.58 | 6300000000 | 9700000000 | 9600000 | 64322.10 | 54.18 | 4.00 | 5.10 | 3.45 |
| 29 | 2009 | 17.6 | 15.83 | 57.29 | 62.08 | 5600000000 | 9000000000 | 9300000 | 58163.30 | 47.13 | 5.00 | 5.36 | 21.46 |
| 29 | 2010 | 18.1 | 15.50 | 56.63 | 64.08 | 5700000000 | 9100000000 | 9400000 | 58041.40 | 50.52 | 5.00 | 5.62 | 11.14 |
| 29 | 2011 | 16 | 16.63 | 55.63 | 59.75 | 6400000000 | 9800000000 | 8500000 | 61753.60 | 53.82 | 5.00 | 5.37 | 19.07 |
| 29 | 2012 | 18.52 | 20.38 | 55.75 | 60.92 | 6100000000 | 9600000000 | 8400000 | 58507.50 | 54.63 | 5.00 | 5.79 | 9.20 |
| 29 | 2013 | 18.5 | 22.42 | 55.08 | 59.50 | 6500000000 | 10000000000 | 8600000 | 61191.20 | 54.83 | 5.00 | 5.62 | 18.13 |
| 29 | 2014 | 18.94 | 23.33 | 55.71 | 58.83 | 7600000000 | 10000000000 | 10000000 | 62549.00 | 54.61 | 7.00 | 5.61 | 53.86 |
| 29 | 2015 | 18.98 | 21.54 | 58.67 | 57.75 | 6700000000 | 8900000000 | 10000000 | 53254.90 | 55.42 | 9.00 | 6.73 | 83.88 |
| 29 | 2016 | 18.29 | 22.54 | 56.04 | 58.00 | 7500000000 | 9200000000 | 11000000 | 54664.00 | 53.43 | 9.00 | 6.73 | 32.53 |
| 29 | 2017 | 18.52 | 21.42 | 57.38 | 58.25 | 8500000000 | 9800000000 | 12000000 | 57141.10 | 55.14 | 10.00 | 6.60 | 15.47 |
| 29 | 2018 | 16.42 | 19.33 | 57.00 | 56.50 | 9100000000 | 10000000000 | 13000000 | 61390.70 | 55.64 | 11.00 | 6.31 | 3.30 |
| 29 | 2019 | 15.79 | 18.33 | 57.00 | 56.25 | 9700000000 | 11000000000 | 33000000 | 60170.30 | 58.32 | 11.00 | 6.03 | 0.40 |
| 30 | 2000 | 16.27 | 12.42 | 62.13 | 58.00 | 25000000000 | 58000000000 | 19000000 | 23635.90 | 30.85 | 27.00 | 0.66 | 80.93 |
| 30 | 2001 | 16.02 | 11.92 | 60.58 | 59.54 | 24000000000 | 56000000000 | 18000000 | 23607.90 | 31.84 | 28.00 | 0.69 | 64.73 |
| 30 | 2002 | 16.9 | 12.96 | 60.08 | 60.75 | 27000000000 | 60000000000 | 18000000 | 25077.70 | 32.59 | 30.00 | 0.67 | 1.50 |
| 30 | 2003 | 18.15 | 17.13 | 58.79 | 60.38 | 30000000000 | 73000000000 | 18000000 | 30243.60 | 32.81 | 30.00 | 0.61 | 8.87 |
| 30 | 2004 | 18.37 | 18.42 | 59.29 | 59.04 | 36000000000 | 80000000000 | 20000000 | 34044.10 | 35.71 | 32.00 | 0.55 | 12.86 |
| 30 | 2005 | 18.29 | 18.00 | 59.29 | 59.29 | 41000000000 | 85000000000 | 22000000 | 34507.40 | 38.06 | 33.00 | 0.55 | 4.83 |
| 30 | 2006 | 15.69 | 14.46 | 60.29 | 56.63 | 46000000000 | 86000000000 | 24000000 | 36323.40 | 41.43 | 34.00 | 0.54 | 39.15 |
| 30 | 2007 | 15.25 | 13.17 | 60.08 | 57.25 | 49000000000 | 96000000000 | 24000000 | 41587.20 | 43.32 | 35.00 | 0.50 | 5.24 |
| 30 | 2008 | 14.02 | 13.54 | 58.13 | 56.38 | 53000000000 | 1.1E+11 | 25000000 | 45427.20 | 43.80 | 37.00 | 0.54 | 72.39 |
| 30 | 2009 | 18.52 | 14.13 | 59.21 | 63.71 | 47000000000 | 93000000000 | 24000000 | 41485.90 | 38.12 | 38.00 | 0.64 | 3.31 |
| 30 | 2010 | 17.12 | 16.42 | 59.04 | 58.79 | 49000000000 | 91000000000 | 27000000 | 41531.90 | 42.57 | 39.00 | 0.65 | 1.08 |
| 30 | 2011 | 17.46 | 18.58 | 58.04 | 58.29 | 53000000000 | 1E+11 | 28000000 | 46644.80 | 45.06 | 42.00 | 0.62 | 0.20 |
| 30 | 2012 | 16.94 | 16.29 | 58.42 | 59.17 | 52000000000 | 96000000000 | 30000000 | 43858.40 | 46.31 | 43.00 | 0.63 | 9.12 |
| 30 | 2013 | 15.81 | 15.63 | 58.00 | 58.00 | 55000000000 | 1E+11 | 32000000 | 46285.80 | 45.42 | 44.00 | 0.64 | 20.79 |
| 30 | 2014 | 14.6 | 14.92 | 58.00 | 56.29 | 59000000000 | 1E+11 | 33000000 | 47960.00 | 45.62 | 46.00 | 0.61 | 8.41 |
| 30 | 2015 | 16.48 | 15.33 | 61.13 | 56.50 | 51000000000 | 85000000000 | 35000000 | 41139.50 | 46.85 | 47.00 | 0.65 | 10.73 |
| 30 | 2016 | 15.27 | 16.33 | 58.25 | 55.96 | 52000000000 | 87000000000 | 36000000 | 42098.90 | 46.02 | 48.00 | 0.74 | 2.65 |
| 30 | 2017 | 15.42 | 17.00 | 58.21 | 55.63 | 56000000000 | 97000000000 | 37000000 | 44349.60 | 47.40 | 51.00 | 0.78 | 5.40 |
| 30 | 2018 | 15.96 | 17.21 | 59.08 | 55.63 | 59000000000 | 1E+11 | 39000000 | 47639.00 | 47.42 | 53.00 | 0.75 | 72.70 |
| 30 | 2019 | 16.75 | 17.67 | 59.25 | 56.58 | 58000000000 | 1E+11 | 40000000 | 46445.30 | 46.89 | 53.00 | 0.72 | 68.52 |
| 31 | 2000 | 40.44 | 47.42 | 67.71 | 65.75 | 11000000 | 15000000 | 60000 | 302.21 | 28.22 | 0.00 | 710.21 | 25.35 |
| 31 | 2001 | 39 | 44.54 | 66.00 | 67.46 | 14000000 | 20000000 | 57000 | 292.57 | 28.96 | 0.00 | 732.40 | 5.60 |
| 31 | 2002 | 40.06 | 46.00 | 65.63 | 68.50 | 16000000 | 26000000 | 58000 | 326.86 | 30.27 | 0.00 | 693.71 | 0.51 |
| 31 | 2003 | 41.56 | 48.21 | 66.42 | 68.50 | 26000000 | 37000000 | 61000 | 396.03 | 32.80 | 0.00 | 579.90 | 1.81 |
| 31 | 2004 | 40.69 | 47.96 | 66.42 | 67.00 | 25000000 | 38000000 | 83000 | 412.59 | 33.29 | 1.00 | 527.34 | 65.96 |
| 31 | 2005 | 41.02 | 50.21 | 66.33 | 65.50 | 27000000 | 42000000 | 81000 | 406.40 | 35.56 | 1.00 | 527.26 | 1.72 |
| 31 | 2006 | 40.96 | 47.88 | 66.71 | 67.33 | 23000000 | 42000000 | 94000 | 407.70 | 35.37 | 1.00 | 522.43 | 2.98 |
| 31 | 2007 | 40.69 | 47.67 | 66.46 | 67.25 | 38000000 | 59000000 | 86000 | 449.14 | 34.21 | 1.00 | 478.63 | 35.50 |
| 31 | 2008 | 39 | 46.50 | 66.04 | 65.46 | 44000000 | 68000000 | 74000 | 544.15 | 34.31 | 1.00 | 446.00 | 2.10 |
| 31 | 2009 | 41.67 | 46.92 | 66.25 | 70.17 | 73000000 | 94000000 | 150000 | 538.44 | 35.55 | 1.00 | 470.29 | 0.98 |
| 31 | 2010 | 37.67 | 47.13 | 60.96 | 67.25 | 110000000 | 89000000 | 202000 | 534.04 | 37.84 | 1.00 | 494.79 | 20.70 |
| 31 | 2011 | 37.73 | 48.75 | 59.21 | 67.50 | 210000000 | 81000000 | 300000 | 587.10 | 43.64 | 1.00 | 471.25 | 0.85 |
| 31 | 2012 | 38.48 | 49.75 | 60.13 | 67.08 | 210000000 | 46000000 | 235000 | 571.81 | 45.21 | 1.00 | 510.56 | 22.93 |
| 31 | 2013 | 38.96 | 51.33 | 60.00 | 66.58 | 230000000 | 58000000 | 327000 | 621.40 | 46.48 | 1.00 | 493.90 | 30.91 |
| 31 | 2014 | 38.87 | 50.25 | 60.50 | 67.00 | 230000000 | 60000000 | 282000 | 640.93 | 39.72 | 1.00 | 493.76 | 3.02 |
| 31 | 2015 | 38.42 | 47.58 | 63.08 | 66.17 | 210000000 | 53000000 | 273000 | 570.91 | 35.85 | 1.00 | 591.21 | 38.58 |
| 31 | 2016 | 38.19 | 47.17 | 62.04 | 67.17 | 220000000 | 55000000 | 338000 | 597.47 | 35.18 | 1.00 | 592.61 | 9.00 |
| 31 | 2017 | 38.33 | 47.58 | 61.96 | 67.13 | 250000000 | 57000000 | 514000 | 626.09 | 33.11 | 1.00 | 580.66 | 31.23 |
| 31 | 2018 | 38.87 | 47.83 | 62.75 | 67.17 | 270000000 | 65000000 | 573000 | 678.96 | 31.32 | 1.00 | 555.45 | 3.53 |
| 31 | 2019 | 38.12 | 47.00 | 62.75 | 66.50 | 290000000 | 73000000 | 876000 | 679.29 | 30.06 | 1.00 | 530.24 | 65.88 |
| 32 | 2000 | 26.87 | 29.08 | 62.17 | 62.50 | 2900000000 | 440000000 | 3000000 | 2869.11 | 35.50 | 1.00 | 16.18 | 15.00 |
| 32 | 2001 | 27.85 | 29.46 | 62.71 | 63.54 | 2800000000 | 430000000 | 2900000 | 2977.15 | 31.89 | 1.00 | 16.69 | 0.53 |
| 32 | 2002 | 30.44 | 33.50 | 63.79 | 63.58 | 2700000000 | 430000000 | 2800000 | 3136.16 | 30.72 | 1.00 | 17.59 | 34.62 |
| 32 | 2003 | 35.56 | 35.79 | 68.13 | 67.21 | 3100000000 | 410000000 | 3300000 | 2445.44 | 42.15 | 1.00 | 29.37 | 0.35 |
| 32 | 2004 | 38.19 | 40.04 | 67.46 | 68.88 | 3200000000 | 450000000 | 3500000 | 2528.58 | 41.15 | 1.00 | 41.93 | 42.25 |
| 32 | 2005 | 32.54 | 36.25 | 64.46 | 64.38 | 3500000000 | 510000000 | 3700000 | 3970.32 | 28.33 | 1.00 | 30.28 | 0.43 |
| 32 | 2006 | 29.33 | 34.08 | 62.75 | 61.83 | 3900000000 | 500000000 | 4000000 | 4134.68 | 28.22 | 1.00 | 33.30 | 64.76 |
| 32 | 2007 | 29.12 | 33.25 | 61.50 | 63.50 | 4100000000 | 530000000 | 4000000 | 4707.79 | 26.76 | 1.00 | 33.17 | 6.60 |
| 32 | 2008 | 29.25 | 33.29 | 61.71 | 63.50 | 4200000000 | 530000000 | 4000000 | 5087.99 | 23.91 | 1.00 | 34.53 | 0.20 |
| 32 | 2009 | 29.58 | 33.38 | 60.25 | 65.54 | 4000000000 | 520000000 | 4000000 | 5039.40 | 21.03 | 1.00 | 35.97 | 14.10 |
| 32 | 2010 | 29.15 | 33.50 | 60.04 | 64.75 | 4200000000 | 660000000 | 4100000 | 5555.39 | 22.68 | 1.00 | 36.82 | 0.02 |
| 32 | 2011 | 31.77 | 34.25 | 62.79 | 66.50 | 4400000000 | 690000000 | 4300000 | 5913.43 | 24.19 | 1.00 | 38.09 | 10.63 |
| 32 | 2012 | 32.1 | 35.25 | 62.67 | 66.29 | 4700000000 | 690000000 | 4600000 | 6110.37 | 24.58 | 1.00 | 39.32 | 11.61 |
| 32 | 2013 | 32.04 | 35.08 | 62.75 | 66.25 | 5100000000 | 680000000 | 4700000 | 6238.13 | 25.17 | 1.00 | 41.79 | 0.68 |
| 32 | 2014 | 29.12 | 32.46 | 61.63 | 64.17 | 5600000000 | 720000000 | 5100000 | 6608.83 | 25.15 | 1.00 | 43.55 | 14.10 |
| 32 | 2015 | 27.65 | 31.42 | 61.33 | 62.54 | 6100000000 | 800000000 | 5600000 | 6921.52 | 23.76 | 1.00 | 45.05 | 3.80 |
| 32 | 2016 | 26.31 | 30.79 | 60.67 | 61.17 | 6700000000 | 880000000 | 6000000 | 7280.88 | 23.91 | 1.00 | 46.06 | 8.43 |
| 32 | 2017 | 26.73 | 30.92 | 60.17 | 62.38 | 7200000000 | 940000000 | 6200000 | 7609.34 | 23.68 | 1.00 | 47.53 | 2.32 |
| 32 | 2018 | 28.46 | 32.96 | 60.79 | 63.17 | 7600000000 | 960000000 | 6600000 | 8050.63 | 23.55 | 1.00 | 49.51 | 0.19 |
| 32 | 2019 | 27.12 | 33.50 | 59.25 | 61.50 | 7900000000 | 1100000000 | 7600000 | 8282.12 | 23.07 | 1.00 | 51.49 | 1.29 |
| 33 | 2000 | 40.4 | 47.67 | 67.13 | 66.00 | 4200000000 | 8200000000 | 21000000 | 1771.59 | 44.06 | 16.00 | 28.13 | 77.69 |
| 33 | 2001 | 31.85 | 41.63 | 60.21 | 61.88 | 4700000000 | 9800000000 | 22000000 | 2100.35 | 36.89 | 18.00 | 29.17 | 31.78 |
| 33 | 2002 | 30.31 | 37.00 | 61.46 | 62.17 | 5300000000 | 11000000000 | 23000000 | 2377.53 | 35.21 | 18.00 | 31.35 | 62.39 |
| 33 | 2003 | 27.04 | 34.54 | 57.54 | 62.00 | 5900000000 | 13000000000 | 23000000 | 2975.13 | 35.25 | 20.00 | 30.69 | 83.89 |
| 33 | 2004 | 23.85 | 31.67 | 56.96 | 59.08 | 7300000000 | 16000000000 | 22000000 | 4102.36 | 34.42 | 22.00 | 28.81 | 67.80 |
| 33 | 2005 | 22.58 | 32.92 | 55.29 | 56.96 | 7800000000 | 18000000000 | 22000000 | 5323.46 | 35.20 | 24.00 | 28.28 | 3.20 |
| 33 | 2006 | 20.87 | 33.17 | 53.38 | 55.21 | 9700000000 | 19000000000 | 22000000 | 6920.19 | 33.73 | 24.00 | 27.19 | 23.50 |
| 33 | 2007 | 21.87 | 34.00 | 54.63 | 55.13 | 12000000000 | 22000000000 | 23000000 | 9101.25 | 30.16 | 24.00 | 25.58 | 0.20 |
| 33 | 2008 | 23.67 | 34.25 | 55.54 | 57.54 | 16000000000 | 26000000000 | 24000000 | 11635.30 | 31.31 | 24.00 | 24.85 | 4.39 |
| 33 | 2009 | 34.02 | 36.79 | 58.79 | 72.46 | 12000000000 | 24000000000 | 21000000 | 8562.81 | 27.94 | 24.00 | 31.74 | 33.83 |
| 33 | 2010 | 27.42 | 36.13 | 55.50 | 63.21 | 13000000000 | 30000000000 | 22000000 | 10675.00 | 29.22 | 25.00 | 30.37 | 10.23 |
| 33 | 2011 | 26.56 | 38.04 | 54.46 | 60.63 | 17000000000 | 37000000000 | 25000000 | 14311.10 | 28.06 | 25.00 | 29.38 | 1.45 |
| 33 | 2012 | 27.62 | 40.50 | 54.88 | 59.88 | 18000000000 | 48000000000 | 28000000 | 15420.90 | 26.91 | 26.00 | 30.84 | 52.89 |
| 33 | 2013 | 28.5 | 41.96 | 54.92 | 60.13 | 20000000000 | 60000000000 | 31000000 | 15974.60 | 25.85 | 26.00 | 31.84 | 40.81 |
| 33 | 2014 | 32.27 | 43.75 | 58.21 | 62.58 | 19000000000 | 55000000000 | 32000000 | 14095.60 | 27.11 | 27.00 | 38.38 | 0.79 |
| 33 | 2015 | 38.15 | 44.25 | 62.38 | 69.67 | 13000000000 | 38000000000 | 34000000 | 9313.01 | 28.70 | 27.00 | 60.94 | 6.08 |
| 33 | 2016 | 34.52 | 43.50 | 58.29 | 67.25 | 13000000000 | 28000000000 | 25000000 | 8704.90 | 25.85 | 27.00 | 67.06 | 10.68 |
| 33 | 2017 | 30.96 | 42.88 | 55.79 | 63.25 | 15000000000 | 36000000000 | 24000000 | 10720.30 | 26.09 | 29.00 | 58.34 | 0.88 |
| 33 | 2018 | 29.25 | 41.38 | 56.13 | 61.00 | 19000000000 | 39000000000 | 25000000 | 11370.80 | 30.52 | 30.00 | 62.67 | 27.02 |
| 33 | 2019 | 27.98 | 41.83 | 54.46 | 59.67 | 18000000000 | 41000000000 | 24000000 | 11585.00 | 28.31 | 30.00 | 66.99 | 8.53 |
| 34 | 2000 | 50.15 | 44.58 | 79.25 | 76.46 | 450000000 | 420000000 | 627000 | 1445.28 | 32.13 | 4.00 | 1.00 | 14.58 |
| 34 | 2001 | 39.6 | 41.63 | 68.38 | 69.21 | 440000000 | 470000000 | 641000 | 1894.62 | 23.22 | 5.00 | 1.00 | 5.10 |
| 34 | 2002 | 39.81 | 43.50 | 68.50 | 67.63 | 450000000 | 510000000 | 683000 | 2172.10 | 21.49 | 5.00 | 1.00 | 8.40 |
| 34 | 2003 | 38.04 | 42.71 | 66.67 | 66.71 | 410000000 | 500000000 | 761000 | 2425.85 | 22.60 | 5.00 | 1.00 | 29.72 |
| 34 | 2004 | 35.69 | 42.67 | 65.75 | 62.96 | 460000000 | 580000000 | 819000 | 2691.28 | 24.55 | 5.00 | 1.00 | 0.72 |
| 34 | 2005 | 34.31 | 42.42 | 64.38 | 61.83 | 490000000 | 640000000 | 860000 | 3002.14 | 27.62 | 5.00 | 1.00 | 3.49 |
| 34 | 2006 | 36.17 | 46.04 | 63.92 | 62.38 | 490000000 | 710000000 | 841000 | 3328.88 | 30.33 | 5.00 | 1.00 | 17.06 |
| 34 | 2007 | 34.48 | 44.88 | 63.50 | 60.58 | 630000000 | 730000000 | 937000 | 3567.84 | 31.93 | 5.00 | 1.00 | 44.01 |
| 34 | 2008 | 34.83 | 45.96 | 62.67 | 61.04 | 750000000 | 790000000 | 1000000 | 4249.02 | 34.16 | 5.00 | 1.00 | 21.42 |
| 34 | 2009 | 38.71 | 46.13 | 60.96 | 70.33 | 670000000 | 810000000 | 968000 | 4231.62 | 25.25 | 5.00 | 1.00 | 56.40 |
| 34 | 2010 | 36.1 | 47.04 | 59.00 | 66.17 | 790000000 | 860000000 | 1000000 | 4633.59 | 27.90 | 5.00 | 1.00 | 62.00 |
| 34 | 2011 | 36.27 | 48.04 | 59.50 | 65.00 | 850000000 | 920000000 | 1100000 | 5200.56 | 31.12 | 5.00 | 1.00 | 30.30 |
| 34 | 2012 | 34.69 | 45.13 | 59.75 | 64.50 | 1000000000 | 940000000 | 1300000 | 5682.04 | 30.16 | 5.00 | 1.00 | 61.85 |
| 34 | 2013 | 32.96 | 42.46 | 59.75 | 63.71 | 1300000000 | 990000000 | 1400000 | 6056.33 | 28.64 | 5.00 | 1.00 | 27.74 |
| 34 | 2014 | 32.5 | 41.29 | 60.21 | 63.50 | 1500000000 | 990000000 | 1700000 | 6377.09 | 28.05 | 6.00 | 1.00 | 4.32 |
| 34 | 2015 | 37.1 | 44.00 | 63.96 | 66.25 | 1600000000 | 990000000 | 1700000 | 6124.49 | 21.26 | 6.00 | 1.00 | 10.00 |
| 34 | 2016 | 39.21 | 46.13 | 63.54 | 68.75 | 1700000000 | 1500000000 | 1600000 | 6060.09 | 19.50 | 6.00 | 1.00 | 4.90 |
| 34 | 2017 | 36.67 | 44.63 | 62.75 | 65.96 | 2000000000 | 1500000000 | 1800000 | 6213.50 | 20.83 | 6.00 | 1.00 | 0.88 |
| 34 | 2018 | 35.83 | 43.96 | 62.79 | 64.92 | 2300000000 | 1500000000 | 2500000 | 6295.94 | 22.60 | 6.00 | 1.00 | 0.90 |
| 34 | 2019 | 34.23 | 40.38 | 63.54 | 64.54 | 2300000000 | 1700000000 | 2100000 | 6183.82 | 23.39 | 6.00 | 1.00 | 7.49 |
| 35 | 2000 | 19.75 | 20.25 | 62.75 | 56.50 | 39000000000 | 29000000000 | 77000000 | 22364.00 | 28.60 | 29.00 | 0.66 | 22.77 |
| 35 | 2001 | 18.95 | 19.96 | 60.79 | 57.17 | 38000000000 | 29000000000 | 75000000 | 22433.60 | 28.27 | 30.00 | 0.69 | 33.24 |
| 35 | 2002 | 19.04 | 19.58 | 60.46 | 58.04 | 41000000000 | 30000000000 | 77000000 | 24177.30 | 27.53 | 30.00 | 0.67 | 1.98 |
| 35 | 2003 | 20.54 | 20.92 | 60.54 | 59.63 | 47000000000 | 36000000000 | 75000000 | 29568.40 | 26.11 | 30.00 | 0.61 | 2.60 |
| 35 | 2004 | 22.33 | 23.75 | 60.83 | 60.08 | 53000000000 | 36000000000 | 74000000 | 33741.30 | 26.47 | 30.00 | 0.55 | 65.61 |
| 35 | 2005 | 22.29 | 22.88 | 61.42 | 60.29 | 52000000000 | 39000000000 | 75000000 | 34760.20 | 27.03 | 32.00 | 0.55 | 11.66 |
| 35 | 2006 | 23.69 | 24.42 | 62.13 | 60.83 | 55000000000 | 39000000000 | 78000000 | 36443.60 | 27.94 | 32.00 | 0.54 | 7.64 |
| 35 | 2007 | 21.58 | 21.25 | 61.17 | 60.75 | 64000000000 | 46000000000 | 81000000 | 41508.40 | 27.85 | 33.00 | 0.50 | 2.01 |
| 35 | 2008 | 21.79 | 21.08 | 61.00 | 61.50 | 68000000000 | 50000000000 | 79000000 | 45334.10 | 28.12 | 35.00 | 0.54 | 28.18 |
| 35 | 2009 | 24.96 | 21.25 | 61.46 | 67.21 | 59000000000 | 45000000000 | 77000000 | 41575.40 | 24.84 | 36.00 | 0.64 | 7.30 |
| 35 | 2010 | 25.44 | 22.00 | 62.04 | 66.83 | 56000000000 | 47000000000 | 77000000 | 40638.30 | 26.79 | 38.00 | 0.65 | 6.04 |
| 35 | 2011 | 25.92 | 24.46 | 63.63 | 63.75 | 66000000000 | 56000000000 | 80000000 | 43790.70 | 28.42 | 40.00 | 0.62 | 5.84 |
| 35 | 2012 | 28.02 | 27.29 | 64.46 | 64.29 | 64000000000 | 50000000000 | 82000000 | 40874.70 | 29.20 | 41.00 | 0.63 | 17.72 |
| 35 | 2013 | 28.81 | 30.08 | 64.00 | 63.54 | 66000000000 | 52000000000 | 84000000 | 42592.90 | 29.36 | 41.00 | 0.64 | 40.00 |
| 35 | 2014 | 29.17 | 31.71 | 63.92 | 62.71 | 67000000000 | 58000000000 | 84000000 | 43011.30 | 29.67 | 42.00 | 0.61 | 6.68 |
| 35 | 2015 | 28.54 | 29.00 | 66.17 | 61.92 | 66000000000 | 48000000000 | 84000000 | 36638.20 | 30.59 | 44.00 | 0.65 | 8.03 |
| 35 | 2016 | 27.27 | 29.33 | 63.92 | 61.29 | 64000000000 | 49000000000 | 83000000 | 37037.40 | 30.25 | 45.00 | 0.74 | 12.75 |
| 35 | 2017 | 26.19 | 26.67 | 64.29 | 61.42 | 68000000000 | 54000000000 | 87000000 | 38812.20 | 30.95 | 47.00 | 0.78 | 0.22 |
| 35 | 2018 | 25.69 | 26.75 | 64.42 | 60.21 | 73000000000 | 58000000000 | 89000000 | 41631.10 | 31.72 | 48.00 | 0.75 | 41.61 |
| 35 | 2019 | 25.79 | 25.50 | 64.75 | 61.33 | 71000000000 | 61000000000 | 92000000 | 40493.90 | 31.77 | 48.00 | 0.72 | 61.45 |
| 36 | 2000 | 31.62 | 37.08 | 64.17 | 62.00 | 2300000000 | 1800000000 | 2000000 | 1072.81 | 43.34 | 5.00 | 44.19 | 1.14 |
| 36 | 2001 | 31.21 | 34.21 | 64.33 | 63.88 | 2000000000 | 1900000000 | 1800000 | 990.56 | 41.16 | 5.00 | 50.99 | 58.00 |
| 36 | 2002 | 29.04 | 32.54 | 62.42 | 63.13 | 2000000000 | 1900000000 | 1900000 | 1036.16 | 40.01 | 5.00 | 51.60 | 63.00 |
| 36 | 2003 | 29.81 | 32.79 | 63.21 | 63.63 | 1800000000 | 1600000000 | 1900000 | 1048.01 | 42.31 | 5.00 | 54.20 | 1.72 |
| 36 | 2004 | 30.62 | 34.46 | 63.58 | 63.21 | 2400000000 | 1500000000 | 2300000 | 1121.49 | 41.83 | 5.00 | 56.04 | 25.00 |
| 36 | 2005 | 31.27 | 35.92 | 63.29 | 63.33 | 2900000000 | 3300000000 | 2600000 | 1244.35 | 41.23 | 5.00 | 55.09 | 6.33 |
| 36 | 2006 | 30.94 | 38.50 | 61.29 | 62.08 | 4100000000 | 3100000000 | 2800000 | 1452.44 | 41.25 | 5.00 | 51.31 | 7.91 |
| 36 | 2007 | 30.4 | 38.58 | 61.67 | 60.54 | 5500000000 | 3400000000 | 3100000 | 1744.64 | 38.01 | 5.00 | 46.15 | 21.30 |
| 36 | 2008 | 30.85 | 38.79 | 61.96 | 60.96 | 3300000000 | 4300000000 | 3100000 | 1991.23 | 33.36 | 5.00 | 44.32 | 11.48 |
| 36 | 2009 | 32.04 | 38.75 | 60.13 | 65.21 | 2900000000 | 4100000000 | 3000000 | 1905.89 | 30.22 | 6.00 | 47.68 | 21.02 |
| 36 | 2010 | 29.71 | 38.54 | 57.88 | 63.00 | 3400000000 | 6000000000 | 3500000 | 2217.47 | 32.87 | 6.00 | 45.11 | 12.70 |
| 36 | 2011 | 28.6 | 37.83 | 57.04 | 62.33 | 4100000000 | 6100000000 | 3900000 | 2450.73 | 29.11 | 6.00 | 43.31 | 19.97 |
| 36 | 2012 | 28.31 | 38.21 | 56.29 | 62.13 | 5000000000 | 7100000000 | 4300000 | 2694.31 | 27.47 | 6.00 | 42.23 | 55.82 |
| 36 | 2013 | 26.69 | 37.58 | 55.46 | 60.33 | 5600000000 | 8400000000 | 4700000 | 2871.43 | 26.18 | 6.00 | 42.45 | 87.00 |
| 36 | 2014 | 27.58 | 39.17 | 55.42 | 60.58 | 6100000000 | 11000000000 | 4800000 | 2959.65 | 27.35 | 7.00 | 44.40 | 38.81 |
| 36 | 2015 | 27.29 | 39.71 | 54.38 | 60.50 | 6400000000 | 12000000000 | 5400000 | 3001.04 | 27.21 | 7.00 | 45.50 | 21.33 |
| 36 | 2016 | 25.71 | 36.21 | 54.96 | 60.25 | 6300000000 | 12000000000 | 6000000 | 3073.65 | 26.67 | 7.00 | 47.49 | 3.26 |
| 36 | 2017 | 27.85 | 38.38 | 56.00 | 61.33 | 8300000000 | 12000000000 | 6600000 | 3123.23 | 29.55 | 7.00 | 50.40 | 0.47 |
| 36 | 2018 | 29.15 | 39.50 | 56.21 | 62.58 | 9700000000 | 12000000000 | 7200000 | 3252.09 | 30.21 | 7.00 | 52.66 | 82.74 |
| 36 | 2019 | 28.33 | 37.13 | 56.25 | 63.29 | 11000000000 | 13000000000 | 8300000 | 3485.08 | 28.34 | 7.00 | 54.92 | 68.71 |
| 37 | 2000 | 11.92 | 6.75 | 64.29 | 52.79 | 2000000000 | 2300000000 | 2000000 | 24285.50 | 42.03 | 6.00 | 0.66 | 1.80 |
| 37 | 2001 | 10.6 | 5.67 | 61.96 | 53.58 | 2100000000 | 2400000000 | 2000000 | 24946.20 | 39.76 | 6.00 | 0.69 | 11.48 |
| 37 | 2002 | 11.04 | 5.38 | 61.63 | 55.08 | 2200000000 | 2400000000 | 2000000 | 26869.70 | 39.14 | 6.00 | 0.67 | 15.23 |
| 37 | 2003 | 12.02 | 6.54 | 62.67 | 54.83 | 2700000000 | 3000000000 | 2000000 | 32855.10 | 37.32 | 6.00 | 0.61 | 5.99 |
| 37 | 2004 | 12.69 | 6.50 | 63.04 | 55.83 | 3000000000 | 3400000000 | 2100000 | 37702.80 | 38.62 | 6.00 | 0.55 | 42.87 |
| 37 | 2005 | 12.4 | 6.50 | 62.79 | 55.50 | 3100000000 | 3600000000 | 2100000 | 39040.30 | 40.27 | 7.00 | 0.55 | 5.40 |
| 37 | 2006 | 12.5 | 6.54 | 63.29 | 55.17 | 3500000000 | 4100000000 | 2300000 | 41188.10 | 43.07 | 8.00 | 0.54 | 74.48 |
| 37 | 2007 | 11.83 | 6.33 | 63.25 | 54.08 | 4300000000 | 4800000000 | 2500000 | 48414.80 | 43.83 | 8.00 | 0.50 | 3.80 |
| 37 | 2008 | 12.56 | 7.38 | 63.21 | 54.54 | 4900000000 | 5600000000 | 2500000 | 53554.00 | 44.90 | 8.00 | 0.54 | 1.46 |
| 37 | 2009 | 17.37 | 7.63 | 64.38 | 62.75 | 4100000000 | 5200000000 | 2200000 | 47294.00 | 36.08 | 8.00 | 0.64 | 0.24 |
| 37 | 2010 | 18.12 | 7.92 | 65.08 | 63.25 | 4500000000 | 5300000000 | 2300000 | 46460.00 | 38.41 | 8.00 | 0.65 | 11.01 |
| 37 | 2011 | 16.37 | 9.13 | 64.67 | 58.96 | 5600000000 | 6000000000 | 2600000 | 51082.00 | 38.90 | 8.00 | 0.62 | 22.07 |
| 37 | 2012 | 19.6 | 11.04 | 66.04 | 62.13 | 5400000000 | 5800000000 | 2800000 | 47710.80 | 38.81 | 8.00 | 0.63 | 9.74 |
| 37 | 2013 | 20.02 | 12.71 | 65.21 | 62.13 | 5800000000 | 6400000000 | 2800000 | 49878.00 | 38.02 | 8.00 | 0.64 | 11.15 |
| 37 | 2014 | 20.67 | 13.38 | 65.71 | 62.25 | 5400000000 | 6300000000 | 2700000 | 50260.30 | 36.48 | 8.00 | 0.61 | 2.96 |
| 37 | 2015 | 20 | 12.63 | 66.88 | 60.50 | 4000000000 | 5800000000 | 2600000 | 42784.70 | 35.41 | 8.00 | 0.65 | 6.38 |
| 37 | 2016 | 18.46 | 13.17 | 63.46 | 60.29 | 4000000000 | 6200000000 | 2800000 | 43784.30 | 34.81 | 8.00 | 0.74 | 73.50 |
| 37 | 2017 | 18.06 | 12.75 | 63.79 | 59.58 | 5200000000 | 6700000000 | 3200000 | 46316.70 | 37.68 | 8.00 | 0.78 | 81.00 |
| 37 | 2018 | 17.48 | 13.21 | 63.63 | 58.13 | 5800000000 | 7200000000 | 3200000 | 50021.30 | 38.60 | 8.00 | 0.75 | 27.10 |
| 37 | 2019 | 18.48 | 13.58 | 64.13 | 59.25 | 5900000000 | 6900000000 | 3300000 | 48782.80 | 40.18 | 8.00 | 0.72 | 6.50 |
| 38 | 2000 | 31.9 | 31.33 | 66.08 | 66.38 | 79000000 | 14000000 | 79000 | 594.15 | 25.80 | 0.00 | 12.79 | 0.54 |
| 38 | 2001 | 32.85 | 30.67 | 68.67 | 66.38 | 72000000 | 12000000 | 57000 | 505.42 | 21.82 | 0.00 | 15.69 | 1.90 |
| 38 | 2002 | 32.5 | 31.21 | 68.42 | 65.38 | 65000000 | 10000000 | 81000 | 411.77 | 27.16 | 0.00 | 19.92 | 71.66 |
| 38 | 2003 | 33.21 | 30.83 | 71.08 | 64.50 | 58000000 | 8000000 | 89000 | 335.91 | 31.09 | 1.00 | 28.53 | 1.83 |
| 38 | 2004 | 31 | 29.79 | 67.71 | 64.50 | 51000000 | 6000000 | 90000 | 642.76 | 20.59 | 1.00 | 30.03 | 4.89 |
| 38 | 2005 | 32.04 | 31.58 | 66.50 | 66.00 | 59000000 | 7000000 | 108000 | 665.72 | 19.90 | 1.00 | 28.58 | 35.27 |
| 38 | 2006 | 32.62 | 32.75 | 66.50 | 66.00 | 69000000 | 8000000 | 125000 | 662.36 | 21.03 | 2.00 | 28.07 | 2.40 |
| 38 | 2007 | 32.12 | 34.25 | 62.63 | 67.38 | 87000000 | 9000000 | 143000 | 780.38 | 18.03 | 2.00 | 24.87 | 1.40 |
| 38 | 2008 | 31.33 | 34.13 | 63.96 | 64.58 | 80000000 | 10000000 | 147000 | 924.51 | 14.48 | 2.00 | 22.19 | 24.73 |
| 38 | 2009 | 36.46 | 34.54 | 72.67 | 65.71 | 64000000 | 11000000 | 142000 | 833.28 | 15.75 | 2.00 | 26.64 | 1.04 |
| 38 | 2010 | 41.73 | 36.08 | 78.08 | 69.29 | 80000000 | 12000000 | 91000 | 860.64 | 14.67 | 2.00 | 28.01 | 25.93 |
| 38 | 2011 | 42.37 | 38.58 | 77.67 | 68.50 | 86000000 | 13000000 | 106000 | 762.76 | 16.86 | 2.00 | 29.46 | 33.14 |
| 38 | 2012 | 41.29 | 37.96 | 73.00 | 71.63 | 100000000 | 14000000 | 157000 | 742.78 | 19.84 | 2.00 | 32.08 | 3.10 |
| 38 | 2013 | 38 | 39.58 | 65.08 | 71.33 | 78000000 | 10000000 | 171000 | 700.52 | 19.18 | 2.00 | 35.96 | 46.00 |
| 38 | 2014 | 37.58 | 41.00 | 66.50 | 67.67 | 110000000 | 55000000 | 156000 | 607.43 | 19.82 | 2.00 | 41.73 | 10.14 |
| 38 | 2015 | 37.52 | 41.21 | 65.58 | 68.25 | 120000000 | 57000000 | 449000 | 649.51 | 16.27 | 2.00 | 42.51 | 36.22 |
| 38 | 2016 | 38.52 | 41.00 | 66.63 | 69.42 | 120000000 | 76000000 | 450000 | 684.00 | 16.44 | 2.00 | 43.37 | 3.92 |
| 38 | 2017 | 40.83 | 38.71 | 68.96 | 74.00 | 120000000 | 46000000 | 522000 | 676.66 | 22.49 | 2.00 | 46.61 | 70.00 |
| 38 | 2018 | 37.85 | 38.58 | 66.33 | 70.79 | 170000000 | 10000000 | 552000 | 712.51 | 21.85 | 2.00 | 48.15 | 21.50 |
| 38 | 2019 | 36.19 | 35.96 | 65.92 | 70.50 | 220000000 | 28000000 | 619000 | 777.81 | 20.64 | 2.00 | 49.69 | 0.57 |
| 39 | 2000 | 49.96 | 52.25 | 79.96 | 67.71 | 12000000 | 59000000 | 7750 | 1029.58 | 80.30 | 5.00 | 710.21 | 41.24 |
| 39 | 2001 | 40.48 | 46.46 | 70.88 | 63.63 | 23000000 | 82000000 | 8500 | 868.34 | 77.42 | 5.00 | 732.40 | 0.38 |
| 39 | 2002 | 39.83 | 45.08 | 70.00 | 64.58 | 26000000 | 85000000 | 10000 | 912.28 | 81.52 | 5.00 | 693.71 | 48.63 |
| 39 | 2003 | 42.48 | 44.17 | 71.38 | 69.42 | 30000000 | 120000000 | 13000 | 1026.11 | 80.81 | 5.00 | 579.90 | 0.51 |
| 39 | 2004 | 36.58 | 43.96 | 69.00 | 60.21 | 23000000 | 180000000 | 24000 | 1324.22 | 80.53 | 5.00 | 527.34 | 67.97 |
| 39 | 2005 | 35.29 | 43.83 | 67.33 | 59.42 | 20000000 | 170000000 | 35000 | 1680.20 | 84.16 | 5.00 | 527.26 | 7.40 |
| 39 | 2006 | 33.79 | 44.42 | 65.42 | 57.75 | 26000000 | 160000000 | 46000 | 2064.34 | 84.16 | 5.00 | 522.43 | 0.24 |
| 39 | 2007 | 32.04 | 44.17 | 63.04 | 56.88 | 31000000 | 160000000 | 55000 | 2165.75 | 78.53 | 5.00 | 478.63 | 17.10 |
| 39 | 2008 | 31.81 | 44.00 | 62.46 | 57.17 | 37000000 | 220000000 | 62000 | 2956.26 | 75.15 | 5.00 | 446.00 | 0.07 |
| 39 | 2009 | 29.83 | 44.17 | 60.00 | 55.50 | 85000000 | 550000000 | 94000 | 2314.27 | 70.42 | 5.00 | 470.29 | 14.63 |
| 39 | 2010 | 28.65 | 44.50 | 55.79 | 57.00 | 39000000 | 310000000 | 194000 | 2812.44 | 85.12 | 5.00 | 494.79 | 15.08 |
| 39 | 2011 | 28.6 | 45.75 | 53.71 | 57.75 | 47000000 | 330000000 | 218000 | 3286.69 | 87.28 | 5.00 | 471.25 | 0.85 |
| 39 | 2012 | 31.4 | 47.42 | 56.58 | 58.79 | 68000000 | 310000000 | 256000 | 3032.49 | 83.78 | 5.00 | 510.56 | 17.21 |
| 39 | 2013 | 30.58 | 46.00 | 56.13 | 59.04 | 52000000 | 360000000 | 343000 | 3047.93 | 76.53 | 5.00 | 493.90 | 4.01 |
| 39 | 2014 | 29.42 | 46.00 | 55.00 | 57.83 | 57000000 | 440000000 | 226000 | 2996.92 | 72.99 | 5.00 | 493.76 | 7.49 |
| 39 | 2015 | 32.71 | 43.63 | 59.54 | 62.25 | 47000000 | 560000000 | 220000 | 1762.03 | 69.28 | 5.00 | 591.21 | 2.57 |
| 39 | 2016 | 36.71 | 44.71 | 59.29 | 69.42 | 43000000 | 460000000 | 211000 | 1815.30 | 66.47 | 5.00 | 592.61 | 0.22 |
| 39 | 2017 | 39.81 | 47.71 | 63.17 | 68.75 | 39000000 | 360000000 | 149000 | 1767.89 | 90.78 | 5.00 | 580.66 | 3.55 |
| 39 | 2018 | 38.81 | 48.38 | 65.38 | 63.88 | 35000000 | 260000000 | 156000 | 2223.85 | 97.82 | 5.00 | 555.45 | 81.99 |
| 39 | 2019 | 34.17 | 46.83 | 63.25 | 58.25 | 31000000 | 160000000 | 163000 | 2279.97 | 69.09 | 5.00 | 530.24 | 34.99 |
| 40 | 2000 | 55.31 | 68.92 | 69.92 | 71.79 | 3700000 | 61000000 | 103000 | 405.22 | 11.45 | 5.00 | 21.82 | 66.92 |
| 40 | 2001 | 50.91 | 62.13 | 67.67 | 72.04 | 3600000 | 65000000 | 55000 | 153.59 | 11.99 | 5.00 | 206.62 | 84.83 |
| 40 | 2002 | 55.92 | 60.67 | 72.92 | 78.25 | 3500000 | 68000000 | 28000 | 175.01 | 15.67 | 5.00 | 346.49 | 70.10 |
| 40 | 2003 | 53.37 | 60.42 | 76.00 | 70.33 | 3400000 | 61000000 | 35000 | 173.80 | 24.00 | 5.00 | 405.18 | 4.20 |
| 40 | 2004 | 50.29 | 61.63 | 71.54 | 67.42 | 3300000 | 77000000 | 36000 | 194.04 | 22.73 | 5.00 | 399.48 | 26.30 |
| 40 | 2005 | 52.94 | 64.67 | 73.92 | 67.29 | 3200000 | 54000000 | 61000 | 218.39 | 22.94 | 5.00 | 473.91 | 0.22 |
| 40 | 2006 | 52.31 | 63.54 | 72.29 | 68.79 | 3100000 | 93000000 | 55000 | 255.43 | 21.70 | 5.00 | 468.28 | 4.79 |
| 40 | 2007 | 50.92 | 62.17 | 71.67 | 68.00 | 700000 | 110000000 | 47000 | 286.33 | 39.07 | 5.00 | 516.75 | 32.81 |
| 40 | 2008 | 48.23 | 61.58 | 66.96 | 67.92 | 700000 | 130000000 | 50000 | 327.56 | 39.03 | 5.00 | 559.29 | 12.71 |
| 40 | 2009 | 55.1 | 59.38 | 75.58 | 75.25 | 24000000 | 120000000 | 53000 | 298.62 | 26.81 | 5.00 | 809.79 | 1.79 |
| 40 | 2010 | 50.94 | 59.79 | 68.38 | 73.71 | 11000000 | 150000000 | 81000 | 334.02 | 41.11 | 5.00 | 905.91 | 55.19 |
| 40 | 2011 | 49.48 | 61.79 | 65.29 | 71.88 | 11000000 | 300000000 | 186000 | 387.08 | 39.52 | 5.00 | 919.49 | 46.81 |
| 40 | 2012 | 44.87 | 60.33 | 62.25 | 67.17 | 6900000 | 110000000 | 167000 | 424.60 | 30.80 | 5.00 | 919.76 | 1.29 |
| 40 | 2013 | 47.52 | 62.29 | 64.00 | 68.75 | 8400000 | 170000000 | 191000 | 457.96 | 36.45 | 5.00 | 919.57 | 6.40 |
| 40 | 2014 | 46.65 | 63.50 | 63.38 | 66.42 | 45000000 | 280000000 | 334000 | 486.79 | 36.83 | 5.00 | 925.23 | 15.03 |
| 40 | 2015 | 43.65 | 61.38 | 62.50 | 63.42 | 100000 | 130000000 | 354000 | 497.32 | 27.73 | 5.00 | 925.99 | 1.10 |
| 40 | 2016 | 44.29 | 60.63 | 62.29 | 65.67 | 4300000 | 60000000 | 351000 | 471.32 | 23.96 | 5.00 | 1010.30 | 28.98 |
| 40 | 2017 | 50.81 | 61.96 | 66.75 | 72.92 | 6000000 | 68000000 | 352500 | 467.07 | 35.25 | 5.00 | 1464.42 | 9.66 |
| 40 | 2018 | 45.56 | 61.83 | 59.88 | 69.42 | 61000000 | 120000000 | 354000 | 557.06 | 34.34 | 5.00 | 1622.52 | 15.46 |
| 40 | 2019 | 44.65 | 58.67 | 59.46 | 71.17 | 120000000 | 160000000 | 355500 | 580.72 | 30.11 | 5.00 | 1780.63 | 5.70 |
| 41 | 2000 | 41.54 | 51.00 | 65.67 | 66.42 | 1300000000 | 1500000000 | 557000 | 2520.48 | 15.92 | 5.00 | 2087.90 | 12.55 |
| 41 | 2001 | 38.54 | 48.04 | 62.13 | 66.92 | 1500000000 | 1600000000 | 616000 | 2439.68 | 15.39 | 5.00 | 2299.63 | 36.47 |
| 41 | 2002 | 38.62 | 48.29 | 62.29 | 66.67 | 1200000000 | 1400000000 | 567000 | 2396.63 | 14.82 | 5.00 | 2504.24 | 1.74 |
| 41 | 2003 | 38.65 | 46.13 | 63.96 | 67.21 | 1200000000 | 1300000000 | 625000 | 2281.40 | 16.58 | 5.00 | 2877.65 | 3.75 |
| 41 | 2004 | 36.65 | 42.83 | 64.21 | 66.25 | 1500000000 | 1500000000 | 791000 | 2782.62 | 16.77 | 5.00 | 2628.61 | 20.09 |
| 41 | 2005 | 35.87 | 42.25 | 63.71 | 65.79 | 1900000000 | 1900000000 | 933000 | 3414.47 | 16.97 | 5.00 | 2320.83 | 47.88 |
| 41 | 2006 | 34.4 | 42.75 | 62.08 | 63.96 | 2400000000 | 2200000000 | 2000000 | 3741.09 | 17.73 | 6.00 | 2361.14 | 24.00 |
| 41 | 2007 | 34.4 | 43.54 | 63.13 | 62.13 | 2600000000 | 2500000000 | 2300000 | 4714.07 | 16.43 | 6.00 | 2078.29 | 56.90 |
| 41 | 2008 | 34.37 | 41.67 | 63.96 | 63.13 | 2900000000 | 2800000000 | 2300000 | 5472.54 | 18.11 | 6.00 | 1967.71 | 61.00 |
| 41 | 2009 | 35.5 | 41.00 | 63.67 | 66.33 | 3100000000 | 2800000000 | 2400000 | 5193.24 | 16.42 | 6.00 | 2158.26 | 32.36 |
| 41 | 2010 | 32.37 | 38.83 | 60.08 | 65.83 | 3400000000 | 3200000000 | 1400000 | 6336.71 | 16.34 | 6.00 | 1898.57 | 62.72 |
| 41 | 2011 | 30.73 | 37.46 | 60.00 | 64.00 | 3800000000 | 3600000000 | 2000000 | 7335.11 | 19.28 | 7.00 | 1848.14 | 38.97 |
| 41 | 2012 | 31.21 | 40.71 | 58.75 | 62.96 | 4400000000 | 4400000000 | 2200000 | 8050.06 | 18.81 | 7.00 | 1796.90 | 5.65 |
| 41 | 2013 | 31.52 | 42.46 | 58.25 | 62.33 | 4800000000 | 4500000000 | 2300000 | 8218.03 | 18.09 | 7.00 | 1868.79 | 12.80 |
| 41 | 2014 | 30.58 | 40.00 | 59.00 | 62.17 | 4900000000 | 5200000000 | 2600000 | 8114.08 | 16.63 | 8.00 | 2001.78 | 5.25 |
| 41 | 2015 | 34.15 | 40.25 | 64.29 | 63.75 | 5200000000 | 4800000000 | 3000000 | 6175.88 | 15.65 | 8.00 | 2741.88 | 1.05 |
| 41 | 2016 | 34.02 | 38.75 | 63.04 | 66.25 | 5600000000 | 4800000000 | 3300000 | 5871.22 | 14.72 | 8.00 | 3054.12 | 0.90 |
| 41 | 2017 | 32.29 | 38.25 | 60.71 | 65.63 | 5900000000 | 5100000000 | 3600000 | 6377.85 | 15.15 | 8.00 | 2951.33 | 8.10 |
| 41 | 2018 | 31.87 | 38.08 | 60.71 | 64.96 | 6600000000 | 5600000000 | 3900000 | 6718.59 | 15.89 | 9.00 | 2955.70 | 25.19 |
| 41 | 2019 | 32.56 | 39.50 | 61.54 | 64.08 | 6800000000 | 5800000000 | 4500000 | 6428.68 | 15.80 | 9.00 | 2960.08 | 35.00 |
| 42 | 2000 | 24.23 | 20.58 | 62.46 | 65.42 | 1600000000 | 550000000 | 1100000 | 3772.87 | 43.11 | 4.00 | 308.19 | 2.39 |
| 42 | 2001 | 23.6 | 18.33 | 62.63 | 66.25 | 1500000000 | 430000000 | 1100000 | 3944.74 | 40.67 | 4.00 | 328.87 | 3.60 |
| 42 | 2002 | 26.33 | 24.21 | 62.96 | 65.50 | 1400000000 | 430000000 | 1100000 | 4024.65 | 39.40 | 5.00 | 359.82 | 70.00 |
| 42 | 2003 | 27.33 | 25.21 | 63.75 | 65.71 | 1600000000 | 480000000 | 1200000 | 4129.63 | 40.85 | 5.00 | 398.66 | 12.93 |
| 42 | 2004 | 27.25 | 26.50 | 62.88 | 65.13 | 1800000000 | 530000000 | 1500000 | 4385.39 | 42.39 | 6.00 | 437.94 | 12.74 |
| 42 | 2005 | 27.46 | 27.75 | 61.67 | 65.50 | 2000000000 | 590000000 | 1700000 | 4654.82 | 43.67 | 6.00 | 477.79 | 2.85 |
| 42 | 2006 | 28.04 | 29.25 | 60.67 | 66.17 | 2100000000 | 650000000 | 1700000 | 5201.51 | 43.53 | 6.00 | 511.30 | 31.18 |
| 42 | 2007 | 25.21 | 27.42 | 59.83 | 63.17 | 2500000000 | 830000000 | 2000000 | 6071.79 | 40.80 | 6.00 | 516.62 | 8.52 |
| 42 | 2008 | 25.75 | 28.13 | 60.08 | 63.29 | 2800000000 | 750000000 | 2100000 | 6859.08 | 39.05 | 6.00 | 526.24 | 9.61 |
| 42 | 2009 | 29.44 | 28.13 | 61.67 | 69.08 | 2200000000 | 500000000 | 1900000 | 6760.48 | 34.84 | 6.00 | 573.29 | 7.38 |
| 42 | 2010 | 26.75 | 26.08 | 59.75 | 67.67 | 2400000000 | 630000000 | 2100000 | 8141.91 | 33.17 | 6.00 | 525.83 | 20.93 |
| 42 | 2011 | 25.15 | 26.88 | 58.25 | 65.17 | 2600000000 | 620000000 | 2200000 | 9121.93 | 32.86 | 6.00 | 505.66 | 52.00 |
| 42 | 2012 | 25.9 | 28.04 | 59.08 | 64.67 | 2800000000 | 650000000 | 2300000 | 9913.21 | 32.17 | 6.00 | 502.90 | 8.30 |
| 42 | 2013 | 27.12 | 30.08 | 59.58 | 64.58 | 3200000000 | 650000000 | 2400000 | 10490.10 | 31.33 | 6.00 | 499.77 | 11.99 |
| 42 | 2014 | 26.94 | 27.88 | 60.88 | 65.13 | 3100000000 | 660000000 | 2500000 | 10547.20 | 32.26 | 7.00 | 538.32 | 13.66 |
| 42 | 2015 | 26.6 | 27.58 | 60.29 | 65.33 | 3400000000 | 900000000 | 2700000 | 11299.10 | 30.82 | 7.00 | 534.57 | 0.31 |
| 42 | 2016 | 26.33 | 28.33 | 60.21 | 64.13 | 3800000000 | 1100000000 | 2900000 | 11666.50 | 32.10 | 7.00 | 544.74 | 54.82 |
| 42 | 2017 | 26.02 | 28.79 | 60.25 | 63.00 | 3800000000 | 1300000000 | 3000000 | 11814.60 | 32.76 | 7.00 | 567.51 | 63.51 |
| 42 | 2018 | 26.21 | 28.25 | 60.58 | 63.58 | 3900000000 | 1300000000 | 3000000 | 12112.10 | 33.48 | 7.00 | 576.97 | 1.50 |
| 42 | 2019 | 27.1 | 28.38 | 61.00 | 64.83 | 4100000000 | 1400000000 | 3400000 | 12409.60 | 34.19 | 7.00 | 586.43 | 63.60 |
| 43 | 2000 | 38.29 | 41.17 | 68.50 | 66.92 | 1900000000 | 1900000000 | 1700000 | 2747.10 | 14.13 | 6.00 | 0.66 | 66.00 |
| 43 | 2001 | 35.75 | 37.96 | 68.17 | 65.38 | 1800000000 | 1800000000 | 1700000 | 2837.74 | 13.23 | 7.00 | 0.69 | 1.75 |
| 43 | 2002 | 35.5 | 37.67 | 68.63 | 64.71 | 1800000000 | 1800000000 | 1700000 | 2999.25 | 11.53 | 7.00 | 0.67 | 26.00 |
| 43 | 2003 | 38.92 | 40.42 | 70.67 | 66.75 | 2000000000 | 2000000000 | 1800000 | 3197.13 | 12.95 | 7.00 | 0.61 | 6.50 |
| 43 | 2004 | 37.08 | 41.79 | 66.88 | 65.50 | 2100000000 | 2100000000 | 2000000 | 3395.71 | 16.02 | 7.00 | 0.55 | 7.96 |
| 43 | 2005 | 35.21 | 40.96 | 64.38 | 65.08 | 2600000000 | 2600000000 | 2300000 | 3786.66 | 21.02 | 8.00 | 0.55 | 28.24 |
| 43 | 2006 | 33.6 | 40.29 | 63.08 | 63.83 | 2400000000 | 2400000000 | 2200000 | 4683.57 | 18.71 | 8.00 | 0.54 | 17.35 |
| 43 | 2007 | 32.52 | 41.00 | 60.88 | 63.17 | 2400000000 | 2400000000 | 2100000 | 5208.72 | 20.34 | 8.00 | 0.50 | 28.18 |
| 43 | 2008 | 33.81 | 42.13 | 62.08 | 63.42 | 2500000000 | 2500000000 | 2300000 | 5411.27 | 20.57 | 9.00 | 0.54 | 16.20 |
| 43 | 2009 | 36.15 | 42.79 | 65.00 | 64.50 | 2200000000 | 2200000000 | 2400000 | 5529.67 | 17.46 | 9.00 | 0.64 | 27.09 |
| 43 | 2010 | 33.96 | 42.67 | 61.75 | 63.50 | 2400000000 | 2400000000 | 2500000 | 5730.35 | 22.57 | 9.00 | 0.65 | 59.72 |
| 43 | 2011 | 34 | 43.08 | 61.13 | 63.79 | 2500000000 | 2500000000 | 2700000 | 6139.72 | 25.10 | 9.00 | 0.62 | 89.51 |
| 43 | 2012 | 33.87 | 43.46 | 60.29 | 64.00 | 2600000000 | 2600000000 | 2800000 | 6497.32 | 25.51 | 9.00 | 0.63 | 44.58 |
| 43 | 2013 | 33.94 | 42.96 | 60.83 | 64.08 | 2600000000 | 2600000000 | 2800000 | 6837.71 | 24.10 | 9.00 | 0.64 | 25.12 |
| 43 | 2014 | 33.69 | 41.25 | 61.13 | 65.00 | 2500000000 | 2500000000 | 3000000 | 7133.34 | 22.08 | 9.00 | 0.61 | 4.29 |
| 43 | 2015 | 31.4 | 39.08 | 59.46 | 64.25 | 2800000000 | 2800000000 | 3500000 | 7694.01 | 17.15 | 9.00 | 0.65 | 0.63 |
| 43 | 2016 | 30.31 | 38.71 | 58.08 | 63.83 | 3100000000 | 3100000000 | 4000000 | 8060.80 | 14.98 | 9.00 | 0.74 | 86.65 |
| 43 | 2017 | 32.4 | 38.96 | 58.92 | 66.92 | 3300000000 | 3300000000 | 4600000 | 8541.21 | 14.54 | 9.00 | 0.78 | 72.16 |
| 43 | 2018 | 31.52 | 38.79 | 56.50 | 67.75 | 2800000000 | 2800000000 | 4700000 | 8821.82 | 14.50 | 9.00 | 0.75 | 2.00 |
| 43 | 2019 | 29.85 | 36.88 | 56.71 | 66.13 | 2600000000 | 2600000000 | 4300000 | 9102.43 | 14.46 | 9.00 | 0.72 | 14.84 |
| 44 | 2000 | 37.08 | 30.00 | 73.58 | 70.58 | 80000000 | 77000000 | 105000 | 954.40 | 96.08 | 0.00 | 182.43 | 18.02 |
| 44 | 2001 | 34.81 | 29.04 | 69.83 | 70.75 | 65000000 | 62000000 | 99000 | 955.67 | 92.87 | 0.00 | 187.32 | 7.20 |
| 44 | 2002 | 36.83 | 30.42 | 70.08 | 73.17 | 53000000 | 44000000 | 104000 | 974.95 | 92.30 | 0.00 | 190.67 | 46.87 |
| 44 | 2003 | 38.06 | 33.29 | 70.13 | 72.71 | 28000000 | 30000000 | 101000 | 997.21 | 89.68 | 0.00 | 193.88 | 5.74 |
| 44 | 2004 | 38 | 34.75 | 68.33 | 72.92 | 41000000 | 37000000 | 122000 | 1056.42 | 95.56 | 0.00 | 198.31 | 79.66 |
| 44 | 2005 | 38.27 | 36.08 | 67.38 | 73.08 | 53000000 | 44000000 | 117000 | 1105.50 | 84.62 | 0.00 | 199.88 | 5.24 |
| 44 | 2006 | 37.9 | 37.88 | 66.83 | 71.08 | 66000000 | 51000000 | 113000 | 1954.13 | 50.37 | 0.00 | 200.19 | 2.01 |
| 44 | 2007 | 39.1 | 40.00 | 66.00 | 72.21 | 78000000 | 58000000 | 134000 | 2331.20 | 51.24 | 0.00 | 202.35 | 0.30 |
| 44 | 2008 | 42.15 | 40.67 | 66.00 | 77.63 | 91000000 | 65000000 | 130000 | 2566.89 | 52.95 | 0.00 | 203.63 | 15.34 |
| 44 | 2009 | 42.31 | 40.50 | 66.29 | 77.83 | 100000000 | 72000000 | 141000 | 2756.82 | 48.93 | 0.00 | 203.95 | 25.10 |
| 44 | 2010 | 38.37 | 40.50 | 64.50 | 71.75 | 120000000 | 79000000 | 152000 | 3033.25 | 38.74 | 1.00 | 203.64 | 11.16 |
| 44 | 2011 | 36.04 | 40.50 | 62.75 | 68.83 | 130000000 | 86000000 | 157000 | 3425.44 | 43.47 | 1.00 | 204.02 | 10.94 |
| 44 | 2012 | 38.42 | 41.75 | 63.25 | 71.83 | 140000000 | 93000000 | 177000 | 3788.15 | 49.47 | 1.00 | 204.36 | 3.27 |
| 44 | 2013 | 36.81 | 42.25 | 62.83 | 68.54 | 150000000 | 100000000 | 200000 | 3934.87 | 46.03 | 1.00 | 205.39 | 6.80 |
| 44 | 2014 | 37.87 | 42.50 | 63.50 | 69.75 | 170000000 | 110000000 | 206000 | 4031.60 | 37.93 | 1.00 | 206.45 | 78.10 |
| 44 | 2015 | 36.48 | 41.46 | 63.29 | 68.21 | 180000000 | 110000000 | 207000 | 4166.13 | 36.01 | 1.00 | 206.50 | 83.70 |
| 44 | 2016 | 34.52 | 39.25 | 63.04 | 66.75 | 190000000 | 120000000 | 235000 | 4542.62 | 41.11 | 1.00 | 206.50 | 28.90 |
| 44 | 2017 | 31.12 | 36.96 | 60.00 | 65.29 | 200000000 | 130000000 | 247000 | 4586.05 | 40.43 | 1.00 | 206.50 | 7.80 |
| 44 | 2018 | 31.83 | 38.25 | 59.75 | 65.67 | 220000000 | 140000000 | 287000 | 4979.00 | 35.47 | 1.00 | 207.72 | 0.64 |
| 44 | 2019 | 34.12 | 40.42 | 61.08 | 66.75 | 230000000 | 140000000 | 315000 | 6609.59 | 30.51 | 1.00 | 208.93 | 2.06 |
| 45 | 2000 | 34.5 | 29.08 | 66.88 | 73.04 | 400000000 | 480000000 | 1700000 | 1229.00 | 56.60 | 0.00 | 142.13 | 72.40 |
| 45 | 2001 | 27.44 | 30.75 | 61.54 | 62.58 | 500000000 | 760000000 | 2700000 | 1490.93 | 45.90 | 0.00 | 146.74 | 2.72 |
| 45 | 2002 | 27.87 | 30.88 | 62.67 | 62.21 | 680000000 | 860000000 | 3700000 | 1658.03 | 46.99 | 0.00 | 153.28 | 5.49 |
| 45 | 2003 | 27.77 | 30.08 | 62.58 | 62.88 | 640000000 | 780000000 | 3200000 | 2068.12 | 48.42 | 1.00 | 149.58 | 47.93 |
| 45 | 2004 | 26.21 | 29.54 | 61.58 | 61.29 | 800000000 | 1000000000 | 4300000 | 2874.29 | 52.50 | 2.00 | 136.04 | 2.40 |
| 45 | 2005 | 25.08 | 27.83 | 60.75 | 61.58 | 800000000 | 940000000 | 4400000 | 3771.28 | 53.19 | 2.00 | 132.88 | 2.03 |
| 45 | 2006 | 23.87 | 25.50 | 62.17 | 60.08 | 970000000 | 1100000000 | 4700000 | 5291.58 | 50.98 | 2.00 | 126.09 | 28.97 |
| 45 | 2007 | 25.56 | 25.38 | 65.33 | 60.42 | 1200000000 | 1400000000 | 5300000 | 6771.42 | 49.31 | 2.00 | 122.55 | 1.52 |
| 45 | 2008 | 27.29 | 24.92 | 65.13 | 64.54 | 1300000000 | 1400000000 | 4100000 | 8513.56 | 57.15 | 3.00 | 120.30 | 28.79 |
| 45 | 2009 | 34.54 | 27.58 | 71.92 | 69.58 | 1200000000 | 1300000000 | 3800000 | 7165.22 | 41.84 | 3.00 | 147.50 | 37.98 |
| 45 | 2010 | 29.23 | 28.71 | 66.17 | 63.58 | 1200000000 | 1500000000 | 4100000 | 9070.49 | 44.24 | 3.00 | 147.36 | 3.60 |
| 45 | 2011 | 28.35 | 31.21 | 65.04 | 60.46 | 1500000000 | 1800000000 | 5700000 | 11634.00 | 46.46 | 3.00 | 146.62 | 53.63 |
| 45 | 2012 | 28.25 | 33.25 | 63.71 | 59.54 | 2200000000 | 3600000000 | 6200000 | 12386.70 | 44.11 | 3.00 | 149.11 | 15.00 |
| 45 | 2013 | 27.1 | 34.08 | 61.25 | 58.88 | 2400000000 | 3800000000 | 6800000 | 13890.60 | 38.62 | 3.00 | 152.13 | 43.90 |
| 45 | 2014 | 29.15 | 34.38 | 62.75 | 61.17 | 2200000000 | 3800000000 | 6300000 | 12807.30 | 39.34 | 4.00 | 179.19 | 4.30 |
| 45 | 2015 | 32.21 | 37.38 | 63.17 | 63.88 | 1800000000 | 3000000000 | 6400000 | 10510.80 | 28.52 | 4.00 | 221.73 | 72.51 |
| 45 | 2016 | 36.92 | 37.71 | 67.75 | 68.38 | 2000000000 | 2600000000 | 6500000 | 7714.84 | 31.84 | 5.00 | 342.16 | 24.66 |
| 45 | 2017 | 32.6 | 37.00 | 62.79 | 65.42 | 2400000000 | 2700000000 | 7700000 | 9247.58 | 32.40 | 5.00 | 326.00 | 0.61 |
| 45 | 2018 | 30.35 | 35.50 | 62.54 | 62.67 | 2700000000 | 2900000000 | 8800000 | 9812.60 | 37.63 | 5.00 | 344.71 | 40.41 |
| 45 | 2019 | 28.56 | 32.38 | 62.83 | 61.92 | 2900000000 | 3000000000 | 8500000 | 9812.39 | 36.24 | 5.00 | 363.41 | 0.43 |
| 46 | 2000 | 43.54 | 51.92 | 66.79 | 68.38 | 130000000 | 170000000 | 140000 | 467.15 | 12.70 | 1.00 | 21.17 | 51.64 |
| 46 | 2001 | 41.5 | 47.83 | 65.63 | 69.54 | 110000000 | 170000000 | 142000 | 417.76 | 12.36 | 1.00 | 24.43 | 0.73 |
| 46 | 2002 | 45.02 | 52.67 | 65.00 | 72.38 | 110000000 | 170000000 | 140000 | 396.63 | 12.13 | 1.00 | 29.25 | 68.93 |
| 46 | 2003 | 48.62 | 55.13 | 68.25 | 73.88 | 96000000 | 200000000 | 136000 | 332.62 | 15.82 | 1.00 | 42.37 | 8.20 |
| 46 | 2004 | 48.15 | 60.38 | 62.83 | 73.08 | 93000000 | 210000000 | 96000 | 391.04 | 15.35 | 1.00 | 38.35 | 1.00 |
| 46 | 2005 | 48.58 | 60.96 | 64.08 | 72.13 | 80000000 | 170000000 | 112000 | 468.76 | 14.04 | 1.00 | 40.45 | 20.70 |
| 46 | 2006 | 45.62 | 60.13 | 62.38 | 68.75 | 130000000 | 240000000 | 108000 | 508.97 | 14.49 | 1.00 | 40.41 | 0.18 |
| 46 | 2007 | 42.98 | 57.54 | 61.54 | 66.88 | 190000000 | 330000000 | 386000 | 619.81 | 13.24 | 1.00 | 36.86 | 19.62 |
| 46 | 2008 | 41.5 | 56.75 | 61.25 | 65.00 | 360000000 | 380000000 | 258000 | 678.85 | 12.73 | 1.00 | 39.11 | 19.77 |
| 46 | 2009 | 47.83 | 57.33 | 68.25 | 70.08 | 420000000 | 430000000 | 387000 | 672.04 | 15.71 | 1.00 | 41.20 | 0.84 |
| 46 | 2010 | 50.4 | 57.63 | 73.67 | 69.50 | 380000000 | 430000000 | 255000 | 665.63 | 15.34 | 1.00 | 39.80 | 19.52 |
| 46 | 2011 | 47.65 | 56.63 | 63.17 | 75.50 | 460000000 | 460000000 | 349000 | 744.22 | 17.45 | 1.00 | 40.52 | 4.40 |
| 46 | 2012 | 40.62 | 54.13 | 58.71 | 68.42 | 450000000 | 470000000 | 349000 | 769.75 | 16.91 | 1.00 | 41.95 | 7.61 |
| 46 | 2013 | 41.02 | 54.50 | 59.13 | 68.42 | 550000000 | 420000000 | 420000 | 815.82 | 18.19 | 1.00 | 43.46 | 2.81 |
| 46 | 2014 | 40.42 | 54.21 | 59.04 | 67.58 | 580000000 | 480000000 | 465200 | 831.87 | 18.52 | 1.00 | 45.22 | 0.29 |
| 46 | 2015 | 41.15 | 56.04 | 59.17 | 67.08 | 610000000 | 500000000 | 516000 | 815.73 | 20.01 | 1.00 | 50.71 | 5.55 |
| 46 | 2016 | 42.85 | 55.50 | 61.88 | 68.33 | 510000000 | 500000000 | 445000 | 735.30 | 20.72 | 1.00 | 63.34 | 82.55 |
| 46 | 2017 | 38.69 | 50.79 | 57.33 | 69.25 | 460000000 | 490000000 | 467000 | 765.73 | 18.84 | 1.00 | 64.77 | 38.01 |
| 46 | 2018 | 38.08 | 49.29 | 58.25 | 68.63 | 620000000 | 600000000 | 447000 | 868.34 | 17.08 | 1.00 | 68.03 | 68.69 |
| 46 | 2019 | 42.52 | 50.96 | 63.04 | 71.04 | 540000000 | 550000000 | 938000 | 1272.49 | 11.07 | 1.00 | 71.29 | 87.76 |
| 47 | 2000 | 51.81 | 28.33 | 79.63 | 82.00 | 8500000000 | 7900000000 | 5300000 | 12257.00 | 33.94 | 7.00 | 1130.96 | 75.70 |
| 47 | 2001 | 52.56 | 25.88 | 80.17 | 82.92 | 7900000000 | 8300000000 | 5100000 | 11561.20 | 31.79 | 7.00 | 1290.99 | 5.50 |
| 47 | 2002 | 53.43 | 30.50 | 80.50 | 82.71 | 7600000000 | 11000000000 | 5300000 | 13165.10 | 29.77 | 7.00 | 1251.09 | 27.20 |
| 47 | 2003 | 45.4 | 32.71 | 73.50 | 70.00 | 7000000000 | 11000000000 | 4800000 | 14672.90 | 31.40 | 7.00 | 1191.61 | 0.23 |
| 47 | 2004 | 49.94 | 35.42 | 81.00 | 70.33 | 8200000000 | 14000000000 | 5800000 | 16496.10 | 36.75 | 7.00 | 1145.32 | 5.61 |
| 47 | 2005 | 36.79 | 37.65 | 61.67 | 64.00 | 8300000000 | 17000000000 | 6000000 | 19402.50 | 35.28 | 7.00 | 1024.12 | 35.83 |
| 47 | 2006 | 35.73 | 38.48 | 60.00 | 64.00 | 8400000000 | 21000000000 | 6200000 | 21743.50 | 35.63 | 7.00 | 954.79 | 19.46 |
| 47 | 2007 | 35.5 | 36.56 | 60.00 | 64.00 | 9300000000 | 24000000000 | 6400000 | 24086.40 | 37.39 | 8.00 | 929.26 | 2.54 |
| 47 | 2008 | 37.73 | 35.85 | 61.67 | 66.50 | 13000000000 | 21000000000 | 6900000 | 21350.40 | 47.64 | 8.00 | 1102.05 | 56.08 |
| 47 | 2009 | 42.5 | 36.08 | 65.13 | 71.71 | 13000000000 | 16000000000 | 7800000 | 19143.90 | 45.19 | 9.00 | 1276.93 | 54.01 |
| 47 | 2010 | 46.31 | 35.63 | 66.54 | 73.67 | 14000000000 | 21000000000 | 8800000 | 23087.20 | 47.10 | 10.00 | 1156.06 | 4.98 |
| 47 | 2011 | 44.73 | 35.42 | 66.58 | 69.88 | 17000000000 | 22000000000 | 9800000 | 25096.30 | 53.34 | 10.00 | 1108.29 | 9.50 |
| 47 | 2012 | 45.9 | 35.23 | 66.50 | 72.00 | 19000000000 | 23000000000 | 11000000 | 25466.80 | 54.09 | 10.00 | 1126.47 | 17.16 |
| 47 | 2013 | 46.81 | 36.42 | 67.25 | 73.50 | 19000000000 | 24000000000 | 12000000 | 27182.70 | 51.29 | 10.00 | 1094.85 | 1.30 |
| 47 | 2014 | 46.79 | 35.56 | 67.79 | 73.63 | 22000000000 | 26000000000 | 14000000 | 29249.60 | 47.83 | 11.00 | 1052.96 | 30.00 |
| 47 | 2015 | 44.19 | 36.35 | 67.25 | 70.13 | 19000000000 | 28000000000 | 13000000 | 28732.20 | 42.99 | 12.00 | 1131.16 | 12.99 |
| 47 | 2016 | 44 | 35.65 | 66.50 | 70.50 | 21000000000 | 30000000000 | 17000000 | 29288.90 | 40.13 | 12.00 | 1160.43 | 18.24 |
| 47 | 2017 | 43.87 | 32.92 | 66.21 | 70.33 | 17000000000 | 34000000000 | 13000000 | 31616.80 | 40.93 | 12.00 | 1130.42 | 6.50 |
| 47 | 2018 | 43.44 | 32.31 | 66.50 | 70.83 | 23000000000 | 38000000000 | 15000000 | 33340.30 | 41.63 | 13.00 | 1100.50 | 15.22 |
| 47 | 2019 | 47.25 | 33.31 | 70.17 | 74.83 | 26000000000 | 35000000000 | 17000000 | 35063.70 | 39.95 | 13.00 | 1070.58 | 42.19 |
| 48 | 2000 | 12.4 | 4.75 | 64.21 | 55.83 | 11000000000 | 14000000000 | 10000000 | 26149.40 | 66.03 | 7.00 | 0.66 | 2.53 |
| 48 | 2001 | 11.6 | 3.92 | 62.38 | 56.92 | 11000000000 | 13000000000 | 9500000 | 26873.30 | 63.30 | 7.00 | 0.69 | 4.51 |
| 48 | 2002 | 14.44 | 8.33 | 62.29 | 58.25 | 12000000000 | 14000000000 | 9600000 | 29204.00 | 60.15 | 7.00 | 0.67 | 29.40 |
| 48 | 2003 | 15.77 | 10.29 | 60.75 | 60.50 | 15000000000 | 16000000000 | 9200000 | 35672.20 | 59.28 | 7.00 | 0.61 | 50.37 |
| 48 | 2004 | 14.12 | 10.46 | 59.42 | 58.38 | 16000000000 | 17000000000 | 9600000 | 40362.40 | 62.67 | 7.00 | 0.55 | 32.25 |
| 48 | 2005 | 16.12 | 14.33 | 59.29 | 58.63 | 16000000000 | 18000000000 | 10000000 | 41979.10 | 65.63 | 7.00 | 0.55 | 60.80 |
| 48 | 2006 | 17.19 | 16.83 | 59.67 | 57.88 | 16000000000 | 18000000000 | 11000000 | 44863.40 | 68.18 | 7.00 | 0.54 | 59.00 |
| 48 | 2007 | 14.6 | 15.58 | 59.08 | 54.54 | 16000000000 | 18000000000 | 11000000 | 51733.40 | 68.78 | 7.00 | 0.50 | 33.52 |
| 48 | 2008 | 14.87 | 14.96 | 59.25 | 55.54 | 17000000000 | 19000000000 | 10000000 | 57644.50 | 69.79 | 7.00 | 0.54 | 69.00 |
| 48 | 2009 | 18.52 | 14.83 | 60.71 | 61.50 | 17000000000 | 19000000000 | 9900000 | 52514.00 | 62.24 | 8.00 | 0.64 | 47.06 |
| 48 | 2010 | 18.31 | 13.63 | 61.58 | 61.42 | 18000000000 | 19000000000 | 11000000 | 50950.00 | 69.80 | 9.00 | 0.65 | 7.83 |
| 48 | 2011 | 17.85 | 14.21 | 61.58 | 59.92 | 18000000000 | 19000000000 | 11000000 | 54159.30 | 75.50 | 9.00 | 0.62 | 16.40 |
| 48 | 2012 | 20.17 | 16.33 | 62.38 | 61.63 | 18000000000 | 20000000000 | 12000000 | 50073.00 | 79.50 | 9.00 | 0.63 | 5.63 |
| 48 | 2013 | 20.33 | 18.33 | 61.75 | 60.58 | 19000000000 | 21000000000 | 13000000 | 52184.10 | 79.88 | 9.00 | 0.64 | 1.25 |
| 48 | 2014 | 18.98 | 16.92 | 62.04 | 59.00 | 20000000000 | 24000000000 | 14000000 | 52830.20 | 80.58 | 11.00 | 0.61 | 0.95 |
| 48 | 2015 | 19.65 | 16.67 | 64.96 | 57.67 | 18000000000 | 20000000000 | 15000000 | 45175.20 | 82.66 | 11.00 | 0.65 | 8.76 |
| 48 | 2016 | 17.4 | 15.79 | 62.50 | 56.50 | 21000000000 | 23000000000 | 16000000 | 46007.90 | 79.54 | 11.00 | 0.74 | 27.88 |
| 48 | 2017 | 16.94 | 15.04 | 63.25 | 55.58 | 23000000000 | 25000000000 | 18000000 | 48675.20 | 83.39 | 11.00 | 0.78 | 37.99 |
| 48 | 2018 | 16.35 | 15.33 | 63.13 | 54.25 | 26000000000 | 26000000000 | 19000000 | 53048.10 | 84.32 | 11.00 | 0.75 | 2.81 |
| 48 | 2019 | 16.79 | 15.67 | 62.75 | 55.17 | 28000000000 | 27000000000 | 20000000 | 52331.30 | 83.33 | 11.00 | 0.72 | 4.76 |
| 49 | 2000 | 57.02 | 62.50 | 64.35 | 63.71 | 540000000 | 200000000 | 124000 | 1627.07 | 36.82 | 2.00 | 1.08 | 68.82 |
| 49 | 2001 | 52.52 | 48.67 | 63.38 | 62.83 | 510000000 | 210000000 | 130000 | 1909.60 | 38.42 | 2.00 | 1.12 | 13.87 |
| 49 | 2002 | 51.46 | 48.50 | 63.63 | 63.13 | 560000000 | 220000000 | 136000 | 2106.64 | 35.36 | 2.00 | 1.06 | 17.25 |
| 49 | 2003 | 46.4 | 41.04 | 63.08 | 65.52 | 610000000 | 270000000 | 142000 | 2789.16 | 30.61 | 2.00 | 0.88 | 4.16 |
| 49 | 2004 | 43.48 | 38.96 | 63.96 | 62.77 | 660000000 | 300000000 | 188000 | 3380.17 | 42.02 | 2.00 | 0.80 | 34.50 |
| 49 | 2005 | 44.08 | 40.54 | 61.38 | 62.60 | 710000000 | 320000000 | 272000 | 3674.62 | 43.55 | 3.00 | 0.80 | 10.52 |
| 49 | 2006 | 43.79 | 40.50 | 61.40 | 62.00 | 760000000 | 430000000 | 378000 | 4425.68 | 40.56 | 3.00 | 0.80 | 15.04 |
| 49 | 2007 | 43.5 | 40.46 | 60.08 | 59.69 | 660000000 | 310000000 | 984000 | 5976.39 | 43.00 | 3.00 | 0.73 | 9.45 |
| 49 | 2008 | 43.21 | 40.42 | 60.00 | 61.33 | 860000000 | 390000000 | 1000000 | 7367.75 | 39.52 | 3.00 | 0.68 | 25.95 |
| 49 | 2009 | 42.92 | 40.38 | 61.42 | 67.75 | 790000000 | 360000000 | 1000000 | 6727.11 | 34.33 | 3.00 | 0.72 | 61.00 |
| 49 | 2010 | 42.63 | 40.33 | 60.92 | 66.13 | 770000000 | 410000000 | 1100000 | 6682.28 | 37.04 | 3.00 | 0.75 | 16.68 |
| 49 | 2011 | 42.34 | 40.29 | 60.35 | 63.96 | 930000000 | 450000000 | 1200000 | 7318.74 | 42.35 | 3.00 | 0.72 | 14.54 |
| 49 | 2012 | 42.05 | 40.25 | 61.02 | 64.69 | 860000000 | 550000000 | 1300000 | 6586.72 | 43.67 | 3.00 | 0.78 | 16.03 |
| 49 | 2013 | 41.76 | 40.21 | 61.13 | 64.44 | 930000000 | 510000000 | 1300000 | 7189.29 | 41.34 | 3.00 | 0.75 | 0.37 |
| 49 | 2014 | 41.47 | 40.17 | 61.13 | 63.96 | 960000000 | 440000000 | 1400000 | 7388.15 | 40.14 | 3.00 | 0.75 | 61.16 |
| 49 | 2015 | 41.18 | 40.13 | 62.81 | 62.65 | 950000000 | 450000000 | 1600000 | 6517.16 | 42.12 | 3.00 | 0.90 | 66.19 |
| 49 | 2016 | 40.89 | 40.08 | 61.79 | 61.31 | 980000000 | 480000000 | 1700000 | 7033.60 | 40.60 | 4.00 | 0.90 | 1.70 |
| 49 | 2017 | 40.6 | 40.04 | 61.60 | 60.69 | 1100000000 | 520000000 | 1900000 | 7803.41 | 41.06 | 5.00 | 0.89 | 69.37 |
| 49 | 2018 | 40.31 | 40.00 | 61.56 | 59.88 | 1200000000 | 560000000 | 2100000 | 8850.09 | 42.88 | 5.00 | 0.85 | 69.45 |
| 49 | 2019 | 40.02 | 39.96 | 61.25 | 60.42 | 1300000000 | 660000000 | 2300000 | 2574.91 | 40.25 | 5.00 | 24.32 | 1.79 |
| 50 | 2000 | 37.58 | 39.08 | 64.50 | 71.58 | 260000000 | 540000000 | 471000 | 1080.46 | 53.97 | 2.00 | 14.84 | 27.00 |
| 50 | 2001 | 34.98 | 36.83 | 64.38 | 68.75 | 260000000 | 510000000 | 518000 | 1120.55 | 51.38 | 2.00 | 15.48 | 6.80 |
| 50 | 2002 | 35.4 | 37.83 | 64.96 | 68.00 | 310000000 | 560000000 | 550000 | 1122.06 | 52.73 | 2.00 | 16.44 | 11.21 |
| 50 | 2003 | 37.73 | 39.50 | 65.42 | 70.54 | 370000000 | 610000000 | 611000 | 1145.50 | 54.09 | 2.00 | 17.35 | 32.91 |
| 50 | 2004 | 37.87 | 40.08 | 68.50 | 67.17 | 420000000 | 660000000 | 641000 | 1204.48 | 58.42 | 2.00 | 18.21 | 22.29 |
| 50 | 2005 | 35.29 | 40.54 | 63.46 | 66.58 | 470000000 | 710000000 | 673000 | 1296.70 | 59.01 | 2.00 | 18.83 | 30.88 |
| 50 | 2006 | 34.29 | 39.33 | 63.13 | 66.13 | 520000000 | 760000000 | 739000 | 1420.14 | 56.05 | 2.00 | 18.90 | 19.70 |
| 50 | 2007 | 33.02 | 40.13 | 61.08 | 64.83 | 550000000 | 660000000 | 804000 | 1572.07 | 53.51 | 2.00 | 18.90 | 33.64 |
| 50 | 2008 | 34.54 | 41.21 | 60.75 | 67.13 | 620000000 | 860000000 | 869000 | 1727.83 | 51.33 | 2.00 | 18.90 | 64.44 |
| 50 | 2009 | 39.4 | 42.67 | 62.13 | 74.00 | 620000000 | 790000000 | 836000 | 1789.71 | 39.53 | 2.00 | 18.90 | 90.60 |
| 50 | 2010 | 35.81 | 40.54 | 60.25 | 70.83 | 630000000 | 770000000 | 863000 | 1904.35 | 45.76 | 2.00 | 18.90 | 48.60 |
| 50 | 2011 | 33.35 | 39.58 | 59.13 | 68.00 | 640000000 | 930000000 | 871000 | 2088.32 | 51.26 | 2.00 | 18.92 | 27.92 |
| 50 | 2012 | 34.12 | 40.83 | 59.67 | 67.75 | 680000000 | 860000000 | 895000 | 2144.34 | 50.90 | 2.00 | 19.50 | 5.28 |
| 50 | 2013 | 34.9 | 41.00 | 60.50 | 68.29 | 620000000 | 930000000 | 863000 | 2102.59 | 47.94 | 2.00 | 20.35 | 0.75 |
| 50 | 2014 | 34.81 | 40.50 | 60.21 | 68.92 | 710000000 | 960000000 | 868000 | 2206.05 | 47.56 | 2.00 | 20.99 | 85.03 |
| 50 | 2015 | 33.96 | 39.63 | 60.67 | 67.63 | 670000000 | 950000000 | 880000 | 2302.20 | 45.17 | 2.00 | 21.95 | 75.16 |
| 50 | 2016 | 33 | 38.79 | 61.08 | 66.13 | 590000000 | 980000000 | 838000 | 2342.58 | 42.71 | 2.00 | 22.84 | 2.20 |
| 50 | 2017 | 32.58 | 39.42 | 59.96 | 65.79 | 610000000 | 1100000000 | 851000 | 2453.73 | 43.58 | 2.00 | 23.49 | 17.66 |
| 50 | 2018 | 32.33 | 39.17 | 60.00 | 65.50 | 600000000 | 1200000000 | 864000 | 2505.78 | 41.76 | 2.00 | 23.90 | 24.66 |
| 50 | 2019 | 32.25 | 39.08 | 59.75 | 65.67 | 560000000 | 1300000000 | 52000000 | 4619.99 | 17.70 | 2.00 | 6.01 | 10.80 |
| 51 | 2000 | 40.5 | 46.00 | 69.00 | 66.00 | 7800000 | 13000000 | 33000 | 363.48 | 24.52 | 2.00 | 1746.87 | 66.09 |
| 51 | 2001 | 39.58 | 47.42 | 65.58 | 66.17 | 8200000 | 26000000 | 38000 | 336.63 | 28.58 | 2.00 | 1950.56 | 5.97 |
| 51 | 2002 | 37.81 | 49.46 | 60.17 | 66.00 | 8600000 | 38000000 | 43000 | 343.54 | 26.48 | 2.00 | 1975.84 | 80.78 |
| 51 | 2003 | 38.08 | 46.63 | 64.29 | 65.25 | 9000000 | 36000000 | 44000 | 393.74 | 25.96 | 2.00 | 1984.93 | 6.21 |
| 51 | 2004 | 41.96 | 47.00 | 68.50 | 68.42 | 9400000 | 29000000 | 45000 | 410.76 | 24.63 | 2.00 | 2243.93 | 2.76 |
| 51 | 2005 | 47.21 | 50.92 | 72.79 | 70.71 | 9800000 | 38000000 | 45000 | 322.42 | 34.79 | 2.00 | 3644.33 | 0.37 |
| 51 | 2006 | 46.27 | 52.42 | 71.04 | 69.08 | 10000000 | 41000000 | 46100 | 453.40 | 23.82 | 2.00 | 5148.75 | 21.80 |
| 51 | 2007 | 46.06 | 55.58 | 67.71 | 68.83 | 1100000 | 96000000 | 30000 | 659.99 | 23.55 | 2.00 | 4197.75 | 28.40 |
| 51 | 2008 | 48.27 | 56.38 | 69.00 | 71.17 | 2400000 | 30000000 | 24000 | 715.10 | 21.64 | 2.00 | 4601.69 | 11.69 |
| 51 | 2009 | 47.81 | 55.63 | 67.50 | 72.50 | 4900000 | 28000000 | 18200 | 674.09 | 22.52 | 2.00 | 4801.08 | 13.80 |
| 51 | 2010 | 47.69 | 56.58 | 69.17 | 69.63 | 2000000 | 16000000 | 12400 | 672.42 | 30.34 | 2.00 | 5726.07 | 4.02 |
| 51 | 2011 | 49.29 | 52.92 | 72.83 | 72.83 | 2100000 | 49000000 | 131000 | 651.14 | 32.60 | 2.00 | 6658.03 | 7.20 |
| 51 | 2012 | 46.94 | 54.42 | 68.46 | 71.00 | 1700000 | 42000000 | 96000 | 717.05 | 33.14 | 2.00 | 6985.83 | 78.80 |
| 51 | 2013 | 55.62 | 56.54 | 77.71 | 77.00 | 9400000 | 61000000 | 56000 | 769.00 | 26.47 | 2.00 | 6907.88 | 85.82 |
| 51 | 2014 | 53.75 | 55.96 | 74.79 | 76.75 | 17000000 | 29000000 | 33000 | 787.24 | 26.69 | 2.00 | 7014.12 | 30.80 |
| 51 | 2015 | 48.25 | 54.29 | 68.96 | 73.25 | 27000000 | 38000000 | 35000 | 769.26 | 21.50 | 2.00 | 7485.52 | 9.40 |
| 51 | 2016 | 48.17 | 50.00 | 72.13 | 74.21 | 17000000 | 16000000 | 63000 | 732.97 | 29.22 | 2.00 | 8967.93 | 0.78 |
| 51 | 2017 | 43.08 | 50.92 | 66.63 | 68.63 | 17000000 | 100000000 | 99000 | 856.57 | 44.36 | 2.00 | 9088.32 | 2.21 |
| 51 | 2018 | 43.52 | 50.17 | 66.08 | 70.79 | 4700000 | 410000000 | 135000 | 983.31 | 44.69 | 2.00 | 9011.13 | 73.20 |
| 51 | 2019 | 43.42 | 51.92 | 64.83 | 70.08 | 10000000 | 420000000 | 207000 | 962.84 | 30.60 | 2.00 | 8933.95 | 3.85 |
| 52 | 2000 | 55 | 49.75 | 83.17 | 77.08 | 3000000 | 5000000 | 7000 | 308.14 | 21.76 | 2.00 | 710.21 | 5.77 |
| 52 | 2001 | 51.65 | 47.29 | 80.50 | 75.50 | 2800000 | 5500000 | 7800 | 319.68 | 21.19 | 2.00 | 732.40 | 51.93 |
| 52 | 2002 | 51.98 | 51.83 | 78.13 | 74.00 | 2600000 | 10000000 | 7000 | 331.49 | 17.83 | 2.00 | 693.71 | 3.00 |
| 52 | 2003 | 52.4 | 52.04 | 78.75 | 74.00 | 2400000 | 21000000 | 6200 | 371.22 | 18.68 | 2.00 | 579.90 | 2.93 |
| 52 | 2004 | 47.62 | 43.04 | 78.21 | 74.00 | 2200000 | 22000000 | 5600 | 404.35 | 18.46 | 2.00 | 527.34 | 37.00 |
| 52 | 2005 | 48.29 | 44.38 | 78.21 | 74.00 | 9100000 | 19000000 | 5000 | 436.30 | 16.15 | 2.00 | 527.26 | 1.80 |
| 52 | 2006 | 47.85 | 43.88 | 78.33 | 73.50 | 10000000 | 18000000 | 11600 | 429.62 | 12.85 | 2.00 | 522.43 | 34.80 |
| 52 | 2007 | 44.67 | 44.13 | 77.17 | 68.04 | 11000000 | 41000000 | 15600 | 493.07 | 20.19 | 2.00 | 478.63 | 41.44 |
| 52 | 2008 | 43.6 | 44.63 | 73.42 | 69.17 | 12000000 | 46000000 | 18000 | 597.58 | 19.85 | 2.00 | 446.00 | 4.40 |
| 52 | 2009 | 43.67 | 43.75 | 73.33 | 70.25 | 13000000 | 26000000 | 16000 | 557.17 | 18.85 | 2.00 | 470.29 | 59.17 |
| 52 | 2010 | 43.77 | 45.29 | 72.42 | 69.83 | 14000000 | 30000000 | 22300 | 557.63 | 14.91 | 2.00 | 494.79 | 18.74 |
| 52 | 2011 | 43.9 | 47.13 | 71.17 | 69.50 | 15000000 | 39000000 | 36700 | 702.74 | 25.67 | 2.00 | 471.25 | 49.90 |
| 52 | 2012 | 41.25 | 50.13 | 63.88 | 68.50 | 6800000 | 25000000 | 35400 | 616.41 | 15.48 | 2.00 | 510.56 | 4.72 |
| 52 | 2013 | 37.96 | 50.17 | 59.50 | 66.25 | 2200000 | 32000000 | 35500 | 634.48 | 18.26 | 2.00 | 493.90 | 78.92 |
| 52 | 2014 | 37.27 | 49.21 | 60.25 | 65.08 | 5800000 | 39000000 | 36100 | 622.48 | 20.21 | 2.00 | 493.76 | 28.30 |
| 52 | 2015 | 38.77 | 49.17 | 62.88 | 65.50 | 9300000 | 46000000 | 43800 | 603.16 | 27.54 | 2.00 | 591.21 | 0.65 |
| 52 | 2016 | 35.54 | 49.29 | 57.13 | 64.67 | 13000000 | 53000000 | 45200 | 661.01 | 26.51 | 2.00 | 592.61 | 46.90 |
| 52 | 2017 | 36.19 | 48.63 | 58.92 | 64.83 | 16000000 | 60000000 | 46600 | 736.73 | 27.77 | 2.00 | 580.66 | 0.97 |
| 52 | 2018 | 35.46 | 47.17 | 60.29 | 63.46 | 20000000 | 75000000 | 48000 | 777.97 | 25.34 | 2.00 | 555.45 | 55.70 |
| 52 | 2019 | 37.06 | 46.38 | 61.08 | 66.67 | 24000000 | 89000000 | 52400 | 697.30 | 23.50 | 2.00 | 530.24 | 0.81 |
| 53 | 2000 | 15.15 | 12.00 | 61.00 | 57.29 | 13000000000 | 15000000000 | 20000000 | 24190.30 | 44.36 | 16.00 | 1.49 | 75.00 |
| 53 | 2001 | 14.58 | 10.13 | 60.50 | 58.54 | 13000000000 | 15000000000 | 20000000 | 23738.20 | 42.19 | 16.00 | 1.55 | 9.00 |
| 53 | 2002 | 14.83 | 12.42 | 60.00 | 57.25 | 13000000000 | 14000000000 | 20000000 | 24169.30 | 40.20 | 16.00 | 1.57 | 1.80 |
| 53 | 2003 | 14.42 | 12.54 | 59.33 | 56.96 | 12000000000 | 16000000000 | 18000000 | 28200.70 | 37.00 | 16.00 | 1.40 | 25.20 |
| 53 | 2004 | 13.15 | 12.79 | 57.38 | 56.13 | 15000000000 | 19000000000 | 19000000 | 32034.30 | 37.46 | 16.00 | 1.30 | 0.22 |
| 53 | 2005 | 14.42 | 13.50 | 58.63 | 56.71 | 16000000000 | 23000000000 | 19000000 | 36266.20 | 36.97 | 16.00 | 1.21 | 20.45 |
| 53 | 2006 | 14.85 | 14.17 | 59.29 | 56.25 | 17000000000 | 26000000000 | 18000000 | 40385.90 | 35.51 | 16.00 | 1.13 | 21.50 |
| 53 | 2007 | 15.42 | 14.17 | 59.92 | 56.75 | 18000000000 | 31000000000 | 18000000 | 44543.00 | 34.32 | 17.00 | 1.07 | 0.91 |
| 53 | 2008 | 15.65 | 13.79 | 59.08 | 58.42 | 18000000000 | 34000000000 | 17000000 | 46594.40 | 34.48 | 18.00 | 1.07 | 20.81 |
| 53 | 2009 | 18.87 | 14.00 | 60.33 | 63.42 | 16000000000 | 30000000000 | 16000000 | 40773.10 | 28.59 | 18.00 | 1.14 | 4.84 |
| 53 | 2010 | 17.12 | 13.33 | 60.17 | 60.75 | 18000000000 | 37000000000 | 16000000 | 47448.00 | 29.24 | 18.00 | 1.03 | 8.07 |
| 53 | 2011 | 16.81 | 12.21 | 60.25 | 61.17 | 20000000000 | 41000000000 | 16000000 | 52087.40 | 30.75 | 18.00 | 0.99 | 3.90 |
| 53 | 2012 | 17.15 | 12.42 | 60.71 | 61.17 | 22000000000 | 45000000000 | 16000000 | 52678.40 | 30.35 | 19.00 | 1.00 | 0.39 |
| 53 | 2013 | 17.71 | 14.04 | 60.13 | 61.25 | 23000000000 | 49000000000 | 16000000 | 52652.60 | 30.33 | 20.00 | 1.03 | 6.77 |
| 53 | 2014 | 17.77 | 14.38 | 60.67 | 60.50 | 25000000000 | 53000000000 | 17000000 | 50893.40 | 31.74 | 20.00 | 1.10 | 86.93 |
| 53 | 2015 | 18.37 | 14.75 | 62.25 | 59.75 | 26000000000 | 57000000000 | 18000000 | 43585.50 | 31.85 | 20.00 | 1.28 | 42.09 |
| 53 | 2016 | 17.81 | 13.04 | 61.38 | 61.21 | 28000000000 | 61000000000 | 20000000 | 42322.50 | 31.50 | 21.00 | 1.33 | 74.30 |
| 53 | 2017 | 17.44 | 13.58 | 60.29 | 61.00 | 29000000000 | 65000000000 | 21000000 | 45148.60 | 31.41 | 21.00 | 1.30 | 82.01 |
| 53 | 2018 | 17.33 | 14.58 | 59.75 | 60.33 | 31000000000 | 69000000000 | 21000000 | 46313.20 | 32.08 | 22.00 | 1.30 | 77.20 |
| 53 | 2019 | 17.94 | 15.38 | 59.75 | 60.75 | 32000000000 | 73000000000 | 32000000 | 46194.70 | 31.64 | 22.00 | 1.29 | 6.11 |
| 54 | 2000 | 43.85 | 39.00 | 77.63 | 71.08 | 360000000 | 160000000 | 399000 | 258.47 | 48.80 | 2.00 | 0.54 | 33.15 |
| 54 | 2001 | 41.31 | 34.79 | 74.00 | 73.83 | 370000000 | 170000000 | 439000 | 269.02 | 45.23 | 2.00 | 0.72 | 0.24 |
| 54 | 2002 | 38.9 | 37.25 | 69.63 | 70.92 | 380000000 | 180000000 | 483000 | 304.57 | 42.62 | 2.00 | 0.79 | 6.89 |
| 54 | 2003 | 37.69 | 37.88 | 68.21 | 69.29 | 440000000 | 220000000 | 531000 | 367.82 | 40.68 | 2.00 | 0.87 | 40.77 |
| 54 | 2004 | 33.77 | 36.46 | 63.08 | 68.00 | 500000000 | 270000000 | 584000 | 417.51 | 39.30 | 2.00 | 0.90 | 30.00 |
| 54 | 2005 | 31.77 | 33.46 | 62.67 | 67.42 | 870000000 | 470000000 | 429000 | 491.95 | 36.45 | 2.00 | 0.91 | 3.88 |
| 54 | 2006 | 30.42 | 31.67 | 62.92 | 66.25 | 910000000 | 580000000 | 497000 | 912.00 | 25.19 | 2.00 | 0.92 | 61.80 |
| 54 | 2007 | 29.04 | 31.83 | 60.33 | 65.92 | 990000000 | 820000000 | 587000 | 1078.16 | 24.53 | 2.00 | 0.93 | 56.74 |
| 54 | 2008 | 31.69 | 32.96 | 62.08 | 68.33 | 970000000 | 870000000 | 698000 | 1210.62 | 25.03 | 2.00 | 1.05 | 8.66 |
| 54 | 2009 | 35.65 | 33.38 | 66.00 | 71.92 | 850000000 | 950000000 | 803000 | 1074.76 | 29.29 | 2.00 | 1.40 | 14.11 |
| 54 | 2010 | 33.5 | 35.08 | 60.71 | 71.21 | 710000000 | 880000000 | 931000 | 1299.34 | 29.48 | 2.00 | 1.43 | 20.03 |
| 54 | 2011 | 32.06 | 34.67 | 61.63 | 67.83 | 800000000 | 1000000000 | 821000 | 1549.46 | 36.94 | 2.00 | 1.52 | 1.60 |
| 54 | 2012 | 32.21 | 34.21 | 62.38 | 67.83 | 1200000000 | 980000000 | 903000 | 1587.56 | 40.36 | 2.00 | 1.82 | 32.30 |
| 54 | 2013 | 33.77 | 35.75 | 62.17 | 69.63 | 1000000000 | 980000000 | 994000 | 2345.39 | 25.83 | 2.00 | 1.98 | 17.10 |
| 54 | 2014 | 37.56 | 36.00 | 67.88 | 71.25 | 1000000000 | 950000000 | 825000 | 1971.03 | 28.82 | 2.00 | 2.90 | 28.63 |
| 54 | 2015 | 37.4 | 36.71 | 65.58 | 72.50 | 910000000 | 1400000000 | 897000 | 1743.85 | 31.96 | 2.00 | 3.71 | 7.30 |
| 54 | 2016 | 35.6 | 35.92 | 64.46 | 70.83 | 950000000 | 1600000000 | 969000 | 1931.39 | 31.88 | 2.00 | 3.91 | 20.83 |
| 54 | 2017 | 33.96 | 35.21 | 63.96 | 68.75 | 920000000 | 1600000000 | 1000000 | 2025.93 | 35.26 | 2.00 | 4.35 | 44.68 |
| 54 | 2018 | 30.77 | 33.17 | 61.88 | 66.50 | 1000000000 | 1300000000 | 1100000 | 2202.31 | 35.26 | 2.00 | 4.59 | 3.67 |
| 54 | 2019 | 31.52 | 33.50 | 63.04 | 66.50 | 1500000000 | 1400000000 | 1200000 | 2202.12 | 35.98 | 2.00 | 4.82 | 6.55 |
| 55 | 2000 | 33.12 | 37.67 | 66.63 | 61.96 | 99000000 | 180000000 | 155000 | 4125.72 | 69.03 | 1.00 | 710.21 | 34.00 |
| 55 | 2001 | 30.85 | 37.33 | 64.50 | 59.88 | 46000000 | 260000000 | 169000 | 3989.56 | 54.28 | 1.00 | 732.40 | 55.11 |
| 55 | 2002 | 33.58 | 39.17 | 64.63 | 63.38 | 77000000 | 230000000 | 208000 | 4121.96 | 52.61 | 1.00 | 693.71 | 35.88 |
| 55 | 2003 | 34.19 | 39.75 | 66.38 | 62.25 | 84000000 | 240000000 | 222000 | 4922.38 | 51.93 | 1.00 | 579.90 | 64.80 |
| 55 | 2004 | 32.67 | 39.83 | 65.04 | 60.46 | 74000000 | 280000000 | 244000 | 5729.30 | 55.04 | 1.00 | 527.34 | 69.90 |
| 55 | 2005 | 27.67 | 37.92 | 61.50 | 55.92 | 13000000 | 350000000 | 269000 | 6888.63 | 60.01 | 1.00 | 527.26 | 35.05 |
| 55 | 2006 | 26.37 | 38.63 | 59.38 | 54.75 | 20000000 | 340000000 | 294000 | 7214.91 | 60.57 | 1.00 | 522.43 | 69.76 |
| 55 | 2007 | 26.29 | 40.13 | 57.46 | 55.00 | 26000000 | 330000000 | 319000 | 8447.08 | 58.53 | 1.00 | 478.63 | 53.30 |
| 55 | 2008 | 26.35 | 40.13 | 57.58 | 55.00 | 27000000 | 490000000 | 344000 | 10212.80 | 62.20 | 1.00 | 446.00 | 11.50 |
| 55 | 2009 | 27.69 | 40.54 | 57.17 | 57.67 | 26000000 | 480000000 | 369000 | 7690.05 | 52.17 | 1.00 | 470.29 | 21.10 |
| 55 | 2010 | 26.08 | 40.08 | 55.42 | 56.67 | 89000000 | 560000000 | 394000 | 8849.36 | 57.66 | 1.00 | 494.79 | 6.02 |
| 55 | 2011 | 24.71 | 42.00 | 53.67 | 53.75 | 25000000 | 660000000 | 419000 | 10809.60 | 61.74 | 1.00 | 471.25 | 5.01 |
| 55 | 2012 | 25.37 | 40.50 | 55.38 | 54.88 | 40000000 | 440000000 | 444000 | 9813.48 | 60.95 | 2.00 | 510.56 | 0.93 |
| 55 | 2013 | 25.81 | 41.63 | 54.50 | 55.50 | 61000000 | 400000000 | 469000 | 9683.58 | 57.36 | 2.00 | 493.90 | 9.47 |
| 55 | 2014 | 27.79 | 42.79 | 54.79 | 58.00 | 44000000 | 510000000 | 494000 | 9663.43 | 44.52 | 2.00 | 493.76 | 48.13 |
| 55 | 2015 | 31.46 | 44.04 | 59.47 | 59.42 | 29000000 | 350000000 | 519000 | 7384.72 | 46.03 | 2.00 | 591.21 | 40.79 |
| 55 | 2016 | 36.12 | 45.29 | 60.63 | 66.33 | 36000000 | 430000000 | 544000 | 6984.45 | 44.04 | 2.00 | 592.61 | 3.95 |
| 55 | 2017 | 35.25 | 44.63 | 62.46 | 63.42 | 32000000 | 390000000 | 569000 | 7230.40 | 50.23 | 2.00 | 580.66 | 5.79 |
| 55 | 2018 | 35.19 | 44.08 | 62.33 | 63.96 | 34000000 | 410000000 | 594000 | 7956.63 | 50.54 | 2.00 | 555.45 | 75.09 |
| 55 | 2019 | 34.56 | 43.63 | 62.75 | 62.75 | 33000000 | 400000000 | 644000 | 7767.01 | 50.67 | 2.00 | 530.24 | 20.00 |
| 56 | 2000 | 25.06 | 23.50 | 62.04 | 64.58 | 4100000000 | 1600000000 | 4800000 | 6011.62 | 48.19 | 3.00 | 38.60 | 20.76 |
| 56 | 2001 | 24.48 | 22.71 | 61.04 | 65.21 | 3700000000 | 2000000000 | 5400000 | 6609.21 | 49.03 | 3.00 | 38.04 | 4.87 |
| 56 | 2002 | 23.87 | 21.46 | 61.92 | 64.38 | 3400000000 | 1800000000 | 4700000 | 8032.90 | 45.12 | 3.00 | 32.74 | 35.90 |
| 56 | 2003 | 22.37 | 21.50 | 60.25 | 63.00 | 4100000000 | 2200000000 | 5100000 | 9773.12 | 46.91 | 3.00 | 28.21 | 16.00 |
| 56 | 2004 | 23.69 | 23.42 | 60.17 | 63.79 | 4900000000 | 2700000000 | 6100000 | 11685.90 | 57.34 | 3.00 | 25.70 | 23.86 |
| 56 | 2005 | 22.25 | 21.58 | 60.54 | 62.38 | 5800000000 | 2600000000 | 6300000 | 13346.20 | 62.18 | 3.00 | 23.96 | 10.18 |
| 56 | 2006 | 21.37 | 21.38 | 60.13 | 61.25 | 6700000000 | 2900000000 | 6400000 | 15183.60 | 65.19 | 3.00 | 22.60 | 28.11 |
| 56 | 2007 | 21.31 | 19.96 | 60.42 | 62.25 | 7800000000 | 3900000000 | 6700000 | 18373.60 | 66.41 | 3.00 | 20.29 | 63.00 |
| 56 | 2008 | 22.81 | 21.46 | 62.08 | 62.08 | 9200000000 | 5000000000 | 6600000 | 22698.90 | 63.23 | 3.00 | 17.07 | 20.00 |
| 56 | 2009 | 26.37 | 22.00 | 63.75 | 67.00 | 8200000000 | 4300000000 | 6000000 | 19741.60 | 58.68 | 3.00 | 19.06 | 17.08 |
| 56 | 2010 | 24.35 | 21.96 | 61.75 | 65.00 | 8100000000 | 4400000000 | 6300000 | 19808.10 | 66.03 | 3.00 | 19.10 | 18.01 |
| 56 | 2011 | 23.85 | 22.08 | 61.92 | 63.71 | 8900000000 | 4900000000 | 6700000 | 21717.50 | 71.31 | 3.00 | 17.70 | 0.45 |
| 56 | 2012 | 26.42 | 25.00 | 62.54 | 65.29 | 8200000000 | 4600000000 | 7600000 | 19729.90 | 76.17 | 3.00 | 19.58 | 65.34 |
| 56 | 2013 | 26.27 | 25.79 | 61.38 | 65.38 | 7800000000 | 4700000000 | 7900000 | 19916.00 | 76.87 | 3.00 | 19.57 | 70.58 |
| 56 | 2014 | 24 | 24.38 | 61.00 | 62.63 | 7600000000 | 5200000000 | 8100000 | 19744.60 | 82.55 | 3.00 | 20.76 | 1.90 |
| 56 | 2015 | 23.04 | 22.79 | 63.00 | 60.29 | 6800000000 | 4800000000 | 8700000 | 17715.60 | 81.05 | 3.00 | 24.60 | 72.87 |
| 56 | 2016 | 20.85 | 22.13 | 59.83 | 59.75 | 7000000000 | 5000000000 | 9300000 | 18463.40 | 79.56 | 3.00 | 24.44 | 71.67 |
| 56 | 2017 | 20.35 | 21.71 | 60.13 | 58.88 | 7700000000 | 5500000000 | 10000000 | 20379.90 | 79.73 | 3.00 | 23.38 | 1.15 |
| 56 | 2018 | 19.35 | 20.38 | 59.96 | 58.38 | 8300000000 | 6100000000 | 11000000 | 23046.90 | 78.47 | 3.00 | 21.73 | 31.54 |
| 56 | 2019 | 19.98 | 21.38 | 59.67 | 58.92 | 8000000000 | 6000000000 | 11000000 | 23494.60 | 74.39 | 3.00 | 20.08 | 7.00 |
| 57 | 2000 | 50.65 | 51.75 | 70.63 | 78.92 | 130000000 | 140000000 | 2000000 | 563.06 | 38.16 | 4.00 | 0.04 | 14.27 |
| 57 | 2001 | 59.56 | 57.79 | 74.58 | 86.75 | 81000000 | 140000000 | 2200000 | 568.39 | 34.96 | 4.00 | 0.06 | 51.95 |
| 57 | 2002 | 62.23 | 65.71 | 70.50 | 88.25 | 76000000 | 140000000 | 2000000 | 530.53 | 31.83 | 4.00 | 0.06 | 33.82 |
| 57 | 2003 | 65 | 63.08 | 76.67 | 90.25 | 61000000 | 150000000 | 2300000 | 478.01 | 32.40 | 5.00 | 0.70 | 33.83 |
| 57 | 2004 | 64.35 | 58.21 | 80.92 | 89.58 | 190000000 | 150000000 | 1900000 | 483.00 | 34.47 | 5.00 | 5.07 | 23.00 |
| 57 | 2005 | 58.08 | 55.13 | 75.71 | 85.33 | 99000000 | 150000000 | 1600000 | 476.56 | 33.55 | 5.00 | 0.55 | 39.67 |
| 57 | 2006 | 56.54 | 54.33 | 75.08 | 83.67 | 340000000 | 150000000 | 2300000 | 447.86 | 35.96 | 5.00 | 0.54 | 66.00 |
| 57 | 2007 | 56.75 | 57.67 | 72.25 | 83.58 | 370000000 | 160000000 | 2500000 | 431.79 | 37.79 | 5.00 | 0.50 | 91.00 |
| 57 | 2008 | 60.5 | 58.67 | 77.08 | 85.25 | 290000000 | 160000000 | 2000000 | 356.69 | 41.47 | 5.00 | 0.54 | 53.13 |
| 57 | 2009 | 61.6 | 57.38 | 80.17 | 85.67 | 130000000 | 170000000 | 2000000 | 771.60 | 19.48 | 5.00 | 0.64 | 34.66 |
| 57 | 2010 | 52.19 | 56.88 | 73.17 | 74.33 | 140000000 | 200000000 | 2200000 | 948.33 | 29.64 | 5.00 | 0.65 | 6.25 |
| 57 | 2011 | 54.23 | 56.96 | 75.92 | 75.58 | 160000000 | 320000000 | 2400000 | 1093.65 | 34.80 | 5.00 | 0.62 | 0.92 |
| 57 | 2012 | 51.29 | 56.17 | 72.75 | 73.67 | 170000000 | 390000000 | 1800000 | 1304.97 | 25.16 | 5.00 | 0.63 | 85.02 |
| 57 | 2013 | 49.04 | 53.88 | 71.13 | 73.08 | 180000000 | 440000000 | 1800000 | 1430.00 | 21.99 | 5.00 | 0.64 | 78.00 |
| 57 | 2014 | 48.44 | 53.00 | 71.33 | 72.54 | 190000000 | 450000000 | 1900000 | 1434.90 | 20.93 | 5.00 | 0.61 | 2.40 |
| 57 | 2015 | 45.15 | 52.96 | 68.92 | 68.42 | 190000000 | 560000000 | 2100000 | 1445.07 | 19.16 | 5.00 | 0.65 | 20.82 |
| 57 | 2016 | 44.62 | 52.25 | 68.17 | 68.83 | 190000000 | 410000000 | 2200000 | 1464.58 | 19.94 | 5.00 | 0.74 | 26.83 |
| 57 | 2017 | 43.58 | 51.50 | 65.96 | 69.71 | 160000000 | 340000000 | 2400000 | 1548.17 | 19.66 | 5.00 | 0.78 | 18.80 |
| 57 | 2018 | 40 | 46.46 | 64.83 | 68.71 | 180000000 | 370000000 | 2600000 | 1683.74 | 19.00 | 5.00 | 0.75 | 70.68 |
| 57 | 2019 | 44.65 | 48.33 | 64.75 | 76.21 | 170000000 | 360000000 | 2300000 | 1463.99 | 18.34 | 5.00 | 0.72 | 6.22 |
| 58 | 2000 | 40.1 | 43.58 | 71.13 | 65.50 | 130000000 | 240000000 | 295000 | 649.99 | 25.91 | 1.00 | 710.21 | 83.67 |
| 58 | 2001 | 36.35 | 42.50 | 68.13 | 62.08 | 180000000 | 200000000 | 321000 | 651.10 | 25.84 | 1.00 | 732.40 | 6.88 |
| 58 | 2002 | 37.06 | 44.21 | 67.58 | 62.33 | 120000000 | 210000000 | 347000 | 707.89 | 24.30 | 1.00 | 693.71 | 4.29 |
| 58 | 2003 | 37.6 | 45.21 | 66.71 | 63.29 | 270000000 | 270000000 | 373000 | 865.96 | 20.93 | 1.00 | 579.90 | 0.44 |
| 58 | 2004 | 36.12 | 44.71 | 65.21 | 62.33 | 210000000 | 390000000 | 399000 | 1009.94 | 22.71 | 1.00 | 527.34 | 25.60 |
| 58 | 2005 | 33.54 | 40.50 | 64.46 | 62.13 | 230000000 | 480000000 | 425000 | 1011.88 | 24.60 | 1.00 | 527.26 | 32.29 |
| 58 | 2006 | 31.23 | 35.58 | 63.58 | 63.29 | 230000000 | 520000000 | 451000 | 1062.14 | 25.64 | 1.00 | 522.43 | 12.94 |
| 58 | 2007 | 27.08 | 34.67 | 60.29 | 59.21 | 250000000 | 470000000 | 477000 | 1194.07 | 28.89 | 1.00 | 478.63 | 18.20 |
| 58 | 2008 | 26.54 | 34.50 | 59.54 | 59.04 | 170000000 | 560000000 | 487000 | 1371.75 | 29.06 | 1.00 | 446.00 | 11.00 |
| 58 | 2009 | 27.1 | 35.17 | 56.58 | 62.46 | 270000000 | 480000000 | 498000 | 1314.71 | 21.40 | 1.00 | 470.29 | 7.60 |
| 58 | 2010 | 31.85 | 37.58 | 57.21 | 68.92 | 170000000 | 270000000 | 573000 | 1286.52 | 22.17 | 1.00 | 494.79 | 81.00 |
| 58 | 2011 | 33.02 | 41.88 | 57.29 | 66.88 | 420000000 | 620000000 | 604000 | 1405.09 | 25.93 | 1.00 | 471.25 | 87.42 |
| 58 | 2012 | 34.48 | 44.83 | 57.58 | 66.54 | 380000000 | 670000000 | 817000 | 1354.55 | 26.12 | 2.00 | 510.56 | 32.90 |
| 58 | 2013 | 32.77 | 43.88 | 56.17 | 65.50 | 610000000 | 800000000 | 783000 | 1465.64 | 25.57 | 2.00 | 493.90 | 9.60 |
| 58 | 2014 | 34.35 | 46.17 | 56.96 | 65.58 | 630000000 | 830000000 | 822000 | 1542.62 | 24.94 | 2.00 | 493.76 | 0.92 |
| 58 | 2015 | 37.23 | 47.50 | 60.50 | 66.46 | 480000000 | 740000000 | 897000 | 1327.50 | 22.26 | 2.00 | 591.21 | 2.35 |
| 58 | 2016 | 36.17 | 47.00 | 59.04 | 66.29 | 510000000 | 820000000 | 994000 | 1364.33 | 19.24 | 2.00 | 592.61 | 76.70 |
| 58 | 2017 | 36.65 | 48.71 | 58.79 | 65.79 | 540000000 | 850000000 | 1100000 | 1425.11 | 18.58 | 2.00 | 580.66 | 4.27 |
| 58 | 2018 | 37.77 | 51.88 | 59.33 | 64.33 | 630000000 | 920000000 | 1200000 | 1534.49 | 19.31 | 2.00 | 555.45 | 7.50 |
| 58 | 2019 | 39.27 | 52.79 | 61.04 | 64.71 | 680000000 | 980000000 | 1300000 | 1507.45 | 20.21 | 2.00 | 530.24 | 62.97 |
| 59 | 2000 | 31 | 25.00 | 71.50 | 65.50 | 6400000000 | 8800000000 | 580500 | 29976.10 | 67.28 | 0.00 | 3.64 | 3.50 |
| 59 | 2001 | 28.54 | 22.46 | 71.00 | 63.63 | 6400000000 | 8800000000 | 638400 | 28517.30 | 65.89 | 0.00 | 3.64 | 3.40 |
| 59 | 2002 | 23.54 | 24.75 | 65.33 | 57.00 | 6400000000 | 8800000000 | 696300 | 30214.90 | 60.34 | 0.00 | 3.64 | 44.30 |
| 59 | 2003 | 21.42 | 26.83 | 63.50 | 52.50 | 6400000000 | 8800000000 | 754200 | 34517.80 | 61.70 | 0.00 | 3.64 | 1.90 |
| 59 | 2004 | 21.5 | 27.00 | 63.50 | 52.50 | 6400000000 | 8800000000 | 812100 | 42124.80 | 64.17 | 0.00 | 3.64 | 42.00 |
| 59 | 2005 | 20.5 | 26.50 | 62.50 | 52.00 | 6400000000 | 8800000000 | 870000 | 51455.60 | 65.09 | 0.00 | 3.64 | 44.24 |
| 59 | 2006 | 20.5 | 26.50 | 62.50 | 52.00 | 6400000000 | 8800000000 | 927900 | 59530.20 | 62.82 | 0.00 | 3.64 | 5.20 |
| 59 | 2007 | 20.98 | 27.00 | 61.58 | 53.38 | 6400000000 | 8700000000 | 1000000 | 65421.80 | 60.28 | 0.00 | 3.64 | 63.41 |
| 59 | 2008 | 21.08 | 27.00 | 61.67 | 53.50 | 6500000000 | 8900000000 | 1300000 | 80234.50 | 61.36 | 0.00 | 3.64 | 22.53 |
| 59 | 2009 | 21.04 | 27.00 | 61.88 | 53.21 | 6300000000 | 8500000000 | 1500000 | 59094.40 | 51.13 | 0.00 | 3.64 | 55.22 |
| 59 | 2010 | 18.44 | 27.00 | 59.88 | 50.00 | 6600000000 | 9300000000 | 1700000 | 67403.20 | 62.32 | 0.00 | 3.64 | 9.00 |
| 59 | 2011 | 19.69 | 29.75 | 59.00 | 50.63 | 6000000000 | 7800000000 | 2100000 | 82409.60 | 72.62 | 0.00 | 3.64 | 82.23 |
| 59 | 2012 | 18.67 | 27.83 | 58.33 | 51.17 | 7200000000 | 11000000000 | 2300000 | 85076.10 | 76.47 | 0.00 | 3.64 | 32.42 |
| 59 | 2013 | 17.62 | 27.50 | 57.00 | 50.75 | 8500000000 | 12000000000 | 2600000 | 85050.90 | 72.72 | 1.00 | 3.64 | 1.65 |
| 59 | 2014 | 17.98 | 27.42 | 57.04 | 51.50 | 11000000000 | 13000000000 | 2800000 | 83858.50 | 68.00 | 1.00 | 3.64 | 50.08 |
| 59 | 2015 | 18.42 | 26.50 | 57.83 | 52.50 | 12000000000 | 12000000000 | 2900000 | 63039.00 | 57.06 | 1.00 | 3.64 | 0.70 |
| 59 | 2016 | 21.75 | 26.50 | 59.50 | 57.50 | 13000000000 | 13000000000 | 2900000 | 57163.10 | 47.71 | 1.00 | 3.64 | 55.80 |
| 59 | 2017 | 26.62 | 29.00 | 61.96 | 62.29 | 16000000000 | 12000000000 | 2300000 | 61264.40 | 51.04 | 1.00 | 3.64 | 1.57 |
| 59 | 2018 | 27.56 | 30.13 | 62.00 | 63.00 | 15000000000 | 12000000000 | 1800000 | 68793.80 | 53.60 | 1.00 | 3.64 | 74.00 |
| 59 | 2019 | 23.37 | 29.29 | 60.46 | 57.00 | 16000000000 | 13000000000 | 2100000 | 62088.10 | 52.35 | 1.00 | 3.64 | 9.80 |
| 60 | 2000 | 45.08 | 50.17 | 75.08 | 64.92 | 53000000 | 290000000 | 81000 | 651.31 | 40.78 | 4.00 | 710.21 | 2.50 |
| 60 | 2001 | 42.65 | 45.25 | 72.83 | 67.21 | 58000000 | 290000000 | 94000 | 664.13 | 40.70 | 4.00 | 732.40 | 30.57 |
| 60 | 2002 | 42.44 | 46.88 | 72.25 | 65.75 | 56000000 | 490000000 | 107000 | 716.53 | 47.46 | 4.00 | 693.71 | 0.22 |
| 60 | 2003 | 44.9 | 54.17 | 69.00 | 66.63 | 76000000 | 550000000 | 120000 | 869.71 | 41.89 | 4.00 | 579.90 | 23.39 |
| 60 | 2004 | 43.56 | 56.54 | 65.46 | 65.13 | 91000000 | 570000000 | 133000 | 921.20 | 46.41 | 4.00 | 527.34 | 33.10 |
| 60 | 2005 | 45.44 | 60.42 | 64.21 | 66.25 | 93000000 | 550000000 | 146000 | 930.83 | 49.90 | 4.00 | 527.26 | 1.56 |
| 60 | 2006 | 43.15 | 57.21 | 65.17 | 63.92 | 100000000 | 580000000 | 159000 | 949.13 | 52.37 | 4.00 | 522.43 | 21.71 |
| 60 | 2007 | 41.87 | 56.67 | 64.04 | 63.04 | 120000000 | 610000000 | 182000 | 1061.15 | 47.23 | 4.00 | 478.63 | 5.33 |
| 60 | 2008 | 40.77 | 55.79 | 64.25 | 61.50 | 130000000 | 610000000 | 205000 | 1235.61 | 47.12 | 4.00 | 446.00 | 8.43 |
| 60 | 2009 | 42.15 | 56.25 | 63.04 | 65.00 | 160000000 | 590000000 | 231000 | 1210.30 | 50.85 | 4.00 | 470.29 | 5.30 |
| 60 | 2010 | 41.77 | 57.58 | 62.21 | 63.75 | 210000000 | 570000000 | 252000 | 1213.11 | 50.63 | 4.00 | 494.79 | 0.70 |
| 60 | 2011 | 41.85 | 55.13 | 61.54 | 67.04 | 190000000 | 580000000 | 270000 | 1208.58 | 53.82 | 4.00 | 471.25 | 8.00 |
| 60 | 2012 | 38.87 | 51.58 | 62.21 | 63.96 | 170000000 | 560000000 | 289000 | 1243.27 | 48.93 | 5.00 | 510.56 | 90.57 |
| 60 | 2013 | 39.12 | 53.17 | 60.75 | 64.33 | 190000000 | 580000000 | 380000 | 1415.87 | 41.53 | 5.00 | 493.90 | 44.13 |
| 60 | 2014 | 38.27 | 52.42 | 60.58 | 63.54 | 200000000 | 630000000 | 471000 | 1561.46 | 39.27 | 5.00 | 493.76 | 75.40 |
| 60 | 2015 | 38.04 | 49.96 | 62.46 | 63.67 | 210000000 | 560000000 | 1400000 | 1972.55 | 27.36 | 5.00 | 591.21 | 90.00 |
| 60 | 2016 | 36.92 | 50.00 | 60.33 | 63.50 | 480000000 | 490000000 | 1600000 | 2013.38 | 24.60 | 5.00 | 592.61 | 79.20 |
| 60 | 2017 | 37.25 | 50.46 | 60.13 | 63.92 | 510000000 | 560000000 | 1800000 | 2111.03 | 24.92 | 5.00 | 580.66 | 10.08 |
| 60 | 2018 | 37.1 | 50.08 | 60.25 | 63.88 | 570000000 | 630000000 | 2000000 | 2302.61 | 22.60 | 5.00 | 555.45 | 35.60 |
| 60 | 2019 | 39.06 | 50.63 | 64.25 | 63.25 | 620000000 | 690000000 | 2100000 | 2276.33 | 23.78 | 5.00 | 530.24 | 0.25 |
| 61 | 2000 | 19.6 | 32.00 | 52.00 | 55.21 | 390000000 | 2900000000 | 1900000 | 18440.40 | 56.47 | 4.00 | 0.31 | 7.12 |
| 61 | 2001 | 16.5 | 26.54 | 51.63 | 54.83 | 290000000 | 3200000000 | 2100000 | 16587.20 | 51.31 | 4.00 | 0.31 | 42.31 |
| 61 | 2002 | 17.81 | 26.88 | 51.92 | 56.83 | 320000000 | 3400000000 | 2300000 | 17846.30 | 44.62 | 4.00 | 0.30 | 36.00 |
| 61 | 2003 | 15.54 | 24.67 | 52.54 | 53.88 | 330000000 | 3800000000 | 2600000 | 22148.40 | 52.09 | 4.00 | 0.30 | 5.80 |
| 61 | 2004 | 14.87 | 22.50 | 53.88 | 53.38 | 400000000 | 4100000000 | 3100000 | 27011.70 | 56.92 | 4.00 | 0.29 | 66.05 |
| 61 | 2005 | 14.87 | 23.04 | 54.21 | 52.50 | 410000000 | 5000000000 | 3500000 | 35591.00 | 63.98 | 4.00 | 0.29 | 58.00 |
| 61 | 2006 | 14.06 | 22.96 | 53.67 | 51.50 | 510000000 | 6100000000 | 3900000 | 42781.40 | 65.55 | 4.00 | 0.29 | 12.30 |
| 61 | 2007 | 13.73 | 21.58 | 53.79 | 52.08 | 530000000 | 7300000000 | 4500000 | 45782.30 | 63.42 | 4.00 | 0.28 | 21.06 |
| 61 | 2008 | 14.37 | 22.38 | 54.75 | 51.63 | 610000000 | 8300000000 | 4700000 | 55494.90 | 66.76 | 4.00 | 0.27 | 18.20 |
| 61 | 2009 | 20.35 | 25.71 | 56.58 | 58.42 | 660000000 | 6800000000 | 5100000 | 37561.70 | 59.43 | 4.00 | 0.29 | 1.90 |
| 61 | 2010 | 17.92 | 27.00 | 54.42 | 54.42 | 570000000 | 7100000000 | 5200000 | 38577.50 | 66.67 | 4.00 | 0.29 | 34.80 |
| 61 | 2011 | 19.02 | 30.29 | 54.63 | 53.13 | 640000000 | 8900000000 | 5600000 | 48631.70 | 73.20 | 4.00 | 0.28 | 27.53 |
| 61 | 2012 | 19.79 | 33.42 | 54.33 | 51.83 | 780000000 | 10000000000 | 5700000 | 51979.10 | 74.73 | 5.00 | 0.28 | 34.37 |
| 61 | 2013 | 19.5 | 33.25 | 53.00 | 52.75 | 620000000 | 11000000000 | 6200000 | 49388.10 | 70.86 | 5.00 | 0.28 | 8.30 |
| 61 | 2014 | 18.58 | 31.58 | 52.58 | 53.00 | 620000000 | 12000000000 | 6500000 | 44062.30 | 68.51 | 5.00 | 0.28 | 25.88 |
| 61 | 2015 | 20.58 | 32.25 | 53.71 | 55.21 | 930000000 | 13000000000 | 6900000 | 29869.50 | 53.77 | 5.00 | 0.30 | 46.00 |
| 61 | 2016 | 28.25 | 33.83 | 57.17 | 65.50 | 830000000 | 13000000000 | 7100000 | 27653.10 | 47.63 | 5.00 | 0.30 | 7.90 |
| 61 | 2017 | 26.12 | 33.17 | 56.50 | 62.58 | 640000000 | 14000000000 | 7400000 | 29759.40 | 51.20 | 5.00 | 0.30 | 11.00 |
| 61 | 2018 | 23.52 | 33.08 | 56.04 | 57.92 | 920000000 | 14000000000 | 8500000 | 33994.40 | 56.72 | 5.00 | 0.30 | 39.30 |
| 61 | 2019 | 22.52 | 31.25 | 56.46 | 57.33 | 1200000000 | 17000000000 | 8600000 | 32000.40 | 62.25 | 5.00 | 0.30 | 59.60 |
| 62 | 2000 | 30.58 | 32.08 | 64.71 | 64.38 | 2900000000 | 630000000 | 5800000 | 4849.53 | 35.25 | 8.00 | 8.28 | 38.20 |
| 62 | 2001 | 25.73 | 25.71 | 63.38 | 62.38 | 3500000000 | 680000000 | 6500000 | 5391.09 | 37.24 | 8.00 | 8.34 | 66.70 |
| 62 | 2002 | 26.56 | 25.88 | 63.38 | 63.88 | 4000000000 | 850000000 | 6900000 | 6233.05 | 36.21 | 8.00 | 7.87 | 69.00 |
| 62 | 2003 | 27.83 | 27.04 | 63.63 | 65.00 | 6500000000 | 710000000 | 7400000 | 8060.26 | 35.89 | 8.00 | 6.70 | 34.51 |
| 62 | 2004 | 26.79 | 25.88 | 63.29 | 64.42 | 6900000000 | 880000000 | 7900000 | 9662.78 | 36.90 | 8.00 | 6.03 | 72.03 |
| 62 | 2005 | 24.94 | 24.75 | 62.13 | 63.00 | 7600000000 | 790000000 | 7700000 | 10530.20 | 36.73 | 8.00 | 5.95 | 61.00 |
| 62 | 2006 | 24.85 | 24.50 | 62.21 | 63.00 | 8300000000 | 770000000 | 8000000 | 11703.30 | 37.92 | 8.00 | 5.84 | 14.00 |
| 62 | 2007 | 26.96 | 24.75 | 65.79 | 63.38 | 9600000000 | 1000000000 | 8600000 | 13962.80 | 37.83 | 9.00 | 5.36 | 23.60 |
| 62 | 2008 | 27.33 | 25.96 | 66.13 | 62.58 | 12000000000 | 1200000000 | 8700000 | 16309.40 | 36.40 | 10.00 | 4.94 | 6.21 |
| 62 | 2009 | 29.56 | 25.42 | 66.58 | 67.13 | 9300000000 | 1000000000 | 8700000 | 14556.00 | 32.66 | 10.00 | 5.28 | 6.89 |
| 62 | 2010 | 30.37 | 24.63 | 66.67 | 69.46 | 8300000000 | 860000000 | 9100000 | 13923.60 | 36.18 | 10.00 | 5.50 | 1.00 |
| 62 | 2011 | 29.85 | 25.63 | 67.08 | 67.00 | 9600000000 | 920000000 | 9900000 | 14566.60 | 38.86 | 11.00 | 5.34 | 12.02 |
| 62 | 2012 | 31.81 | 27.21 | 69.75 | 66.67 | 8900000000 | 960000000 | 10000000 | 13238.10 | 39.53 | 11.00 | 5.85 | 59.39 |
| 62 | 2013 | 32.54 | 30.33 | 68.67 | 66.08 | 9700000000 | 920000000 | 11000000 | 13642.20 | 40.43 | 11.00 | 5.70 | 44.53 |
| 62 | 2014 | 31.19 | 31.13 | 64.79 | 66.46 | 10000000000 | 870000000 | 12000000 | 13600.20 | 43.30 | 11.00 | 5.75 | 4.38 |
| 62 | 2015 | 31.67 | 30.63 | 67.13 | 65.58 | 8200000000 | 770000000 | 13000000 | 11782.90 | 46.38 | 11.00 | 6.86 | 7.92 |
| 62 | 2016 | 28.48 | 30.63 | 63.17 | 63.17 | 9200000000 | 950000000 | 14000000 | 12360.50 | 47.63 | 12.00 | 6.81 | 78.39 |
| 62 | 2017 | 28.1 | 30.13 | 63.58 | 62.50 | 11000000000 | 1400000000 | 16000000 | 13412.30 | 50.03 | 14.00 | 6.62 | 23.00 |
| 62 | 2018 | 24.35 | 28.46 | 62.25 | 58.00 | 11000000000 | 1700000000 | 17000000 | 14920.20 | 50.50 | 14.00 | 6.28 | 23.92 |
| 62 | 2019 | 23.6 | 26.75 | 61.83 | 58.63 | 12000000000 | 1800000000 | 60000000 | 14936.10 | 51.96 | 14.00 | 5.93 | 5.55 |
| 63 | 2000 | 40.96 | 49.08 | 65.25 | 67.58 | 500000000 | 160000000 | 899000 | 397.48 | 21.59 | 2.00 | 76.18 | 37.30 |
| 63 | 2001 | 38.6 | 44.42 | 64.63 | 68.17 | 540000000 | 180000000 | 841000 | 395.33 | 22.93 | 4.00 | 78.56 | 22.60 |
| 63 | 2002 | 40.17 | 48.29 | 65.00 | 67.04 | 510000000 | 170000000 | 838000 | 389.54 | 24.90 | 4.00 | 78.75 | 41.20 |
| 63 | 2003 | 34.94 | 39.00 | 63.42 | 67.46 | 620000000 | 180000000 | 927000 | 429.79 | 24.09 | 4.00 | 75.94 | 11.23 |
| 63 | 2004 | 33.4 | 38.83 | 60.83 | 67.13 | 800000000 | 180000000 | 1200000 | 451.67 | 26.61 | 4.00 | 79.17 | 34.00 |
| 63 | 2005 | 34.83 | 42.54 | 60.25 | 66.88 | 970000000 | 190000000 | 1400000 | 511.62 | 28.51 | 4.00 | 75.55 | 64.00 |
| 63 | 2006 | 36 | 43.50 | 61.79 | 66.71 | 1200000000 | 200000000 | 1500000 | 685.96 | 22.98 | 4.00 | 72.10 | 26.80 |
| 63 | 2007 | 35.65 | 43.31 | 61.29 | 66.71 | 1500000000 | 200000000 | 1700000 | 825.67 | 21.92 | 4.00 | 67.32 | 27.40 |
| 63 | 2008 | 37.31 | 44.21 | 61.29 | 69.13 | 1400000000 | 210000000 | 1100000 | 902.07 | 22.67 | 5.00 | 69.18 | 20.00 |
| 63 | 2009 | 38.04 | 43.96 | 62.21 | 69.92 | 1100000000 | 220000000 | 1400000 | 905.13 | 20.03 | 5.00 | 77.35 | 0.54 |
| 63 | 2010 | 36.12 | 43.17 | 60.50 | 68.58 | 1600000000 | 220000000 | 1500000 | 951.69 | 20.66 | 5.00 | 79.23 | 67.38 |
| 63 | 2011 | 37.62 | 44.88 | 61.21 | 69.17 | 1800000000 | 230000000 | 1800000 | 971.63 | 21.63 | 7.00 | 88.81 | 72.50 |
| 63 | 2012 | 38.87 | 46.58 | 61.21 | 69.96 | 2000000000 | 240000000 | 1600000 | 1136.87 | 22.23 | 7.00 | 84.53 | 2.30 |
| 63 | 2013 | 37.46 | 44.46 | 61.54 | 68.92 | 1800000000 | 240000000 | 1400000 | 1210.39 | 19.93 | 8.00 | 86.12 | 73.45 |
| 63 | 2014 | 37.35 | 44.58 | 61.54 | 68.58 | 1700000000 | 250000000 | 1300000 | 1315.80 | 18.30 | 8.00 | 87.92 | 74.25 |
| 63 | 2015 | 37.15 | 43.50 | 61.92 | 68.88 | 1600000000 | 260000000 | 1100000 | 1336.88 | 16.57 | 8.00 | 98.18 | 1.61 |
| 63 | 2016 | 37.25 | 42.79 | 62.63 | 69.08 | 1500000000 | 260000000 | 1300000 | 1410.53 | 14.33 | 8.00 | 101.50 | 33.88 |
| 63 | 2017 | 37.54 | 43.33 | 62.75 | 69.00 | 1600000000 | 270000000 | 1400000 | 1572.34 | 13.23 | 8.00 | 103.41 | 7.50 |
| 63 | 2018 | 36.19 | 42.38 | 62.25 | 67.75 | 1800000000 | 280000000 | 1500000 | 1707.99 | 13.17 | 9.00 | 101.30 | 18.90 |
| 63 | 2019 | 35.54 | 40.83 | 63.00 | 67.25 | 1800000000 | 280000000 | 2000000 | 1816.55 | 12.03 | 9.00 | 99.19 | 53.00 |
| 64 | 2000 | 28.35 | 31.75 | 61.13 | 63.83 | 170000000 | 280000000 | 509000 | 3351.23 | 36.90 | 1.00 | 0.61 | 39.08 |
| 64 | 2001 | 25.27 | 26.17 | 60.88 | 63.50 | 150000000 | 260000000 | 591000 | 3566.17 | 38.13 | 1.00 | 0.63 | 39.22 |
| 64 | 2002 | 24.44 | 24.88 | 60.50 | 63.50 | 200000000 | 270000000 | 848000 | 4125.21 | 36.64 | 1.00 | 0.62 | 27.43 |
| 64 | 2003 | 22.52 | 22.21 | 60.50 | 62.33 | 270000000 | 370000000 | 971000 | 5127.59 | 36.17 | 1.00 | 0.57 | 45.00 |
| 64 | 2004 | 23.94 | 25.42 | 60.50 | 61.96 | 340000000 | 430000000 | 1100000 | 6343.14 | 39.13 | 1.00 | 0.54 | 70.00 |
| 64 | 2005 | 23.15 | 22.83 | 60.96 | 62.50 | 450000000 | 660000000 | 1100000 | 7552.87 | 43.21 | 2.00 | 0.56 | 93.00 |
| 64 | 2006 | 23.54 | 23.79 | 60.79 | 62.50 | 620000000 | 790000000 | 1500000 | 9663.37 | 39.99 | 2.00 | 0.56 | 58.97 |
| 64 | 2007 | 31.6 | 25.17 | 73.54 | 64.50 | 880000000 | 1000000000 | 1700000 | 14042.50 | 38.46 | 2.00 | 0.51 | 37.74 |
| 64 | 2008 | 30.75 | 25.29 | 69.50 | 66.71 | 1100000000 | 1300000000 | 1700000 | 16377.00 | 39.47 | 2.00 | 0.48 | 6.15 |
| 64 | 2009 | 36.73 | 29.25 | 70.13 | 74.08 | 1400000000 | 1500000000 | 1300000 | 12257.20 | 42.47 | 2.00 | 0.51 | 1.13 |
| 64 | 2010 | 35.27 | 30.88 | 71.79 | 67.88 | 1700000000 | 1700000000 | 1400000 | 11348.40 | 53.57 | 2.00 | 0.53 | 86.84 |
| 64 | 2011 | 31.48 | 28.75 | 68.21 | 66.00 | 1900000000 | 2000000000 | 1500000 | 13832.40 | 57.77 | 2.00 | 0.50 | 79.00 |
| 64 | 2012 | 34.21 | 30.96 | 72.13 | 65.33 | 2200000000 | 2200000000 | 1400000 | 13850.60 | 61.20 | 2.00 | 0.55 | 2.60 |
| 64 | 2013 | 33.46 | 29.92 | 74.00 | 63.00 | 2400000000 | 2400000000 | 1500000 | 15041.20 | 60.26 | 2.00 | 0.53 | 27.72 |
| 64 | 2014 | 30.9 | 27.08 | 74.21 | 60.50 | 2700000000 | 2700000000 | 1800000 | 15740.30 | 61.20 | 2.00 | 0.61 | 29.00 |
| 64 | 2015 | 28.08 | 26.21 | 68.63 | 61.33 | 2900000000 | 2900000000 | 2000000 | 13698.90 | 60.72 | 2.00 | 0.65 | 24.60 |
| 64 | 2016 | 25.71 | 25.88 | 65.29 | 60.25 | 3200000000 | 3100000000 | 1800000 | 14153.40 | 60.40 | 2.00 | 0.74 | 71.58 |
| 64 | 2017 | 26.4 | 26.71 | 65.46 | 60.63 | 3500000000 | 3400000000 | 1900000 | 15586.60 | 62.13 | 2.00 | 0.78 | 9.00 |
| 64 | 2018 | 27.42 | 28.50 | 65.25 | 61.08 | 3700000000 | 3600000000 | 1900000 | 17805.30 | 61.50 | 2.00 | 0.75 | 82.49 |
| 64 | 2019 | 26.1 | 26.46 | 65.25 | 60.50 | 4000000000 | 3800000000 | 8300000 | 17828.90 | 60.13 | 2.00 | 0.72 | 7.63 |
| 65 | 2000 | 40.29 | 38.67 | 69.92 | 72.00 | 740000000 | 2100000000 | 742000 | 4491.64 | 14.18 | 5.00 | 1507.50 | 4.50 |
| 65 | 2001 | 41.52 | 36.67 | 69.88 | 76.50 | 840000000 | 2500000000 | 837000 | 4422.39 | 15.60 | 5.00 | 1507.50 | 0.56 |
| 65 | 2002 | 44.19 | 40.00 | 73.54 | 74.83 | 3800000000 | 2900000000 | 956000 | 4579.46 | 16.10 | 5.00 | 1507.50 | 30.00 |
| 65 | 2003 | 44.79 | 40.58 | 75.00 | 74.00 | 6800000000 | 3300000000 | 1000000 | 4576.39 | 16.74 | 5.00 | 1507.50 | 34.33 |
| 65 | 2004 | 42.87 | 39.46 | 72.29 | 74.00 | 5900000000 | 3700000000 | 1300000 | 4630.79 | 35.05 | 5.00 | 1507.50 | 14.33 |
| 65 | 2005 | 41.92 | 41.29 | 68.50 | 74.04 | 6000000000 | 3600000000 | 1100000 | 4575.11 | 36.62 | 5.00 | 1507.50 | 23.90 |
| 65 | 2006 | 42.54 | 42.29 | 68.50 | 74.29 | 5500000000 | 3800000000 | 1100000 | 4626.86 | 35.59 | 5.00 | 1507.50 | 18.20 |
| 65 | 2007 | 41.62 | 43.75 | 68.50 | 71.00 | 5800000000 | 3900000000 | 1000000 | 5207.80 | 37.14 | 5.00 | 1507.50 | 8.10 |
| 65 | 2008 | 42.17 | 43.50 | 69.96 | 70.88 | 6300000000 | 4300000000 | 1300000 | 6111.33 | 38.55 | 5.00 | 1507.50 | 81.60 |
| 65 | 2009 | 42.02 | 42.92 | 72.08 | 69.04 | 7200000000 | 4900000000 | 1800000 | 7354.95 | 33.20 | 5.00 | 1507.50 | 89.63 |
| 65 | 2010 | 39.08 | 41.46 | 71.96 | 64.75 | 8000000000 | 4900000000 | 2200000 | 7761.65 | 35.02 | 5.00 | 1507.50 | 35.10 |
| 65 | 2011 | 40.46 | 44.46 | 69.46 | 67.00 | 6800000000 | 4400000000 | 1700000 | 7674.84 | 36.36 | 5.00 | 1507.50 | 9.80 |
| 65 | 2012 | 38.35 | 45.71 | 65.00 | 66.00 | 7400000000 | 4500000000 | 1400000 | 7950.70 | 28.80 | 5.00 | 1507.50 | 0.94 |
| 65 | 2013 | 41.31 | 47.21 | 65.63 | 69.79 | 7000000000 | 4700000000 | 1300000 | 7931.08 | 26.63 | 5.00 | 1507.50 | 2.30 |
| 65 | 2014 | 41.5 | 48.00 | 62.00 | 73.00 | 6800000000 | 5200000000 | 1400000 | 7686.26 | 23.74 | 5.00 | 1507.50 | 80.30 |
| 65 | 2015 | 36.96 | 45.50 | 61.21 | 67.21 | 7100000000 | 5200000000 | 1500000 | 7644.55 | 23.14 | 5.00 | 1507.50 | 5.44 |
| 65 | 2016 | 39.1 | 45.21 | 63.50 | 69.50 | 7400000000 | 5300000000 | 1700000 | 7629.89 | 21.70 | 5.00 | 1507.50 | 9.50 |
| 65 | 2017 | 41.9 | 44.38 | 67.00 | 72.42 | 8100000000 | 5700000000 | 1900000 | 7801.18 | 21.88 | 5.00 | 1507.50 | 64.43 |
| 65 | 2018 | 42.19 | 45.38 | 67.13 | 71.88 | 8700000000 | 6400000000 | 2000000 | 8024.80 | 20.72 | 5.00 | 1507.50 | 4.00 |
| 65 | 2019 | 44.19 | 45.25 | 68.00 | 75.13 | 8700000000 | 6800000000 | 1900000 | 7583.69 | 35.36 | 5.00 | 1507.50 | 3.84 |
| 66 | 2000 | 28.67 | 33.83 | 60.00 | 63.50 | 430000000 | 260000000 | 1100000 | 3297.35 | 38.55 | 2.00 | 4.00 | 53.10 |
| 66 | 2001 | 26 | 28.58 | 60.38 | 63.04 | 430000000 | 230000000 | 1300000 | 3530.15 | 44.04 | 2.00 | 4.00 | 2.00 |
| 66 | 2002 | 25.67 | 26.08 | 62.75 | 62.50 | 560000000 | 330000000 | 1400000 | 4146.99 | 47.34 | 2.00 | 3.68 | 50.80 |
| 66 | 2003 | 23.83 | 21.92 | 63.25 | 62.50 | 700000000 | 480000000 | 1500000 | 5505.54 | 46.17 | 2.00 | 3.06 | 50.58 |
| 66 | 2004 | 24.35 | 24.38 | 62.71 | 61.63 | 830000000 | 640000000 | 1800000 | 6706.97 | 47.35 | 3.00 | 2.78 | 6.10 |
| 66 | 2005 | 23.31 | 22.92 | 62.71 | 61.00 | 970000000 | 560000000 | 2000000 | 7863.16 | 53.84 | 4.00 | 2.77 | 66.84 |
| 66 | 2006 | 24.02 | 24.21 | 62.83 | 61.00 | 1100000000 | 640000000 | 2200000 | 9240.64 | 55.62 | 4.00 | 2.75 | 30.14 |
| 66 | 2007 | 26.56 | 24.50 | 65.79 | 62.83 | 1200000000 | 640000000 | 1500000 | 12297.90 | 50.36 | 4.00 | 2.52 | 59.76 |
| 66 | 2008 | 27.77 | 23.79 | 66.96 | 64.79 | 1400000000 | 730000000 | 1600000 | 14961.60 | 57.14 | 4.00 | 2.36 | 10.80 |
| 66 | 2009 | 30.62 | 24.08 | 69.08 | 68.08 | 1500000000 | 810000000 | 1300000 | 11837.40 | 51.94 | 4.00 | 2.48 | 87.31 |
| 66 | 2010 | 32.4 | 28.50 | 65.71 | 70.58 | 1600000000 | 980000000 | 1500000 | 11957.10 | 64.12 | 4.00 | 2.61 | 36.60 |
| 66 | 2011 | 32.23 | 28.75 | 69.21 | 66.50 | 1800000000 | 1200000000 | 1800000 | 14354.20 | 73.08 | 4.00 | 2.48 | 1.63 |
| 66 | 2012 | 30.48 | 28.83 | 66.88 | 65.25 | 1900000000 | 1600000000 | 1900000 | 14339.30 | 78.41 | 4.00 | 2.69 | 58.86 |
| 66 | 2013 | 26.21 | 26.46 | 62.38 | 63.58 | 2000000000 | 2300000000 | 2000000 | 15702.10 | 78.79 | 4.00 | 2.60 | 1.07 |
| 66 | 2014 | 24.54 | 25.88 | 61.71 | 61.50 | 2200000000 | 3400000000 | 2100000 | 16548.40 | 72.34 | 4.00 | 2.60 | 55.90 |
| 66 | 2015 | 25.19 | 25.00 | 64.38 | 61.00 | 2300000000 | 5200000000 | 2100000 | 14249.10 | 68.82 | 4.00 | 0.65 | 1.80 |
| 66 | 2016 | 24.71 | 25.46 | 62.54 | 61.42 | 2400000000 | 8000000000 | 2300000 | 14999.50 | 67.58 | 4.00 | 0.74 | 71.00 |
| 66 | 2017 | 26.31 | 26.54 | 64.29 | 61.79 | 2600000000 | 13000000000 | 2500000 | 16882.60 | 73.61 | 4.00 | 0.78 | 10.00 |
| 66 | 2018 | 26.98 | 27.33 | 66.25 | 60.38 | 2700000000 | 20000000000 | 2800000 | 19080.60 | 75.62 | 4.00 | 0.75 | 3.10 |
| 66 | 2019 | 25.98 | 25.79 | 66.46 | 59.71 | 2800000000 | 32000000000 | 6200000 | 19601.90 | 77.45 | 4.00 | 0.72 | 31.40 |
| 67 | 2000 | 32 | 39.67 | 58.92 | 65.42 | 84000000 | 500000000 | 174000 | 7142.77 | 31.56 | 5.00 | 0.51 | 0.22 |
| 67 | 2001 | 28.64 | 40.71 | 56.58 | 60.00 | 90000000 | 570000000 | 169000 | 6266.49 | 26.54 | 5.00 | 0.61 | 27.50 |
| 67 | 2002 | 30.4 | 38.50 | 63.13 | 59.17 | 200000000 | 650000000 | 135000 | 3703.05 | 44.77 | 5.00 | 1.27 | 41.30 |
| 67 | 2003 | 24.79 | 37.25 | 54.46 | 57.88 | 240000000 | 690000000 | 142000 | 4673.14 | 57.32 | 5.00 | 1.29 | 2.68 |
| 67 | 2004 | 23.15 | 34.71 | 54.75 | 56.83 | 260000000 | 790000000 | 149000 | 5800.60 | 63.76 | 5.00 | 1.30 | 26.34 |
| 67 | 2005 | 20.9 | 34.88 | 52.50 | 54.42 | 300000000 | 920000000 | 156000 | 8163.01 | 63.72 | 5.00 | 1.31 | 6.50 |
| 67 | 2006 | 18.83 | 32.54 | 51.88 | 53.25 | 240000000 | 920000000 | 163000 | 9336.36 | 73.56 | 5.00 | 1.31 | 10.00 |
| 67 | 2007 | 18.92 | 32.46 | 51.88 | 53.50 | 99000000 | 1000000000 | 170000 | 11300.20 | 72.70 | 5.00 | 1.26 | 7.30 |
| 67 | 2008 | 18.37 | 31.58 | 51.17 | 54.00 | 99000000 | 1300000000 | 177000 | 14382.60 | 72.51 | 5.00 | 1.22 | 0.76 |
| 67 | 2009 | 25.17 | 32.13 | 53.46 | 64.75 | 160000000 | 1700000000 | 184000 | 10275.30 | 59.24 | 5.00 | 1.25 | 9.30 |
| 67 | 2010 | 20.19 | 32.63 | 51.33 | 56.42 | 170000000 | 2200000000 | 191000 | 12064.80 | 65.61 | 5.00 | 1.27 | 92.08 |
| 67 | 2011 | 28.79 | 43.63 | 53.54 | 60.42 | 180000000 | 2400000000 | 198000 | 5554.18 | 54.83 | 5.00 | 1.22 | 48.27 |
| 67 | 2012 | 24.67 | 40.71 | 54.17 | 54.46 | 190000000 | 2700000000 | 205000 | 13025.30 | 74.62 | 5.00 | 1.26 | 78.00 |
| 67 | 2013 | 27.21 | 45.63 | 55.00 | 53.79 | 200000000 | 2600000000 | 212000 | 10363.80 | 70.43 | 5.00 | 1.27 | 91.00 |
| 67 | 2014 | 39.29 | 48.29 | 56.00 | 74.29 | 210000000 | 1300000000 | 219000 | 6466.91 | 47.01 | 5.00 | 1.27 | 81.30 |
| 67 | 2015 | 46.04 | 47.96 | 61.21 | 82.92 | 230000000 | 1100000000 | 226000 | 4337.92 | 39.97 | 5.00 | 1.38 | 12.11 |
| 67 | 2016 | 49.15 | 47.46 | 62.71 | 88.13 | 240000000 | 810000000 | 233000 | 4035.19 | 26.14 | 5.00 | 1.39 | 38.10 |
| 67 | 2017 | 45.4 | 46.71 | 60.42 | 83.67 | 250000000 | 2000000000 | 240000 | 5756.70 | 49.94 | 5.00 | 1.39 | 0.26 |
| 67 | 2018 | 33.94 | 46.50 | 53.38 | 68.00 | 260000000 | 2000000000 | 247000 | 7877.12 | 56.95 | 5.00 | 1.36 | 7.50 |
| 67 | 2019 | 32.33 | 46.83 | 52.58 | 65.25 | 270000000 | 2100000000 | 254000 | 7685.95 | 64.37 | 5.00 | 1.34 | 49.81 |
| 68 | 2000 | 10.81 | 8.58 | 59.63 | 53.42 | 2200000000 | 1900000000 | 852000 | 48736.00 | 148.49 | 1.00 | 0.66 | 38.00 |
| 68 | 2001 | 8.9 | 5.83 | 57.46 | 54.50 | 2800000000 | 2200000000 | 836000 | 48179.40 | 147.44 | 1.00 | 0.69 | 8.78 |
| 68 | 2002 | 8.5 | 5.33 | 56.79 | 54.88 | 2500000000 | 1900000000 | 885000 | 52930.60 | 141.97 | 1.00 | 0.67 | 70.00 |
| 68 | 2003 | 9.12 | 5.50 | 57.25 | 55.50 | 3100000000 | 2400000000 | 867000 | 65445.90 | 138.90 | 1.00 | 0.61 | 64.00 |
| 68 | 2004 | 9.81 | 6.88 | 56.96 | 55.79 | 3800000000 | 2900000000 | 878000 | 75716.40 | 153.04 | 1.00 | 0.55 | 15.94 |
| 68 | 2005 | 10.1 | 7.00 | 57.21 | 56.00 | 3800000000 | 3000000000 | 913000 | 80289.70 | 161.68 | 1.00 | 0.55 | 31.36 |
| 68 | 2006 | 10.17 | 7.00 | 57.33 | 56.00 | 3800000000 | 3100000000 | 908000 | 89739.70 | 176.25 | 1.00 | 0.54 | 20.10 |
| 68 | 2007 | 10.5 | 7.00 | 57.54 | 56.46 | 4400000000 | 3500000000 | 914000 | 106018.00 | 182.89 | 1.00 | 0.50 | 2.40 |
| 68 | 2008 | 10.56 | 7.25 | 58.00 | 55.88 | 4900000000 | 3800000000 | 877000 | 114294.00 | 187.11 | 1.00 | 0.54 | 44.30 |
| 68 | 2009 | 12.06 | 8.58 | 58.92 | 56.63 | 4500000000 | 3700000000 | 847000 | 103199.00 | 164.07 | 1.00 | 0.64 | 34.07 |
| 68 | 2010 | 13.79 | 8.50 | 58.08 | 61.00 | 4500000000 | 3600000000 | 805000 | 104965.00 | 174.52 | 1.00 | 0.65 | 36.40 |
| 68 | 2011 | 12.44 | 8.50 | 57.08 | 59.29 | 5400000000 | 3900000000 | 874000 | 115762.00 | 177.99 | 1.00 | 0.62 | 9.30 |
| 68 | 2012 | 12.02 | 10.17 | 57.38 | 56.50 | 5400000000 | 3100000000 | 950000 | 106749.00 | 186.44 | 1.00 | 0.63 | 32.70 |
| 68 | 2013 | 12.71 | 11.17 | 56.75 | 57.50 | 5600000000 | 3300000000 | 945000 | 113625.00 | 190.63 | 1.00 | 0.64 | 49.00 |
| 68 | 2014 | 12.81 | 11.42 | 57.21 | 57.00 | 6000000000 | 3400000000 | 1000000 | 118824.00 | 212.61 | 1.00 | 0.61 | 9.78 |
| 68 | 2015 | 14.35 | 12.25 | 60.29 | 56.17 | 6200000000 | 2300000000 | 1100000 | 101377.00 | 221.20 | 1.00 | 0.65 | 17.90 |
| 68 | 2016 | 13.17 | 12.79 | 57.79 | 55.75 | 6200000000 | 2300000000 | 1100000 | 104278.00 | 213.04 | 1.00 | 0.74 | 41.80 |
| 68 | 2017 | 13.27 | 13.46 | 58.08 | 55.00 | 6200000000 | 2900000000 | 1000000 | 107627.00 | 217.62 | 1.00 | 0.78 | 62.40 |
| 68 | 2018 | 13.29 | 13.33 | 58.25 | 55.00 | 6700000000 | 3400000000 | 1000000 | 116654.00 | 211.56 | 1.00 | 0.75 | 42.40 |
| 68 | 2019 | 12.52 | 11.38 | 58.25 | 55.42 | 6300000000 | 3700000000 | 41000000 | 4405.49 | 36.36 | 1.00 | 0.72 | 69.40 |
| 69 | 2000 | 39.65 | 35.75 | 68.75 | 74.79 | 390000000 | 450000000 | 5300000 | 1659.91 | 21.59 | 8.00 | 2.17 | 69.00 |
| 69 | 2001 | 35.37 | 31.75 | 67.96 | 71.04 | 420000000 | 480000000 | 4900000 | 1825.18 | 22.11 | 8.00 | 2.91 | 33.99 |
| 69 | 2002 | 32.33 | 31.25 | 64.79 | 68.63 | 400000000 | 450000000 | 4800000 | 2119.88 | 24.01 | 8.00 | 3.31 | 79.70 |
| 69 | 2003 | 29.17 | 28.92 | 61.54 | 67.88 | 520000000 | 570000000 | 5600000 | 2679.41 | 24.21 | 8.00 | 3.32 | 62.00 |
| 69 | 2004 | 27.96 | 28.33 | 61.08 | 66.50 | 610000000 | 670000000 | 6600000 | 3494.94 | 25.74 | 8.00 | 3.26 | 17.30 |
| 69 | 2005 | 28.27 | 29.79 | 61.04 | 65.71 | 1300000000 | 1100000000 | 5800000 | 4617.93 | 24.54 | 8.00 | 2.91 | 24.30 |
| 69 | 2006 | 29.44 | 30.75 | 62.50 | 65.63 | 1700000000 | 1500000000 | 6000000 | 5757.50 | 24.77 | 8.00 | 2.81 | 15.30 |
| 69 | 2007 | 31 | 30.92 | 64.54 | 66.54 | 2100000000 | 1700000000 | 7700000 | 8360.17 | 24.71 | 9.00 | 2.44 | 9.96 |
| 69 | 2008 | 34.87 | 30.88 | 67.63 | 71.25 | 2600000000 | 2400000000 | 8900000 | 10435.00 | 26.16 | 9.00 | 2.52 | 1.06 |
| 69 | 2009 | 35.56 | 30.96 | 67.33 | 72.83 | 1700000000 | 1800000000 | 7600000 | 8548.12 | 26.02 | 9.00 | 3.05 | 13.80 |
| 69 | 2010 | 33.87 | 31.46 | 65.33 | 70.96 | 1600000000 | 1900000000 | 7500000 | 8209.92 | 32.40 | 10.00 | 3.18 | 63.12 |
| 69 | 2011 | 33.75 | 33.17 | 65.42 | 68.92 | 2000000000 | 2300000000 | 7600000 | 9105.00 | 37.03 | 11.00 | 3.05 | 48.83 |
| 69 | 2012 | 33.87 | 35.17 | 65.88 | 66.71 | 1900000000 | 2100000000 | 7900000 | 8535.05 | 37.41 | 11.00 | 3.47 | 5.12 |
| 69 | 2013 | 31.37 | 34.08 | 63.04 | 65.63 | 2300000000 | 2100000000 | 8000000 | 9555.24 | 39.87 | 11.00 | 3.33 | 6.92 |
| 69 | 2014 | 29.79 | 34.54 | 62.50 | 62.54 | 2400000000 | 2700000000 | 8400000 | 10027.00 | 41.16 | 11.00 | 3.35 | 83.56 |
| 69 | 2015 | 30 | 33.54 | 64.96 | 61.50 | 2200000000 | 2500000000 | 9300000 | 8977.44 | 41.02 | 11.00 | 4.01 | 26.00 |
| 69 | 2016 | 28.27 | 32.79 | 61.54 | 62.21 | 2500000000 | 2900000000 | 10000000 | 9567.13 | 41.19 | 11.00 | 4.06 | 26.55 |
| 69 | 2017 | 28.5 | 33.92 | 60.92 | 62.17 | 3600000000 | 5000000000 | 11000000 | 10807.80 | 41.47 | 12.00 | 4.05 | 6.31 |
| 69 | 2018 | 28.98 | 34.25 | 60.58 | 63.13 | 3900000000 | 6100000000 | 12000000 | 12408.60 | 41.59 | 12.00 | 3.94 | 41.56 |
| 69 | 2019 | 29.83 | 34.00 | 61.71 | 63.96 | 4200000000 | 7200000000 | 1000000 | 114705.00 | 208.75 | 12.00 | 0.72 | 28.90 |
| 70 | 2000 | 36.9 | 36.75 | 69.17 | 67.88 | 150000000 | 140000000 | 160000 | 293.61 | 19.12 | 1.00 | 1353.50 | 45.00 |
| 70 | 2001 | 33.47 | 32.71 | 66.25 | 68.00 | 150000000 | 180000000 | 170000 | 334.44 | 20.07 | 2.00 | 1317.70 | 12.50 |
| 70 | 2002 | 39.73 | 42.83 | 66.96 | 69.67 | 110000000 | 190000000 | 62000 | 319.22 | 21.82 | 2.00 | 1366.39 | 45.00 |
| 70 | 2003 | 39.58 | 39.08 | 68.08 | 72.00 | 120000000 | 67000000 | 139000 | 368.80 | 13.69 | 2.00 | 1238.33 | 68.00 |
| 70 | 2004 | 39.9 | 38.88 | 72.58 | 68.33 | 240000000 | 110000000 | 229000 | 284.49 | 19.46 | 2.00 | 1868.86 | 35.83 |
| 70 | 2005 | 39.4 | 39.58 | 73.21 | 66.00 | 280000000 | 160000000 | 277000 | 319.54 | 24.32 | 2.00 | 2003.03 | 46.00 |
| 70 | 2006 | 38.27 | 38.25 | 68.29 | 70.00 | 380000000 | 170000000 | 312000 | 338.75 | 27.49 | 2.00 | 2142.30 | 21.60 |
| 70 | 2007 | 37.77 | 37.63 | 68.92 | 69.00 | 400000000 | 190000000 | 344000 | 438.66 | 27.80 | 3.00 | 1873.88 | 0.75 |
| 70 | 2008 | 38.37 | 37.50 | 68.58 | 70.67 | 440000000 | 260000000 | 375000 | 536.35 | 27.71 | 3.00 | 1708.37 | 69.85 |
| 70 | 2009 | 40.54 | 41.67 | 67.54 | 71.88 | 330000000 | 230000000 | 163000 | 467.54 | 20.38 | 3.00 | 1956.21 | 74.10 |
| 70 | 2010 | 40.67 | 44.67 | 64.17 | 72.50 | 430000000 | 220000000 | 196000 | 471.96 | 21.87 | 3.00 | 2089.95 | 2.80 |
| 70 | 2011 | 39.46 | 43.13 | 62.13 | 73.67 | 510000000 | 290000000 | 225000 | 531.27 | 22.72 | 3.00 | 2025.12 | 75.17 |
| 70 | 2012 | 38.27 | 44.88 | 61.17 | 70.50 | 600000000 | 240000000 | 256000 | 518.15 | 21.78 | 3.00 | 2194.97 | 76.00 |
| 70 | 2013 | 37.46 | 47.54 | 60.88 | 66.50 | 610000000 | 260000000 | 196000 | 541.07 | 23.27 | 3.00 | 2206.91 | 1.28 |
| 70 | 2014 | 42.06 | 44.83 | 68.29 | 71.00 | 740000000 | 320000000 | 222000 | 530.86 | 28.28 | 3.00 | 2414.81 | 43.00 |
| 70 | 2015 | 38.37 | 45.29 | 64.83 | 66.63 | 700000000 | 240000000 | 244000 | 467.24 | 28.39 | 3.00 | 2933.51 | 8.00 |
| 70 | 2016 | 35.83 | 42.38 | 62.29 | 67.00 | 910000000 | 300000000 | 293000 | 475.96 | 29.09 | 3.00 | 3176.54 | 19.80 |
| 70 | 2017 | 35.25 | 41.79 | 61.71 | 67.00 | 850000000 | 320000000 | 255000 | 515.29 | 30.90 | 3.00 | 3116.11 | 55.00 |
| 70 | 2018 | 35.69 | 43.54 | 61.29 | 66.54 | 880000000 | 310000000 | 291000 | 527.50 | 28.68 | 3.00 | 3334.75 | 40.10 |
| 70 | 2019 | 34.87 | 42.08 | 60.67 | 67.00 | 950000000 | 320000000 | 13000000 | 12919.50 | 40.35 | 3.00 | 3.83 | 40.65 |
| 71 | 2000 | 20.79 | 13.75 | 63.04 | 64.79 | 730000000 | 220000000 | 1200000 | 10391.00 | 126.78 | 4.00 | 0.44 | 31.80 |
| 71 | 2001 | 21.7 | 13.88 | 62.79 | 66.75 | 700000000 | 200000000 | 1200000 | 10346.60 | 115.58 | 4.00 | 0.45 | 46.23 |
| 71 | 2002 | 21.31 | 14.13 | 62.50 | 66.00 | 760000000 | 180000000 | 1100000 | 11281.10 | 117.19 | 4.00 | 0.43 | 75.00 |
| 71 | 2003 | 20.5 | 13.46 | 63.00 | 64.54 | 870000000 | 240000000 | 1100000 | 13587.80 | 108.53 | 4.00 | 0.38 | 93.39 |
| 71 | 2004 | 20.02 | 13.25 | 62.38 | 64.42 | 950000000 | 290000000 | 1200000 | 15063.20 | 103.78 | 4.00 | 0.34 | 62.32 |
| 71 | 2005 | 19.62 | 13.50 | 62.21 | 63.54 | 920000000 | 310000000 | 1200000 | 15857.60 | 104.23 | 4.00 | 0.35 | 42.75 |
| 71 | 2006 | 20.48 | 15.17 | 62.29 | 63.50 | 970000000 | 360000000 | 1100000 | 16671.60 | 123.46 | 4.00 | 0.34 | 6.00 |
| 71 | 2007 | 21.96 | 15.58 | 63.00 | 65.33 | 1200000000 | 280000000 | 1200000 | 19375.60 | 129.54 | 4.00 | 0.31 | 2.40 |
| 71 | 2008 | 19.92 | 13.75 | 62.04 | 64.04 | 1400000000 | 210000000 | 1300000 | 21928.70 | 148.48 | 4.00 | 0.54 | 88.72 |
| 71 | 2009 | 20.81 | 14.17 | 62.38 | 65.08 | 1600000000 | 250000000 | 1200000 | 20675.60 | 147.74 | 4.00 | 0.64 | 82.00 |
| 71 | 2010 | 23.37 | 14.67 | 63.08 | 69.00 | 1800000000 | 270000000 | 1300000 | 21107.40 | 153.26 | 4.00 | 0.65 | 3.00 |
| 71 | 2011 | 22.56 | 15.42 | 61.83 | 67.88 | 2100000000 | 290000000 | 1400000 | 22858.60 | 160.41 | 4.00 | 0.62 | 31.40 |
| 71 | 2012 | 24.75 | 20.63 | 65.38 | 63.50 | 2300000000 | 310000000 | 1400000 | 21916.40 | 165.33 | 4.00 | 0.63 | 43.00 |
| 71 | 2013 | 24.85 | 20.25 | 65.96 | 63.50 | 2500000000 | 330000000 | 1600000 | 23837.30 | 156.99 | 4.00 | 0.64 | 29.03 |
| 71 | 2014 | 24.75 | 20.25 | 67.00 | 62.25 | 2700000000 | 350000000 | 1700000 | 26008.80 | 148.87 | 4.00 | 0.61 | 77.28 |
| 71 | 2015 | 24.77 | 19.75 | 69.50 | 60.29 | 2900000000 | 370000000 | 1800000 | 24002.50 | 154.25 | 4.00 | 0.65 | 25.00 |
| 71 | 2016 | 21.75 | 17.92 | 66.54 | 59.04 | 3200000000 | 390000000 | 2000000 | 25133.00 | 152.01 | 4.00 | 0.74 | 86.89 |
| 71 | 2017 | 19.83 | 16.50 | 67.00 | 56.17 | 3400000000 | 410000000 | 2300000 | 27239.10 | 149.72 | 4.00 | 0.78 | 9.20 |
| 71 | 2018 | 19.06 | 16.88 | 67.75 | 53.50 | 3600000000 | 430000000 | 2600000 | 30133.50 | 144.93 | 4.00 | 0.75 | 5.00 |
| 71 | 2019 | 19.81 | 18.88 | 66.75 | 54.00 | 3800000000 | 450000000 | 486000 | 523.36 | 26.60 | 4.00 | 3553.39 | 0.72 |
| 72 | 2000 | 40.21 | 33.42 | 73.63 | 73.38 | 29000000 | 53000000 | 228000 | 156.39 | 25.60 | 1.00 | 59.54 | 36.50 |
| 72 | 2001 | 40.33 | 36.83 | 74.83 | 69.00 | 40000000 | 52000000 | 266000 | 150.15 | 27.99 | 1.00 | 72.20 | 36.50 |
| 72 | 2002 | 43.42 | 43.33 | 71.17 | 72.33 | 45000000 | 86000000 | 383000 | 298.43 | 15.86 | 1.00 | 76.69 | 15.90 |
| 72 | 2003 | 45.81 | 44.50 | 73.13 | 74.00 | 66000000 | 70000000 | 424000 | 267.40 | 20.17 | 1.00 | 97.43 | 29.90 |
| 72 | 2004 | 43.87 | 42.00 | 71.75 | 74.00 | 75000000 | 70000000 | 427000 | 282.57 | 18.85 | 1.00 | 108.90 | 31.60 |
| 72 | 2005 | 43.33 | 42.08 | 70.58 | 74.00 | 48000000 | 84000000 | 438000 | 289.56 | 18.12 | 1.00 | 118.42 | 8.37 |
| 72 | 2006 | 42.27 | 43.67 | 73.38 | 67.50 | 45000000 | 85000000 | 638000 | 308.16 | 17.63 | 2.00 | 136.01 | 83.70 |
| 72 | 2007 | 40.35 | 43.96 | 68.79 | 67.96 | 43000000 | 79000000 | 735000 | 332.26 | 23.31 | 2.00 | 139.96 | 90.72 |
| 72 | 2008 | 38.5 | 44.46 | 65.88 | 66.67 | 43000000 | 86000000 | 742000 | 387.61 | 22.66 | 2.00 | 140.52 | 37.50 |
| 72 | 2009 | 34.92 | 41.71 | 62.88 | 65.25 | 46000000 | 91000000 | 755000 | 438.21 | 20.03 | 2.00 | 141.17 | 11.09 |
| 72 | 2010 | 36.17 | 41.29 | 64.04 | 67.00 | 45000000 | 90000000 | 746000 | 478.67 | 22.79 | 2.00 | 150.49 | 1.00 |
| 72 | 2011 | 36.29 | 43.58 | 62.50 | 66.50 | 36000000 | 92000000 | 767000 | 534.95 | 20.78 | 2.00 | 156.52 | 2.45 |
| 72 | 2012 | 41.31 | 46.17 | 68.29 | 68.17 | 35000000 | 96000000 | 770000 | 391.56 | 26.20 | 2.00 | 249.11 | 80.30 |
| 72 | 2013 | 41.79 | 47.04 | 66.38 | 70.17 | 33000000 | 90000000 | 795000 | 348.43 | 35.66 | 2.00 | 364.41 | 7.80 |
| 72 | 2014 | 39.9 | 48.00 | 62.96 | 68.83 | 36000000 | 110000000 | 819000 | 371.27 | 33.70 | 2.00 | 424.90 | 13.00 |
| 72 | 2015 | 40.56 | 48.29 | 63.67 | 69.17 | 39000000 | 120000000 | 805000 | 380.60 | 29.16 | 2.00 | 499.61 | 68.82 |
| 72 | 2016 | 43.83 | 48.58 | 68.21 | 70.88 | 30000000 | 100000000 | 849000 | 315.78 | 32.97 | 2.00 | 718.01 | 6.40 |
| 72 | 2017 | 41.08 | 46.54 | 64.42 | 71.21 | 35000000 | 120000000 | 837000 | 356.72 | 29.16 | 2.00 | 730.27 | 4.30 |
| 72 | 2018 | 38.9 | 46.29 | 63.50 | 68.00 | 43000000 | 140000000 | 871000 | 381.26 | 30.88 | 2.00 | 732.33 | 69.00 |
| 72 | 2019 | 38.33 | 44.54 | 63.75 | 68.38 | 47000000 | 150000000 | 3500000 | 29820.60 | 143.82 | 2.00 | 0.72 | 2.70 |
| 73 | 2000 | 24.19 | 32.83 | 58.00 | 57.54 | 5900000000 | 2500000000 | 10000000 | 4043.66 | 119.81 | 2.00 | 710.21 | 61.40 |
| 73 | 2001 | 23.83 | 30.75 | 58.17 | 58.75 | 7600000000 | 3400000000 | 13000000 | 3913.43 | 110.40 | 2.00 | 732.40 | 56.55 |
| 73 | 2002 | 23.62 | 28.25 | 58.29 | 60.71 | 8100000000 | 3300000000 | 13000000 | 4165.73 | 108.31 | 2.00 | 693.71 | 7.20 |
| 73 | 2003 | 23.79 | 28.00 | 59.33 | 60.25 | 6800000000 | 3400000000 | 11000000 | 4461.85 | 106.94 | 2.00 | 579.90 | 68.42 |
| 73 | 2004 | 20.33 | 24.46 | 58.33 | 57.88 | 9200000000 | 3800000000 | 16000000 | 4952.21 | 115.37 | 2.00 | 527.34 | 43.68 |
| 73 | 2005 | 20.56 | 23.58 | 58.33 | 59.21 | 10000000000 | 4300000000 | 16000000 | 5587.03 | 112.90 | 2.00 | 527.26 | 62.12 |
| 73 | 2006 | 20.31 | 23.88 | 57.92 | 58.83 | 12000000000 | 5100000000 | 18000000 | 6209.12 | 112.19 | 2.00 | 522.43 | 14.00 |
| 73 | 2007 | 18.98 | 23.38 | 56.75 | 57.83 | 18000000000 | 6600000000 | 21000000 | 7243.46 | 106.17 | 2.00 | 478.63 | 90.62 |
| 73 | 2008 | 20.52 | 26.21 | 56.83 | 58.00 | 19000000000 | 7700000000 | 22000000 | 8474.59 | 99.50 | 3.00 | 446.00 | 39.93 |
| 73 | 2009 | 23.54 | 27.33 | 57.08 | 62.67 | 17000000000 | 7200000000 | 24000000 | 7292.49 | 91.42 | 3.00 | 470.29 | 1.70 |
| 73 | 2010 | 21.73 | 26.67 | 56.42 | 60.38 | 20000000000 | 9300000000 | 25000000 | 9040.57 | 86.93 | 3.00 | 494.79 | 63.00 |
| 73 | 2011 | 22.02 | 27.42 | 56.92 | 59.71 | 21000000000 | 11000000000 | 25000000 | 10399.40 | 85.26 | 3.00 | 471.25 | 2.26 |
| 73 | 2012 | 22.9 | 29.25 | 56.88 | 59.67 | 22000000000 | 13000000000 | 25000000 | 10817.40 | 79.30 | 4.00 | 510.56 | 56.30 |
| 73 | 2013 | 22.17 | 27.25 | 57.63 | 59.46 | 23000000000 | 13000000000 | 26000000 | 10970.10 | 75.63 | 4.00 | 493.90 | 2.00 |
| 73 | 2014 | 21.79 | 26.92 | 56.75 | 59.92 | 24000000000 | 14000000000 | 27000000 | 11319.10 | 73.79 | 4.00 | 493.76 | 71.69 |
| 73 | 2015 | 24.58 | 27.96 | 61.42 | 59.79 | 19000000000 | 12000000000 | 26000000 | 9955.24 | 69.45 | 4.00 | 591.21 | 10.20 |
| 73 | 2016 | 25.94 | 30.38 | 61.25 | 60.25 | 20000000000 | 11000000000 | 27000000 | 9817.74 | 66.78 | 4.00 | 592.61 | 3.70 |
| 73 | 2017 | 25.4 | 30.08 | 60.29 | 60.42 | 20000000000 | 12000000000 | 26000000 | 10254.20 | 70.05 | 4.00 | 580.66 | 34.77 |
| 73 | 2018 | 24.12 | 28.54 | 60.21 | 59.50 | 22000000000 | 13000000000 | 26000000 | 11373.20 | 68.76 | 4.00 | 555.45 | 0.25 |
| 73 | 2019 | 24.04 | 28.92 | 59.67 | 59.50 | 22000000000 | 14000000000 | 26000000 | 411.55 | 31.01 | 4.00 | 734.39 | 32.30 |
| 74 | 2000 | 34.65 | 35.33 | 69.54 | 64.42 | 47000000 | 66000000 | 86000 | 269.87 | 22.48 | 3.00 | 3.80 | 52.00 |
| 74 | 2001 | 39.1 | 36.88 | 68.50 | 72.83 | 91000000 | 65000000 | 89000 | 307.44 | 26.31 | 3.00 | 3.80 | 4.17 |
| 74 | 2002 | 41.73 | 39.33 | 68.13 | 76.00 | 110000000 | 62000000 | 96000 | 334.84 | 28.09 | 3.00 | 3.80 | 31.05 |
| 74 | 2003 | 41.67 | 38.58 | 68.75 | 76.00 | 140000000 | 94000000 | 110000 | 392.53 | 25.77 | 3.00 | 3.80 | 11.60 |
| 74 | 2004 | 41.1 | 40.00 | 68.21 | 74.00 | 140000000 | 130000000 | 113000 | 440.17 | 23.68 | 4.00 | 3.80 | 24.00 |
| 74 | 2005 | 36.35 | 40.50 | 66.71 | 65.50 | 150000000 | 130000000 | 143000 | 489.02 | 22.99 | 4.00 | 3.79 | 10.00 |
| 74 | 2006 | 36.69 | 41.58 | 66.29 | 65.50 | 180000000 | 200000000 | 153000 | 523.04 | 28.43 | 4.00 | 3.67 | 0.83 |
| 74 | 2007 | 35.75 | 41.42 | 66.58 | 63.50 | 230000000 | 200000000 | 164000 | 596.69 | 24.15 | 4.00 | 3.44 | 11.50 |
| 74 | 2008 | 36 | 41.58 | 66.08 | 64.33 | 290000000 | 230000000 | 98000 | 694.28 | 25.14 | 4.00 | 3.34 | 93.39 |
| 74 | 2009 | 36.31 | 41.04 | 64.92 | 66.67 | 200000000 | 190000000 | 77500 | 698.90 | 21.71 | 4.00 | 3.52 | 53.30 |
| 74 | 2010 | 35.35 | 40.88 | 62.17 | 67.67 | 210000000 | 170000000 | 54750 | 710.27 | 22.84 | 4.00 | 3.22 | 78.21 |
| 74 | 2011 | 33.77 | 42.33 | 59.21 | 66.00 | 220000000 | 170000000 | 43375 | 837.60 | 22.73 | 4.00 | 3.06 | 90.00 |
| 74 | 2012 | 36.35 | 46.83 | 59.88 | 66.00 | 140000000 | 160000000 | 32000 | 778.62 | 27.86 | 4.00 | 3.09 | 83.90 |
| 74 | 2013 | 38.1 | 49.38 | 59.92 | 66.92 | 180000000 | 190000000 | 57000 | 805.03 | 24.94 | 4.00 | 3.15 | 15.90 |
| 74 | 2014 | 38.58 | 48.54 | 62.08 | 66.54 | 210000000 | 230000000 | 53000 | 848.30 | 22.55 | 4.00 | 3.27 | 40.90 |
| 74 | 2015 | 39.33 | 48.63 | 64.00 | 66.04 | 190000000 | 210000000 | 15000 | 751.48 | 24.04 | 4.00 | 3.91 | 0.58 |
| 74 | 2016 | 36.5 | 48.25 | 60.13 | 64.63 | 200000000 | 210000000 | 41500 | 780.39 | 23.45 | 4.00 | 4.15 | 8.00 |
| 74 | 2017 | 39.04 | 47.54 | 63.29 | 67.25 | 210000000 | 220000000 | 11000 | 830.56 | 22.20 | 4.00 | 4.30 | 52.99 |
| 74 | 2018 | 39.31 | 49.13 | 63.17 | 66.33 | 230000000 | 260000000 | 14000 | 900.11 | 24.38 | 4.00 | 4.04 | 41.00 |
| 74 | 2019 | 39.48 | 50.71 | 62.75 | 65.50 | 260000000 | 290000000 | 26000000 | 11414.20 | 65.22 | 4.00 | 3.77 | 12.00 |
| 75 | 2000 | 17.33 | 10.00 | 66.67 | 58.00 | 1.2E+11 | 85000000000 | 51000000 | 36334.90 | 10.69 | 22.00 | 1.00 | 75.71 |
| 75 | 2001 | 18.47 | 13.08 | 63.63 | 60.25 | 1.1E+11 | 82000000000 | 47000000 | 37133.20 | 9.68 | 22.00 | 1.00 | 70.00 |
| 75 | 2002 | 22.1 | 20.38 | 64.17 | 59.67 | 97000000000 | 79000000000 | 44000000 | 38023.20 | 9.13 | 22.00 | 1.00 | 16.70 |
| 75 | 2003 | 24 | 19.67 | 66.88 | 61.46 | 95000000000 | 82000000000 | 41000000 | 39496.50 | 9.04 | 22.00 | 1.00 | 31.59 |
| 75 | 2004 | 22.48 | 17.42 | 66.88 | 60.67 | 1.1E+11 | 93000000000 | 46000000 | 41712.80 | 9.64 | 22.00 | 1.00 | 22.40 |
| 75 | 2005 | 24 | 18.25 | 68.08 | 61.67 | 1.2E+11 | 99000000000 | 49000000 | 44114.70 | 10.01 | 22.00 | 1.00 | 2.90 |
| 75 | 2006 | 25.23 | 19.67 | 69.21 | 61.58 | 1.2E+11 | 1.1E+11 | 51000000 | 46298.70 | 10.66 | 22.00 | 1.00 | 48.50 |
| 75 | 2007 | 26.21 | 22.58 | 68.92 | 60.92 | 1.3E+11 | 1.1E+11 | 56000000 | 47976.00 | 11.49 | 22.00 | 1.00 | 36.80 |
| 75 | 2008 | 24.04 | 19.75 | 66.92 | 61.42 | 1.5E+11 | 1.2E+11 | 58000000 | 48382.60 | 12.49 | 22.00 | 1.00 | 39.82 |
| 75 | 2009 | 25.92 | 16.04 | 66.83 | 68.96 | 1.4E+11 | 1.2E+11 | 55000000 | 47100.00 | 10.95 | 22.00 | 1.00 | 10.50 |
| 75 | 2010 | 22.85 | 18.21 | 63.50 | 64.00 | 1.6E+11 | 1.2E+11 | 60000000 | 48467.50 | 12.32 | 23.00 | 1.00 | 37.37 |
| 75 | 2011 | 23.92 | 18.79 | 64.46 | 64.58 | 1.8E+11 | 1.3E+11 | 63000000 | 49886.80 | 13.53 | 23.00 | 1.00 | 53.00 |
| 75 | 2012 | 23.75 | 16.38 | 67.13 | 64.00 | 2E+11 | 1.4E+11 | 67000000 | 51610.60 | 13.53 | 23.00 | 1.00 | 12.50 |
| 75 | 2013 | 24.19 | 18.58 | 67.21 | 62.58 | 2.1E+11 | 1.3E+11 | 72000000 | 53117.70 | 13.54 | 23.00 | 1.00 | 23.30 |
| 75 | 2014 | 23.71 | 20.00 | 67.17 | 60.25 | 2.2E+11 | 1.4E+11 | 75000000 | 55047.70 | 13.54 | 24.00 | 1.00 | 46.40 |
| 75 | 2015 | 22.4 | 16.83 | 67.46 | 60.50 | 2.3E+11 | 1.4E+11 | 78000000 | 56822.50 | 12.44 | 25.00 | 1.00 | 65.80 |
| 75 | 2016 | 20.85 | 15.67 | 65.33 | 60.71 | 2.3E+11 | 1.5E+11 | 76000000 | 57927.50 | 11.87 | 25.00 | 1.00 | 44.40 |
| 75 | 2017 | 22.5 | 16.00 | 67.50 | 61.50 | 2.3E+11 | 1.6E+11 | 77000000 | 59957.70 | 12.09 | 25.00 | 1.00 | 72.00 |
| 75 | 2018 | 22.31 | 15.38 | 67.50 | 61.75 | 2.4E+11 | 1.7E+11 | 80000000 | 62840.00 | 12.23 | 25.00 | 1.00 | 71.00 |
| 75 | 2019 | 22.56 | 16.42 | 66.75 | 61.96 | 2.3E+11 | 1.8E+11 | 217000 | 879.01 | 23.32 | 25.00 | 530.24 | 42.00 |
| 76 | 2000 | 34.65 | 30.17 | 66.67 | 72.46 | 43000000 | 54000000 | 154000 | 474.22 | 54.00 | 0.00 | 1076.67 | 80.46 |
| 76 | 2001 | 33.04 | 27.92 | 66.92 | 71.25 | 49000000 | 59000000 | 187000 | 524.06 | 50.32 | 0.00 | 1097.70 | 65.00 |
| 76 | 2002 | 36 | 28.50 | 68.50 | 75.00 | 140000000 | 130000000 | 229000 | 571.59 | 50.73 | 0.00 | 1110.31 | 20.70 |
| 76 | 2003 | 36.08 | 28.67 | 68.50 | 75.00 | 150000000 | 140000000 | 201000 | 646.19 | 52.35 | 1.00 | 1146.54 | 27.67 |
| 76 | 2004 | 34.21 | 31.00 | 68.04 | 69.38 | 210000000 | 210000000 | 301000 | 797.98 | 60.79 | 2.00 | 1185.30 | 25.00 |
| 76 | 2005 | 33.94 | 32.71 | 65.17 | 70.00 | 200000000 | 140000000 | 339000 | 998.83 | 58.77 | 2.00 | 1205.25 | 12.35 |
| 76 | 2006 | 32.15 | 31.79 | 64.50 | 68.00 | 260000000 | 200000000 | 404000 | 1334.21 | 59.44 | 2.00 | 1179.70 | 2.50 |
| 76 | 2007 | 30.75 | 31.50 | 64.00 | 66.00 | 350000000 | 220000000 | 452000 | 1632.73 | 59.61 | 2.00 | 1170.40 | 15.90 |
| 76 | 2008 | 30.81 | 32.33 | 63.00 | 66.29 | 270000000 | 250000000 | 446000 | 2136.56 | 54.02 | 2.00 | 1165.80 | 67.50 |
| 76 | 2009 | 33.35 | 31.33 | 64.67 | 70.71 | 250000000 | 240000000 | 412000 | 1714.36 | 50.28 | 2.00 | 1437.80 | 53.68 |
| 76 | 2010 | 31.4 | 31.33 | 61.92 | 69.54 | 290000000 | 320000000 | 456000 | 2643.29 | 46.68 | 2.00 | 1357.06 | 7.50 |
| 76 | 2011 | 31.69 | 31.96 | 62.21 | 69.21 | 200000000 | 460000000 | 460000 | 3757.56 | 52.55 | 3.00 | 1265.52 | 10.92 |
| 76 | 2012 | 32.46 | 33.75 | 61.17 | 70.00 | 220000000 | 560000000 | 476000 | 4351.89 | 43.57 | 3.00 | 1357.58 | 85.00 |
| 76 | 2013 | 31.48 | 33.29 | 62.58 | 67.08 | 240000000 | 640000000 | 418000 | 4366.08 | 38.89 | 3.00 | 1523.93 | 27.20 |
| 76 | 2014 | 35.17 | 33.75 | 64.67 | 71.92 | 260000000 | 530000000 | 393000 | 4158.53 | 52.25 | 3.00 | 1817.94 | 30.65 |
| 76 | 2015 | 36.44 | 32.08 | 66.42 | 74.38 | 280000000 | 470000000 | 386000 | 3918.58 | 45.65 | 4.00 | 1970.31 | 10.00 |
| 76 | 2016 | 31.96 | 32.75 | 66.29 | 64.88 | 380000000 | 540000000 | 404000 | 3660.15 | 50.20 | 4.00 | 2140.29 | 45.00 |
| 76 | 2017 | 34.17 | 31.00 | 67.71 | 69.63 | 460000000 | 620000000 | 469000 | 3669.42 | 59.79 | 5.00 | 2439.78 | 34.30 |
| 76 | 2018 | 33.42 | 30.58 | 67.50 | 68.75 | 530000000 | 820000000 | 529000 | 4134.99 | 58.47 | 5.00 | 2472.48 | 49.00 |
| 76 | 2019 | 30.4 | 29.54 | 64.92 | 66.33 | 610000000 | 1000000000 | 170000000 | 65297.50 | 11.73 | 5.00 | 1.00 | 14.90 |
| 77 | 2000 | 37.81 | 45.00 | 64.13 | 66.50 | 59000000 | 470000000 | 199000 | 418.07 | 12.34 | 3.00 | 52.14 | 51.00 |
| 77 | 2001 | 38.12 | 45.63 | 64.33 | 66.29 | 59000000 | 340000000 | 207000 | 415.03 | 13.39 | 3.00 | 55.81 | 78.00 |
| 77 | 2002 | 39.42 | 48.42 | 64.79 | 65.63 | 59000000 | 310000000 | 207000 | 413.08 | 12.41 | 3.00 | 57.89 | 48.00 |
| 77 | 2003 | 38.02 | 51.25 | 60.54 | 64.25 | 59000000 | 390000000 | 245000 | 446.31 | 11.43 | 3.00 | 58.15 | 50.00 |
| 77 | 2004 | 37.65 | 51.67 | 59.13 | 64.50 | 76000000 | 440000000 | 271000 | 475.29 | 11.15 | 3.00 | 59.51 | 25.60 |
| 77 | 2005 | 37.52 | 49.54 | 60.08 | 65.42 | 82000000 | 370000000 | 208000 | 499.46 | 14.39 | 3.00 | 64.33 | 1.10 |
| 77 | 2006 | 37.46 | 49.17 | 60.50 | 65.25 | 79000000 | 480000000 | 200000 | 509.64 | 16.35 | 3.00 | 68.93 | 74.89 |
| 77 | 2007 | 37.58 | 50.67 | 60.00 | 64.50 | 76000000 | 530000000 | 289000 | 558.05 | 17.00 | 3.00 | 68.87 | 76.50 |
| 77 | 2008 | 37.31 | 50.13 | 60.00 | 64.50 | 77000000 | 740000000 | 467000 | 634.99 | 17.66 | 3.00 | 68.60 | 3.10 |
| 77 | 2009 | 35.54 | 46.29 | 59.13 | 65.67 | 77000000 | 650000000 | 267000 | 702.26 | 16.94 | 3.00 | 69.04 | 78.74 |
| 77 | 2010 | 34.62 | 46.13 | 57.50 | 65.63 | 100000000 | 840000000 | 303000 | 781.15 | 16.02 | 3.00 | 69.65 | 79.49 |
| 77 | 2011 | 36.71 | 50.38 | 57.75 | 65.29 | 97000000 | 820000000 | 155000 | 861.76 | 19.92 | 3.00 | 74.15 | 2.00 |
| 77 | 2012 | 38.48 | 51.50 | 59.50 | 65.96 | 110000000 | 890000000 | 125000 | 883.11 | 20.16 | 3.00 | 81.86 | 65.00 |
| 77 | 2013 | 38.15 | 51.83 | 59.04 | 65.42 | 130000000 | 1300000000 | 148000 | 981.84 | 19.54 | 3.00 | 78.10 | 9.00 |
| 77 | 2014 | 36.54 | 50.38 | 57.92 | 64.79 | 150000000 | 780000000 | 125000 | 1118.85 | 18.99 | 3.00 | 77.64 | 24.76 |
| 77 | 2015 | 35.19 | 48.83 | 56.75 | 64.79 | 150000000 | 830000000 | 643000 | 1248.45 | 17.34 | 3.00 | 77.95 | 77.00 |
| 77 | 2016 | 33.67 | 47.25 | 55.17 | 64.92 | 210000000 | 840000000 | 830000 | 1401.62 | 16.65 | 3.00 | 78.47 | 42.70 |
| 77 | 2017 | 34.04 | 48.29 | 55.21 | 64.58 | 350000000 | 1100000000 | 1000000 | 1563.91 | 15.04 | 3.00 | 80.44 | 45.69 |
| 77 | 2018 | 34.73 | 47.25 | 56.46 | 65.75 | 360000000 | 1200000000 | 1200000 | 1698.35 | 14.80 | 3.00 | 83.47 | 39.65 |
| 77 | 2019 | 33.85 | 44.79 | 57.83 | 65.08 | 390000000 | 1400000000 | 637000 | 4339.84 | 60.10 | 3.00 | 2505.19 | 47.98 |
| 78 | 2000 | 32.29 | 39.33 | 62.58 | 62.67 | 860000000 | 640000000 | 800000 | 1955.59 | 16.77 | 12.00 | 3.49 | 81.61 |
| 78 | 2001 | 30.17 | 31.75 | 62.71 | 65.88 | 760000000 | 770000000 | 901000 | 1941.48 | 16.60 | 12.00 | 3.51 | 94.82 |
| 78 | 2002 | 31 | 36.25 | 62.54 | 63.21 | 840000000 | 810000000 | 1100000 | 2021.24 | 17.27 | 12.00 | 3.52 | 61.95 |
| 78 | 2003 | 31.46 | 37.29 | 62.50 | 63.13 | 1000000000 | 850000000 | 1100000 | 2145.64 | 19.01 | 12.00 | 3.48 | 43.89 |
| 78 | 2004 | 30.42 | 35.67 | 61.96 | 63.21 | 1200000000 | 850000000 | 1400000 | 2417.04 | 22.91 | 12.00 | 3.41 | 9.00 |
| 78 | 2005 | 30.71 | 37.17 | 61.58 | 62.67 | 1400000000 | 970000000 | 1600000 | 2729.50 | 26.85 | 12.00 | 3.30 | 3.00 |
| 78 | 2006 | 28.75 | 36.83 | 59.88 | 60.79 | 1800000000 | 1000000000 | 1700000 | 3154.33 | 30.54 | 12.00 | 3.27 | 89.81 |
| 78 | 2007 | 26.69 | 36.13 | 58.33 | 58.92 | 2000000000 | 1200000000 | 1900000 | 3606.07 | 31.52 | 12.00 | 3.13 | 81.27 |
| 78 | 2008 | 27.1 | 37.50 | 57.58 | 59.13 | 2400000000 | 1400000000 | 2100000 | 4220.62 | 29.73 | 12.00 | 2.92 | 3.50 |
| 78 | 2009 | 29.77 | 37.42 | 58.38 | 63.75 | 2400000000 | 1400000000 | 2100000 | 4196.31 | 26.44 | 13.00 | 3.01 | 38.00 |
| 78 | 2010 | 28.54 | 37.83 | 57.38 | 61.88 | 2500000000 | 1600000000 | 2300000 | 5082.35 | 27.83 | 13.00 | 2.83 | 49.00 |
| 78 | 2011 | 27.87 | 37.25 | 56.83 | 61.67 | 2800000000 | 1800000000 | 2600000 | 5869.32 | 30.50 | 13.00 | 2.75 | 31.37 |
| 78 | 2012 | 27.48 | 36.38 | 57.04 | 61.54 | 3100000000 | 1900000000 | 2800000 | 6528.97 | 27.45 | 13.00 | 2.64 | 77.82 |
| 78 | 2013 | 27.15 | 36.25 | 57.00 | 61.04 | 3900000000 | 2100000000 | 3200000 | 6756.75 | 24.81 | 13.00 | 2.70 | 29.00 |
| 78 | 2014 | 28.77 | 38.21 | 58.04 | 61.29 | 3900000000 | 2100000000 | 3200000 | 6672.88 | 22.61 | 14.00 | 2.84 | 88.71 |
| 78 | 2015 | 30.79 | 39.13 | 59.08 | 63.38 | 4100000000 | 2500000000 | 3500000 | 6229.10 | 21.29 | 14.00 | 3.18 | 10.87 |
| 78 | 2016 | 29.06 | 35.63 | 58.50 | 64.00 | 4300000000 | 2700000000 | 3700000 | 6205.00 | 22.60 | 14.00 | 3.38 | 5.60 |
| 78 | 2017 | 28.33 | 35.92 | 57.17 | 63.58 | 4600000000 | 2900000000 | 4000000 | 6710.51 | 24.72 | 14.00 | 3.26 | 1.20 |
| 78 | 2018 | 27.67 | 35.21 | 57.08 | 63.04 | 4500000000 | 3400000000 | 4400000 | 6941.24 | 25.38 | 14.00 | 3.29 | 40.35 |
| 78 | 2019 | 26.44 | 33.88 | 56.92 | 62.08 | 4800000000 | 3700000000 | 323000 | 1855.74 | 15.32 | 14.00 | 86.49 | 39.21 |
| 79 | 2000 | 41.44 | 53.83 | 64.67 | 64.38 | 200000000 | 30000000 | 416000 | 190.61 | 0.54 | 0.00 | 6.52 | 16.02 |
| 79 | 2001 | 39.37 | 52.42 | 63.25 | 63.08 | 130000000 | 32000000 | 475000 | 137.17 | 0.47 | 0.00 | 6.75 | 30.00 |
| 79 | 2002 | 38.19 | 51.38 | 61.50 | 63.50 | 140000000 | 34000000 | 487000 | 142.08 | 0.29 | 0.00 | 6.64 | 50.60 |
| 79 | 2003 | 39.54 | 51.79 | 60.54 | 66.75 | 70000000 | 36000000 | 597000 | 217.39 | 0.21 | 0.00 | 6.14 | 9.00 |
| 79 | 2004 | 41.79 | 53.54 | 60.50 | 69.54 | 97000000 | 32000000 | 657000 | 217.59 | 0.19 | 0.00 | 5.81 | 83.76 |
| 79 | 2005 | 41.15 | 53.50 | 60.50 | 68.29 | 83000000 | 34000000 | 660000 | 244.88 | 0.18 | 0.00 | 5.82 | 91.42 |
| 79 | 2006 | 40.33 | 53.13 | 60.50 | 67.04 | 59000000 | 40000000 | 630000 | 294.16 | 0.16 | 0.00 | 5.84 | 35.61 |
| 79 | 2007 | 42.77 | 53.04 | 64.50 | 68.00 | 97000000 | 39000000 | 716000 | 406.73 | 0.14 | 0.00 | 5.62 | 15.90 |
| 79 | 2008 | 43.1 | 53.42 | 64.17 | 68.63 | 80000000 | 50000000 | 731000 | 638.15 | 0.12 | 0.00 | 5.44 | 2.00 |
| 79 | 2009 | 47.58 | 53.50 | 68.96 | 72.71 | 75000000 | 60000000 | 763000 | 734.45 | 0.10 | 0.00 | 5.58 | 2.67 |
| 79 | 2010 | 47.6 | 53.63 | 69.58 | 72.00 | 91000000 | 96000000 | 792000 | 979.05 | 19.59 | 0.00 | 5.63 | 83.00 |
| 79 | 2011 | 41.87 | 53.83 | 63.21 | 66.71 | 330000000 | 130000000 | 816000 | 1176.24 | 0.10 | 0.00 | 5.44 | 9.00 |
| 79 | 2012 | 38.67 | 47.96 | 61.96 | 67.42 | 550000000 | 270000000 | 1100000 | 1165.79 | 11.50 | 0.00 | 640.65 | 18.00 |
| 79 | 2013 | 36.52 | 46.29 | 60.17 | 66.58 | 960000000 | 130000000 | 2000000 | 1162.33 | 19.64 | 0.00 | 933.57 | 70.49 |
| 79 | 2014 | 36.92 | 48.50 | 58.96 | 66.38 | 1700000000 | 120000000 | 3100000 | 1251.82 | 20.09 | 1.00 | 984.35 | 8.40 |
| 79 | 2015 | 36.75 | 48.25 | 61.08 | 64.17 | 2200000000 | 150000000 | 4700000 | 1287.43 | 22.98 | 1.00 | 1162.62 | 5.00 |
| 79 | 2016 | 37.27 | 46.92 | 61.54 | 66.08 | 2300000000 | 200000000 | 2900000 | 1266.55 | 25.94 | 1.00 | 1234.87 | 69.00 |
| 79 | 2017 | 38.54 | 47.42 | 62.50 | 67.17 | 2000000000 | 140000000 | 3400000 | 1291.54 | 28.36 | 1.00 | 1360.36 | 2.90 |
| 79 | 2018 | 36.48 | 48.21 | 59.08 | 65.67 | 1700000000 | 120000000 | 3600000 | 1418.18 | 30.39 | 1.00 | 1429.81 | 65.77 |
| 79 | 2019 | 36.75 | 48.04 | 59.29 | 66.17 | 2500000000 | 210000000 | 5300000 | 6977.70 | 24.19 | 1.00 | 3.31 | 57.79 |
| 80 | 2000 | 48.42 | 39.08 | 73.04 | 84.71 | 57000000 | 86000000 | 77000 | 440.67 | 49.60 | 0.00 | 12.43 | 8.80 |
| 80 | 2001 | 37.64 | 35.08 | 71.71 | 68.50 | 58000000 | 90000000 | 75000 | 507.56 | 50.06 | 0.00 | 12.87 | 69.75 |
| 80 | 2002 | 35.56 | 32.46 | 70.42 | 68.25 | 72000000 | 110000000 | 73000 | 570.97 | 52.46 | 0.00 | 13.57 | 52.00 |
| 80 | 2003 | 35.81 | 31.83 | 69.29 | 70.50 | 79000000 | 120000000 | 71000 | 682.53 | 53.31 | 0.00 | 13.94 | 63.64 |
| 80 | 2004 | 33.94 | 32.42 | 66.79 | 68.67 | 110000000 | 140000000 | 69000 | 897.45 | 51.19 | 0.00 | 12.33 | 14.00 |
| 80 | 2005 | 33.73 | 36.75 | 63.71 | 67.00 | 140000000 | 170000000 | 67000 | 1034.71 | 51.16 | 1.00 | 12.60 | 90.03 |
| 80 | 2006 | 34.27 | 37.88 | 63.67 | 67.00 | 150000000 | 220000000 | 63000 | 1183.38 | 45.26 | 1.00 | 13.13 | 40.01 |
| 80 | 2007 | 38.25 | 38.00 | 68.75 | 69.75 | 230000000 | 290000000 | 70000 | 1531.69 | 45.58 | 1.00 | 12.14 | 1.90 |
| 80 | 2008 | 39.12 | 38.54 | 69.71 | 70.00 | 290000000 | 360000000 | 73000 | 2111.20 | 40.82 | 1.00 | 10.39 | 68.02 |
| 80 | 2009 | 40.4 | 40.13 | 69.75 | 70.92 | 240000000 | 310000000 | 60000 | 1899.01 | 36.87 | 1.00 | 11.11 | 3.33 |
| 80 | 2010 | 42.15 | 42.08 | 65.75 | 76.46 | 220000000 | 300000000 | 64000 | 2437.53 | 27.83 | 1.00 | 12.37 | 61.00 |
| 80 | 2011 | 38 | 40.50 | 65.00 | 70.50 | 250000000 | 350000000 | 75000 | 2942.26 | 32.54 | 1.00 | 11.74 | 2.20 |
| 80 | 2012 | 36.77 | 40.63 | 64.17 | 68.75 | 280000000 | 400000000 | 89000 | 3045.74 | 31.13 | 1.00 | 12.11 | 69.73 |
| 80 | 2013 | 36.02 | 41.37 | 63.29 | 67.38 | 320000000 | 430000000 | 95600 | 3322.04 | 32.09 | 1.00 | 12.59 | 12.50 |
| 80 | 2014 | 35.21 | 40.00 | 64.92 | 65.50 | 330000000 | 440000000 | 93900 | 3328.80 | 31.17 | 1.00 | 14.04 | 4.50 |
| 80 | 2015 | 39 | 41.29 | 68.21 | 68.50 | 300000000 | 370000000 | 94400 | 2732.46 | 31.90 | 1.00 | 18.82 | 36.01 |
| 80 | 2016 | 39.77 | 41.58 | 65.46 | 72.50 | 340000000 | 330000000 | 121000 | 2880.44 | 32.31 | 1.00 | 19.92 | 0.98 |
| 80 | 2017 | 36.42 | 40.79 | 64.79 | 67.25 | 440000000 | 380000000 | 145000 | 3509.69 | 31.10 | 1.00 | 18.50 | 38.00 |
| 80 | 2018 | 34.81 | 40.46 | 63.42 | 65.75 | 500000000 | 440000000 | 160000 | 4234.00 | 30.14 | 1.00 | 16.80 | 46.11 |
| 80 | 2019 | 34.06 | 39.92 | 62.21 | 66.00 | 530000000 | 480000000 | 4400000 | 1407.81 | 32.42 | 1.00 | 1499.26 | 4.30 |
| 81 | 2000 | 28.65 | 31.25 | 63.00 | 63.04 | 2300000000 | 510000000 | 4300000 | 1334.94 | 26.79 | 6.00 | 10.63 | 37.18 |
| 81 | 2001 | 28 | 29.88 | 63.29 | 62.83 | 3000000000 | 590000000 | 4400000 | 1339.29 | 28.21 | 7.00 | 11.30 | 12.00 |
| 81 | 2002 | 28.21 | 30.08 | 62.75 | 63.58 | 3200000000 | 670000000 | 4500000 | 1416.49 | 28.92 | 7.00 | 11.02 | 33.97 |
| 81 | 2003 | 25.69 | 27.00 | 61.33 | 63.04 | 3800000000 | 850000000 | 4800000 | 1725.46 | 27.50 | 7.00 | 9.57 | 10.60 |
| 81 | 2004 | 23.5 | 26.17 | 58.96 | 61.88 | 4500000000 | 910000000 | 5500000 | 1952.90 | 28.11 | 8.00 | 8.87 | 0.90 |
| 81 | 2005 | 25.37 | 27.75 | 58.13 | 64.88 | 5400000000 | 1000000000 | 5800000 | 2018.03 | 30.92 | 8.00 | 8.87 | 13.80 |
| 81 | 2006 | 24.33 | 27.38 | 57.75 | 63.54 | 6900000000 | 1100000000 | 6600000 | 2196.01 | 32.76 | 8.00 | 8.80 | 93.49 |
| 81 | 2007 | 26.94 | 29.29 | 58.38 | 66.21 | 8300000000 | 1400000000 | 7400000 | 2499.26 | 34.57 | 8.00 | 8.19 | 55.25 |
| 81 | 2008 | 26.9 | 29.75 | 58.96 | 65.08 | 8900000000 | 1900000000 | 7900000 | 2890.36 | 35.74 | 8.00 | 7.75 | 79.05 |
| 81 | 2009 | 26.73 | 30.33 | 58.79 | 64.33 | 8000000000 | 1700000000 | 8300000 | 2866.92 | 28.00 | 8.00 | 8.06 | 92.77 |
| 81 | 2010 | 27.42 | 30.46 | 59.25 | 65.13 | 8200000000 | 1900000000 | 9300000 | 2839.93 | 32.23 | 8.00 | 8.42 | 85.19 |
| 81 | 2011 | 29.65 | 31.79 | 59.63 | 67.88 | 9100000000 | 2300000000 | 9300000 | 3046.95 | 34.70 | 8.00 | 8.09 | 18.90 |
| 81 | 2012 | 29.83 | 32.92 | 59.71 | 67.04 | 8500000000 | 2100000000 | 9400000 | 2912.66 | 34.93 | 9.00 | 8.63 | 42.20 |
| 81 | 2013 | 32.71 | 35.67 | 60.58 | 69.17 | 8200000000 | 2000000000 | 10000000 | 3121.68 | 32.78 | 9.00 | 8.41 | 0.90 |
| 81 | 2014 | 31.98 | 35.79 | 61.08 | 67.08 | 9100000000 | 2300000000 | 10000000 | 3171.70 | 34.63 | 9.00 | 8.41 | 9.80 |
| 81 | 2015 | 32.08 | 35.75 | 63.13 | 65.29 | 7800000000 | 2200000000 | 10000000 | 2875.26 | 34.80 | 9.00 | 9.76 | 56.86 |
| 81 | 2016 | 30.15 | 34.88 | 59.75 | 65.67 | 7900000000 | 2300000000 | 10000000 | 2896.72 | 35.35 | 9.00 | 9.81 | 47.50 |
| 81 | 2017 | 29.87 | 35.00 | 59.63 | 65.13 | 9100000000 | 2800000000 | 11000000 | 3036.33 | 37.21 | 9.00 | 9.69 | 15.00 |
| 81 | 2018 | 30.6 | 36.50 | 59.79 | 64.92 | 9500000000 | 3000000000 | 12000000 | 3222.20 | 38.73 | 9.00 | 9.39 | 74.44 |
| 81 | 2019 | 30.42 | 36.42 | 59.58 | 64.83 | 10000000000 | 3200000000 | 174000 | 4503.52 | 31.41 | 9.00 | 15.11 | 67.34 |
| 82 | 2000 | 44.58 | 40.75 | 73.29 | 75.13 | 96000000 | 120000000 | 223000 | 313.57 | 11.43 | 1.00 | 15.23 | 17.46 |
| 82 | 2001 | 40.93 | 36.88 | 70.58 | 74.42 | 100000000 | 130000000 | 323000 | 291.47 | 18.69 | 1.00 | 20.70 | 32.00 |
| 82 | 2002 | 39.43 | 36.42 | 67.95 | 74.50 | 65000000 | 120000000 | 541000 | 299.68 | 21.54 | 1.00 | 23.68 | 23.67 |
| 82 | 2003 | 38.65 | 36.79 | 66.00 | 74.50 | 110000000 | 140000000 | 441000 | 323.52 | 22.56 | 1.00 | 23.78 | 3.20 |
| 82 | 2004 | 36.75 | 35.63 | 65.88 | 72.00 | 96000000 | 140000000 | 470000 | 381.16 | 25.68 | 1.00 | 22.58 | 55.20 |
| 82 | 2005 | 34.1 | 33.96 | 66.75 | 67.50 | 140000000 | 190000000 | 578000 | 415.35 | 27.02 | 1.00 | 23.06 | 39.10 |
| 82 | 2006 | 29.71 | 31.08 | 63.33 | 65.00 | 150000000 | 200000000 | 664000 | 434.03 | 27.07 | 1.00 | 25.40 | 43.07 |
| 82 | 2007 | 29.15 | 30.83 | 61.08 | 66.38 | 180000000 | 210000000 | 771000 | 480.93 | 27.33 | 1.00 | 25.84 | 12.30 |
| 82 | 2008 | 29.75 | 30.50 | 62.17 | 66.83 | 210000000 | 240000000 | 1200000 | 564.48 | 26.45 | 1.00 | 24.30 | 40.22 |
| 82 | 2009 | 32.1 | 30.33 | 65.79 | 68.08 | 220000000 | 250000000 | 1500000 | 519.76 | 27.10 | 1.00 | 27.52 | 56.00 |
| 82 | 2010 | 37.06 | 30.75 | 69.42 | 73.96 | 140000000 | 250000000 | 1700000 | 471.18 | 28.46 | 1.00 | 33.96 | 13.01 |
| 82 | 2011 | 32.87 | 33.17 | 63.75 | 68.83 | 170000000 | 260000000 | 1900000 | 594.59 | 30.10 | 1.00 | 29.07 | 28.71 |
| 82 | 2012 | 32.92 | 33.29 | 62.04 | 70.50 | 220000000 | 250000000 | 2100000 | 657.65 | 28.88 | 1.00 | 28.37 | 51.40 |
| 82 | 2013 | 39.6 | 34.25 | 68.96 | 76.00 | 230000000 | 310000000 | 1900000 | 664.08 | 26.74 | 1.00 | 30.10 | 67.09 |
| 82 | 2014 | 44.02 | 35.88 | 72.92 | 79.25 | 230000000 | 330000000 | 1700000 | 673.97 | 31.28 | 1.00 | 31.35 | 51.65 |
| 82 | 2015 | 45.08 | 36.21 | 76.33 | 77.63 | 200000000 | 320000000 | 1600000 | 589.86 | 31.08 | 1.00 | 39.98 | 72.20 |
| 82 | 2016 | 49.23 | 40.04 | 81.79 | 76.63 | 110000000 | 290000000 | 1600000 | 428.93 | 33.55 | 1.00 | 63.06 | 71.00 |
| 82 | 2017 | 49.27 | 41.67 | 77.17 | 79.71 | 160000000 | 240000000 | 1400000 | 461.42 | 38.58 | 1.00 | 63.58 | 50.00 |
| 82 | 2018 | 43.98 | 40.75 | 69.83 | 77.38 | 330000000 | 140000000 | 2700000 | 498.96 | 47.76 | 1.00 | 60.33 | 81.23 |
| 82 | 2019 | 42.56 | 41.46 | 68.17 | 75.50 | 320000000 | 99000000 | 13000000 | 3204.09 | 39.11 | 1.00 | 9.08 | 68.02 |
| 83 | 2000 | 28.92 | 30.33 | 63.96 | 63.54 | 9100000000 | 6400000000 | 21000000 | 7157.81 | 25.41 | 21.00 | 9.46 | 22.50 |
| 83 | 2001 | 26.94 | 26.88 | 63.50 | 63.50 | 9200000000 | 6700000000 | 20000000 | 7544.57 | 22.67 | 21.00 | 9.34 | 37.44 |
| 83 | 2002 | 29.58 | 31.42 | 63.79 | 63.96 | 9500000000 | 7100000000 | 20000000 | 7593.14 | 22.55 | 22.00 | 9.66 | 32.00 |
| 83 | 2003 | 28.83 | 30.83 | 63.79 | 63.04 | 10000000000 | 7300000000 | 19000000 | 7075.37 | 24.36 | 23.00 | 10.79 | 14.91 |
| 83 | 2004 | 25.69 | 28.00 | 61.42 | 61.96 | 12000000000 | 8000000000 | 21000000 | 7484.49 | 28.41 | 24.00 | 11.29 | 5.00 |
| 83 | 2005 | 24.15 | 27.04 | 58.96 | 62.29 | 13000000000 | 9000000000 | 22000000 | 8277.67 | 30.38 | 25.00 | 10.90 | 19.00 |
| 83 | 2006 | 24.17 | 26.88 | 58.54 | 62.92 | 13000000000 | 9400000000 | 21000000 | 9068.29 | 27.33 | 26.00 | 10.90 | 68.87 |
| 83 | 2007 | 21.9 | 24.50 | 58.33 | 60.96 | 14000000000 | 9900000000 | 22000000 | 9642.68 | 27.53 | 27.00 | 10.93 | 54.39 |
| 83 | 2008 | 23.56 | 26.00 | 59.33 | 61.79 | 15000000000 | 10000000000 | 23000000 | 10016.60 | 27.70 | 29.00 | 11.13 | 10.07 |
| 83 | 2009 | 30.42 | 29.13 | 61.50 | 70.21 | 13000000000 | 8700000000 | 22000000 | 8002.97 | 27.16 | 29.00 | 13.51 | 12.28 |
| 83 | 2010 | 27.21 | 31.13 | 58.42 | 64.88 | 13000000000 | 9000000000 | 23000000 | 9271.40 | 29.70 | 31.00 | 12.64 | 85.38 |
| 83 | 2011 | 26.04 | 30.83 | 58.79 | 62.46 | 12000000000 | 9700000000 | 23000000 | 10203.40 | 31.04 | 31.00 | 12.42 | 34.90 |
| 83 | 2012 | 27.12 | 32.25 | 59.25 | 62.75 | 13000000000 | 11000000000 | 23000000 | 10241.70 | 32.27 | 31.00 | 13.17 | 35.07 |
| 83 | 2013 | 25.94 | 31.04 | 58.79 | 62.04 | 14000000000 | 12000000000 | 24000000 | 10725.20 | 31.31 | 32.00 | 12.77 | 11.50 |
| 83 | 2014 | 27.85 | 33.83 | 58.79 | 63.08 | 17000000000 | 13000000000 | 29000000 | 10922.40 | 31.89 | 33.00 | 13.29 | 52.25 |
| 83 | 2015 | 32.79 | 40.04 | 62.25 | 63.29 | 19000000000 | 13000000000 | 32000000 | 9605.95 | 34.56 | 34.00 | 15.85 | 38.30 |
| 83 | 2016 | 32.79 | 39.58 | 62.04 | 63.96 | 21000000000 | 13000000000 | 35000000 | 8739.76 | 37.06 | 35.00 | 18.66 | 54.66 |
| 83 | 2017 | 33.25 | 40.04 | 61.96 | 64.50 | 22000000000 | 14000000000 | 39000000 | 9278.42 | 37.69 | 35.00 | 18.93 | 18.20 |
| 83 | 2018 | 32.21 | 38.42 | 61.75 | 64.25 | 24000000000 | 14000000000 | 41000000 | 9673.44 | 39.29 | 36.00 | 19.24 | 55.80 |
| 83 | 2019 | 30.81 | 36.46 | 60.50 | 64.67 | 26000000000 | 12000000000 | 2000000 | 503.57 | 41.01 | 36.00 | 57.07 | 85.00 |
| 84 | 2000 | 22.92 | 24.92 | 57.92 | 63.00 | 150000000 | 22000000 | 656000 | 2136.44 | 41.67 | 0.00 | 6.94 | 60.00 |
| 84 | 2001 | 23.14 | 23.58 | 59.33 | 63.38 | 250000000 | 24000000 | 670000 | 1906.29 | 42.01 | 0.00 | 8.61 | 54.20 |
| 84 | 2002 | 23.87 | 23.50 | 60.08 | 64.17 | 240000000 | 26000000 | 757000 | 1769.18 | 47.21 | 0.00 | 10.54 | 26.40 |
| 84 | 2003 | 23.25 | 24.21 | 57.79 | 64.50 | 380000000 | 28000000 | 695000 | 2560.77 | 44.46 | 0.00 | 7.56 | 2.90 |
| 84 | 2004 | 21.85 | 23.46 | 56.58 | 63.67 | 430000000 | 30000000 | 716000 | 3396.91 | 40.59 | 0.00 | 6.46 | 76.92 |
| 84 | 2005 | 21.81 | 23.00 | 58.13 | 62.50 | 360000000 | 32000000 | 778000 | 3674.00 | 41.24 | 0.00 | 6.38 | 78.39 |
| 84 | 2006 | 22.4 | 22.50 | 59.29 | 63.00 | 470000000 | 34000000 | 833000 | 3974.52 | 46.30 | 0.00 | 6.77 | 6.50 |
| 84 | 2007 | 20.4 | 22.46 | 58.08 | 60.25 | 540000000 | 36000000 | 929000 | 4356.24 | 50.48 | 1.00 | 7.05 | 80.03 |
| 84 | 2008 | 19.33 | 21.00 | 58.50 | 59.17 | 490000000 | 38000000 | 931000 | 4153.25 | 54.35 | 1.00 | 8.25 | 79.00 |
| 84 | 2009 | 18.81 | 21.00 | 58.13 | 58.50 | 510000000 | 40000000 | 980000 | 4265.26 | 52.35 | 1.00 | 8.52 | 3.50 |
| 84 | 2010 | 25.37 | 21.13 | 60.75 | 68.88 | 470000000 | 42000000 | 984000 | 5318.01 | 47.76 | 1.00 | 7.33 | 71.75 |
| 84 | 2011 | 26.25 | 23.25 | 61.58 | 67.67 | 520000000 | 55000000 | 1000000 | 5723.33 | 45.53 | 1.00 | 7.30 | 9.96 |
| 84 | 2012 | 27.17 | 24.50 | 62.33 | 67.50 | 420000000 | 53000000 | 1100000 | 5942.27 | 43.41 | 1.00 | 8.19 | 29.34 |
| 84 | 2013 | 28.12 | 25.00 | 63.96 | 67.29 | 360000000 | 47000000 | 1200000 | 5377.73 | 37.56 | 2.00 | 9.75 | 88.00 |
| 84 | 2014 | 26.46 | 25.00 | 62.38 | 65.54 | 550000000 | 67000000 | 1300000 | 5435.17 | 39.25 | 2.00 | 10.84 | 40.30 |
| 84 | 2015 | 26.58 | 25.00 | 63.21 | 64.96 | 540000000 | 71000000 | 1400000 | 4869.38 | 35.56 | 2.00 | 12.88 | 48.56 |
| 84 | 2016 | 28.85 | 25.58 | 64.63 | 67.50 | 300000000 | 82000000 | 1500000 | 4523.09 | 35.17 | 2.00 | 14.71 | 46.91 |
| 84 | 2017 | 29.4 | 25.67 | 63.71 | 69.42 | 450000000 | 73000000 | 1500000 | 5303.31 | 34.03 | 2.00 | 13.31 | 51.90 |
| 84 | 2018 | 29.48 | 26.00 | 62.96 | 70.00 | 490000000 | 69000000 | 1500000 | 5495.43 | 36.34 | 2.00 | 13.23 | 80.72 |
| 84 | 2019 | 29.54 | 27.00 | 62.58 | 69.50 | 450000000 | 89000000 | 97000000 | 9946.03 | 38.83 | 2.00 | 19.56 | 96.21 |
| 85 | 2000 | 30.81 | 34.92 | 63.08 | 63.63 | 3300000000 | 2700000000 | 5900000 | 3032.43 | 27.16 | 4.00 | 6.94 | 62.31 |
| 85 | 2001 | 28.94 | 31.54 | 63.50 | 62.83 | 3300000000 | 2400000000 | 5800000 | 2666.47 | 29.37 | 4.00 | 8.61 | 45.06 |
| 85 | 2002 | 32.21 | 36.42 | 64.33 | 63.67 | 3700000000 | 2300000000 | 6400000 | 2502.28 | 31.78 | 4.00 | 10.54 | 16.00 |
| 85 | 2003 | 31.08 | 35.54 | 62.54 | 64.08 | 6700000000 | 3700000000 | 6500000 | 3751.28 | 26.89 | 5.00 | 7.56 | 3.73 |
| 85 | 2004 | 26.94 | 29.42 | 60.50 | 63.96 | 7600000000 | 4200000000 | 6700000 | 4833.63 | 25.47 | 6.00 | 6.46 | 92.26 |
| 85 | 2005 | 26.62 | 29.38 | 60.17 | 63.71 | 8600000000 | 4800000000 | 7400000 | 5383.66 | 26.45 | 8.00 | 6.36 | 82.35 |
| 85 | 2006 | 26.81 | 30.75 | 60.58 | 62.29 | 9200000000 | 5200000000 | 8400000 | 5602.01 | 29.27 | 8.00 | 6.77 | 4.00 |
| 85 | 2007 | 27.08 | 31.17 | 61.04 | 61.96 | 10000000000 | 6100000000 | 9100000 | 6095.62 | 31.17 | 9.00 | 7.05 | 42.32 |
| 85 | 2008 | 28.62 | 32.38 | 62.67 | 62.21 | 9200000000 | 6900000000 | 9600000 | 5760.81 | 35.62 | 9.00 | 8.26 | 63.80 |
| 85 | 2009 | 31.02 | 32.00 | 61.83 | 68.21 | 8700000000 | 6400000000 | 7000000 | 5862.80 | 27.91 | 9.00 | 8.47 | 35.14 |
| 85 | 2010 | 29.96 | 32.88 | 59.54 | 67.50 | 10000000000 | 8100000000 | 8100000 | 7328.62 | 28.62 | 9.00 | 7.32 | 81.44 |
| 85 | 2011 | 30.46 | 33.29 | 61.58 | 66.04 | 11000000000 | 8400000000 | 8300000 | 8007.41 | 30.46 | 9.00 | 7.26 | 36.24 |
| 85 | 2012 | 30.77 | 34.04 | 61.33 | 66.17 | 11000000000 | 7100000000 | 9200000 | 7501.47 | 29.72 | 9.00 | 8.21 | 89.88 |
| 85 | 2013 | 32.04 | 34.46 | 63.29 | 66.33 | 10000000000 | 6500000000 | 9500000 | 6832.46 | 30.97 | 10.00 | 9.66 | 12.45 |
| 85 | 2014 | 33.04 | 36.71 | 62.54 | 66.83 | 10000000000 | 6300000000 | 9500000 | 6433.19 | 31.47 | 10.00 | 10.85 | 6.11 |
| 85 | 2015 | 32.79 | 36.21 | 62.88 | 66.50 | 9100000000 | 5700000000 | 8900000 | 5734.63 | 30.15 | 11.00 | 12.76 | 1.68 |
| 85 | 2016 | 34.02 | 37.33 | 62.88 | 67.83 | 8800000000 | 5400000000 | 10000000 | 5272.92 | 30.58 | 11.00 | 14.71 | 48.98 |
| 85 | 2017 | 32.87 | 37.25 | 60.79 | 67.71 | 9700000000 | 6100000000 | 10000000 | 6132.48 | 29.63 | 12.00 | 13.32 | 47.50 |
| 85 | 2018 | 30.33 | 34.13 | 60.21 | 66.33 | 9800000000 | 6300000000 | 10000000 | 6374.03 | 29.91 | 13.00 | 13.23 | 21.20 |
| 85 | 2019 | 31.58 | 34.42 | 61.42 | 67.33 | 9100000000 | 5900000000 | 1700000 | 4957.46 | 35.81 | 13.00 | 13.16 | 30.50 |
| 86 | 2000 | 48.83 | 39.50 | 78.04 | 80.13 | 150000000 | 130000000 | 486000 | 1007.50 | 20.11 | 1.00 | 12.68 | 61.91 |
| 86 | 2001 | 43.1 | 34.79 | 78.08 | 73.33 | 180000000 | 130000000 | 483000 | 1034.55 | 19.04 | 1.00 | 13.37 | 9.80 |
| 86 | 2002 | 43.23 | 36.88 | 77.08 | 72.50 | 200000000 | 130000000 | 472000 | 1000.94 | 18.85 | 1.00 | 14.25 | 84.07 |
| 86 | 2003 | 46.83 | 41.17 | 74.92 | 77.58 | 230000000 | 140000000 | 526000 | 1005.73 | 20.71 | 1.00 | 15.10 | 92.86 |
| 86 | 2004 | 43.81 | 38.17 | 73.46 | 76.00 | 250000000 | 150000000 | 615000 | 1080.27 | 23.06 | 1.00 | 15.94 | 56.84 |
| 86 | 2005 | 37.67 | 35.63 | 69.83 | 69.88 | 280000000 | 160000000 | 712000 | 1162.29 | 24.38 | 1.00 | 16.73 | 18.12 |
| 86 | 2006 | 36.87 | 34.13 | 69.08 | 70.54 | 300000000 | 220000000 | 749000 | 1226.64 | 31.27 | 1.00 | 17.57 | 3.10 |
| 86 | 2007 | 35.15 | 32.54 | 66.46 | 71.29 | 330000000 | 240000000 | 800000 | 1327.96 | 33.06 | 1.00 | 18.45 | 2.89 |
| 86 | 2008 | 36.54 | 34.17 | 66.42 | 72.50 | 350000000 | 270000000 | 858000 | 1499.26 | 34.47 | 1.00 | 19.37 | 83.00 |
| 86 | 2009 | 37.85 | 38.08 | 63.71 | 73.92 | 380000000 | 260000000 | 932000 | 1444.37 | 33.98 | 1.00 | 20.34 | 10.60 |
| 86 | 2010 | 39.27 | 37.83 | 65.21 | 75.50 | 400000000 | 230000000 | 1000000 | 1503.87 | 40.47 | 1.00 | 21.36 | 24.00 |
| 86 | 2011 | 37.27 | 38.08 | 66.29 | 70.17 | 430000000 | 240000000 | 1100000 | 1655.80 | 45.02 | 2.00 | 22.42 | 73.43 |
| 86 | 2012 | 36.92 | 37.21 | 66.54 | 70.08 | 450000000 | 240000000 | 1200000 | 1760.45 | 47.47 | 2.00 | 23.55 | 12.00 |
| 86 | 2013 | 36.04 | 36.29 | 66.63 | 69.17 | 480000000 | 280000000 | 1200000 | 1811.64 | 45.21 | 2.00 | 24.72 | 7.50 |
| 86 | 2014 | 35.75 | 37.58 | 66.50 | 67.42 | 500000000 | 290000000 | 1300000 | 1934.07 | 45.01 | 2.00 | 25.96 | 69.30 |
| 86 | 2015 | 35.42 | 38.50 | 65.79 | 66.54 | 530000000 | 270000000 | 1400000 | 2049.85 | 40.08 | 2.00 | 27.26 | 5.00 |
| 86 | 2016 | 35.65 | 39.04 | 65.83 | 66.42 | 550000000 | 310000000 | 1500000 | 2107.57 | 38.88 | 2.00 | 28.62 | 70.45 |
| 86 | 2017 | 35.83 | 39.96 | 65.21 | 66.50 | 580000000 | 370000000 | 1800000 | 2159.16 | 41.34 | 2.00 | 30.05 | 61.94 |
| 86 | 2018 | 36.92 | 41.88 | 65.13 | 66.83 | 600000000 | 350000000 | 1300000 | 2020.55 | 42.15 | 2.00 | 31.55 | 10.50 |
| 86 | 2019 | 40.02 | 42.21 | 65.50 | 72.33 | 630000000 | 310000000 | 15000000 | 6001.40 | 29.85 | 2.00 | 13.14 | 73.12 |
| 87 | 2000 | 37.62 | 39.08 | 71.17 | 65.00 | 23000000 | 32000000 | 50000 | 197.52 | 14.33 | 2.00 | 710.21 | 61.25 |
| 87 | 2001 | 40.31 | 39.29 | 71.33 | 70.00 | 30000000 | 33000000 | 52000 | 208.07 | 13.46 | 2.00 | 732.40 | 67.23 |
| 87 | 2002 | 41.85 | 41.25 | 73.13 | 69.33 | 20000000 | 29000000 | 39000 | 227.14 | 11.92 | 2.00 | 693.71 | 15.25 |
| 87 | 2003 | 42.65 | 41.54 | 74.75 | 69.00 | 28000000 | 39000000 | 55000 | 267.85 | 13.27 | 2.00 | 579.90 | 91.95 |
| 87 | 2004 | 41.02 | 42.08 | 73.21 | 66.75 | 32000000 | 42000000 | 57000 | 285.10 | 14.30 | 2.00 | 527.34 | 45.88 |
| 87 | 2005 | 40 | 43.29 | 72.21 | 64.50 | 44000000 | 42000000 | 58000 | 321.09 | 14.43 | 2.00 | 527.26 | 2.30 |
| 87 | 2006 | 40.96 | 44.08 | 70.83 | 67.00 | 39000000 | 42000000 | 60000 | 335.34 | 13.90 | 2.00 | 522.43 | 68.20 |
| 87 | 2007 | 41.71 | 45.38 | 69.67 | 68.38 | 44000000 | 48000000 | 48000 | 387.78 | 13.13 | 2.00 | 478.63 | 4.35 |
| 87 | 2008 | 35.85 | 46.00 | 59.50 | 66.21 | 86000000 | 98000000 | 73000 | 473.42 | 13.27 | 2.00 | 446.00 | 65.80 |
| 87 | 2009 | 38.15 | 46.71 | 61.46 | 68.13 | 69000000 | 84000000 | 66000 | 459.42 | 15.07 | 2.00 | 470.29 | 2.80 |
| 87 | 2010 | 46.71 | 51.33 | 67.50 | 74.58 | 110000000 | 95000000 | 74000 | 473.30 | 16.32 | 2.00 | 494.79 | 74.70 |
| 87 | 2011 | 40.73 | 50.08 | 62.29 | 69.08 | 54000000 | 57000000 | 82000 | 508.40 | 15.41 | 2.00 | 471.25 | 16.40 |
| 87 | 2012 | 47.17 | 50.46 | 69.38 | 74.50 | 51000000 | 130000000 | 94000 | 526.35 | 16.21 | 2.00 | 510.56 | 5.00 |
| 87 | 2013 | 44.94 | 52.13 | 67.38 | 70.38 | 59000000 | 81000000 | 123000 | 548.89 | 17.09 | 3.00 | 493.90 | 38.20 |
| 87 | 2014 | 44.46 | 52.25 | 67.25 | 69.42 | 100000000 | 130000000 | 135000 | 562.12 | 16.02 | 3.00 | 493.76 | 4.00 |
| 87 | 2015 | 47.92 | 51.42 | 71.92 | 72.50 | 80000000 | 140000000 | 135000 | 483.34 | 13.65 | 3.00 | 591.21 | 43.37 |
| 87 | 2016 | 46.9 | 50.96 | 70.00 | 72.83 | 84000000 | 110000000 | 152000 | 494.72 | 11.96 | 3.00 | 592.61 | 55.42 |
| 87 | 2017 | 45.08 | 50.63 | 69.96 | 69.58 | 91000000 | 120000000 | 164000 | 516.89 | 12.80 | 4.00 | 580.66 | 6.00 |
| 87 | 2018 | 45.43 | 50.83 | 69.92 | 70.12 | 110000000 | 140000000 | 157000 | 571.53 | 11.31 | 4.00 | 555.45 | 39.75 |
| 87 | 2019 | 46.85 | 50.58 | 72.75 | 70.38 | 140000000 | 160000000 | 1500000 | 1912.90 | 45.11 | 4.00 | 33.06 | 12.94 |
| 88 | 2000 | 43.65 | 54.25 | 66.67 | 66.38 | 190000000 | 610000000 | 1500000 | 567.93 | 36.02 | 1.00 | 101.70 | 41.00 |
| 88 | 2001 | 39.35 | 53.38 | 61.08 | 64.25 | 170000000 | 860000000 | 1800000 | 590.38 | 28.25 | 1.00 | 111.23 | 13.50 |
| 88 | 2002 | 48.48 | 61.21 | 64.33 | 71.42 | 260000000 | 910000000 | 2000000 | 741.75 | 23.24 | 1.00 | 120.58 | 1.05 |
| 88 | 2003 | 44.9 | 58.46 | 62.29 | 69.04 | 58000000 | 2100000000 | 2300000 | 795.39 | 26.75 | 1.00 | 129.22 | 16.10 |
| 88 | 2004 | 43 | 56.29 | 61.46 | 68.25 | 49000000 | 1300000000 | 2600000 | 1007.87 | 20.25 | 1.00 | 132.89 | 94.65 |
| 88 | 2005 | 39.21 | 55.79 | 58.17 | 64.46 | 140000000 | 500000000 | 2800000 | 1268.38 | 21.03 | 2.00 | 131.27 | 60.34 |
| 88 | 2006 | 34.35 | 54.00 | 52.33 | 62.38 | 210000000 | 3500000000 | 3100000 | 1656.42 | 29.52 | 2.00 | 128.65 | 79.50 |
| 88 | 2007 | 34.4 | 56.17 | 51.96 | 60.67 | 340000000 | 6700000000 | 5200000 | 1883.46 | 21.24 | 2.00 | 125.81 | 93.18 |
| 88 | 2008 | 35.4 | 56.50 | 53.08 | 61.21 | 960000000 | 11000000000 | 5800000 | 2242.87 | 25.67 | 2.00 | 118.57 | 85.20 |
| 88 | 2009 | 41.35 | 54.38 | 58.42 | 69.92 | 790000000 | 6200000000 | 6100000 | 1891.34 | 18.63 | 2.00 | 148.88 | 20.32 |
| 88 | 2010 | 37.42 | 54.71 | 53.00 | 67.13 | 740000000 | 8300000000 | 6100000 | 2292.45 | 25.66 | 2.00 | 150.30 | 48.10 |
| 88 | 2011 | 34.73 | 54.00 | 51.88 | 63.58 | 690000000 | 9500000000 | 3800000 | 2520.40 | 31.62 | 2.00 | 153.86 | 2.50 |
| 88 | 2012 | 35.92 | 55.00 | 53.42 | 63.42 | 640000000 | 9200000000 | 4700000 | 2746.99 | 31.55 | 2.00 | 157.50 | 10.80 |
| 88 | 2013 | 36.25 | 55.58 | 53.50 | 63.42 | 620000000 | 9200000000 | 4000000 | 2998.07 | 18.05 | 2.00 | 157.31 | 60.69 |
| 88 | 2014 | 36.19 | 57.21 | 52.88 | 62.29 | 610000000 | 9200000000 | 4800000 | 3222.69 | 18.44 | 2.00 | 158.55 | 54.00 |
| 88 | 2015 | 39.94 | 55.75 | 58.38 | 65.75 | 460000000 | 9000000000 | 6000000 | 2730.43 | 10.67 | 2.00 | 192.44 | 18.29 |
| 88 | 2016 | 41.62 | 55.92 | 59.88 | 67.46 | 1100000000 | 4500000000 | 5300000 | 2176.00 | 9.22 | 2.00 | 253.49 | 76.71 |
| 88 | 2017 | 41.77 | 55.79 | 58.38 | 69.38 | 2600000000 | 8200000000 | 5600000 | 1968.56 | 13.17 | 2.00 | 305.79 | 68.35 |
| 88 | 2018 | 37.77 | 54.08 | 54.96 | 66.50 | 2000000000 | 13000000000 | 6000000 | 2032.73 | 15.50 | 2.00 | 306.08 | 21.00 |
| 88 | 2019 | 35.65 | 52.21 | 53.17 | 65.92 | 1500000000 | 16000000000 | 192000 | 553.90 | 11.02 | 2.00 | 530.24 | 34.68 |
| 89 | 2000 | 11.37 | 14.67 | 55.00 | 53.08 | 2500000000 | 4900000000 | 3100000 | 38131.50 | 45.73 | 4.00 | 8.80 | 26.46 |
| 89 | 2001 | 7.92 | 9.29 | 53.21 | 53.33 | 2400000000 | 4700000000 | 3100000 | 38542.70 | 44.96 | 4.00 | 8.99 | 3.95 |
| 89 | 2002 | 8.77 | 10.33 | 54.08 | 53.13 | 2600000000 | 5600000000 | 3100000 | 43084.50 | 40.37 | 4.00 | 7.98 | 59.52 |
| 89 | 2003 | 9.06 | 11.17 | 53.08 | 53.88 | 3000000000 | 7100000000 | 3300000 | 50134.90 | 39.63 | 4.00 | 7.08 | 41.44 |
| 89 | 2004 | 7.71 | 11.88 | 51.54 | 52.00 | 3500000000 | 8900000000 | 3600000 | 57603.80 | 41.09 | 5.00 | 6.74 | 45.13 |
| 89 | 2005 | 7.62 | 11.63 | 52.04 | 51.58 | 4200000000 | 10000000000 | 3800000 | 66810.50 | 43.40 | 7.00 | 6.44 | 16.00 |
| 89 | 2006 | 8 | 11.63 | 52.38 | 52.00 | 4300000000 | 11000000000 | 4100000 | 74148.30 | 44.64 | 7.00 | 6.41 | 49.05 |
| 89 | 2007 | 8.12 | 11.17 | 52.92 | 52.17 | 5300000000 | 13000000000 | 4400000 | 85140.00 | 43.30 | 7.00 | 5.86 | 60.27 |
| 89 | 2008 | 8.81 | 11.46 | 53.50 | 52.67 | 5700000000 | 15000000000 | 4300000 | 96944.10 | 45.92 | 7.00 | 5.64 | 14.10 |
| 89 | 2009 | 11.6 | 12.13 | 55.42 | 55.67 | 4900000000 | 13000000000 | 4300000 | 79977.70 | 39.24 | 7.00 | 6.29 | 35.27 |
| 89 | 2010 | 9.46 | 11.25 | 53.33 | 54.33 | 5300000000 | 15000000000 | 4800000 | 87693.80 | 39.76 | 8.00 | 6.04 | 54.45 |
| 89 | 2011 | 10.15 | 12.00 | 54.13 | 54.17 | 6600000000 | 17000000000 | 5000000 | 100601.00 | 41.27 | 8.00 | 5.60 | 69.81 |
| 89 | 2012 | 9.77 | 12.00 | 53.71 | 53.83 | 6800000000 | 18000000000 | 4500000 | 101524.00 | 40.68 | 8.00 | 5.82 | 55.07 |
| 89 | 2013 | 9 | 12.00 | 53.25 | 52.75 | 7200000000 | 20000000000 | 4800000 | 102913.00 | 39.20 | 8.00 | 5.88 | 72.90 |
| 89 | 2014 | 8.73 | 11.00 | 53.46 | 53.00 | 7500000000 | 20000000000 | 4900000 | 97019.20 | 39.00 | 8.00 | 6.30 | 72.00 |
| 89 | 2015 | 12.46 | 12.08 | 58.08 | 54.75 | 6400000000 | 16000000000 | 5400000 | 74355.50 | 37.80 | 9.00 | 8.06 | 58.00 |
| 89 | 2016 | 11.6 | 13.46 | 55.00 | 54.75 | 6300000000 | 16000000000 | 6000000 | 70459.20 | 35.46 | 9.00 | 8.40 | 81.64 |
| 89 | 2017 | 11.83 | 12.71 | 55.42 | 55.54 | 6800000000 | 18000000000 | 6300000 | 75496.80 | 36.33 | 9.00 | 8.27 | 70.58 |
| 89 | 2018 | 10.21 | 10.92 | 55.25 | 54.25 | 7100000000 | 19000000000 | 5700000 | 81734.50 | 38.44 | 9.00 | 8.13 | 24.30 |
| 89 | 2019 | 10.9 | 12.63 | 55.08 | 54.08 | 6900000000 | 18000000000 | 5100000 | 2229.86 | 14.22 | 9.00 | 306.38 | 33.79 |
| 90 | 2000 | 19.87 | 10.42 | 67.33 | 62.00 | 6000000000 | 2800000000 | 5600000 | 11497.80 | 28.20 | 10.00 | 0.66 | 37.50 |
| 90 | 2001 | 20.46 | 11.04 | 66.42 | 63.46 | 6300000000 | 2600000000 | 5400000 | 11724.60 | 27.44 | 12.00 | 0.69 | 17.45 |
| 90 | 2002 | 21.48 | 13.08 | 65.58 | 64.29 | 6600000000 | 2600000000 | 5600000 | 12875.30 | 27.07 | 12.00 | 0.67 | 7.10 |
| 90 | 2003 | 21.54 | 13.71 | 64.54 | 64.83 | 7700000000 | 3000000000 | 5400000 | 15763.00 | 27.37 | 12.00 | 0.61 | 22.73 |
| 90 | 2004 | 21.9 | 16.50 | 63.58 | 63.71 | 8900000000 | 3400000000 | 5700000 | 18031.00 | 27.67 | 13.00 | 0.55 | 70.80 |
| 90 | 2005 | 22.23 | 16.17 | 64.63 | 63.67 | 9000000000 | 3700000000 | 5800000 | 18773.10 | 27.08 | 13.00 | 0.55 | 55.83 |
| 90 | 2006 | 23.19 | 15.92 | 65.79 | 64.67 | 10000000000 | 4100000000 | 6300000 | 19822.80 | 30.36 | 13.00 | 0.54 | 12.58 |
| 90 | 2007 | 22.21 | 16.25 | 63.92 | 64.25 | 13000000000 | 4900000000 | 6800000 | 22782.10 | 31.19 | 13.00 | 0.50 | 14.52 |
| 90 | 2008 | 23.31 | 16.79 | 65.33 | 64.50 | 14000000000 | 5300000000 | 7000000 | 24847.50 | 31.26 | 13.00 | 0.54 | 87.48 |
| 90 | 2009 | 26.85 | 17.96 | 69.50 | 66.25 | 12000000000 | 4600000000 | 6400000 | 23059.80 | 27.29 | 13.00 | 0.64 | 37.00 |
| 90 | 2010 | 28.79 | 21.00 | 67.08 | 69.50 | 13000000000 | 4700000000 | 6800000 | 22498.70 | 30.07 | 14.00 | 0.65 | 36.80 |
| 90 | 2011 | 30.06 | 26.08 | 64.92 | 69.13 | 15000000000 | 5000000000 | 7300000 | 23186.90 | 34.45 | 14.00 | 0.62 | 13.47 |
| 90 | 2012 | 31.65 | 27.67 | 65.63 | 70.00 | 15000000000 | 4500000000 | 7500000 | 20564.90 | 37.78 | 15.00 | 0.63 | 55.05 |
| 90 | 2013 | 30.79 | 29.42 | 63.46 | 68.71 | 16000000000 | 4900000000 | 9200000 | 21647.00 | 39.61 | 15.00 | 0.64 | 42.30 |
| 90 | 2014 | 27.87 | 27.63 | 63.67 | 64.46 | 18000000000 | 5200000000 | 10000000 | 22074.30 | 40.22 | 16.00 | 0.61 | 57.20 |
| 90 | 2015 | 26.35 | 24.38 | 66.17 | 62.17 | 16000000000 | 4600000000 | 12000000 | 19242.40 | 40.62 | 16.00 | 0.65 | 22.50 |
| 90 | 2016 | 23.27 | 22.00 | 62.79 | 61.75 | 17000000000 | 4700000000 | 13000000 | 19978.40 | 40.21 | 16.00 | 0.74 | 59.90 |
| 90 | 2017 | 21.79 | 19.79 | 62.83 | 60.96 | 22000000000 | 5500000000 | 15000000 | 21490.40 | 42.72 | 16.00 | 0.78 | 88.00 |
| 90 | 2018 | 21.81 | 19.83 | 63.33 | 60.46 | 24000000000 | 6400000000 | 16000000 | 23461.60 | 43.71 | 16.00 | 0.75 | 66.45 |
| 90 | 2019 | 22.52 | 20.29 | 64.00 | 60.75 | 25000000000 | 7000000000 | 5900000 | 75419.60 | 36.95 | 16.00 | 7.99 | 73.00 |
| 91 | 2000 | 16.77 | 19.83 | 52.50 | 61.21 | 6000000000 | 43000000000 | 4800000 | 38532.00 | 10.62 | 11.00 | 107.77 | 29.40 |
| 91 | 2001 | 15.12 | 14.08 | 54.00 | 62.17 | 5800000000 | 36000000000 | 4800000 | 33846.50 | 10.23 | 11.00 | 121.53 | 4.60 |
| 91 | 2002 | 15.71 | 14.21 | 53.00 | 64.21 | 6100000000 | 35000000000 | 5200000 | 32289.30 | 11.02 | 11.00 | 125.39 | 78.25 |
| 91 | 2003 | 13.92 | 14.33 | 50.96 | 62.54 | 11000000000 | 37000000000 | 5200000 | 34808.40 | 11.64 | 11.00 | 115.93 | 80.00 |
| 91 | 2004 | 14.19 | 16.21 | 51.79 | 60.38 | 14000000000 | 48000000000 | 6100000 | 37688.70 | 12.97 | 12.00 | 108.19 | 8.90 |
| 91 | 2005 | 15.62 | 16.92 | 53.50 | 60.83 | 16000000000 | 48000000000 | 6700000 | 37217.60 | 14.01 | 13.00 | 110.22 | 80.62 |
| 91 | 2006 | 15 | 16.42 | 54.25 | 59.33 | 11000000000 | 38000000000 | 7300000 | 35434.00 | 15.87 | 13.00 | 116.30 | 83.45 |
| 91 | 2007 | 15.81 | 19.38 | 54.00 | 58.25 | 12000000000 | 37000000000 | 8300000 | 35275.20 | 17.49 | 14.00 | 117.75 | 5.10 |
| 91 | 2008 | 17.37 | 21.75 | 54.08 | 58.92 | 14000000000 | 39000000000 | 8400000 | 39339.30 | 17.42 | 14.00 | 103.36 | 72.00 |
| 91 | 2009 | 21.06 | 21.50 | 56.38 | 64.25 | 13000000000 | 35000000000 | 6800000 | 40855.20 | 12.52 | 14.00 | 93.57 | 10.90 |
| 91 | 2010 | 18.81 | 19.38 | 56.75 | 61.50 | 15000000000 | 39000000000 | 8600000 | 44507.70 | 15.04 | 14.00 | 87.78 | 36.90 |
| 91 | 2011 | 19.35 | 20.54 | 56.33 | 61.83 | 13000000000 | 40000000000 | 6200000 | 48168.00 | 14.93 | 16.00 | 79.81 | 90.00 |
| 91 | 2012 | 20.23 | 21.17 | 56.75 | 62.54 | 16000000000 | 41000000000 | 8400000 | 48603.50 | 14.54 | 16.00 | 79.79 | 44.03 |
| 91 | 2013 | 20.42 | 17.67 | 60.67 | 62.50 | 17000000000 | 32000000000 | 10000000 | 40454.40 | 15.92 | 17.00 | 97.60 | 51.04 |
| 91 | 2014 | 19.06 | 17.88 | 57.75 | 62.50 | 21000000000 | 29000000000 | 13000000 | 38109.40 | 17.54 | 18.00 | 105.95 | 54.17 |
| 91 | 2015 | 18.87 | 17.00 | 59.04 | 61.71 | 27000000000 | 23000000000 | 20000000 | 34524.50 | 17.61 | 19.00 | 121.04 | 53.06 |
| 91 | 2016 | 17.98 | 16.92 | 57.54 | 61.50 | 33000000000 | 26000000000 | 24000000 | 38761.80 | 16.27 | 20.00 | 108.79 | 82.17 |
| 91 | 2017 | 16.79 | 15.88 | 57.04 | 60.67 | 37000000000 | 26000000000 | 29000000 | 38386.50 | 17.75 | 21.00 | 112.17 | 96.55 |
| 91 | 2018 | 16.96 | 17.83 | 56.67 | 59.42 | 45000000000 | 28000000000 | 31000000 | 39159.40 | 18.52 | 22.00 | 110.42 | 62.85 |
| 91 | 2019 | 18.4 | 19.04 | 57.58 | 60.17 | 49000000000 | 29000000000 | 17000000 | 23252.10 | 43.51 | 22.00 | 0.72 | 48.52 |
| 92 | 2000 | 15.98 | 13.33 | 64.29 | 54.33 | 4800000000 | 9000000000 | 3800000 | 29624.90 | 43.26 | 11.00 | 9.16 | 30.00 |
| 92 | 2001 | 14.27 | 8.58 | 64.79 | 55.17 | 5200000000 | 7900000000 | 4100000 | 27247.90 | 42.92 | 12.00 | 10.33 | 9.10 |
| 92 | 2002 | 15.42 | 8.38 | 63.46 | 59.00 | 5700000000 | 8200000000 | 4300000 | 29899.20 | 41.25 | 12.00 | 9.74 | 94.63 |
| 92 | 2003 | 13.52 | 9.08 | 61.00 | 56.96 | 6500000000 | 9400000000 | 4300000 | 37321.80 | 40.33 | 12.00 | 8.09 | 84.17 |
| 92 | 2004 | 12.6 | 9.04 | 60.04 | 56.13 | 7700000000 | 11000000000 | 4700000 | 42821.70 | 42.69 | 13.00 | 7.35 | 4.50 |
| 92 | 2005 | 12.67 | 9.63 | 59.71 | 56.00 | 7600000000 | 11000000000 | 4900000 | 43437.10 | 45.02 | 14.00 | 7.47 | 45.90 |
| 92 | 2006 | 12.52 | 10.38 | 60.17 | 54.50 | 9700000000 | 11000000000 | 4700000 | 46593.60 | 47.49 | 15.00 | 7.38 | 67.97 |
| 92 | 2007 | 12.21 | 11.13 | 60.04 | 53.25 | 12000000000 | 14000000000 | 5200000 | 53700.00 | 47.61 | 15.00 | 6.76 | 40.28 |
| 92 | 2008 | 13.44 | 11.50 | 60.58 | 54.79 | 12000000000 | 15000000000 | 4600000 | 56152.60 | 49.09 | 15.00 | 6.59 | 81.92 |
| 92 | 2009 | 16.81 | 11.46 | 61.92 | 60.25 | 9800000000 | 12000000000 | 4900000 | 46947.00 | 43.45 | 15.00 | 7.65 | 48.10 |
| 92 | 2010 | 16.46 | 11.42 | 59.54 | 61.96 | 11000000000 | 14000000000 | 5200000 | 52869.00 | 44.68 | 15.00 | 7.21 | 91.51 |
| 92 | 2011 | 14.77 | 13.00 | 60.33 | 56.21 | 12000000000 | 16000000000 | 5200000 | 60755.80 | 45.27 | 15.00 | 6.49 | 14.00 |
| 92 | 2012 | 15.29 | 12.67 | 60.75 | 57.17 | 12000000000 | 18000000000 | 5100000 | 58037.80 | 45.03 | 16.00 | 6.78 | 6.60 |
| 92 | 2013 | 14.71 | 12.79 | 60.00 | 56.63 | 13000000000 | 20000000000 | 5200000 | 61126.90 | 42.53 | 16.00 | 6.51 | 2.20 |
| 92 | 2014 | 16.23 | 13.58 | 61.38 | 57.50 | 14000000000 | 22000000000 | 5700000 | 60020.40 | 43.27 | 16.00 | 6.86 | 51.70 |
| 92 | 2015 | 16.1 | 14.00 | 62.83 | 55.38 | 15000000000 | 23000000000 | 6500000 | 51545.50 | 43.77 | 16.00 | 8.43 | 45.96 |
| 92 | 2016 | 14.17 | 13.54 | 59.17 | 55.63 | 16000000000 | 25000000000 | 6800000 | 51965.20 | 42.69 | 16.00 | 8.56 | 27.93 |
| 92 | 2017 | 14.67 | 13.08 | 60.33 | 55.92 | 17000000000 | 27000000000 | 7100000 | 53791.50 | 43.73 | 16.00 | 8.55 | 31.00 |
| 92 | 2018 | 15.77 | 14.63 | 61.00 | 55.92 | 18000000000 | 29000000000 | 7400000 | 54589.10 | 45.68 | 16.00 | 8.69 | 63.30 |
| 92 | 2019 | 15.75 | 14.00 | 60.83 | 56.67 | 19000000000 | 31000000000 | 32000000 | 40246.90 | 19.30 | 16.00 | 108.68 | 10.60 |
| 93 | 2000 | 11.23 | 11.92 | 55.79 | 54.75 | 9100000000 | 7800000000 | 7800000 | 37868.30 | 52.12 | 4.00 | 1.69 | 84.77 |
| 93 | 2001 | 9.4 | 7.75 | 56.17 | 54.88 | 9400000000 | 7500000000 | 7500000 | 38539.00 | 50.89 | 5.00 | 1.69 | 93.96 |
| 93 | 2002 | 8.33 | 7.38 | 53.63 | 55.67 | 9200000000 | 7600000000 | 6900000 | 41376.40 | 48.94 | 5.00 | 1.56 | 60.31 |
| 93 | 2003 | 8.96 | 8.50 | 53.17 | 56.25 | 10000000000 | 9100000000 | 6500000 | 48087.60 | 48.11 | 6.00 | 1.35 | 17.80 |
| 93 | 2004 | 9.02 | 11.04 | 51.92 | 55.08 | 11000000000 | 11000000000 | 6900000 | 53340.10 | 51.47 | 6.00 | 1.24 | 4.50 |
| 93 | 2005 | 10.44 | 10.75 | 53.96 | 56.17 | 12000000000 | 11000000000 | 7200000 | 54952.70 | 53.79 | 6.00 | 1.25 | 3.10 |
| 93 | 2006 | 10.17 | 11.00 | 54.63 | 54.71 | 13000000000 | 12000000000 | 7900000 | 57579.50 | 56.49 | 6.00 | 1.25 | 85.80 |
| 93 | 2007 | 10.17 | 11.08 | 55.29 | 53.96 | 15000000000 | 13000000000 | 8400000 | 63555.20 | 61.30 | 8.00 | 1.20 | 15.00 |
| 93 | 2008 | 11.44 | 11.58 | 56.38 | 54.92 | 18000000000 | 14000000000 | 8600000 | 72487.80 | 62.66 | 10.00 | 1.08 | 30.54 |
| 93 | 2009 | 14.94 | 13.33 | 56.63 | 59.92 | 17000000000 | 14000000000 | 8300000 | 69927.50 | 57.23 | 11.00 | 1.09 | 74.11 |
| 93 | 2010 | 13.06 | 14.00 | 55.29 | 56.83 | 18000000000 | 14000000000 | 8600000 | 74605.70 | 63.97 | 12.00 | 1.04 | 15.50 |
| 93 | 2011 | 12.35 | 13.33 | 55.58 | 55.79 | 21000000000 | 18000000000 | 8500000 | 88415.60 | 65.47 | 13.00 | 0.89 | 10.00 |
| 93 | 2012 | 11.81 | 12.29 | 54.71 | 56.63 | 20000000000 | 19000000000 | 8600000 | 83538.20 | 67.03 | 13.00 | 0.94 | 85.30 |
| 93 | 2013 | 10.79 | 12.00 | 53.79 | 55.79 | 20000000000 | 20000000000 | 9000000 | 85112.50 | 71.92 | 13.00 | 0.93 | 12.00 |
| 93 | 2014 | 10.46 | 12.00 | 53.88 | 55.04 | 21000000000 | 21000000000 | 9200000 | 86605.60 | 64.27 | 13.00 | 0.92 | 75.46 |
| 93 | 2015 | 11.5 | 12.13 | 54.63 | 56.25 | 20000000000 | 20000000000 | 9300000 | 82081.60 | 62.22 | 13.00 | 0.96 | 66.75 |
| 93 | 2016 | 11.73 | 12.33 | 54.63 | 56.50 | 20000000000 | 20000000000 | 9200000 | 80172.20 | 65.74 | 13.00 | 0.99 | 13.00 |
| 93 | 2017 | 11.06 | 12.38 | 54.50 | 55.25 | 20000000000 | 22000000000 | 9900000 | 80450.00 | 65.03 | 13.00 | 0.98 | 75.23 |
| 93 | 2018 | 10.96 | 12.42 | 54.75 | 54.75 | 21000000000 | 21000000000 | 10000000 | 82818.10 | 66.13 | 13.00 | 0.98 | 70.50 |
| 93 | 2019 | 11.62 | 12.67 | 55.08 | 55.50 | 21000000000 | 21000000000 | 7600000 | 51615.00 | 47.02 | 13.00 | 8.84 | 68.45 |
| 94 | 2000 | 24.92 | 28.83 | 57.50 | 63.50 | 440000000 | 220000000 | 795000 | 2001.54 | 26.83 | 1.00 | 8.76 | 16.50 |
| 94 | 2001 | 24.96 | 27.50 | 58.08 | 64.33 | 450000000 | 250000000 | 735000 | 2072.30 | 25.01 | 1.00 | 1.00 | 93.78 |
| 94 | 2002 | 28.25 | 34.38 | 57.75 | 64.38 | 520000000 | 270000000 | 798000 | 2124.10 | 25.51 | 1.00 | 1.00 | 49.76 |
| 94 | 2003 | 30.15 | 35.38 | 60.50 | 64.42 | 660000000 | 310000000 | 720000 | 2209.50 | 25.92 | 1.00 | 1.00 | 3.00 |
| 94 | 2004 | 29.79 | 32.25 | 62.25 | 65.08 | 750000000 | 370000000 | 951000 | 2278.43 | 25.91 | 1.00 | 1.00 | 68.91 |
| 94 | 2005 | 30.87 | 32.88 | 62.63 | 66.25 | 660000000 | 290000000 | 1100000 | 2428.57 | 25.27 | 1.00 | 1.00 | 5.05 |
| 94 | 2006 | 30.33 | 33.04 | 62.50 | 65.13 | 690000000 | 310000000 | 1300000 | 2631.82 | 25.94 | 1.00 | 1.00 | 57.06 |
| 94 | 2007 | 31.33 | 34.00 | 63.21 | 65.46 | 790000000 | 360000000 | 1300000 | 2786.16 | 25.57 | 1.00 | 1.00 | 3.50 |
| 94 | 2008 | 32.21 | 33.17 | 65.67 | 65.58 | 710000000 | 330000000 | 1400000 | 2933.40 | 26.74 | 1.00 | 1.00 | 71.40 |
| 94 | 2009 | 32.4 | 33.29 | 65.33 | 66.17 | 550000000 | 250000000 | 1100000 | 2858.48 | 23.95 | 1.00 | 1.00 | 17.70 |
| 94 | 2010 | 30.65 | 32.00 | 63.38 | 65.92 | 650000000 | 280000000 | 1200000 | 2983.24 | 26.95 | 1.00 | 1.00 | 6.63 |
| 94 | 2011 | 29.65 | 31.75 | 62.25 | 65.29 | 730000000 | 240000000 | 1200000 | 3266.05 | 28.98 | 1.00 | 1.00 | 39.20 |
| 94 | 2012 | 31.54 | 33.08 | 63.00 | 67.00 | 900000000 | 290000000 | 1300000 | 3428.38 | 28.57 | 1.00 | 1.00 | 8.00 |
| 94 | 2013 | 31.79 | 33.50 | 63.08 | 67.00 | 1100000000 | 270000000 | 1300000 | 3509.53 | 29.72 | 1.00 | 1.00 | 60.00 |
| 94 | 2014 | 32.6 | 34.88 | 63.83 | 66.50 | 1300000000 | 280000000 | 1300000 | 3589.04 | 29.55 | 1.00 | 1.00 | 56.00 |
| 94 | 2015 | 33.96 | 35.83 | 66.58 | 65.50 | 1200000000 | 330000000 | 1400000 | 3705.58 | 29.49 | 1.00 | 1.00 | 7.29 |
| 94 | 2016 | 32.23 | 36.58 | 63.04 | 64.83 | 1200000000 | 450000000 | 1400000 | 3805.99 | 28.46 | 1.00 | 1.00 | 43.46 |
| 94 | 2017 | 32.06 | 37.04 | 62.33 | 64.75 | 1200000000 | 470000000 | 1600000 | 3910.26 | 29.03 | 1.00 | 1.00 | 13.90 |
| 94 | 2018 | 31.79 | 36.50 | 62.83 | 64.25 | 1400000000 | 490000000 | 1700000 | 4067.66 | 28.78 | 1.00 | 1.00 | 46.50 |
| 94 | 2019 | 30.75 | 34.25 | 62.42 | 64.83 | 1700000000 | 540000000 | 12000000 | 81993.70 | 66.03 | 1.00 | 0.97 | 15.50 |
| 95 | 2000 | 8.48 | 51.00 | 59.96 | 36.63 | 170000000 | 110000000 | 258000 | 914.79 | 8.78 | 2.00 | 63.17 | 1.15 |
| 95 | 2001 | 12.23 | 49.21 | 60.75 | 40.75 | 190000000 | 120000000 | 285000 | 1727.28 | 20.69 | 2.00 | 66.91 | 19.10 |
| 95 | 2002 | 15.98 | 47.42 | 61.54 | 44.88 | 77000000 | 110000000 | 312000 | 2283.85 | 20.09 | 2.00 | 64.41 | 95.05 |
| 95 | 2003 | 19.73 | 45.63 | 62.33 | 49.00 | 160000000 | 140000000 | 339000 | 3005.43 | 23.15 | 2.00 | 57.59 | 62.10 |
| 95 | 2004 | 23.48 | 43.83 | 63.13 | 53.13 | 220000000 | 210000000 | 392000 | 3502.80 | 25.14 | 3.00 | 58.38 | 88.22 |
| 95 | 2005 | 27.23 | 42.04 | 63.92 | 57.25 | 310000000 | 260000000 | 453000 | 3720.48 | 27.97 | 3.00 | 66.72 | 94.78 |
| 95 | 2006 | 30.98 | 40.25 | 64.71 | 61.38 | 420000000 | 320000000 | 469000 | 4382.62 | 29.44 | 4.00 | 67.16 | 86.34 |
| 95 | 2007 | 34.73 | 38.46 | 65.50 | 65.50 | 1000000000 | 1200000000 | 696000 | 5848.48 | 27.29 | 5.00 | 58.45 | 23.11 |
| 95 | 2008 | 38.48 | 41.04 | 66.29 | 69.63 | 1100000000 | 1400000000 | 646000 | 7101.04 | 28.44 | 5.00 | 55.73 | 53.45 |
| 95 | 2009 | 40.15 | 39.75 | 69.38 | 71.17 | 990000000 | 1100000000 | 645000 | 6169.11 | 26.35 | 5.00 | 67.60 | 4.00 |
| 95 | 2010 | 41.54 | 38.46 | 70.42 | 74.21 | 950000000 | 1100000000 | 683000 | 5735.42 | 32.26 | 5.00 | 77.72 | 13.10 |
| 95 | 2011 | 37.83 | 39.92 | 67.00 | 68.75 | 1100000000 | 1300000000 | 764000 | 6809.16 | 33.00 | 5.00 | 73.36 | 65.45 |
| 95 | 2012 | 40.9 | 40.88 | 70.92 | 70.00 | 1100000000 | 1200000000 | 810000 | 6015.95 | 35.84 | 5.00 | 87.96 | 60.50 |
| 95 | 2013 | 38.96 | 41.50 | 66.00 | 70.42 | 1200000000 | 1300000000 | 922000 | 6755.07 | 39.85 | 5.00 | 85.16 | 21.90 |
| 95 | 2014 | 36.69 | 39.75 | 63.63 | 70.00 | 1400000000 | 1400000000 | 1000000 | 6600.06 | 42.08 | 5.00 | 88.41 | 77.88 |
| 95 | 2015 | 37.75 | 39.25 | 66.25 | 70.00 | 1300000000 | 1300000000 | 1100000 | 5585.12 | 45.27 | 5.00 | 108.81 | 72.68 |
| 95 | 2016 | 34.15 | 38.04 | 62.88 | 67.38 | 1500000000 | 1400000000 | 1300000 | 5756.38 | 48.62 | 6.00 | 111.28 | 22.70 |
| 95 | 2017 | 32.52 | 37.96 | 62.46 | 64.63 | 1700000000 | 1500000000 | 1500000 | 6284.19 | 50.54 | 6.00 | 107.76 | 37.40 |
| 95 | 2018 | 31.46 | 36.75 | 62.58 | 63.58 | 1900000000 | 1800000000 | 1700000 | 7246.19 | 50.78 | 6.00 | 100.18 | 28.94 |
| 95 | 2019 | 30.75 | 36.21 | 62.29 | 63.00 | 2000000000 | 2000000000 | 2600000 | 4187.25 | 29.54 | 6.00 | 1.00 | 4.40 |
| 96 | 2000 | 63.08 | 60.33 | 85.96 | 79.88 | 10000000 | 35000000 | 15800 | 138.70 | 18.13 | 4.00 | 2092.13 | 63.80 |
| 96 | 2001 | 56.16 | 55.13 | 82.13 | 75.08 | 14000000 | 42000000 | 24100 | 229.38 | 7.84 | 4.00 | 1986.15 | 43.80 |
| 96 | 2002 | 49.83 | 47.54 | 77.63 | 74.50 | 38000000 | 39000000 | 28400 | 252.40 | 8.55 | 4.00 | 2099.03 | 46.25 |
| 96 | 2003 | 48.44 | 44.08 | 78.29 | 74.50 | 60000000 | 38000000 | 38100 | 266.45 | 13.92 | 4.00 | 2347.94 | 19.70 |
| 96 | 2004 | 44.77 | 38.63 | 77.25 | 73.67 | 58000000 | 30000000 | 43600 | 266.57 | 16.47 | 4.00 | 2701.30 | 54.90 |
| 96 | 2005 | 43.62 | 37.13 | 78.13 | 72.00 | 64000000 | 34000000 | 40000 | 292.35 | 17.57 | 4.00 | 2889.59 | 64.50 |
| 96 | 2006 | 43.94 | 38.46 | 77.42 | 72.00 | 23000000 | 15000000 | 33700 | 323.39 | 16.87 | 5.00 | 2961.91 | 15.50 |
| 96 | 2007 | 40.33 | 38.58 | 73.75 | 68.33 | 22000000 | 17000000 | 32200 | 360.37 | 15.60 | 5.00 | 2985.19 | 40.95 |
| 96 | 2008 | 34.1 | 39.54 | 62.04 | 66.63 | 34000000 | 24000000 | 36000 | 408.48 | 13.52 | 5.00 | 2981.51 | 57.69 |
| 96 | 2009 | 36.79 | 39.50 | 64.75 | 69.33 | 25000000 | 26000000 | 37000 | 391.20 | 13.50 | 5.00 | 3385.65 | 71.64 |
| 96 | 2010 | 40.92 | 38.75 | 68.42 | 74.67 | 26000000 | 22000000 | 39000 | 401.84 | 16.80 | 5.00 | 3978.09 | 59.87 |
| 96 | 2011 | 36.79 | 40.13 | 62.46 | 71.00 | 44000000 | 26000000 | 52000 | 448.34 | 16.27 | 6.00 | 4349.16 | 74.20 |
| 96 | 2012 | 36.98 | 41.46 | 61.50 | 71.00 | 47000000 | 32000000 | 60000 | 566.38 | 32.91 | 7.00 | 4344.04 | 80.90 |
| 96 | 2013 | 37.08 | 42.17 | 61.63 | 70.38 | 66000000 | 71000000 | 81000 | 716.84 | 28.63 | 7.00 | 4332.50 | 66.00 |
| 96 | 2014 | 37.25 | 43.75 | 62.17 | 68.58 | 35000000 | 45000000 | 44000 | 714.70 | 30.76 | 7.00 | 4524.16 | 82.78 |
| 96 | 2015 | 39.58 | 45.04 | 63.38 | 70.75 | 37000000 | 34000000 | 24000 | 588.23 | 19.36 | 7.00 | 5080.75 | 72.64 |
| 96 | 2016 | 43.98 | 43.21 | 66.08 | 78.67 | 41000000 | 36000000 | 55000 | 501.42 | 24.91 | 7.00 | 6290.30 | 26.20 |
| 96 | 2017 | 41.96 | 42.96 | 71.25 | 69.71 | 39000000 | 56000000 | 51000 | 499.38 | 26.05 | 7.00 | 7384.43 | 37.10 |
| 96 | 2018 | 45.37 | 41.63 | 74.13 | 75.00 | 39000000 | 61000000 | 57000 | 533.99 | 17.47 | 7.00 | 7931.63 | 41.90 |
| 96 | 2019 | 42.37 | 40.04 | 70.17 | 74.54 | 39000000 | 66000000 | 1800000 | 7411.84 | 51.05 | 7.00 | 92.59 | 20.00 |
| 97 | 2000 | 36.9 | 42.08 | 68.21 | 63.50 | 150000000 | 130000000 | 424000 | 604.65 | 27.93 | 4.00 | 710.21 | 9.20 |
| 97 | 2001 | 34.45 | 39.04 | 65.25 | 64.63 | 180000000 | 110000000 | 493000 | 615.27 | 28.73 | 4.00 | 732.40 | 29.95 |
| 97 | 2002 | 34.02 | 39.08 | 64.13 | 64.83 | 210000000 | 110000000 | 562000 | 656.62 | 28.55 | 4.00 | 693.71 | 70.25 |
| 97 | 2003 | 35.04 | 40.33 | 64.75 | 65.00 | 270000000 | 130000000 | 631000 | 823.72 | 26.62 | 4.00 | 579.90 | 58.46 |
| 97 | 2004 | 35.25 | 41.29 | 64.21 | 65.00 | 290000000 | 140000000 | 700000 | 940.55 | 27.14 | 4.00 | 527.34 | 15.10 |
| 97 | 2005 | 35.21 | 40.96 | 64.21 | 65.25 | 330000000 | 140000000 | 769000 | 993.93 | 27.02 | 4.00 | 527.26 | 14.94 |
| 97 | 2006 | 35.52 | 41.25 | 64.29 | 65.50 | 330000000 | 140000000 | 866000 | 1040.90 | 25.63 | 5.00 | 522.43 | 89.84 |
| 97 | 2007 | 35.17 | 40.75 | 64.08 | 65.50 | 620000000 | 350000000 | 875000 | 1222.37 | 20.10 | 5.00 | 478.63 | 41.40 |
| 97 | 2008 | 37.04 | 41.50 | 65.58 | 67.00 | 640000000 | 280000000 | 867000 | 1411.93 | 20.64 | 5.00 | 446.00 | 38.50 |
| 97 | 2009 | 39.67 | 43.75 | 66.75 | 68.83 | 480000000 | 260000000 | 810000 | 1317.24 | 19.18 | 5.00 | 470.29 | 15.40 |
| 97 | 2010 | 39.98 | 44.54 | 66.13 | 69.29 | 460000000 | 220000000 | 900000 | 1280.23 | 19.83 | 5.00 | 494.79 | 58.00 |
| 97 | 2011 | 36.92 | 46.13 | 60.71 | 67.00 | 530000000 | 250000000 | 968000 | 1373.52 | 21.19 | 6.00 | 471.25 | 45.80 |
| 97 | 2012 | 37.67 | 44.96 | 62.38 | 68.00 | 470000000 | 250000000 | 962000 | 1329.98 | 22.26 | 7.00 | 510.56 | 60.10 |
| 97 | 2013 | 35.85 | 43.21 | 61.50 | 67.00 | 500000000 | 270000000 | 1100000 | 1376.07 | 22.20 | 7.00 | 493.90 | 29.50 |
| 97 | 2014 | 36.33 | 44.50 | 61.75 | 66.42 | 480000000 | 280000000 | 963000 | 1396.66 | 21.78 | 7.00 | 493.76 | 64.70 |
| 97 | 2015 | 38.21 | 44.83 | 64.63 | 66.96 | 420000000 | 260000000 | 1000000 | 1219.25 | 22.68 | 7.00 | 591.21 | 90.40 |
| 97 | 2016 | 36.92 | 44.46 | 63.21 | 66.17 | 440000000 | 280000000 | 1200000 | 1269.90 | 21.56 | 7.00 | 592.61 | 70.22 |
| 97 | 2017 | 36.87 | 43.83 | 65.58 | 64.33 | 470000000 | 300000000 | 1400000 | 1361.70 | 21.93 | 7.00 | 580.66 | 75.00 |
| 97 | 2018 | 35.83 | 42.33 | 64.92 | 64.42 | 560000000 | 320000000 | 1500000 | 1465.59 | 23.01 | 7.00 | 555.45 | 33.89 |
| 97 | 2019 | 36.71 | 41.75 | 67.08 | 64.58 | 640000000 | 350000000 | 1700000 | 9896.77 | 44.69 | 7.00 | 0.81 | 7.70 |
| 98 | 2000 | 21.44 | 24.67 | 58.00 | 60.21 | 2100000000 | 540000000 | 2700000 | 14388.30 | 69.96 | 3.00 | 0.62 | 83.49 |
| 98 | 2001 | 20.41 | 23.13 | 56.88 | 60.83 | 2200000000 | 570000000 | 2700000 | 14821.40 | 68.21 | 4.00 | 0.64 | 84.24 |
| 98 | 2002 | 18.87 | 22.08 | 56.67 | 59.00 | 2200000000 | 580000000 | 2400000 | 16093.20 | 62.41 | 4.00 | 0.61 | 21.40 |
| 98 | 2003 | 17.02 | 17.88 | 57.17 | 59.00 | 2300000000 | 700000000 | 2300000 | 20252.20 | 57.76 | 4.00 | 0.52 | 81.00 |
| 98 | 2004 | 16.69 | 17.50 | 56.71 | 59.17 | 2600000000 | 910000000 | 2300000 | 23792.60 | 56.89 | 4.00 | 0.47 | 84.00 |
| 98 | 2005 | 16.92 | 17.50 | 58.67 | 57.67 | 2600000000 | 1000000000 | 2500000 | 24959.30 | 55.69 | 4.00 | 0.46 | 6.50 |
| 98 | 2006 | 18.44 | 17.21 | 59.17 | 60.50 | 2700000000 | 1000000000 | 2400000 | 26729.30 | 53.44 | 4.00 | 0.46 | 76.92 |
| 98 | 2007 | 18.79 | 17.46 | 59.42 | 60.71 | 3100000000 | 1600000000 | 2400000 | 31244.90 | 53.26 | 4.00 | 0.43 | 12.00 |
| 98 | 2008 | 19.6 | 17.75 | 60.46 | 61.00 | 3200000000 | 2000000000 | 2400000 | 35397.40 | 50.08 | 4.00 | 0.54 | 43.00 |
| 98 | 2009 | 20.29 | 18.25 | 61.33 | 61.00 | 2500000000 | 1700000000 | 2100000 | 32109.20 | 48.72 | 4.00 | 0.64 | 90.50 |
| 98 | 2010 | 24.98 | 18.88 | 65.92 | 65.17 | 2100000000 | 1300000000 | 2200000 | 31023.60 | 50.38 | 4.00 | 0.65 | 44.92 |
| 98 | 2011 | 29.52 | 22.83 | 69.21 | 67.00 | 2600000000 | 1400000000 | 2400000 | 32396.40 | 53.50 | 4.00 | 0.62 | 54.55 |
| 98 | 2012 | 32.7 | 28.08 | 68.02 | 69.29 | 2600000000 | 1300000000 | 2500000 | 28912.20 | 55.31 | 4.00 | 0.63 | 59.02 |
| 98 | 2013 | 35.12 | 32.08 | 68.67 | 69.50 | 2900000000 | 1300000000 | 2400000 | 27729.20 | 61.36 | 4.00 | 0.64 | 55.49 |
| 98 | 2014 | 33.75 | 30.13 | 68.04 | 69.33 | 2900000000 | 1300000000 | 2400000 | 27129.60 | 66.11 | 4.00 | 0.61 | 85.00 |
| 98 | 2015 | 30.48 | 25.67 | 68.63 | 66.67 | 2500000000 | 1500000000 | 2700000 | 23333.70 | 70.33 | 4.00 | 0.65 | 98.16 |
| 98 | 2016 | 26.6 | 25.17 | 64.04 | 64.00 | 2900000000 | 1600000000 | 3200000 | 24532.50 | 70.75 | 4.00 | 0.74 | 66.60 |
| 98 | 2017 | 26.71 | 25.33 | 64.08 | 64.00 | 3300000000 | 1400000000 | 3700000 | 26338.70 | 73.02 | 4.00 | 0.78 | 49.92 |
| 98 | 2018 | 24.71 | 25.67 | 63.75 | 60.00 | 3400000000 | 1600000000 | 3900000 | 28689.70 | 73.07 | 4.00 | 0.75 | 36.74 |
| 98 | 2019 | 24.56 | 25.13 | 64.25 | 59.75 | 3200000000 | 1600000000 | 71000 | 527.53 | 15.99 | 4.00 | 8478.83 | 9.40 |
| 99 | 2000 | 26.75 | 34.08 | 57.63 | 61.79 | 3400000000 | 14000000000 | 6600000 | 9171.33 | 43.41 | 0.00 | 3.75 | 95.99 |
| 99 | 2001 | 21.39 | 31.08 | 54.38 | 57.33 | 2800000000 | 11000000000 | 6700000 | 8684.65 | 39.63 | 0.00 | 3.75 | 86.19 |
| 99 | 2002 | 26.27 | 31.50 | 56.63 | 64.42 | 4000000000 | 7400000000 | 7500000 | 8695.40 | 40.95 | 0.00 | 3.75 | 5.70 |
| 99 | 2003 | 23.44 | 33.13 | 54.63 | 59.13 | 3400000000 | 4200000000 | 7300000 | 9609.97 | 45.85 | 0.00 | 3.75 | 49.58 |
| 99 | 2004 | 21.83 | 33.29 | 54.00 | 56.38 | 4600000000 | 18000000000 | 8600000 | 11185.10 | 50.99 | 0.00 | 3.75 | 70.52 |
| 99 | 2005 | 18.37 | 32.13 | 52.50 | 52.13 | 4700000000 | 31000000000 | 8000000 | 13791.40 | 57.05 | 0.00 | 3.75 | 45.59 |
| 99 | 2006 | 18.17 | 31.71 | 52.63 | 52.00 | 5800000000 | 26000000000 | 8600000 | 15384.70 | 59.83 | 0.00 | 3.75 | 83.75 |
| 99 | 2007 | 18.75 | 31.08 | 53.29 | 53.13 | 6900000000 | 21000000000 | 12000000 | 16516.60 | 59.94 | 0.00 | 3.75 | 49.60 |
| 99 | 2008 | 19.81 | 30.88 | 54.38 | 54.38 | 6800000000 | 16000000000 | 15000000 | 20078.30 | 62.11 | 1.00 | 3.75 | 86.53 |
| 99 | 2009 | 25.17 | 29.38 | 56.92 | 64.04 | 6700000000 | 21000000000 | 11000000 | 16113.10 | 47.09 | 1.00 | 3.75 | 15.56 |
| 99 | 2010 | 22.56 | 30.29 | 55.63 | 59.21 | 7500000000 | 22000000000 | 11000000 | 19262.50 | 49.57 | 2.00 | 3.75 | 7.11 |
| 99 | 2011 | 19.42 | 32.00 | 52.25 | 54.58 | 9300000000 | 18000000000 | 14000000 | 23745.80 | 56.05 | 2.00 | 3.75 | 3.00 |
| 99 | 2012 | 18.94 | 32.21 | 52.00 | 53.67 | 8400000000 | 18000000000 | 16000000 | 25243.40 | 54.27 | 2.00 | 3.75 | 52.57 |
| 99 | 2013 | 19.29 | 32.42 | 51.92 | 54.25 | 8700000000 | 19000000000 | 16000000 | 24844.70 | 51.92 | 2.00 | 3.75 | 53.00 |
| 99 | 2014 | 20.42 | 34.50 | 51.83 | 54.50 | 9300000000 | 25000000000 | 18000000 | 24463.90 | 46.88 | 3.00 | 3.75 | 29.07 |
| 99 | 2015 | 25.04 | 34.13 | 53.71 | 62.25 | 11000000000 | 20000000000 | 18000000 | 20627.90 | 33.32 | 4.00 | 3.75 | 32.00 |
| 99 | 2016 | 30.54 | 34.63 | 57.08 | 69.38 | 13000000000 | 18000000000 | 18000000 | 19879.30 | 31.14 | 4.00 | 3.75 | 66.00 |
| 99 | 2017 | 29.04 | 36.17 | 55.33 | 66.58 | 15000000000 | 19000000000 | 16000000 | 20803.70 | 34.85 | 4.00 | 3.75 | 11.40 |
| 99 | 2018 | 26.37 | 35.17 | 54.58 | 63.00 | 17000000000 | 18000000000 | 15000000 | 23339.00 | 39.90 | 5.00 | 3.75 | 87.56 |
| 99 | 2019 | 24.42 | 33.54 | 54.38 | 60.92 | 20000000000 | 16000000000 | 15000000 | 1446.83 | 22.79 | 5.00 | 530.24 | 91.67 |
| 100 | 2000 | 40.29 | 48.08 | 64.50 | 68.00 | 390000000 | 380000000 | 400000 | 869.70 | 39.02 | 7.00 | 77.01 | 61.00 |
| 100 | 2001 | 40.75 | 45.25 | 66.75 | 69.50 | 350000000 | 400000000 | 337000 | 832.80 | 37.33 | 7.00 | 89.38 | 19.08 |
| 100 | 2002 | 36.83 | 38.71 | 64.54 | 70.42 | 590000000 | 440000000 | 393000 | 867.49 | 34.91 | 7.00 | 95.66 | 6.40 |
| 100 | 2003 | 36.69 | 40.79 | 63.42 | 69.17 | 710000000 | 460000000 | 501000 | 982.20 | 34.65 | 7.00 | 96.52 | 3.32 |
| 100 | 2004 | 38.62 | 45.96 | 62.96 | 68.33 | 810000000 | 500000000 | 566000 | 1065.78 | 35.33 | 7.00 | 101.19 | 87.12 |
| 100 | 2005 | 40.54 | 48.58 | 63.08 | 69.42 | 730000000 | 550000000 | 549000 | 1248.70 | 32.34 | 7.00 | 100.50 | 25.52 |
| 100 | 2006 | 38.67 | 46.17 | 63.67 | 67.50 | 730000000 | 670000000 | 560000 | 1435.82 | 30.13 | 7.00 | 103.91 | 38.07 |
| 100 | 2007 | 39.5 | 46.21 | 63.38 | 69.42 | 750000000 | 710000000 | 494000 | 1630.39 | 29.12 | 7.00 | 110.62 | 74.23 |
| 100 | 2008 | 37.1 | 46.50 | 61.54 | 66.17 | 800000000 | 780000000 | 438000 | 2037.32 | 24.84 | 7.00 | 108.33 | 16.36 |
| 100 | 2009 | 38.02 | 43.92 | 63.21 | 68.92 | 750000000 | 740000000 | 448000 | 2090.40 | 21.33 | 7.00 | 114.95 | 16.21 |
| 100 | 2010 | 35.96 | 41.54 | 61.96 | 68.42 | 1000000000 | 830000000 | 654000 | 2799.65 | 19.55 | 8.00 | 113.06 | 91.49 |
| 100 | 2011 | 35.9 | 42.29 | 62.00 | 67.50 | 1400000000 | 930000000 | 856000 | 3200.83 | 20.90 | 8.00 | 110.57 | 19.27 |
| 100 | 2012 | 39.06 | 45.00 | 65.67 | 67.46 | 1800000000 | 1200000000 | 1000000 | 3350.52 | 19.82 | 8.00 | 127.60 | 78.70 |
| 100 | 2013 | 38.6 | 45.92 | 64.42 | 66.88 | 2500000000 | 1800000000 | 1300000 | 3610.29 | 20.32 | 8.00 | 129.07 | 68.57 |
| 100 | 2014 | 37.54 | 44.92 | 64.00 | 66.17 | 3300000000 | 1900000000 | 1500000 | 3819.25 | 21.09 | 8.00 | 130.57 | 16.50 |
| 100 | 2015 | 34 | 40.58 | 63.42 | 64.00 | 4000000000 | 2200000000 | 1800000 | 3843.78 | 21.01 | 8.00 | 135.86 | 75.83 |
| 100 | 2016 | 34.58 | 40.71 | 64.00 | 64.46 | 4600000000 | 2300000000 | 2100000 | 3886.29 | 21.17 | 8.00 | 145.58 | 73.00 |
| 100 | 2017 | 36.06 | 42.79 | 63.42 | 65.92 | 5100000000 | 2400000000 | 2100000 | 4077.04 | 21.83 | 8.00 | 152.45 | 72.13 |
| 100 | 2018 | 35.42 | 41.00 | 63.79 | 66.04 | 5600000000 | 2500000000 | 2300000 | 4080.57 | 22.92 | 8.00 | 162.47 | 17.76 |
| 100 | 2019 | 35.83 | 41.21 | 64.08 | 66.38 | 4700000000 | 2400000000 | 4100000 | 27858.40 | 71.87 | 8.00 | 0.72 | 94.67 |
| 101 | 2000 | 28.15 | 23.58 | 63.38 | 69.33 | 440000000 | 340000000 | 1100000 | 5413.15 | 53.21 | 5.00 | 46.04 | 54.08 |
| 101 | 2001 | 26.08 | 22.00 | 63.04 | 67.13 | 650000000 | 340000000 | 1200000 | 5717.22 | 57.12 | 5.00 | 48.35 | 3.70 |
| 101 | 2002 | 25.9 | 23.67 | 62.38 | 65.75 | 740000000 | 510000000 | 1400000 | 6533.55 | 56.97 | 5.00 | 45.33 | 73.17 |
| 101 | 2003 | 25.25 | 21.75 | 63.17 | 65.58 | 880000000 | 660000000 | 1400000 | 8712.70 | 62.33 | 5.00 | 36.77 | 5.83 |
| 101 | 2004 | 24.71 | 24.00 | 61.21 | 64.21 | 930000000 | 900000000 | 1400000 | 10671.80 | 69.05 | 5.00 | 32.26 | 63.67 |
| 101 | 2005 | 24.65 | 23.21 | 62.63 | 63.46 | 1300000000 | 1100000000 | 1500000 | 11685.80 | 72.30 | 5.00 | 31.02 | 7.00 |
| 101 | 2006 | 24.92 | 23.50 | 63.25 | 63.08 | 1700000000 | 1200000000 | 1600000 | 13159.80 | 81.24 | 5.00 | 29.70 | 73.00 |
| 101 | 2007 | 23.44 | 21.13 | 63.92 | 61.83 | 2400000000 | 1800000000 | 1700000 | 16085.60 | 83.38 | 6.00 | 24.69 | 19.94 |
| 101 | 2008 | 23.12 | 20.79 | 64.17 | 61.29 | 3100000000 | 2700000000 | 1800000 | 18677.30 | 80.15 | 7.00 | 21.36 | 13.90 |
| 101 | 2009 | 26.04 | 22.67 | 63.71 | 65.71 | 2500000000 | 2300000000 | 1300000 | 16531.70 | 68.04 | 8.00 | 0.64 | 40.20 |
| 101 | 2010 | 28.92 | 23.75 | 63.08 | 71.00 | 2300000000 | 2100000000 | 1300000 | 16727.30 | 77.32 | 8.00 | 0.65 | 11.52 |
| 101 | 2011 | 26.29 | 25.46 | 63.21 | 63.92 | 2500000000 | 2500000000 | 1500000 | 18338.40 | 85.02 | 8.00 | 0.62 | 67.00 |
| 101 | 2012 | 27.69 | 25.33 | 65.42 | 64.63 | 2400000000 | 2300000000 | 1500000 | 17460.00 | 91.04 | 8.00 | 0.63 | 56.80 |
| 101 | 2013 | 26.58 | 26.17 | 64.50 | 62.50 | 2700000000 | 2500000000 | 1700000 | 18237.00 | 93.62 | 8.00 | 0.64 | 9.24 |
| 101 | 2014 | 25.23 | 26.54 | 64.21 | 59.71 | 2600000000 | 2600000000 | 1500000 | 18670.90 | 91.52 | 8.00 | 0.61 | 44.39 |
| 101 | 2015 | 27.6 | 27.58 | 67.54 | 60.08 | 2500000000 | 2300000000 | 1700000 | 16309.10 | 92.02 | 8.00 | 0.65 | 14.84 |
| 101 | 2016 | 25.69 | 26.50 | 64.75 | 60.13 | 2800000000 | 2400000000 | 2000000 | 16506.00 | 93.73 | 8.00 | 0.74 | 49.00 |
| 101 | 2017 | 24.94 | 25.79 | 64.58 | 59.50 | 3000000000 | 2600000000 | 2200000 | 17554.30 | 95.17 | 9.00 | 0.78 | 17.60 |
| 101 | 2018 | 25.67 | 26.00 | 65.50 | 59.83 | 3300000000 | 2800000000 | 2300000 | 19428.10 | 96.22 | 9.00 | 0.75 | 1.25 |
| 101 | 2019 | 25.1 | 24.71 | 66.25 | 59.25 | 3400000000 | 2800000000 | 20000000 | 23139.80 | 36.05 | 9.00 | 3.75 | 21.00 |
| 102 | 2000 | 22.83 | 20.17 | 62.13 | 63.38 | 1000000000 | 540000000 | 1100000 | 10201.30 | 50.14 | 1.00 | 222.66 | 96.30 |
| 102 | 2001 | 22.18 | 19.08 | 60.54 | 64.75 | 1100000000 | 560000000 | 1200000 | 10479.80 | 51.75 | 1.00 | 242.75 | 64.59 |
| 102 | 2002 | 20.75 | 19.54 | 58.96 | 63.00 | 1200000000 | 650000000 | 1300000 | 11777.20 | 52.32 | 1.00 | 240.25 | 89.11 |
| 102 | 2003 | 20.19 | 19.25 | 59.13 | 62.00 | 1400000000 | 810000000 | 1400000 | 14849.00 | 51.00 | 1.00 | 207.11 | 92.52 |
| 102 | 2004 | 20.62 | 20.04 | 60.38 | 60.83 | 1700000000 | 940000000 | 1500000 | 17233.10 | 55.05 | 1.00 | 192.38 | 87.40 |
| 102 | 2005 | 20.19 | 18.58 | 61.71 | 60.08 | 1900000000 | 1000000000 | 1600000 | 18098.90 | 59.81 | 1.00 | 192.71 | 24.76 |
| 102 | 2006 | 20.94 | 19.46 | 61.92 | 60.50 | 2100000000 | 1100000000 | 1600000 | 19673.00 | 64.87 | 1.00 | 191.03 | 62.08 |
| 102 | 2007 | 22.42 | 20.92 | 63.42 | 60.50 | 2500000000 | 1300000000 | 1800000 | 23787.60 | 67.90 | 2.00 | 0.50 | 6.06 |
| 102 | 2008 | 24.29 | 22.58 | 64.50 | 61.50 | 3000000000 | 1600000000 | 2000000 | 27483.30 | 66.31 | 2.00 | 0.54 | 17.70 |
| 102 | 2009 | 25.29 | 21.38 | 65.54 | 63.67 | 2800000000 | 1800000000 | 1800000 | 24694.20 | 57.26 | 2.00 | 0.64 | 69.33 |
| 102 | 2010 | 28.6 | 22.17 | 65.58 | 69.46 | 2800000000 | 1600000000 | 2000000 | 23509.50 | 64.27 | 2.00 | 0.65 | 64.71 |
| 102 | 2011 | 27.04 | 25.13 | 66.21 | 62.75 | 3000000000 | 1700000000 | 2200000 | 25095.10 | 70.25 | 4.00 | 0.62 | 25.80 |
| 102 | 2012 | 31.62 | 26.71 | 71.29 | 65.25 | 2800000000 | 1500000000 | 2400000 | 22643.10 | 72.89 | 5.00 | 0.63 | 79.98 |
| 102 | 2013 | 33.62 | 30.21 | 70.00 | 67.04 | 3000000000 | 1600000000 | 2500000 | 23496.60 | 74.22 | 5.00 | 0.64 | 71.59 |
| 102 | 2014 | 32.54 | 28.38 | 70.21 | 66.50 | 3100000000 | 1600000000 | 2700000 | 24214.90 | 76.15 | 5.00 | 0.61 | 24.64 |
| 102 | 2015 | 31.27 | 29.46 | 71.96 | 61.13 | 2600000000 | 1400000000 | 3000000 | 20881.80 | 77.15 | 5.00 | 0.65 | 40.08 |
| 102 | 2016 | 27.19 | 28.58 | 65.96 | 59.83 | 2700000000 | 1500000000 | 3400000 | 21622.60 | 77.97 | 5.00 | 0.74 | 34.89 |
| 102 | 2017 | 25.79 | 27.79 | 65.29 | 58.50 | 3100000000 | 1600000000 | 4000000 | 23500.80 | 82.96 | 6.00 | 0.78 | 7.00 |
| 102 | 2018 | 24.87 | 28.17 | 65.25 | 56.33 | 3400000000 | 1900000000 | 4400000 | 26054.50 | 84.77 | 6.00 | 0.75 | 65.10 |
| 102 | 2019 | 23.44 | 26.63 | 65.25 | 55.00 | 3200000000 | 1800000000 | 2000000 | 3853.08 | 23.12 | 6.00 | 172.48 | 46.16 |
| 103 | 2000 | 52.35 | 66.17 | 72.04 | 66.50 | 5000000 | 55000000 | 38000 | 366.17 | 15.98 | 0.00 | 2.57 | 51.04 |
| 103 | 2001 | 47.19 | 59.58 | 68.67 | 66.13 | 3000000 | 74000000 | 50000 | 383.02 | 11.40 | 0.00 | 2.59 | 23.40 |
| 103 | 2002 | 45.81 | 56.54 | 69.08 | 66.00 | 110000000 | 91000000 | 52000 | 418.25 | 13.98 | 0.00 | 2.63 | 57.00 |
| 103 | 2003 | 45.33 | 54.92 | 69.83 | 65.92 | 17000000 | 120000000 | 52000 | 484.85 | 14.83 | 1.00 | 2.61 | 68.77 |
| 103 | 2004 | 43.31 | 51.04 | 70.29 | 65.29 | 21000000 | 180000000 | 61000 | 573.31 | 17.76 | 1.00 | 2.58 | 16.90 |
| 103 | 2005 | 43.1 | 53.00 | 68.54 | 64.67 | 110000000 | 670000000 | 246000 | 689.21 | 19.18 | 1.00 | 2.44 | 46.24 |
| 103 | 2006 | 42.81 | 54.00 | 67.88 | 63.75 | 190000000 | 1400000000 | 328000 | 905.31 | 19.07 | 1.00 | 2.17 | 61.46 |
| 103 | 2007 | 43.81 | 55.29 | 68.13 | 64.21 | 390000000 | 1500000000 | 436000 | 1128.41 | 21.89 | 1.00 | 2.02 | 76.19 |
| 103 | 2008 | 45.04 | 56.17 | 66.92 | 67.00 | 260000000 | 1300000000 | 440000 | 1304.53 | 24.10 | 1.00 | 2.09 | 63.21 |
| 103 | 2009 | 46.19 | 57.21 | 65.17 | 70.00 | 230000000 | 870000000 | 420000 | 1163.80 | 15.97 | 1.00 | 2.30 | 79.87 |
| 103 | 2010 | 47.79 | 59.33 | 66.88 | 69.38 | 82000000 | 1100000000 | 495000 | 1401.48 | 17.13 | 1.00 | 2.31 | 79.03 |
| 103 | 2011 | 49.83 | 60.58 | 68.54 | 70.54 | 180000000 | 940000000 | 536000 | 1437.77 | 14.32 | 2.00 | 2.67 | 70.00 |
| 103 | 2012 | 54.35 | 62.63 | 69.29 | 76.79 | 770000000 | 700000000 | 575000 | 1457.89 | 9.22 | 2.00 | 3.57 | 85.50 |
| 103 | 2013 | 56.21 | 61.92 | 72.17 | 78.33 | 770000000 | 460000000 | 591000 | 1557.23 | 8.84 | 2.00 | 4.76 | 75.65 |
| 103 | 2014 | 53.35 | 64.21 | 71.00 | 71.50 | 970000000 | 440000000 | 684000 | 1710.00 | 8.15 | 2.00 | 5.74 | 29.98 |
| 103 | 2015 | 52.4 | 64.58 | 69.67 | 70.54 | 950000000 | 200000000 | 741000 | 1909.74 | 8.18 | 2.00 | 6.03 | 40.40 |
| 103 | 2016 | 51.77 | 65.00 | 69.67 | 68.88 | 1000000000 | 200000000 | 800000 | 1299.26 | 9.83 | 3.00 | 6.21 | 54.62 |
| 103 | 2017 | 51.92 | 62.96 | 70.58 | 70.29 | 1000000000 | 29000000 | 813000 | 1111.87 | 9.69 | 3.00 | 6.68 | 22.55 |
| 103 | 2018 | 57.21 | 62.50 | 80.33 | 71.58 | 1000000000 | 13000000 | 836000 | 623.87 | 10.25 | 3.00 | 24.33 | 13.21 |
| 103 | 2019 | 60.77 | 60.79 | 81.50 | 79.25 | 1100000000 | 21000000 | 859000 | 19266.30 | 92.44 | 3.00 | 0.72 | 39.35 |
| 104 | 2000 | 37.98 | 37.75 | 68.71 | 69.50 | 42000000 | 67000000 | 57000 | 2012.26 | 44.49 | 1.00 | 1.32 | 75.02 |
| 104 | 2001 | 35.46 | 32.04 | 66.67 | 72.21 | 26000000 | 62000000 | 54000 | 1750.56 | 45.86 | 1.00 | 2.18 | 55.64 |
| 104 | 2002 | 37.37 | 34.63 | 72.63 | 67.50 | 17000000 | 54000000 | 60000 | 2267.72 | 47.24 | 1.00 | 2.35 | 21.00 |
| 104 | 2003 | 35.56 | 34.83 | 64.58 | 71.71 | 18000000 | 68000000 | 82000 | 2611.36 | 48.62 | 2.00 | 2.60 | 17.14 |
| 104 | 2004 | 33.12 | 35.50 | 61.25 | 69.50 | 52000000 | 85000000 | 138000 | 3006.19 | 49.99 | 2.00 | 2.73 | 91.61 |
| 104 | 2005 | 33.08 | 37.00 | 61.83 | 67.33 | 96000000 | 94000000 | 161000 | 3590.63 | 51.37 | 2.00 | 2.73 | 46.20 |
| 104 | 2006 | 32.65 | 36.79 | 61.71 | 66.79 | 110000000 | 33000000 | 154000 | 5197.72 | 52.75 | 2.00 | 2.74 | 41.00 |
| 104 | 2007 | 30.5 | 37.00 | 59.08 | 64.92 | 73000000 | 28000000 | 167000 | 5744.76 | 54.12 | 2.00 | 2.75 | 19.00 |
| 104 | 2008 | 30.25 | 37.00 | 59.50 | 64.00 | 83000000 | 35000000 | 151000 | 6831.97 | 56.91 | 2.00 | 2.75 | 61.11 |
| 104 | 2009 | 31 | 37.00 | 59.63 | 65.38 | 70000000 | 35000000 | 151000 | 7408.39 | 44.12 | 2.00 | 2.75 | 47.90 |
| 104 | 2010 | 28.62 | 37.00 | 55.88 | 64.38 | 69000000 | 41000000 | 205000 | 8255.80 | 52.55 | 2.00 | 2.75 | 63.25 |
| 104 | 2011 | 29.81 | 36.88 | 58.54 | 64.21 | 69000000 | 49000000 | 220000 | 8263.17 | 60.97 | 2.00 | 3.27 | 38.20 |
| 104 | 2012 | 27.04 | 35.92 | 54.25 | 63.92 | 79000000 | 58000000 | 240000 | 9201.01 | 69.39 | 2.00 | 3.30 | 68.04 |
| 104 | 2013 | 26.5 | 36.00 | 55.75 | 61.25 | 92000000 | 75000000 | 249000 | 9402.23 | 77.81 | 2.00 | 3.30 | 90.50 |
| 104 | 2014 | 27.85 | 36.00 | 56.79 | 62.92 | 100000000 | 90000000 | 252000 | 9472.01 | 86.24 | 2.00 | 3.30 | 73.53 |
| 104 | 2015 | 29.71 | 36.00 | 59.38 | 64.04 | 99000000 | 130000000 | 228000 | 8561.97 | 94.66 | 2.00 | 3.42 | 77.00 |
| 104 | 2016 | 37.79 | 36.00 | 70.00 | 69.58 | 74000000 | 110000000 | 256000 | 5539.07 | 103.08 | 2.00 | 6.23 | 37.82 |
| 104 | 2017 | 37.27 | 35.58 | 64.25 | 74.71 | 61000000 | 99000000 | 278000 | 5626.80 | 111.51 | 2.00 | 7.49 | 13.86 |
| 104 | 2018 | 34.31 | 35.63 | 64.25 | 68.75 | 73000000 | 100000000 | 300000 | 6003.72 | 119.93 | 2.00 | 7.46 | 83.49 |
| 104 | 2019 | 34.17 | 36.21 | 64.75 | 67.38 | 64000000 | 94000000 | 4700000 | 25946.20 | 83.74 | 2.00 | 0.72 | 88.41 |
| 105 | 2000 | 25.44 | 28.08 | 61.54 | 61.25 | 9900000000 | 3200000000 | 9600000 | 2007.74 | 64.84 | 4.00 | 40.11 | 12.40 |
| 105 | 2001 | 25.87 | 27.96 | 61.88 | 61.92 | 9400000000 | 3300000000 | 10000000 | 1893.26 | 63.25 | 4.00 | 44.43 | 83.94 |
| 105 | 2002 | 24.75 | 27.58 | 60.04 | 61.88 | 10000000000 | 3900000000 | 11000000 | 2096.19 | 60.65 | 4.00 | 42.96 | 84.56 |
| 105 | 2003 | 24.98 | 29.00 | 60.38 | 60.58 | 10000000000 | 3500000000 | 10000000 | 2359.12 | 61.52 | 4.00 | 41.48 | 7.90 |
| 105 | 2004 | 24.35 | 30.71 | 59.13 | 58.88 | 13000000000 | 5300000000 | 12000000 | 2660.13 | 65.97 | 4.00 | 40.22 | 78.00 |
| 105 | 2005 | 27.02 | 34.75 | 58.21 | 61.08 | 12000000000 | 4900000000 | 12000000 | 2894.06 | 68.40 | 5.00 | 40.22 | 14.00 |
| 105 | 2006 | 30.52 | 39.71 | 59.00 | 62.33 | 17000000000 | 6200000000 | 14000000 | 3369.54 | 68.68 | 5.00 | 37.88 | 49.72 |
| 105 | 2007 | 31.94 | 43.63 | 58.50 | 61.75 | 21000000000 | 6900000000 | 14000000 | 3973.02 | 68.87 | 5.00 | 34.52 | 93.48 |
| 105 | 2008 | 31.46 | 41.72 | 58.63 | 62.58 | 23000000000 | 6700000000 | 15000000 | 4379.66 | 71.42 | 5.00 | 33.31 | 51.21 |
| 105 | 2009 | 34.44 | 42.50 | 58.33 | 68.04 | 20000000000 | 5700000000 | 14000000 | 4213.01 | 64.44 | 5.00 | 34.29 | 58.33 |
| 105 | 2010 | 31.83 | 44.33 | 56.17 | 63.17 | 24000000000 | 7200000000 | 16000000 | 5076.34 | 66.49 | 5.00 | 31.69 | 62.23 |
| 105 | 2011 | 29.87 | 42.83 | 56.25 | 60.67 | 31000000000 | 7500000000 | 19000000 | 5492.12 | 70.85 | 5.00 | 30.49 | 56.66 |
| 105 | 2012 | 30.12 | 41.17 | 56.96 | 62.13 | 35000000000 | 8100000000 | 22000000 | 5860.58 | 68.95 | 5.00 | 31.08 | 85.05 |
| 105 | 2013 | 31.4 | 42.71 | 57.50 | 62.58 | 42000000000 | 8200000000 | 27000000 | 6168.26 | 67.17 | 5.00 | 30.73 | 98.20 |
| 105 | 2014 | 32.87 | 45.79 | 57.79 | 62.17 | 38000000000 | 8800000000 | 25000000 | 5951.88 | 68.39 | 5.00 | 32.48 | 68.00 |
| 105 | 2015 | 32.33 | 45.00 | 58.13 | 61.54 | 45000000000 | 9500000000 | 30000000 | 5840.05 | 67.64 | 5.00 | 34.25 | 52.60 |
| 105 | 2016 | 31.02 | 44.38 | 57.25 | 60.42 | 48000000000 | 11000000000 | 33000000 | 5994.23 | 67.06 | 5.00 | 35.30 | 37.31 |
| 105 | 2017 | 29.23 | 44.33 | 55.46 | 58.67 | 57000000000 | 13000000000 | 36000000 | 6592.92 | 66.68 | 5.00 | 33.94 | 14.00 |
| 105 | 2018 | 28.48 | 43.50 | 55.54 | 57.92 | 61000000000 | 14000000000 | 38000000 | 7295.48 | 64.87 | 5.00 | 32.31 | 96.33 |
| 105 | 2019 | 28.17 | 42.38 | 55.50 | 58.46 | 65000000000 | 17000000000 | 41000000 | 441.51 | 7.67 | 5.00 | 41.97 | 87.59 |
| 106 | 2000 | 40.5 | 35.67 | 77.96 | 67.38 | 380000000 | 370000000 | 459000 | 410.95 | 10.81 | 6.00 | 800.41 | 7.12 |
| 106 | 2001 | 40.89 | 37.04 | 79.25 | 65.50 | 630000000 | 360000000 | 501000 | 406.54 | 13.83 | 6.00 | 876.41 | 54.22 |
| 106 | 2002 | 42.65 | 39.88 | 79.92 | 65.50 | 640000000 | 360000000 | 550000 | 411.97 | 14.28 | 6.00 | 966.58 | 70.10 |
| 106 | 2003 | 42.08 | 39.54 | 79.13 | 65.50 | 650000000 | 380000000 | 552000 | 431.28 | 15.09 | 6.00 | 1038.42 | 48.94 |
| 106 | 2004 | 40.04 | 38.88 | 75.00 | 66.21 | 760000000 | 470000000 | 566000 | 459.26 | 16.04 | 6.00 | 1089.33 | 78.01 |
| 106 | 2005 | 36.98 | 38.13 | 68.33 | 67.50 | 840000000 | 580000000 | 590000 | 492.63 | 16.98 | 6.00 | 1128.93 | 36.00 |
| 106 | 2006 | 37.06 | 37.42 | 69.21 | 67.50 | 990000000 | 570000000 | 622000 | 485.50 | 18.42 | 7.00 | 1251.90 | 86.42 |
| 106 | 2007 | 34.58 | 37.13 | 65.92 | 66.13 | 1200000000 | 620000000 | 692000 | 552.84 | 19.83 | 7.00 | 1245.04 | 16.50 |
| 106 | 2008 | 33.65 | 36.46 | 61.83 | 69.00 | 1300000000 | 750000000 | 750000 | 687.39 | 19.46 | 7.00 | 1196.31 | 7.62 |
| 106 | 2009 | 34.81 | 36.29 | 62.71 | 70.63 | 1200000000 | 810000000 | 695000 | 695.22 | 18.19 | 7.00 | 1320.31 | 3.80 |
| 106 | 2010 | 37.08 | 36.71 | 62.46 | 75.00 | 1300000000 | 860000000 | 754000 | 743.40 | 19.61 | 8.00 | 1395.63 | 55.90 |
| 106 | 2011 | 33.98 | 36.33 | 62.13 | 69.50 | 1400000000 | 930000000 | 843000 | 781.44 | 21.63 | 8.00 | 1557.43 | 59.76 |
| 106 | 2012 | 34.27 | 37.92 | 61.13 | 69.50 | 1800000000 | 1000000000 | 1000000 | 867.87 | 22.37 | 8.00 | 1571.70 | 37.31 |
| 106 | 2013 | 35.23 | 39.08 | 61.75 | 69.63 | 1900000000 | 1100000000 | 1100000 | 970.42 | 19.01 | 8.00 | 1597.56 | 34.00 |
| 106 | 2014 | 37.83 | 41.54 | 64.21 | 69.92 | 2000000000 | 1200000000 | 1100000 | 1030.09 | 18.07 | 8.00 | 1653.23 | 70.83 |
| 106 | 2015 | 38.9 | 41.00 | 67.88 | 68.92 | 1900000000 | 1300000000 | 1100000 | 947.93 | 17.10 | 8.00 | 1991.39 | 12.20 |
| 106 | 2016 | 36.35 | 40.33 | 63.88 | 68.50 | 2100000000 | 990000000 | 1200000 | 966.48 | 16.35 | 8.00 | 2177.09 | 89.90 |
| 106 | 2017 | 36.58 | 42.21 | 62.75 | 68.21 | 2300000000 | 870000000 | 1300000 | 1004.84 | 15.14 | 8.00 | 2228.86 | 91.72 |
| 106 | 2018 | 35.19 | 42.38 | 61.67 | 66.33 | 2500000000 | 820000000 | 1400000 | 1060.99 | 13.94 | 8.00 | 2263.78 | 68.12 |
| 106 | 2019 | 34.12 | 41.21 | 61.96 | 65.08 | 2600000000 | 740000000 | 1500000 | 6359.83 | 128.35 | 8.00 | 7.44 | 27.10 |
| 107 | 2000 | 25.75 | 28.00 | 60.50 | 63.00 | 370000000 | 190000000 | 399000 | 6435.16 | 29.74 | 3.00 | 6.30 | 8.20 |
| 107 | 2001 | 25.48 | 28.17 | 60.50 | 62.29 | 360000000 | 170000000 | 383000 | 6939.80 | 22.74 | 3.00 | 6.23 | 3.54 |
| 107 | 2002 | 27.92 | 34.96 | 59.96 | 60.92 | 400000000 | 210000000 | 384000 | 7053.07 | 30.43 | 3.00 | 6.25 | 90.00 |
| 107 | 2003 | 24.48 | 33.50 | 56.25 | 59.21 | 440000000 | 140000000 | 409000 | 8807.90 | 33.85 | 3.00 | 6.30 | 25.00 |
| 107 | 2004 | 21.96 | 29.96 | 55.46 | 58.50 | 570000000 | 140000000 | 443000 | 10293.80 | 36.20 | 3.00 | 6.30 | 45.78 |
| 107 | 2005 | 19.21 | 27.29 | 54.63 | 56.50 | 590000000 | 230000000 | 463000 | 12327.20 | 39.66 | 3.00 | 6.30 | 75.67 |
| 107 | 2006 | 17.83 | 27.71 | 53.25 | 54.71 | 520000000 | 150000000 | 461000 | 14102.50 | 36.52 | 3.00 | 6.31 | 22.74 |
| 107 | 2007 | 18.81 | 28.13 | 54.17 | 55.33 | 620000000 | 160000000 | 449000 | 16539.90 | 31.13 | 3.00 | 6.33 | 20.68 |
| 107 | 2008 | 18.81 | 28.00 | 54.00 | 55.63 | 560000000 | 100000000 | 437000 | 21204.10 | 30.82 | 3.00 | 6.29 | 92.88 |
| 107 | 2009 | 20.08 | 28.00 | 52.17 | 60.00 | 550000000 | 140000000 | 419000 | 14514.10 | 18.07 | 3.00 | 6.32 | 38.44 |
| 107 | 2010 | 21.56 | 27.42 | 52.50 | 63.21 | 630000000 | 97000000 | 388000 | 16683.40 | 28.53 | 3.00 | 6.38 | 72.00 |
| 107 | 2011 | 21.65 | 27.00 | 54.17 | 62.13 | 650000000 | 190000000 | 431000 | 19034.10 | 29.94 | 3.00 | 6.41 | 69.85 |
| 107 | 2012 | 21.77 | 28.63 | 53.25 | 61.67 | 850000000 | 220000000 | 455000 | 19157.40 | 26.17 | 3.00 | 6.43 | 16.59 |
| 107 | 2013 | 23.1 | 31.71 | 53.50 | 61.00 | 860000000 | 120000000 | 434000 | 20143.70 | 24.77 | 3.00 | 6.44 | 79.20 |
| 107 | 2014 | 22.85 | 31.13 | 54.00 | 60.58 | 880000000 | 170000000 | 412000 | 20270.90 | 16.69 | 3.00 | 6.41 | 74.00 |
| 107 | 2015 | 24 | 32.83 | 53.46 | 61.71 | 810000000 | 250000000 | 440000 | 18289.70 | 15.19 | 3.00 | 6.38 | 71.38 |
| 107 | 2016 | 27.75 | 33.17 | 55.08 | 67.25 | 710000000 | 230000000 | 409000 | 16176.90 | 14.69 | 3.00 | 6.67 | 19.02 |
| 107 | 2017 | 30.67 | 34.29 | 56.54 | 70.50 | 720000000 | 150000000 | 395000 | 16238.20 | 13.39 | 3.00 | 6.78 | 96.38 |
| 107 | 2018 | 27.94 | 35.25 | 54.75 | 65.88 | 540000000 | 150000000 | 375000 | 17129.90 | 12.39 | 3.00 | 6.77 | 55.76 |
| 107 | 2019 | 25.81 | 35.00 | 54.25 | 62.38 | 480000000 | 130000000 | 40000000 | 7806.74 | 59.77 | 3.00 | 30.68 | 4.17 |
| 108 | 2000 | 27 | 25.00 | 66.21 | 62.79 | 2000000000 | 3400000000 | 5100000 | 2211.83 | 39.55 | 9.00 | 1.37 | 75.96 |
| 108 | 2001 | 26.48 | 26.17 | 64.17 | 62.63 | 2100000000 | 3500000000 | 5400000 | 2253.04 | 42.82 | 9.00 | 1.44 | 5.30 |
| 108 | 2002 | 28 | 28.79 | 63.83 | 63.38 | 1800000000 | 3500000000 | 5100000 | 2344.41 | 40.78 | 9.00 | 1.42 | 71.06 |
| 108 | 2003 | 27.31 | 27.71 | 63.67 | 63.25 | 1900000000 | 3500000000 | 5100000 | 2760.41 | 39.48 | 9.00 | 1.29 | 10.33 |
| 108 | 2004 | 26.29 | 26.83 | 62.92 | 62.83 | 2400000000 | 3500000000 | 6000000 | 3111.38 | 42.22 | 9.00 | 1.25 | 74.55 |
| 108 | 2005 | 26.48 | 27.04 | 63.00 | 62.92 | 2800000000 | 3600000000 | 6400000 | 3193.21 | 44.93 | 9.00 | 1.30 | 22.50 |
| 108 | 2006 | 26.31 | 27.08 | 62.67 | 62.88 | 3000000000 | 3500000000 | 6600000 | 3370.03 | 46.03 | 9.00 | 1.33 | 14.40 |
| 108 | 2007 | 26.73 | 27.75 | 62.58 | 63.13 | 3400000000 | 4300000000 | 6800000 | 3775.75 | 51.09 | 9.00 | 1.28 | 40.85 |
| 108 | 2008 | 28.08 | 27.92 | 63.96 | 64.29 | 3900000000 | 4500000000 | 7100000 | 4307.16 | 55.66 | 9.00 | 1.23 | 21.73 |
| 108 | 2009 | 29.02 | 28.00 | 62.92 | 67.13 | 3500000000 | 5100000000 | 7800000 | 4128.46 | 45.04 | 9.00 | 1.35 | 69.00 |
| 108 | 2010 | 27.65 | 27.50 | 60.96 | 66.83 | 3500000000 | 5800000000 | 7800000 | 4141.98 | 49.50 | 9.00 | 1.43 | 57.08 |
| 108 | 2011 | 34.33 | 36.00 | 61.54 | 71.13 | 2500000000 | 5400000000 | 5700000 | 4264.67 | 48.53 | 9.00 | 1.41 | 16.93 |
| 108 | 2012 | 35.4 | 35.42 | 64.38 | 71.00 | 2900000000 | 4600000000 | 7000000 | 4152.68 | 48.55 | 9.00 | 1.56 | 57.43 |
| 108 | 2013 | 36.33 | 39.13 | 64.42 | 69.13 | 2900000000 | 5000000000 | 7400000 | 4222.70 | 46.97 | 9.00 | 1.62 | 25.69 |
| 108 | 2014 | 36.79 | 40.00 | 64.75 | 68.83 | 3000000000 | 5300000000 | 7200000 | 4305.47 | 44.92 | 9.00 | 1.70 | 51.92 |
| 108 | 2015 | 35.75 | 37.25 | 65.58 | 68.67 | 1900000000 | 5600000000 | 5400000 | 3861.69 | 40.64 | 9.00 | 1.96 | 19.70 |
| 108 | 2016 | 34.81 | 36.79 | 64.29 | 68.54 | 1700000000 | 5700000000 | 5700000 | 3697.93 | 40.64 | 9.00 | 2.15 | 2.48 |
| 108 | 2017 | 35.81 | 36.25 | 66.29 | 69.08 | 1800000000 | 5900000000 | 7100000 | 3481.23 | 44.39 | 9.00 | 2.42 | 36.00 |
| 108 | 2018 | 37.19 | 37.79 | 67.04 | 69.54 | 2300000000 | 6100000000 | 8300000 | 3438.79 | 49.04 | 9.00 | 2.65 | 96.81 |
| 108 | 2019 | 38 | 36.75 | 68.33 | 70.92 | 2700000000 | 4900000000 | 1500000 | 1122.12 | 12.74 | 9.00 | 2298.71 | 68.63 |
| 109 | 2000 | 43.6 | 40.92 | 72.75 | 73.54 | 17000000000 | 310000000 | 9600000 | 4316.55 | 39.55 | 9.00 | 0.63 | 91.06 |
| 109 | 2001 | 47.35 | 43.58 | 74.42 | 76.71 | 18000000000 | 320000000 | 11000000 | 3119.57 | 42.82 | 9.00 | 1.23 | 90.61 |
| 109 | 2002 | 42.42 | 42.21 | 69.42 | 73.21 | 18000000000 | 300000000 | 13000000 | 3659.94 | 40.78 | 9.00 | 1.51 | 87.48 |
| 109 | 2003 | 40.44 | 38.33 | 69.46 | 73.08 | 19000000000 | 360000000 | 13000000 | 4718.20 | 39.48 | 9.00 | 1.50 | 26.80 |
| 109 | 2004 | 35.73 | 33.04 | 67.71 | 70.71 | 20000000000 | 430000000 | 17000000 | 6040.61 | 42.22 | 9.00 | 1.43 | 65.32 |
| 109 | 2005 | 32.1 | 30.67 | 66.75 | 66.79 | 21000000000 | 450000000 | 20000000 | 7384.25 | 44.93 | 9.00 | 1.34 | 6.34 |
| 109 | 2006 | 33.62 | 34.17 | 66.75 | 66.33 | 19000000000 | 500000000 | 19000000 | 8035.38 | 46.03 | 9.00 | 1.43 | 27.00 |
| 109 | 2007 | 34.73 | 36.96 | 67.17 | 65.33 | 22000000000 | 530000000 | 26000000 | 9711.87 | 51.09 | 9.00 | 1.30 | 71.72 |
| 109 | 2008 | 36.67 | 39.50 | 69.00 | 64.83 | 26000000000 | 560000000 | 30000000 | 10854.20 | 55.66 | 9.00 | 1.30 | 69.62 |
| 109 | 2009 | 39.1 | 40.79 | 68.96 | 68.46 | 26000000000 | 480000000 | 30000000 | 9038.52 | 45.04 | 9.00 | 1.55 | 12.10 |
| 109 | 2010 | 36.67 | 42.63 | 65.71 | 65.00 | 26000000000 | 610000000 | 31000000 | 10672.40 | 49.50 | 9.00 | 1.50 | 77.63 |
| 109 | 2011 | 37.4 | 42.96 | 67.33 | 64.50 | 30000000000 | 680000000 | 35000000 | 11335.50 | 48.53 | 10.00 | 1.67 | 73.10 |
| 109 | 2012 | 38.1 | 43.33 | 66.13 | 66.75 | 32000000000 | 670000000 | 36000000 | 11707.30 | 48.55 | 11.00 | 1.80 | 26.61 |
| 109 | 2013 | 36.56 | 44.33 | 63.79 | 65.00 | 38000000000 | 770000000 | 38000000 | 12519.40 | 46.97 | 11.00 | 1.90 | 42.76 |
| 109 | 2014 | 39.44 | 47.83 | 65.17 | 65.88 | 40000000000 | 770000000 | 40000000 | 12095.90 | 44.92 | 13.00 | 2.19 | 39.32 |
| 109 | 2015 | 38.62 | 47.67 | 65.50 | 64.08 | 36000000000 | 770000000 | 39000000 | 10948.70 | 40.64 | 15.00 | 2.72 | 20.00 |
| 109 | 2016 | 37.15 | 46.79 | 62.79 | 64.71 | 27000000000 | 820000000 | 30000000 | 10820.60 | 40.64 | 16.00 | 3.02 | 69.20 |
| 109 | 2017 | 38.58 | 47.42 | 64.75 | 65.00 | 32000000000 | 870000000 | 38000000 | 10513.60 | 44.39 | 17.00 | 3.65 | 46.50 |
| 109 | 2018 | 39.98 | 48.21 | 66.33 | 65.42 | 37000000000 | 950000000 | 46000000 | 9370.18 | 49.04 | 18.00 | 4.83 | 53.74 |
| 109 | 2019 | 39.87 | 46.92 | 64.46 | 68.38 | 42000000000 | 890000000 | 480000 | 17398.00 | 11.39 | 18.00 | 6.76 | 28.81 |
| 110 | 2000 | 29.98 | 35.17 | 60.17 | 64.63 | 500000000 | 220000000 | 895000 | 1664.30 | 19.45 | 3.00 | 7.76 | 64.00 |
| 110 | 2001 | 29.17 | 32.38 | 60.54 | 65.42 | 590000000 | 270000000 | 974000 | 1575.43 | 26.58 | 3.00 | 7.86 | 71.20 |
| 110 | 2002 | 32.48 | 36.54 | 61.46 | 66.96 | 650000000 | 330000000 | 1100000 | 1710.34 | 24.46 | 3.00 | 7.82 | 17.83 |
| 110 | 2003 | 32.6 | 38.46 | 60.17 | 66.58 | 650000000 | 370000000 | 1100000 | 1765.35 | 22.24 | 3.00 | 7.94 | 48.88 |
| 110 | 2004 | 29.96 | 34.29 | 59.42 | 66.21 | 630000000 | 490000000 | 1200000 | 1889.69 | 22.75 | 3.00 | 7.95 | 64.57 |
| 110 | 2005 | 29.42 | 33.75 | 59.00 | 66.08 | 790000000 | 530000000 | 1300000 | 2101.54 | 21.02 | 3.00 | 7.63 | 78.69 |
| 110 | 2006 | 29.83 | 35.29 | 59.25 | 65.13 | 920000000 | 660000000 | 1400000 | 2287.94 | 21.65 | 3.00 | 7.60 | 66.83 |
| 110 | 2007 | 30.52 | 36.33 | 59.71 | 65.00 | 1100000000 | 740000000 | 1400000 | 2531.21 | 21.22 | 3.00 | 7.67 | 87.48 |
| 110 | 2008 | 33.21 | 38.00 | 61.21 | 67.21 | 1100000000 | 820000000 | 1500000 | 2848.50 | 22.83 | 3.00 | 7.56 | 79.01 |
| 110 | 2009 | 33.87 | 39.50 | 61.17 | 67.08 | 1100000000 | 750000000 | 1100000 | 2695.22 | 22.57 | 3.00 | 8.16 | 74.00 |
| 110 | 2010 | 33.02 | 39.50 | 59.63 | 66.92 | 1100000000 | 840000000 | 1100000 | 2898.94 | 20.45 | 3.00 | 8.06 | 88.22 |
| 110 | 2011 | 32.81 | 38.92 | 60.04 | 66.67 | 1100000000 | 880000000 | 1200000 | 3281.67 | 22.26 | 3.00 | 7.79 | 72.83 |
| 110 | 2012 | 32.21 | 38.25 | 60.67 | 65.50 | 1100000000 | 930000000 | 1200000 | 3408.78 | 23.67 | 3.00 | 7.83 | 31.87 |
| 110 | 2013 | 32.25 | 38.46 | 60.54 | 65.50 | 1200000000 | 1000000000 | 1200000 | 3522.77 | 22.27 | 3.00 | 7.86 | 42.22 |
| 110 | 2014 | 33.25 | 40.00 | 61.00 | 65.50 | 1200000000 | 1000000000 | 1400000 | 3779.64 | 23.76 | 3.00 | 7.73 | 59.10 |
| 110 | 2015 | 32.31 | 39.58 | 60.75 | 64.29 | 1200000000 | 1000000000 | 1500000 | 3994.64 | 23.35 | 3.00 | 7.65 | 24.09 |
| 110 | 2016 | 29.92 | 36.33 | 60.00 | 63.50 | 1200000000 | 1000000000 | 1600000 | 4173.30 | 21.97 | 3.00 | 7.60 | 58.00 |
| 110 | 2017 | 30.4 | 38.29 | 59.17 | 63.33 | 1200000000 | 1100000000 | 1700000 | 4451.45 | 24.77 | 3.00 | 7.35 | 45.33 |
| 110 | 2018 | 28.83 | 37.58 | 57.21 | 62.88 | 1200000000 | 1100000000 | 1800000 | 4472.89 | 29.53 | 3.00 | 7.52 | 77.35 |
| 110 | 2019 | 27.65 | 35.33 | 57.08 | 62.88 | 1200000000 | 1100000000 | 9400000 | 3317.45 | 49.30 | 3.00 | 2.87 | 58.14 |
| 111 | 2000 | 34.02 | 42.58 | 60.13 | 65.33 | 470000000 | 1600000000 | 469000 | 4842.04 | 29.74 | 3.00 | 0.68 | 17.00 |
| 111 | 2001 | 30.1 | 40.58 | 56.83 | 62.79 | 680000000 | 1700000000 | 584000 | 4986.68 | 22.74 | 3.00 | 0.72 | 22.06 |
| 111 | 2002 | 42.58 | 50.96 | 63.83 | 70.38 | 480000000 | 1500000000 | 432000 | 3700.88 | 30.43 | 3.00 | 1.16 | 92.00 |
| 111 | 2003 | 42.12 | 51.25 | 61.92 | 71.08 | 380000000 | 1300000000 | 337000 | 3272.61 | 33.85 | 3.00 | 1.61 | 60.11 |
| 111 | 2004 | 38.46 | 48.92 | 64.08 | 63.92 | 550000000 | 1600000000 | 486000 | 4325.70 | 36.20 | 3.00 | 1.89 | 45.00 |
| 111 | 2005 | 32.48 | 47.00 | 56.00 | 61.96 | 720000000 | 1800000000 | 706000 | 5504.98 | 39.66 | 3.00 | 2.09 | 21.00 |
| 111 | 2006 | 30.65 | 47.54 | 54.33 | 59.42 | 840000000 | 1800000000 | 748000 | 6833.38 | 36.52 | 3.00 | 2.15 | 76.63 |
| 111 | 2007 | 32.52 | 48.54 | 56.75 | 59.75 | 970000000 | 2300000000 | 756000 | 8454.47 | 31.13 | 3.00 | 2.15 | 50.30 |
| 111 | 2008 | 33.37 | 50.25 | 55.21 | 61.29 | 1100000000 | 2700000000 | 699000 | 11432.70 | 30.82 | 3.00 | 2.15 | 66.36 |
| 111 | 2009 | 39.71 | 51.67 | 56.50 | 71.25 | 1100000000 | 3000000000 | 562000 | 11765.10 | 18.07 | 3.00 | 2.15 | 42.95 |
| 111 | 2010 | 45.71 | 53.54 | 63.79 | 74.08 | 890000000 | 2900000000 | 526000 | 13825.40 | 28.53 | 3.00 | 2.58 | 70.97 |
| 111 | 2011 | 39.73 | 53.71 | 54.83 | 70.92 | 810000000 | 4000000000 | 595000 | 10955.30 | 29.94 | 3.00 | 4.29 | 90.60 |
| 111 | 2012 | 38.02 | 53.29 | 56.17 | 66.58 | 900000000 | 4600000000 | 988000 | 12985.50 | 26.17 | 3.00 | 4.29 | 76.85 |
| 111 | 2013 | 44.54 | 54.88 | 63.71 | 70.50 | 930000000 | 5400000000 | 986000 | 12456.70 | 24.77 | 3.00 | 6.05 | 78.20 |
| 111 | 2014 | 45.46 | 55.29 | 60.13 | 75.50 | 900000000 | 4100000000 | 857000 | 16054.50 | 16.69 | 3.00 | 6.28 | 41.25 |
| 111 | 2015 | 48.5 | 55.92 | 59.58 | 81.50 | 650000000 | 3200000000 | 789000 | 19652.30 | 15.19 | 3.00 | 6.28 | 15.37 |
| 111 | 2016 | 56.42 | 56.00 | 70.88 | 85.96 | 550000000 | 2900000000 | 601000 | 23250.00 | 14.69 | 3.00 | 9.26 | 83.80 |
| 111 | 2017 | 53.56 | 54.63 | 67.50 | 85.00 | 440000000 | 3100000000 | 427000 | 26847.80 | 13.39 | 3.00 | 9.98 | 87.24 |
| 111 | 2018 | 54.12 | 54.08 | 68.92 | 85.25 | 330000000 | 3200000000 | 514000 | 30445.60 | 12.39 | 3.00 | 10.69 | 13.00 |
| 111 | 2019 | 57.9 | 56.42 | 75.50 | 83.88 | 220000000 | 3400000000 | 2600000 | 4494.33 | 34.29 | 3.00 | 7.69 | 84.32 |
| 112 | 2000 | 14.33 | 23.17 | 51.50 | 54.00 | 130000000 | 430000000 | 91000 | 18012.60 | 67.35 | 1.00 | 1.72 | 86.54 |
| 112 | 2001 | 12.27 | 20.38 | 50.67 | 53.50 | 160000000 | 460000000 | 98000 | 16472.10 | 69.53 | 1.00 | 1.79 | 9.60 |
| 112 | 2002 | 11.75 | 19.50 | 50.50 | 53.50 | 110000000 | 400000000 | 105000 | 16850.20 | 67.12 | 1.00 | 1.79 | 80.00 |
| 112 | 2003 | 12.21 | 18.63 | 50.29 | 55.50 | 120000000 | 470000000 | 112000 | 18560.60 | 69.29 | 1.00 | 1.74 | 12.39 |
| 112 | 2004 | 12.02 | 18.33 | 50.00 | 55.71 | 180000000 | 380000000 | 119000 | 21902.10 | 68.80 | 1.00 | 1.69 | 53.40 |
| 112 | 2005 | 11.19 | 18.21 | 52.50 | 51.67 | 190000000 | 370000000 | 126000 | 26105.30 | 70.17 | 1.00 | 1.66 | 98.00 |
| 112 | 2006 | 10.9 | 16.79 | 52.50 | 52.50 | 220000000 | 410000000 | 158000 | 30979.90 | 71.72 | 1.00 | 1.59 | 54.00 |
| 112 | 2007 | 11.44 | 16.50 | 53.42 | 52.96 | 230000000 | 430000000 | 179000 | 32663.60 | 67.85 | 1.00 | 1.51 | 60.87 |
| 112 | 2008 | 11.65 | 16.58 | 53.75 | 52.96 | 240000000 | 460000000 | 226000 | 37934.40 | 78.30 | 1.00 | 1.42 | 71.11 |
| 112 | 2009 | 14.52 | 17.50 | 55.00 | 56.54 | 250000000 | 480000000 | 157000 | 27955.70 | 72.78 | 1.00 | 1.45 | 59.83 |
| 112 | 2010 | 12.5 | 17.50 | 53.50 | 54.00 | 150000000 | 520000000 | 214000 | 35269.60 | 67.41 | 1.00 | 1.36 | 86.52 |
| 112 | 2011 | 12.58 | 17.50 | 53.67 | 54.00 | 120000000 | 550000000 | 242000 | 47055.80 | 69.51 | 1.00 | 1.26 | 98.24 |
| 112 | 2012 | 11.83 | 18.50 | 52.17 | 53.00 | 92000000 | 590000000 | 209000 | 47740.50 | 70.16 | 1.00 | 1.25 | 73.30 |
| 112 | 2013 | 12.54 | 19.88 | 51.79 | 53.42 | 96000000 | 620000000 | 225000 | 44740.10 | 68.04 | 1.00 | 1.25 | 60.26 |
| 112 | 2014 | 13.29 | 22.54 | 51.50 | 52.54 | 79000000 | 620000000 | 201000 | 41726.80 | 68.18 | 1.00 | 1.27 | 39.36 |
| 112 | 2015 | 16.81 | 26.58 | 52.96 | 54.08 | 150000000 | 460000000 | 218000 | 31164.60 | 52.21 | 1.00 | 1.37 | 13.96 |
| 112 | 2016 | 26.87 | 26.71 | 55.54 | 71.50 | 140000000 | 500000000 | 219000 | 27157.80 | 49.58 | 1.00 | 1.38 | 96.97 |
| 112 | 2017 | 24.54 | 26.50 | 54.75 | 67.83 | 180000000 | 500000000 | 259000 | 28572.10 | 49.57 | 1.00 | 1.38 | 84.17 |
| 112 | 2018 | 21.23 | 24.92 | 53.04 | 64.50 | 190000000 | 590000000 | 278000 | 31628.30 | 51.93 | 1.00 | 1.35 | 11.31 |
| 112 | 2019 | 17.98 | 26.33 | 51.13 | 58.50 | 200000000 | 670000000 | 297000 | 31086.80 | 57.95 | 1.00 | 11.41 | 63.87 |
| 113 | 2000 | 36.5 | 42.83 | 64.83 | 65.33 | 180000000 | 150000000 | 193000 | 261.87 | 10.65 | 2.00 | 1644.48 | 73.09 |
| 113 | 2001 | 34.75 | 40.04 | 62.96 | 66.50 | 190000000 | 160000000 | 205000 | 239.47 | 11.52 | 3.00 | 1755.66 | 54.06 |
| 113 | 2002 | 37.48 | 43.83 | 64.63 | 66.50 | 190000000 | 160000000 | 254000 | 245.50 | 11.21 | 3.00 | 1797.55 | 79.27 |
| 113 | 2003 | 37.77 | 43.46 | 65.58 | 66.50 | 190000000 | 170000000 | 305000 | 243.90 | 11.39 | 3.00 | 1963.72 | 55.50 |
| 113 | 2004 | 38.21 | 44.58 | 65.33 | 66.50 | 270000000 | 160000000 | 512000 | 296.05 | 12.70 | 3.00 | 1810.30 | 87.70 |
| 113 | 2005 | 37.48 | 45.00 | 64.92 | 65.04 | 380000000 | 190000000 | 468000 | 325.59 | 14.18 | 3.00 | 1780.54 | 18.50 |
| 113 | 2006 | 37.6 | 44.75 | 64.63 | 65.83 | 350000000 | 200000000 | 539000 | 347.99 | 15.28 | 3.00 | 1831.45 | 8.12 |
| 113 | 2007 | 37.75 | 44.92 | 64.58 | 66.00 | 400000000 | 220000000 | 642000 | 416.90 | 16.73 | 3.00 | 1723.49 | 6.21 |
| 113 | 2008 | 36.87 | 46.21 | 61.21 | 66.33 | 540000000 | 320000000 | 844000 | 467.90 | 24.28 | 3.00 | 1720.44 | 58.14 |
| 113 | 2009 | 37.23 | 46.00 | 61.96 | 66.50 | 680000000 | 350000000 | 807000 | 793.48 | 18.77 | 3.00 | 2030.49 | 65.88 |
| 113 | 2010 | 36.77 | 46.63 | 60.29 | 66.63 | 800000000 | 460000000 | 946000 | 815.87 | 13.94 | 3.00 | 2177.56 | 42.98 |
| 113 | 2011 | 37.9 | 49.29 | 59.75 | 66.75 | 980000000 | 540000000 | 1200000 | 825.84 | 12.96 | 3.00 | 2522.80 | 35.66 |
| 113 | 2012 | 39.67 | 50.46 | 59.38 | 69.50 | 1200000000 | 640000000 | 1200000 | 783.72 | 15.62 | 3.00 | 2504.56 | 74.59 |
| 113 | 2013 | 43.79 | 51.67 | 61.42 | 74.50 | 960000000 | 350000000 | 1200000 | 803.51 | 16.65 | 3.00 | 2586.89 | 12.23 |
| 113 | 2014 | 41.67 | 51.71 | 61.71 | 69.92 | 980000000 | 340000000 | 1300000 | 876.35 | 15.07 | 3.00 | 2599.79 | 92.84 |
| 113 | 2015 | 41.08 | 48.17 | 63.75 | 70.25 | 1000000000 | 280000000 | 1300000 | 840.40 | 12.98 | 3.00 | 3240.65 | 90.41 |
| 113 | 2016 | 39.96 | 48.96 | 60.46 | 70.50 | 1100000000 | 310000000 | 1300000 | 730.63 | 12.53 | 3.00 | 3420.10 | 69.88 |
| 113 | 2017 | 39.29 | 49.08 | 59.92 | 69.58 | 960000000 | 350000000 | 1400000 | 747.20 | 16.66 | 3.00 | 3611.22 | 29.50 |
| 113 | 2018 | 37.37 | 48.00 | 59.58 | 67.17 | 1100000000 | 340000000 | 1500000 | 767.10 | 15.16 | 3.00 | 3727.07 | 9.80 |
| 113 | 2019 | 37.65 | 47.63 | 60.33 | 67.33 | 1200000000 | 360000000 | 333000 | 794.34 | 17.20 | 3.00 | 1.32 | 3.76 |
| 114 | 2000 | 39.9 | 40.67 | 67.63 | 71.50 | 560000000 | 560000000 | 6400000 | 635.70 | 62.44 | 2.00 | 5.44 | 91.16 |
| 114 | 2001 | 34.62 | 42.63 | 60.71 | 65.92 | 760000000 | 680000000 | 9200000 | 780.32 | 52.52 | 2.00 | 5.37 | 34.67 |
| 114 | 2002 | 33 | 42.25 | 60.50 | 63.25 | 1000000000 | 790000000 | 11000000 | 878.62 | 52.16 | 2.00 | 5.33 | 48.05 |
| 114 | 2003 | 31.65 | 40.42 | 60.17 | 62.71 | 1200000000 | 950000000 | 13000000 | 1047.50 | 54.69 | 2.00 | 5.33 | 76.48 |
| 114 | 2004 | 29.08 | 38.21 | 58.63 | 61.33 | 2900000000 | 2700000000 | 16000000 | 1366.02 | 60.26 | 2.00 | 5.32 | 23.12 |
| 114 | 2005 | 27.23 | 32.33 | 60.42 | 61.71 | 3500000000 | 3100000000 | 18000000 | 1826.93 | 48.75 | 3.00 | 5.12 | 23.20 |
| 114 | 2006 | 28.5 | 32.42 | 59.88 | 64.71 | 4000000000 | 3200000000 | 19000000 | 2300.77 | 44.15 | 3.00 | 5.05 | 95.12 |
| 114 | 2007 | 29.71 | 32.33 | 61.71 | 65.38 | 5300000000 | 4000000000 | 23000000 | 3065.61 | 42.46 | 4.00 | 5.05 | 41.21 |
| 114 | 2008 | 31.6 | 31.79 | 63.42 | 68.00 | 6700000000 | 4600000000 | 25000000 | 3887.24 | 44.43 | 4.00 | 5.27 | 78.37 |
| 114 | 2009 | 40.96 | 37.75 | 69.04 | 75.13 | 4300000000 | 3800000000 | 21000000 | 2543.00 | 43.91 | 4.00 | 7.79 | 72.70 |
| 114 | 2010 | 35.83 | 36.17 | 64.50 | 71.00 | 4700000000 | 4100000000 | 21000000 | 2965.14 | 47.05 | 4.00 | 7.94 | 16.60 |
| 114 | 2011 | 35.21 | 36.25 | 66.33 | 67.83 | 5400000000 | 4800000000 | 21000000 | 3569.76 | 49.82 | 6.00 | 7.97 | 79.84 |
| 114 | 2012 | 34.33 | 35.29 | 66.38 | 67.00 | 6000000000 | 5500000000 | 23000000 | 3855.42 | 47.72 | 6.00 | 7.99 | 76.11 |
| 114 | 2013 | 34.08 | 36.50 | 64.17 | 67.50 | 5900000000 | 6300000000 | 25000000 | 4029.71 | 42.96 | 8.00 | 7.99 | 74.38 |
| 114 | 2014 | 42.65 | 42.83 | 70.88 | 71.58 | 2300000000 | 5500000000 | 13000000 | 3104.64 | 48.59 | 8.00 | 11.89 | 20.27 |
| 114 | 2015 | 46.62 | 41.58 | 73.50 | 78.17 | 1700000000 | 5400000000 | 12000000 | 2124.66 | 52.60 | 8.00 | 21.84 | 98.14 |
| 114 | 2016 | 40.81 | 39.54 | 69.63 | 72.46 | 1700000000 | 6300000000 | 13000000 | 2187.73 | 49.30 | 8.00 | 25.55 | 59.50 |
| 114 | 2017 | 37.85 | 38.96 | 67.67 | 69.08 | 2000000000 | 7500000000 | 14000000 | 2640.68 | 48.01 | 9.00 | 26.60 | 4.71 |
| 114 | 2018 | 36.81 | 39.88 | 65.50 | 68.25 | 2300000000 | 8300000000 | 14000000 | 3096.82 | 45.20 | 9.00 | 27.20 | 78.08 |
| 114 | 2019 | 34.19 | 36.79 | 64.46 | 67.13 | 2600000000 | 8900000000 | 14000000 | 3659.03 | 41.17 | 9.00 | 3842.91 | 11.47 |
| 115 | 2000 | 26.85 | 26.00 | 63.96 | 63.75 | 830000000 | 380000000 | 2000000 | 6875.02 | 16.70 | 1.00 | 12.10 | 78.79 |
| 115 | 2001 | 25.19 | 21.00 | 64.46 | 64.92 | 700000000 | 340000000 | 1900000 | 6284.46 | 16.80 | 1.00 | 13.32 | 11.11 |
| 115 | 2002 | 32.19 | 25.00 | 69.38 | 70.00 | 410000000 | 240000000 | 1300000 | 4090.90 | 20.62 | 1.00 | 21.26 | 85.54 |
| 115 | 2003 | 36.58 | 29.13 | 72.46 | 71.58 | 420000000 | 240000000 | 1400000 | 3624.20 | 27.43 | 1.00 | 28.21 | 22.27 |
| 115 | 2004 | 31.56 | 28.25 | 69.88 | 65.00 | 590000000 | 270000000 | 1800000 | 4120.56 | 32.11 | 1.00 | 28.70 | 18.02 |
| 115 | 2005 | 29.23 | 25.67 | 69.58 | 63.21 | 700000000 | 330000000 | 1800000 | 5226.94 | 30.40 | 1.00 | 24.48 | 45.46 |
| 115 | 2006 | 31.06 | 28.46 | 69.58 | 64.08 | 710000000 | 310000000 | 1700000 | 5887.85 | 30.30 | 1.00 | 24.07 | 25.07 |
| 115 | 2007 | 30.27 | 29.33 | 68.21 | 63.00 | 930000000 | 350000000 | 1800000 | 7026.51 | 29.09 | 1.00 | 23.47 | 71.00 |
| 115 | 2008 | 29.42 | 29.58 | 67.42 | 61.83 | 1200000000 | 470000000 | 1900000 | 9091.08 | 30.20 | 1.00 | 20.95 | 58.27 |
| 115 | 2009 | 29.65 | 30.46 | 65.50 | 63.33 | 1500000000 | 440000000 | 2100000 | 9451.93 | 27.10 | 1.00 | 22.57 | 17.52 |
| 115 | 2010 | 25.48 | 27.38 | 61.58 | 62.00 | 1700000000 | 550000000 | 2400000 | 11992.00 | 26.34 | 1.00 | 20.06 | 59.54 |
| 115 | 2011 | 23.42 | 26.33 | 59.58 | 60.92 | 2400000000 | 800000000 | 2900000 | 14236.70 | 26.42 | 1.00 | 19.31 | 31.03 |
| 115 | 2012 | 24.6 | 27.67 | 59.88 | 61.67 | 2400000000 | 1000000000 | 2700000 | 15171.60 | 25.92 | 1.00 | 20.31 | 54.00 |
| 115 | 2013 | 25.56 | 29.13 | 59.46 | 62.54 | 2200000000 | 1500000000 | 2700000 | 16973.70 | 23.35 | 1.00 | 20.48 | 24.57 |
| 115 | 2014 | 27.37 | 27.92 | 62.42 | 64.42 | 2000000000 | 1600000000 | 2700000 | 16832.00 | 23.54 | 1.00 | 23.25 | 4.32 |
| 115 | 2015 | 28.27 | 27.75 | 63.54 | 65.25 | 2100000000 | 1400000000 | 2800000 | 15613.80 | 22.48 | 2.00 | 27.33 | 25.67 |
| 115 | 2016 | 29.54 | 28.54 | 64.08 | 66.46 | 2200000000 | 1100000000 | 3000000 | 15387.10 | 21.43 | 2.00 | 30.16 | 97.30 |
| 115 | 2017 | 27.71 | 28.38 | 61.92 | 65.13 | 2700000000 | 1200000000 | 3700000 | 17322.10 | 21.43 | 2.00 | 28.68 | 70.42 |
| 115 | 2018 | 25.42 | 27.67 | 61.25 | 61.92 | 2400000000 | 1300000000 | 3500000 | 17278.00 | 21.00 | 2.00 | 30.73 | 93.18 |
| 115 | 2019 | 27.29 | 27.92 | 62.38 | 64.29 | 2100000000 | 1500000000 | 14000000 | 16190.10 | 21.72 | 2.00 | 27.80 | 89.65 |
| 116 | 2000 | 22.29 | 21.75 | 63.21 | 59.63 | 37000000000 | 9000000000 | 46000000 | 14713.10 | 28.56 | 41.00 | 0.66 | 89.13 |
| 116 | 2001 | 19.65 | 17.88 | 61.42 | 60.00 | 40000000000 | 10000000000 | 49000000 | 15355.70 | 27.86 | 43.00 | 0.69 | 29.00 |
| 116 | 2002 | 19.4 | 17.50 | 60.79 | 60.50 | 43000000000 | 11000000000 | 50000000 | 17025.50 | 26.55 | 43.00 | 0.67 | 67.06 |
| 116 | 2003 | 19.6 | 17.33 | 61.25 | 60.63 | 46000000000 | 12000000000 | 51000000 | 21463.40 | 25.63 | 44.00 | 0.61 | 11.77 |
| 116 | 2004 | 20.79 | 19.96 | 61.00 | 60.63 | 49000000000 | 13000000000 | 52000000 | 24861.30 | 25.41 | 44.00 | 0.55 | 25.66 |
| 116 | 2005 | 21.69 | 20.38 | 62.46 | 60.54 | 52000000000 | 14000000000 | 56000000 | 26419.30 | 24.98 | 46.00 | 0.55 | 75.90 |
| 116 | 2006 | 21.65 | 19.08 | 63.67 | 60.54 | 55000000000 | 15000000000 | 58000000 | 28365.30 | 25.24 | 47.00 | 0.54 | 74.88 |
| 116 | 2007 | 22.42 | 20.46 | 63.88 | 60.50 | 58000000000 | 16000000000 | 59000000 | 32550.00 | 25.98 | 49.00 | 0.50 | 16.40 |
| 116 | 2008 | 24.31 | 21.13 | 65.00 | 62.50 | 61000000000 | 17000000000 | 57000000 | 35366.30 | 25.62 | 50.00 | 0.54 | 80.48 |
| 116 | 2009 | 28.35 | 24.42 | 66.38 | 65.92 | 64000000000 | 18000000000 | 52000000 | 32042.50 | 23.06 | 51.00 | 0.64 | 75.50 |
| 116 | 2010 | 30.73 | 26.88 | 65.83 | 68.75 | 67000000000 | 19000000000 | 53000000 | 30502.70 | 25.95 | 52.00 | 0.65 | 14.10 |
| 116 | 2011 | 31.23 | 30.50 | 65.96 | 66.00 | 70000000000 | 20000000000 | 56000000 | 31636.40 | 29.54 | 54.00 | 0.62 | 45.40 |
| 116 | 2012 | 33.54 | 31.79 | 67.50 | 67.79 | 73000000000 | 21000000000 | 57000000 | 28324.40 | 31.46 | 55.00 | 0.63 | 47.50 |
| 116 | 2013 | 31.92 | 32.25 | 65.08 | 66.50 | 76000000000 | 21000000000 | 61000000 | 29059.50 | 32.96 | 55.00 | 0.64 | 13.50 |
| 116 | 2014 | 29 | 30.25 | 64.79 | 62.96 | 79000000000 | 22000000000 | 65000000 | 29461.60 | 33.48 | 55.00 | 0.61 | 73.30 |
| 116 | 2015 | 27.62 | 27.83 | 66.63 | 60.79 | 82000000000 | 23000000000 | 68000000 | 25732.00 | 33.63 | 56.00 | 0.65 | 49.60 |
| 116 | 2016 | 25.75 | 28.17 | 63.54 | 59.79 | 85000000000 | 24000000000 | 75000000 | 26505.30 | 33.88 | 57.00 | 0.74 | 58.35 |
| 116 | 2017 | 24.77 | 26.46 | 63.08 | 60.00 | 88000000000 | 25000000000 | 82000000 | 28170.40 | 35.18 | 58.00 | 0.78 | 34.51 |
| 116 | 2018 | 24.73 | 26.00 | 63.67 | 59.79 | 90000000000 | 26000000000 | 83000000 | 30337.70 | 35.12 | 59.00 | 0.75 | 60.00 |
| 116 | 2019 | 24.1 | 24.71 | 63.79 | 59.71 | 93000000000 | 27000000000 | 3500000 | 29600.40 | 34.89 | 59.00 | 32.77 | 90.00 |
| 117 | 2000 | 24.69 | 20.67 | 67.83 | 60.88 | 9300000000 | 4600000000 | 13000000 | 12043.00 | 23.72 | 16.00 | 365.40 | 21.88 |
| 117 | 2001 | 24.27 | 21.58 | 66.83 | 60.13 | 9200000000 | 4200000000 | 14000000 | 12538.20 | 22.79 | 16.00 | 0.69 | 53.00 |
| 117 | 2002 | 25.48 | 23.38 | 66.54 | 61.04 | 10000000000 | 2500000000 | 14000000 | 14110.30 | 20.11 | 16.00 | 0.67 | 84.95 |
| 117 | 2003 | 24.4 | 22.04 | 64.46 | 62.29 | 11000000000 | 2400000000 | 14000000 | 18477.60 | 18.54 | 16.00 | 0.61 | 80.56 |
| 117 | 2004 | 25.12 | 23.04 | 64.50 | 62.71 | 13000000000 | 2900000000 | 13000000 | 21955.10 | 20.71 | 16.00 | 0.55 | 69.09 |
| 117 | 2005 | 26.06 | 22.75 | 65.67 | 63.71 | 13000000000 | 3000000000 | 15000000 | 22551.70 | 21.31 | 16.00 | 0.55 | 89.42 |
| 117 | 2006 | 25.83 | 23.04 | 65.21 | 63.42 | 15000000000 | 3000000000 | 16000000 | 24801.20 | 21.17 | 16.00 | 0.54 | 84.45 |
| 117 | 2007 | 26.52 | 23.21 | 66.29 | 63.54 | 16000000000 | 3400000000 | 16000000 | 28827.30 | 22.52 | 17.00 | 0.50 | 78.00 |
| 117 | 2008 | 28.52 | 25.42 | 67.33 | 64.29 | 18000000000 | 3900000000 | 16000000 | 31997.30 | 23.36 | 17.00 | 0.54 | 88.47 |
| 117 | 2009 | 31.23 | 27.00 | 66.58 | 68.88 | 16000000000 | 4100000000 | 15000000 | 29711.00 | 18.98 | 17.00 | 0.64 | 79.26 |
| 117 | 2010 | 36.54 | 30.33 | 69.92 | 72.83 | 14000000000 | 3400000000 | 15000000 | 26917.80 | 22.10 | 17.00 | 0.65 | 34.25 |
| 117 | 2011 | 38.12 | 32.54 | 70.21 | 73.50 | 16000000000 | 3800000000 | 16000000 | 25916.30 | 25.54 | 17.00 | 0.62 | 44.37 |
| 117 | 2012 | 38.08 | 33.21 | 69.88 | 73.08 | 15000000000 | 3000000000 | 16000000 | 22242.70 | 28.68 | 17.00 | 0.63 | 64.35 |
| 117 | 2013 | 35.87 | 34.79 | 67.75 | 69.21 | 17000000000 | 3800000000 | 18000000 | 21874.80 | 30.35 | 17.00 | 0.64 | 24.58 |
| 117 | 2014 | 35.25 | 34.04 | 70.42 | 66.04 | 19000000000 | 4000000000 | 22000000 | 21761.00 | 32.37 | 17.00 | 0.61 | 21.23 |
| 117 | 2015 | 32.52 | 29.79 | 72.17 | 63.08 | 18000000000 | 3500000000 | 24000000 | 18167.80 | 31.55 | 17.00 | 0.65 | 53.23 |
| 117 | 2016 | 33.06 | 29.42 | 71.21 | 65.50 | 17000000000 | 3400000000 | 25000000 | 18116.50 | 30.06 | 18.00 | 0.74 | 79.65 |
| 117 | 2017 | 31.94 | 28.79 | 71.50 | 63.58 | 19000000000 | 3300000000 | 27000000 | 18930.20 | 32.99 | 18.00 | 0.78 | 61.32 |
| 117 | 2018 | 28.92 | 26.25 | 70.50 | 61.08 | 22000000000 | 3900000000 | 30000000 | 20324.30 | 36.13 | 18.00 | 0.75 | 22.00 |
| 117 | 2019 | 28.12 | 24.00 | 71.17 | 61.08 | 23000000000 | 4200000000 | 130000000 | 19582.50 | 37.19 | 18.00 | 0.72 | 25.45 |
| 118 | 2000 | 20.75 | 29.50 | 56.00 | 56.00 | 8200000000 | 13000000000 | 8800000 | 25756.70 | 126.03 | 0.00 | 7.79 | 94.78 |
| 118 | 2001 | 16.6 | 23.04 | 55.46 | 54.71 | 7900000000 | 12000000000 | 8900000 | 25230.20 | 122.77 | 0.00 | 7.80 | 62.30 |
| 118 | 2002 | 15.54 | 20.46 | 55.50 | 55.13 | 9800000000 | 12000000000 | 11000000 | 24665.90 | 132.08 | 0.00 | 7.80 | 53.00 |
| 118 | 2003 | 17.52 | 23.38 | 55.75 | 55.92 | 9000000000 | 11000000000 | 9700000 | 23977.00 | 150.78 | 1.00 | 7.79 | 25.51 |
| 118 | 2004 | 16.44 | 21.75 | 56.13 | 55.00 | 12000000000 | 13000000000 | 14000000 | 24928.10 | 167.78 | 1.00 | 7.79 | 83.56 |
| 118 | 2005 | 15.87 | 20.04 | 57.25 | 54.46 | 14000000000 | 13000000000 | 15000000 | 26649.80 | 177.45 | 1.00 | 7.78 | 53.20 |
| 118 | 2006 | 14.81 | 18.46 | 58.00 | 53.17 | 16000000000 | 14000000000 | 16000000 | 28224.20 | 185.19 | 1.00 | 7.77 | 71.85 |
| 118 | 2007 | 14.46 | 17.79 | 58.71 | 52.42 | 18000000000 | 15000000000 | 17000000 | 30594.00 | 186.41 | 1.00 | 7.80 | 47.69 |
| 118 | 2008 | 15.12 | 16.92 | 59.21 | 54.13 | 20000000000 | 16000000000 | 17000000 | 31515.70 | 193.42 | 1.00 | 7.79 | 74.29 |
| 118 | 2009 | 19.65 | 18.38 | 58.96 | 61.96 | 20000000000 | 16000000000 | 17000000 | 30697.30 | 178.14 | 1.00 | 7.75 | 94.82 |
| 118 | 2010 | 17.92 | 18.33 | 58.42 | 59.08 | 27000000000 | 17000000000 | 20000000 | 32550.00 | 205.32 | 1.00 | 7.77 | 80.19 |
| 118 | 2011 | 15.1 | 19.63 | 58.00 | 52.58 | 33000000000 | 19000000000 | 22000000 | 35142.50 | 212.85 | 2.00 | 7.78 | 79.00 |
| 118 | 2012 | 18.12 | 21.08 | 58.33 | 56.83 | 37000000000 | 20000000000 | 24000000 | 36730.90 | 215.85 | 2.00 | 7.76 | 44.95 |
| 118 | 2013 | 19.58 | 23.92 | 58.50 | 56.75 | 42000000000 | 21000000000 | 26000000 | 38403.80 | 221.61 | 2.00 | 7.76 | 18.62 |
| 118 | 2014 | 19.44 | 23.46 | 58.63 | 56.79 | 46000000000 | 22000000000 | 28000000 | 40315.30 | 213.09 | 2.00 | 7.75 | 84.11 |
| 118 | 2015 | 19.12 | 20.83 | 58.88 | 58.54 | 42000000000 | 23000000000 | 27000000 | 42431.90 | 195.90 | 2.00 | 7.75 | 88.10 |
| 118 | 2016 | 19.25 | 22.71 | 58.50 | 57.29 | 38000000000 | 24000000000 | 27000000 | 43731.10 | 187.02 | 2.00 | 7.76 | 14.34 |
| 118 | 2017 | 16.9 | 20.50 | 58.08 | 55.21 | 38000000000 | 25000000000 | 28000000 | 46165.90 | 188.92 | 2.00 | 7.79 | 87.94 |
| 118 | 2018 | 17.1 | 19.96 | 58.25 | 56.00 | 42000000000 | 26000000000 | 29000000 | 48542.90 | 188.36 | 2.00 | 7.84 | 86.55 |
| 118 | 2019 | 19.15 | 22.79 | 58.50 | 57.00 | 33000000000 | 28000000000 | 34000000 | 48713.50 | 177.46 | 2.00 | 0.72 | 11.21 |
| 119 | 2000 | 10.1 | 14.17 | 54.58 | 51.46 | 5100000000 | 4500000000 | 6100000 | 23852.30 | 188.35 | 0.00 | 1.72 | 85.00 |
| 119 | 2001 | 9.25 | 11.38 | 54.63 | 52.50 | 4600000000 | 6600000000 | 5900000 | 21700.00 | 182.89 | 0.00 | 1.79 | 17.11 |
| 119 | 2002 | 9.42 | 10.88 | 54.58 | 53.38 | 4500000000 | 8200000000 | 5900000 | 22159.70 | 184.09 | 0.00 | 1.79 | 61.08 |
| 119 | 2003 | 11.48 | 13.29 | 54.50 | 55.17 | 3800000000 | 8400000000 | 4700000 | 23730.20 | 202.59 | 0.00 | 1.74 | 95.88 |
| 119 | 2004 | 11.27 | 16.21 | 54.08 | 52.25 | 5300000000 | 9300000000 | 6600000 | 27608.50 | 213.95 | 0.00 | 1.69 | 59.95 |
| 119 | 2005 | 11.96 | 16.25 | 55.25 | 52.42 | 6200000000 | 10000000000 | 7100000 | 29961.30 | 225.16 | 0.00 | 1.66 | 67.47 |
| 119 | 2006 | 11.62 | 15.83 | 55.54 | 51.88 | 7500000000 | 11000000000 | 7600000 | 33769.20 | 228.04 | 0.00 | 1.59 | 74.44 |
| 119 | 2007 | 12 | 15.50 | 55.50 | 53.00 | 9100000000 | 14000000000 | 8000000 | 39432.90 | 212.78 | 0.00 | 1.51 | 63.41 |
| 119 | 2008 | 12.92 | 15.63 | 55.96 | 54.25 | 11000000000 | 16000000000 | 7800000 | 40007.50 | 228.99 | 0.00 | 1.41 | 87.68 |
| 119 | 2009 | 16.48 | 16.00 | 55.63 | 61.33 | 9200000000 | 16000000000 | 7500000 | 38927.20 | 190.85 | 0.00 | 1.45 | 98.26 |
| 119 | 2010 | 15.58 | 15.50 | 55.33 | 60.33 | 14000000000 | 19000000000 | 9200000 | 47237.00 | 198.00 | 0.00 | 1.36 | 75.99 |
| 119 | 2011 | 12.42 | 15.92 | 54.75 | 54.17 | 18000000000 | 21000000000 | 10000000 | 53890.40 | 203.33 | 0.00 | 1.26 | 64.89 |
| 119 | 2012 | 13.15 | 16.92 | 54.54 | 54.83 | 19000000000 | 23000000000 | 11000000 | 55546.50 | 196.72 | 0.00 | 1.25 | 41.41 |
| 119 | 2013 | 13.54 | 17.00 | 54.50 | 55.58 | 19000000000 | 24000000000 | 12000000 | 56967.40 | 195.08 | 0.00 | 1.25 | 15.88 |
| 119 | 2014 | 12.92 | 17.67 | 54.50 | 53.67 | 19000000000 | 26000000000 | 12000000 | 57562.50 | 191.95 | 0.00 | 1.27 | 97.10 |
| 119 | 2015 | 13.73 | 18.29 | 55.33 | 53.83 | 17000000000 | 24000000000 | 12000000 | 55646.60 | 178.39 | 1.00 | 1.37 | 84.39 |
| 119 | 2016 | 13.85 | 18.79 | 54.13 | 54.79 | 19000000000 | 24000000000 | 13000000 | 56828.30 | 165.09 | 1.00 | 1.38 | 12.36 |
| 119 | 2017 | 14.33 | 19.17 | 54.25 | 55.25 | 20000000000 | 25000000000 | 14000000 | 60913.70 | 170.71 | 1.00 | 1.38 | 67.57 |
| 119 | 2018 | 13.77 | 18.75 | 54.17 | 54.63 | 20000000000 | 25000000000 | 15000000 | 66188.80 | 177.68 | 1.00 | 1.35 | 76.01 |
| 119 | 2019 | 13.96 | 18.17 | 54.38 | 55.38 | 21000000000 | 26000000000 | 56000000 | 65233.30 | 173.52 | 1.00 | 7.88 | 59.19 |
| 120 | 2000 | 15.455 | 12.92 | 63.25 | 54.75 | 270000000 | 550000000 | 110000 | 12579.60 | 14.17 | 2.00 | 129.20 | 80.50 |
| 120 | 2001 | 14.895 | 10.71 | 62.81 | 56.27 | 270000000 | 520000000 | 101000 | 13595.70 | 15.27 | 2.00 | 133.24 | 60.05 |
| 120 | 2002 | 14.95 | 10.27 | 62.40 | 57.23 | 270000000 | 500000000 | 104000 | 14611.80 | 16.36 | 2.00 | 126.20 | 87.47 |
| 120 | 2003 | 15.28 | 11.19 | 62.31 | 57.06 | 270000000 | 470000000 | 102000 | 15628.00 | 17.45 | 2.00 | 105.50 | 19.84 |
| 120 | 2004 | 15.02 | 12.85 | 61.50 | 55.69 | 270000000 | 450000000 | 100000 | 16644.10 | 18.54 | 2.00 | 95.93 | 8.65 |
| 120 | 2005 | 15.415 | 13.35 | 61.73 | 55.75 | 270000000 | 430000000 | 101000 | 17660.20 | 19.63 | 2.00 | 95.92 | 8.62 |
| 120 | 2006 | 16.435 | 14.79 | 61.98 | 56.10 | 280000000 | 400000000 | 100000 | 18676.30 | 20.73 | 2.00 | 95.04 | 62.26 |
| 120 | 2007 | 16.28 | 14.58 | 61.23 | 56.75 | 300000000 | 380000000 | 103000 | 19692.40 | 21.82 | 2.00 | 87.07 | 71.58 |
| 120 | 2008 | 17.5 | 15.29 | 62.08 | 57.63 | 270000000 | 350000000 | 104000 | 20708.50 | 22.91 | 2.00 | 81.14 | 57.15 |
| 120 | 2009 | 20.19 | 14.10 | 62.35 | 63.92 | 240000000 | 330000000 | 99000 | 21724.70 | 24.00 | 2.00 | 85.56 | 37.33 |
| 120 | 2010 | 18.925 | 13.77 | 60.73 | 63.35 | 230000000 | 340000000 | 99000 | 22740.80 | 25.10 | 2.00 | 90.01 | 76.43 |
| 120 | 2011 | 16.365 | 14.19 | 59.02 | 59.52 | 290000000 | 360000000 | 112000 | 23756.90 | 26.19 | 2.00 | 85.73 | 12.33 |
| 120 | 2012 | 16.345 | 14.58 | 58.69 | 59.42 | 290000000 | 340000000 | 112000 | 24773.00 | 27.28 | 3.00 | 92.88 | 95.07 |
| 120 | 2013 | 15.98 | 14.79 | 58.75 | 58.42 | 290000000 | 330000000 | 108000 | 25789.10 | 28.37 | 3.00 | 89.85 | 93.20 |
| 120 | 2014 | 15.02 | 15.19 | 58.27 | 56.58 | 290000000 | 330000000 | 107000 | 26805.30 | 29.46 | 3.00 | 89.82 | 71.27 |
| 120 | 2015 | 16.145 | 15.04 | 60.33 | 56.92 | 250000000 | 250000000 | 114000 | 27821.40 | 30.56 | 3.00 | 107.55 | 32.14 |
| 120 | 2016 | 15.475 | 15.31 | 58.38 | 57.27 | 250000000 | 250000000 | 116000 | 28837.50 | 31.65 | 3.00 | 107.81 | 11.40 |
| 120 | 2017 | 15.385 | 16.27 | 57.67 | 56.83 | 250000000 | 290000000 | 121000 | 29853.60 | 32.74 | 3.00 | 105.63 | 3.93 |
| 120 | 2018 | 15.585 | 16.58 | 57.90 | 56.69 | 250000000 | 330000000 | 120000 | 30869.70 | 33.83 | 3.00 | 101.05 | 92.70 |
| 120 | 2019 | 15.53 | 16.10 | 57.77 | 57.19 | 250000000 | 370000000 | 19000000 | 71463.80 | 184.66 | 3.00 | 1.32 | 37.88 |
| 121 | 2000 | 20.81 | 11.67 | 71.92 | 58.04 | 2300000000 | 1200000000 | 1800000 | 13641.10 | 35.75 | 4.00 | 2.20 | 50.32 |
| 121 | 2001 | 20.54 | 10.04 | 71.00 | 60.04 | 2300000000 | 1300000000 | 1900000 | 13882.90 | 35.43 | 4.00 | 2.38 | 78.72 |
| 121 | 2002 | 20.48 | 9.67 | 70.21 | 61.08 | 3200000000 | 1400000000 | 2000000 | 16874.20 | 32.85 | 4.00 | 2.16 | 27.06 |
| 121 | 2003 | 19.08 | 9.08 | 70.13 | 58.96 | 4200000000 | 1600000000 | 2100000 | 21913.70 | 29.84 | 4.00 | 1.72 | 23.20 |
| 121 | 2004 | 18.77 | 9.50 | 68.92 | 59.13 | 5100000000 | 2200000000 | 2300000 | 25420.20 | 29.54 | 4.00 | 1.51 | 97.39 |
| 121 | 2005 | 18.87 | 10.46 | 68.21 | 59.08 | 6500000000 | 2700000000 | 2400000 | 27751.10 | 28.29 | 4.00 | 1.42 | 43.84 |
| 121 | 2006 | 21.25 | 13.75 | 68.42 | 60.33 | 6100000000 | 2500000000 | 2400000 | 26671.30 | 29.59 | 4.00 | 1.54 | 98.00 |
| 121 | 2007 | 20.56 | 13.67 | 66.96 | 60.50 | 7200000000 | 3100000000 | 2400000 | 32511.10 | 29.25 | 4.00 | 1.36 | 67.10 |
| 121 | 2008 | 22.08 | 14.96 | 68.21 | 61.00 | 7000000000 | 3000000000 | 2400000 | 31290.30 | 32.04 | 4.00 | 1.42 | 17.83 |
| 121 | 2009 | 23.9 | 12.21 | 69.08 | 66.50 | 6000000000 | 2600000000 | 2400000 | 28205.70 | 28.74 | 4.00 | 1.60 | 80.11 |
| 121 | 2010 | 22.27 | 12.04 | 66.13 | 66.38 | 6500000000 | 3000000000 | 2400000 | 33700.10 | 30.25 | 4.00 | 1.39 | 78.18 |
| 121 | 2011 | 20.31 | 12.46 | 63.29 | 64.88 | 7300000000 | 3500000000 | 2500000 | 38437.50 | 30.36 | 4.00 | 1.27 | 77.62 |
| 121 | 2012 | 19.54 | 12.25 | 62.83 | 64.00 | 7100000000 | 3700000000 | 2500000 | 39982.80 | 28.85 | 4.00 | 1.23 | 21.76 |
| 121 | 2013 | 18.42 | 12.58 | 63.00 | 61.25 | 7400000000 | 3900000000 | 2600000 | 42963.00 | 28.82 | 4.00 | 1.22 | 97.36 |
| 121 | 2014 | 17.12 | 12.71 | 62.04 | 59.50 | 8600000000 | 4100000000 | 2800000 | 44553.30 | 28.02 | 4.00 | 1.21 | 63.75 |
| 121 | 2015 | 18.56 | 11.79 | 65.33 | 60.00 | 9500000000 | 3700000000 | 3000000 | 38616.00 | 27.92 | 4.00 | 1.43 | 5.25 |
| 121 | 2016 | 17.1 | 11.83 | 62.63 | 59.75 | 9800000000 | 4000000000 | 3400000 | 40105.60 | 26.54 | 4.00 | 1.44 | 81.01 |
| 121 | 2017 | 16.44 | 13.38 | 61.08 | 58.42 | 11000000000 | 4500000000 | 3600000 | 42849.40 | 27.48 | 4.00 | 1.41 | 13.78 |
| 121 | 2018 | 17.4 | 14.42 | 61.63 | 58.75 | 11000000000 | 4600000000 | 3700000 | 42949.90 | 27.98 | 4.00 | 1.45 | 80.14 |
| 121 | 2019 | 17.1 | 14.04 | 61.17 | 59.00 | 11000000000 | 4700000000 | 474000 | 42084.40 | 34.92 | 4.00 | 96.46 | 12.72 |
| 122 | 2000 | 25.98 | 21.33 | 65.58 | 65.04 | 3800000000 | 1700000000 | 9700000 | 4624.28 | 66.86 | 7.00 | 282.18 | 87.27 |
| 122 | 2001 | 24.6 | 22.33 | 62.67 | 64.21 | 4200000000 | 1900000000 | 9800000 | 5276.03 | 64.88 | 8.00 | 286.49 | 23.71 |
| 122 | 2002 | 23.15 | 19.96 | 62.08 | 64.25 | 3800000000 | 2200000000 | 9700000 | 6655.33 | 58.14 | 10.00 | 257.89 | 21.64 |
| 122 | 2003 | 23.15 | 16.42 | 64.88 | 65.00 | 4100000000 | 2700000000 | 9800000 | 8421.10 | 56.33 | 10.00 | 224.31 | 50.45 |
| 122 | 2004 | 24.25 | 18.96 | 64.13 | 65.42 | 4000000000 | 2500000000 | 9600000 | 10285.80 | 59.65 | 10.00 | 202.75 | 23.62 |
| 122 | 2005 | 25.06 | 20.00 | 64.88 | 65.25 | 4800000000 | 2700000000 | 10000000 | 11200.60 | 62.64 | 10.00 | 199.58 | 76.12 |
| 122 | 2006 | 25.21 | 20.67 | 64.92 | 64.83 | 5000000000 | 2300000000 | 9300000 | 11475.80 | 73.87 | 10.00 | 210.39 | 61.76 |
| 122 | 2007 | 26.98 | 22.50 | 65.13 | 66.33 | 5600000000 | 3100000000 | 8600000 | 13919.00 | 77.94 | 10.00 | 183.63 | 20.77 |
| 122 | 2008 | 27.81 | 24.38 | 65.92 | 65.33 | 7500000000 | 3900000000 | 8800000 | 15753.50 | 79.28 | 10.00 | 172.11 | 63.85 |
| 122 | 2009 | 31.65 | 25.21 | 69.33 | 68.75 | 7000000000 | 3300000000 | 9100000 | 13046.50 | 74.40 | 10.00 | 202.34 | 36.84 |
| 122 | 2010 | 30.04 | 23.96 | 67.79 | 68.33 | 6600000000 | 2900000000 | 9500000 | 13113.50 | 81.75 | 10.00 | 207.94 | 56.17 |
| 122 | 2011 | 27.33 | 23.67 | 66.92 | 64.08 | 7200000000 | 3000000000 | 10000000 | 14151.00 | 86.59 | 10.00 | 201.06 | 27.86 |
| 122 | 2012 | 31.19 | 27.83 | 69.71 | 64.83 | 6100000000 | 2500000000 | 10000000 | 12918.20 | 86.32 | 10.00 | 225.10 | 10.22 |
| 122 | 2013 | 30.62 | 27.25 | 68.17 | 65.83 | 6700000000 | 2500000000 | 11000000 | 13687.20 | 85.58 | 10.00 | 223.70 | 15.34 |
| 122 | 2014 | 28.04 | 25.17 | 69.17 | 61.75 | 7500000000 | 2700000000 | 12000000 | 14246.10 | 87.42 | 10.00 | 232.60 | 96.36 |
| 122 | 2015 | 27.48 | 24.38 | 71.13 | 59.46 | 6900000000 | 2500000000 | 14000000 | 12651.60 | 87.98 | 10.00 | 279.33 | 73.79 |
| 122 | 2016 | 24.69 | 23.08 | 66.88 | 59.42 | 7500000000 | 2700000000 | 15000000 | 12992.40 | 87.15 | 10.00 | 281.52 | 91.73 |
| 122 | 2017 | 22.35 | 21.88 | 63.50 | 59.33 | 8400000000 | 3000000000 | 16000000 | 14457.60 | 87.14 | 10.00 | 274.43 | 93.01 |
| 122 | 2018 | 21.69 | 20.54 | 63.13 | 59.71 | 9600000000 | 3200000000 | 18000000 | 16150.80 | 84.94 | 10.00 | 270.21 | 89.69 |
| 122 | 2019 | 22.98 | 21.38 | 64.33 | 60.25 | 10000000000 | 3400000000 | 3900000 | 16731.80 | 82.19 | 10.00 | 1.48 | 33.82 |
| 123 | 2000 | 30.44 | 33.83 | 66.88 | 60.17 | 1500000000 | 870000000 | 2100000 | 1177.63 | 35.39 | 4.00 | 11.23 | 70.33 |
| 123 | 2001 | 28.44 | 34.33 | 62.38 | 60.17 | 1600000000 | 830000000 | 2200000 | 1258.45 | 35.38 | 4.00 | 11.23 | 13.24 |
| 123 | 2002 | 29.71 | 34.54 | 62.63 | 62.25 | 1400000000 | 780000000 | 2700000 | 1263.26 | 38.07 | 4.00 | 11.23 | 29.64 |
| 123 | 2003 | 29.33 | 35.21 | 61.08 | 62.38 | 880000000 | 730000000 | 2600000 | 1253.40 | 33.47 | 4.00 | 11.23 | 80.74 |
| 123 | 2004 | 31.35 | 39.21 | 61.38 | 62.13 | 1900000000 | 690000000 | 3400000 | 1407.18 | 40.05 | 4.00 | 11.23 | 94.18 |
| 123 | 2005 | 32.4 | 40.88 | 60.92 | 63.00 | 2000000000 | 580000000 | 3600000 | 1571.74 | 40.39 | 4.00 | 11.23 | 34.11 |
| 123 | 2006 | 32.96 | 41.75 | 60.63 | 63.54 | 2100000000 | 590000000 | 4200000 | 1748.91 | 39.50 | 5.00 | 11.23 | 81.63 |
| 123 | 2007 | 34.25 | 42.17 | 60.83 | 65.50 | 3000000000 | 710000000 | 4200000 | 2032.62 | 38.64 | 5.00 | 11.23 | 78.89 |
| 123 | 2008 | 34.85 | 42.58 | 59.58 | 67.54 | 3200000000 | 910000000 | 5400000 | 2316.34 | 37.78 | 5.00 | 11.23 | 30.87 |
| 123 | 2009 | 34.65 | 42.50 | 57.21 | 69.58 | 3800000000 | 980000000 | 6100000 | 2600.06 | 36.92 | 5.00 | 11.23 | 48.95 |
| 123 | 2010 | 33.25 | 42.00 | 57.00 | 67.50 | 6300000000 | 1600000000 | 8500000 | 2883.78 | 36.06 | 5.00 | 11.23 | 52.89 |
| 123 | 2011 | 36.87 | 49.42 | 57.00 | 67.33 | 1800000000 | 850000000 | 5100000 | 3167.49 | 35.20 | 6.00 | 48.34 | 16.00 |
| 123 | 2012 | 47.94 | 55.42 | 62.21 | 78.25 | 1800000000 | 1200000000 | 3100000 | 3451.21 | 34.34 | 6.00 | 64.58 | 77.33 |
| 123 | 2013 | 54.04 | 58.71 | 65.63 | 83.75 | 1800000000 | 1000000000 | 1100000 | 3734.93 | 33.48 | 6.00 | 108.73 | 55.50 |
| 123 | 2014 | 57.35 | 58.96 | 69.17 | 86.58 | 1800000000 | 1100000000 | 930000 | 4018.65 | 32.62 | 6.00 | 154.13 | 64.68 |
| 123 | 2015 | 64.02 | 59.50 | 75.13 | 93.42 | 1800000000 | 1200000000 | 790000 | 3902.00 | 31.75 | 6.00 | 237.03 | 40.70 |
| 123 | 2016 | 55 | 58.33 | 66.58 | 85.08 | 1800000000 | 1300000000 | 650000 | 3785.35 | 30.89 | 6.00 | 460.28 | 64.31 |
| 123 | 2017 | 53 | 53.38 | 69.08 | 83.54 | 1800000000 | 1400000000 | 510000 | 3668.70 | 30.03 | 6.00 | 492.61 | 94.87 |
| 123 | 2018 | 51.27 | 50.75 | 69.00 | 82.79 | 1800000000 | 1500000000 | 370000 | 3552.06 | 29.17 | 6.00 | 524.95 | 23.71 |
| 123 | 2019 | 46.67 | 50.04 | 62.92 | 80.38 | 1800000000 | 1600000000 | 61000000 | 17843.90 | 82.73 | 6.00 | 265.99 | 58.89 |
| 124 | 2000 | 31.42 | 26.17 | 64.71 | 71.96 | 1600000000 | 240000000 | 1300000 | 3384.69 | 38.79 | 0.00 | 42.99 | 70.32 |
| 124 | 2001 | 28.93 | 26.04 | 63.75 | 68.08 | 1500000000 | 230000000 | 1300000 | 3431.42 | 35.56 | 0.00 | 46.00 | 84.60 |
| 124 | 2002 | 30.92 | 30.04 | 63.71 | 68.08 | 1500000000 | 270000000 | 1300000 | 3599.97 | 32.76 | 0.00 | 48.42 | 69.89 |
| 124 | 2003 | 31.73 | 29.54 | 64.88 | 69.04 | 1600000000 | 270000000 | 1400000 | 3469.15 | 36.61 | 0.00 | 57.74 | 90.51 |
| 124 | 2004 | 32.4 | 29.54 | 64.33 | 70.92 | 1700000000 | 320000000 | 1400000 | 3725.11 | 37.54 | 0.00 | 61.20 | 84.45 |
| 124 | 2005 | 30.25 | 27.42 | 62.67 | 70.42 | 1800000000 | 290000000 | 1500000 | 4089.20 | 35.27 | 0.00 | 62.28 | 82.01 |
| 124 | 2006 | 29.92 | 26.63 | 63.71 | 69.50 | 2100000000 | 320000000 | 1700000 | 4321.04 | 40.13 | 0.00 | 65.74 | 90.81 |
| 124 | 2007 | 29 | 27.17 | 63.00 | 67.83 | 2100000000 | 340000000 | 1700000 | 4633.94 | 39.73 | 0.00 | 69.19 | 76.75 |
| 124 | 2008 | 30.42 | 26.96 | 64.17 | 69.71 | 2200000000 | 310000000 | 1800000 | 4917.72 | 41.94 | 0.00 | 72.76 | 36.64 |
| 124 | 2009 | 36.5 | 28.00 | 69.46 | 75.54 | 2100000000 | 260000000 | 1800000 | 4316.23 | 34.48 | 0.00 | 87.89 | 55.07 |
| 124 | 2010 | 34.62 | 28.13 | 64.88 | 76.25 | 2100000000 | 240000000 | 1900000 | 4704.05 | 31.34 | 0.00 | 87.20 | 64.74 |
| 124 | 2011 | 32.37 | 28.13 | 64.04 | 72.58 | 2100000000 | 210000000 | 2000000 | 5111.47 | 30.37 | 0.00 | 85.89 | 26.72 |
| 124 | 2012 | 33.12 | 27.50 | 67.08 | 71.67 | 2000000000 | 420000000 | 2000000 | 5209.85 | 30.18 | 0.00 | 88.75 | 49.36 |
| 124 | 2013 | 37.27 | 30.71 | 70.58 | 73.25 | 2000000000 | 390000000 | 2000000 | 4989.17 | 30.58 | 0.00 | 100.40 | 64.04 |
| 124 | 2014 | 33.29 | 29.29 | 68.50 | 68.79 | 2000000000 | 460000000 | 2100000 | 4833.79 | 31.26 | 0.00 | 110.94 | 81.58 |
| 124 | 2015 | 29.54 | 26.58 | 66.54 | 65.96 | 1900000000 | 410000000 | 2100000 | 4907.50 | 29.92 | 1.00 | 116.97 | 63.08 |
| 124 | 2016 | 28.73 | 26.25 | 66.50 | 64.71 | 1900000000 | 410000000 | 2200000 | 4843.34 | 31.82 | 1.00 | 125.10 | 32.00 |
| 124 | 2017 | 27.92 | 26.00 | 65.33 | 64.50 | 1900000000 | 460000000 | 2400000 | 5069.18 | 34.66 | 1.00 | 127.97 | 32.34 |
| 124 | 2018 | 29.19 | 27.96 | 65.42 | 65.00 | 1800000000 | 500000000 | 2500000 | 5354.24 | 37.96 | 1.00 | 128.87 | 90.42 |
| 124 | 2019 | 28.67 | 27.71 | 65.04 | 64.58 | 1800000000 | 550000000 | 2600000 | 5582.26 | 37.96 | 1.00 | 557.28 | 66.79 |
| 125 | 2000 | 43.4 | 45.17 | 68.21 | 73.42 | 52000000 | 56000000 | 45000 | 622.74 | 22.16 | 4.00 | 539.53 | 58.14 |
| 125 | 2001 | 40.31 | 42.38 | 68.88 | 69.38 | 81000000 | 59000000 | 123000 | 694.42 | 24.15 | 4.00 | 555.08 | 27.85 |
| 125 | 2002 | 39.81 | 41.08 | 69.04 | 69.50 | 81000000 | 85000000 | 162000 | 783.24 | 27.83 | 4.00 | 573.35 | 82.33 |
| 125 | 2003 | 39.27 | 40.71 | 69.00 | 68.83 | 90000000 | 97000000 | 206000 | 930.13 | 30.48 | 4.00 | 578.76 | 54.30 |
| 125 | 2004 | 35.23 | 39.83 | 65.25 | 65.38 | 190000000 | 200000000 | 263000 | 1191.92 | 28.19 | 4.00 | 533.45 | 77.33 |
| 125 | 2005 | 33.65 | 41.63 | 61.17 | 64.50 | 240000000 | 260000000 | 319000 | 1643.75 | 27.31 | 4.00 | 457.69 | 49.04 |
| 125 | 2006 | 33.1 | 40.83 | 60.88 | 64.50 | 410000000 | 300000000 | 382000 | 2158.14 | 22.15 | 4.00 | 416.04 | 77.62 |
| 125 | 2007 | 31.79 | 38.21 | 60.88 | 64.50 | 520000000 | 430000000 | 511000 | 3139.28 | 18.19 | 4.00 | 342.08 | 98.45 |
| 125 | 2008 | 32.44 | 39.96 | 60.42 | 64.50 | 560000000 | 490000000 | 558000 | 4010.86 | 14.27 | 4.00 | 305.97 | 83.53 |
| 125 | 2009 | 36.02 | 39.38 | 63.79 | 68.88 | 570000000 | 500000000 | 575000 | 2994.34 | 14.67 | 4.00 | 363.28 | 79.80 |
| 125 | 2010 | 37.37 | 38.83 | 62.42 | 73.50 | 690000000 | 640000000 | 684000 | 3218.37 | 19.75 | 4.00 | 373.66 | 46.92 |
| 125 | 2011 | 36.4 | 40.21 | 62.08 | 70.50 | 760000000 | 760000000 | 758000 | 3525.80 | 22.52 | 4.00 | 372.50 | 21.87 |
| 125 | 2012 | 36.25 | 40.50 | 64.83 | 67.17 | 850000000 | 870000000 | 963000 | 3681.86 | 27.57 | 4.00 | 401.76 | 84.52 |
| 125 | 2013 | 36.35 | 41.00 | 65.50 | 66.21 | 910000000 | 1000000000 | 1100000 | 3838.19 | 28.36 | 4.00 | 409.63 | 89.36 |
| 125 | 2014 | 37.44 | 43.38 | 65.00 | 66.50 | 990000000 | 1100000000 | 1200000 | 3986.23 | 28.57 | 4.00 | 415.92 | 15.68 |
| 125 | 2015 | 38.58 | 44.00 | 67.50 | 65.67 | 960000000 | 1100000000 | 1200000 | 3607.30 | 29.73 | 4.00 | 477.92 | 87.48 |
| 125 | 2016 | 37.6 | 43.71 | 65.50 | 66.00 | 1000000000 | 1000000000 | 1300000 | 3591.83 | 33.15 | 4.00 | 480.49 | 86.55 |
| 125 | 2017 | 36.9 | 42.29 | 65.50 | 66.00 | 1200000000 | 1200000000 | 1500000 | 3914.50 | 37.33 | 4.00 | 482.72 | 12.82 |
| 125 | 2018 | 35 | 40.50 | 64.17 | 65.33 | 1400000000 | 1300000000 | 1700000 | 4220.49 | 37.73 | 4.00 | 482.99 | 90.00 |
| 125 | 2019 | 31.5 | 35.63 | 63.50 | 63.88 | 1600000000 | 1500000000 | 4200000 | 4622.73 | 41.19 | 4.00 | 129.78 | 21.82 |
| 126 | 2000 | 37.77 | 38.67 | 70.88 | 66.00 | 960000000 | 130000000 | 73000 | 554.45 | 51.25 | 3.00 | 161.72 | 64.99 |
| 126 | 2001 | 34.43 | 38.67 | 66.46 | 63.75 | 1000000000 | 140000000 | 76000 | 550.36 | 50.43 | 3.00 | 168.67 | 98.64 |
| 126 | 2002 | 33.77 | 39.79 | 65.25 | 62.50 | 1100000000 | 140000000 | 98000 | 579.85 | 49.30 | 3.00 | 175.63 | 61.81 |
| 126 | 2003 | 33.29 | 38.00 | 65.08 | 63.50 | 1100000000 | 130000000 | 155000 | 620.38 | 52.84 | 3.00 | 183.45 | 70.43 |
| 126 | 2004 | 33.08 | 37.67 | 65.00 | 63.50 | 1100000000 | 180000000 | 274000 | 709.97 | 57.47 | 3.00 | 184.78 | 79.13 |
| 126 | 2005 | 31.44 | 38.38 | 61.50 | 63.00 | 1200000000 | 220000000 | 336000 | 832.84 | 62.93 | 3.00 | 191.51 | 64.78 |
| 126 | 2006 | 30.85 | 37.21 | 61.50 | 63.00 | 1100000000 | 230000000 | 1100000 | 921.42 | 62.40 | 3.00 | 197.05 | 88.66 |
| 126 | 2007 | 35.06 | 37.42 | 61.00 | 71.71 | 1000000000 | 250000000 | 948000 | 1017.29 | 64.43 | 3.00 | 198.95 | 99.01 |
| 126 | 2008 | 35.65 | 38.42 | 60.88 | 72.00 | 1200000000 | 250000000 | 1000000 | 1229.25 | 68.67 | 4.00 | 199.76 | 77.54 |
| 126 | 2009 | 34.58 | 41.42 | 59.50 | 68.25 | 1100000000 | 280000000 | 1000000 | 1116.08 | 65.14 | 4.00 | 202.85 | 70.12 |
| 126 | 2010 | 36.77 | 43.50 | 61.04 | 69.00 | 1300000000 | 250000000 | 1000000 | 1334.78 | 74.86 | 4.00 | 219.59 | 43.46 |
| 126 | 2011 | 41.23 | 48.58 | 61.63 | 72.25 | 910000000 | 260000000 | 829000 | 1374.62 | 81.45 | 4.00 | 213.80 | 17.79 |
| 126 | 2012 | 41.83 | 50.00 | 59.50 | 74.17 | 1000000000 | 150000000 | 874000 | 1446.54 | 85.90 | 4.00 | 214.35 | 97.32 |
| 126 | 2013 | 40 | 50.00 | 56.83 | 73.17 | 1100000000 | 160000000 | 990000 | 1607.15 | 87.96 | 4.00 | 214.89 | 87.04 |
| 126 | 2014 | 38.54 | 50.33 | 57.38 | 69.38 | 1200000000 | 160000000 | 1000000 | 1673.15 | 84.90 | 4.00 | 214.89 | 13.41 |
| 126 | 2015 | 45.94 | 60.38 | 57.50 | 74.00 | 120000000 | 79000000 | 366700 | 1395.44 | 79.00 | 4.00 | 214.89 | 74.82 |
| 126 | 2016 | 49.56 | 60.58 | 60.88 | 77.67 | 120000000 | 56000000 | 183350 | 1033.73 | 74.21 | 4.00 | 241.27 | 80.86 |
| 126 | 2017 | 48.81 | 58.83 | 61.04 | 77.75 | 120000000 | 68000000 | 91675 | 882.40 | 75.98 | 4.00 | 250.25 | 64.31 |
| 126 | 2018 | 50.96 | 59.00 | 62.75 | 80.17 | 120000000 | 45000000 | 4583 | 968.16 | 75.65 | 4.00 | 250.25 | 82.04 |
| 126 | 2019 | 49.67 | 58.00 | 64.00 | 77.33 | 120000000 | 22000000 | 1900000 | 774.33 | 38.13 | 4.00 | 483.26 | 64.61 |
| 127 | 2000 | 53.48 | 67.42 | 68.04 | 71.50 | 270000000 | 1100000000 | 78000 | 735.19 | 75.70 | 1.00 | 2002.40 | 88.89 |
| 127 | 2001 | 51.81 | 62.00 | 69.17 | 72.46 | 250000000 | 1000000000 | 127000 | 799.60 | 65.27 | 1.00 | 2002.40 | 21.17 |
| 127 | 2002 | 53.69 | 64.63 | 69.00 | 73.75 | 230000000 | 930000000 | 200000 | 864.00 | 70.57 | 1.00 | 2002.37 | 9.18 |
| 127 | 2003 | 59.33 | 65.08 | 74.25 | 79.33 | 220000000 | 830000000 | 273000 | 928.41 | 77.39 | 2.00 |  | 11.03 |
| 127 | 2004 | 60.81 | 64.92 | 82.79 | 73.92 | 200000000 | 730000000 | 346000 | 1391.96 | 56.27 | 2.00 | 1453.42 | 64.13 |
| 127 | 2005 | 63.6 | 65.46 | 80.00 | 81.75 | 190000000 | 630000000 | 419000 | 1855.52 | 54.35 | 2.00 | 1472.00 | 73.48 |
| 127 | 2006 | 52.15 | 64.79 | 65.71 | 73.79 | 170000000 | 530000000 | 492000 | 2373.21 | 51.03 | 2.00 | 1467.42 | 62.68 |
| 127 | 2007 | 51.79 | 66.79 | 65.50 | 71.29 | 560000000 | 710000000 | 565000 | 3182.95 | 45.90 | 3.00 | 1254.57 | 38.99 |
| 127 | 2008 | 46.81 | 64.46 | 63.83 | 65.33 | 870000000 | 810000000 | 864000 | 4636.61 | 50.33 | 3.00 | 1193.08 | 78.90 |
| 127 | 2009 | 39.46 | 58.83 | 56.67 | 63.42 | 1400000000 | 1200000000 | 1300000 | 3853.94 | 39.40 | 3.00 | 1170.00 | 32.47 |
| 127 | 2010 | 41.23 | 59.29 | 57.42 | 65.75 | 1700000000 | 1700000000 | 1500000 | 4657.28 | 39.42 | 3.00 | 1170.00 | 96.02 |
| 127 | 2011 | 41.29 | 56.33 | 57.63 | 68.63 | 1600000000 | 1900000000 | 1500000 | 6045.50 | 44.42 | 3.00 | 1170.00 | 92.57 |
| 127 | 2012 | 39.42 | 58.00 | 57.08 | 63.75 | 1600000000 | 2400000000 | 1100000 | 6836.03 | 44.45 | 3.00 | 1166.17 | 71.52 |
| 127 | 2013 | 38.08 | 60.38 | 55.33 | 60.46 | 1700000000 | 4800000000 | 892000 | 7076.55 | 39.68 | 3.00 | 1166.00 | 34.77 |
| 127 | 2014 | 37.73 | 61.13 | 54.83 | 59.50 | 1900000000 | 6100000000 | 673000 | 6818.80 | 41.34 | 4.00 | 1166.00 | 21.83 |
| 127 | 2015 | 41.94 | 61.29 | 55.83 | 66.75 | 2800000000 | 7900000000 | 454000 | 4989.80 | 35.34 | 4.00 | 1167.33 | 4.10 |
| 127 | 2016 | 43.62 | 60.04 | 58.50 | 68.71 | 3100000000 | 7600000000 | 335000 | 4776.73 | 32.10 | 5.00 | 1182.00 | 94.24 |
| 127 | 2017 | 44.31 | 59.42 | 60.17 | 69.04 | 3000000000 | 8100000000 | 216000 | 5205.29 | 37.96 | 5.00 | 1184.00 | 41.10 |
| 127 | 2018 | 40.06 | 55.54 | 59.13 | 65.46 | 2000000000 | 7900000000 | 97000 | 5834.17 | 43.98 | 5.00 | 1182.75 | 52.59 |
| 127 | 2019 | 33.98 | 51.54 | 56.33 | 60.08 | 3600000000 | 11000000000 | 156500 | 5955.11 | 38.05 | 5.00 | 250.25 | 80.69 |
| 128 | 2000 | 34.48 | 38.17 | 65.17 | 65.63 | 680000000 | 670000000 | 1600000 | 1670.01 | 21.47 | 3.00 | 1764.86 | 30.99 |
| 128 | 2001 | 29.62 | 40.29 | 58.75 | 60.21 | 1100000000 | 710000000 | 1700000 | 1909.41 | 19.30 | 3.00 | 1753.99 | 23.20 |
| 128 | 2002 | 33.19 | 41.17 | 63.58 | 61.63 | 1600000000 | 4000000000 | 1900000 | 1911.68 | 24.41 | 3.00 | 6907.03 | 99.65 |
| 128 | 2003 | 32.98 | 41.63 | 61.50 | 62.83 | 1300000000 | 4100000000 | 2000000 | 2253.94 | 24.63 | 4.00 | 8193.89 | 37.55 |
| 128 | 2004 | 29.83 | 40.08 | 54.92 | 64.67 | 1300000000 | 4400000000 | 2200000 | 2756.20 | 25.27 | 6.00 | 8613.99 | 99.60 |
| 128 | 2005 | 29.58 | 39.13 | 55.54 | 64.50 | 1000000000 | 4100000000 | 2400000 | 3246.05 | 30.33 | 8.00 | 8963.96 | 75.29 |
| 128 | 2006 | 27.83 | 38.42 | 54.33 | 62.92 | 1500000000 | 5100000000 | 2500000 | 3774.36 | 29.87 | 9.00 | 9170.94 | 19.05 |
| 128 | 2007 | 32.4 | 41.50 | 55.25 | 68.04 | 2000000000 | 7300000000 | 2200000 | 4904.67 | 28.84 | 9.00 | 9281.15 | 83.58 |
| 128 | 2008 | 29.77 | 43.25 | 54.38 | 61.92 | 2000000000 | 8300000000 | 2000000 | 5717.31 | 26.58 | 10.00 | 9428.53 | 80.25 |
| 128 | 2009 | 32.5 | 44.38 | 52.25 | 68.38 | 2300000000 | 8500000000 | 2100000 | 5709.95 | 22.71 | 11.00 | 9864.30 | 79.72 |
| 128 | 2010 | 33.21 | 48.13 | 52.38 | 65.92 | 2600000000 | 11000000000 | 2900000 | 6599.66 | 24.40 | 13.00 | 10254.20 | 23.25 |
| 128 | 2011 | 33.87 | 49.83 | 53.29 | 64.63 | 2500000000 | 11000000000 | 3400000 | 7781.41 | 25.63 | 14.00 | 10616.30 | 97.06 |
| 128 | 2012 | 37.85 | 51.00 | 55.50 | 69.21 | 2500000000 | 8400000000 | 3800000 | 7927.85 | 24.13 | 16.00 | 12175.50 | 70.68 |
| 128 | 2013 | 39.77 | 50.29 | 55.50 | 73.75 | 3300000000 | 9500000000 | 4800000 | 6018.32 | 26.88 | 17.00 | 18414.40 | 5.79 |
| 128 | 2014 | 39.48 | 46.96 | 63.13 | 68.88 | 4200000000 | 10000000000 | 5000000 | 5585.53 | 23.14 | 18.00 | 25941.70 | 81.66 |
| 128 | 2015 | 33.81 | 43.04 | 56.58 | 68.00 | 4800000000 | 9700000000 | 5200000 | 4904.33 | 19.74 | 19.00 | 29011.50 | 16.09 |
| 128 | 2016 | 28.58 | 41.21 | 53.54 | 62.42 | 3900000000 | 9900000000 | 4900000 | 5253.42 | 22.40 | 21.00 | 30914.90 | 81.20 |
| 128 | 2017 | 26.96 | 40.00 | 51.79 | 62.13 | 4600000000 | 12000000000 | 4900000 | 5520.31 | 24.94 | 22.00 | 33226.30 | 14.33 |
| 128 | 2018 | 30.25 | 43.29 | 53.67 | 63.54 | 5400000000 | 14000000000 | 7300000 | 5787.20 | 27.48 | 23.00 | 40864.30 | 88.50 |
| 128 | 2019 | 35.42 | 43.96 | 52.21 | 74.67 | 6100000000 | 16000000000 | 9700000 | 6463.04 | 25.27 | 23.00 | 1181.50 | 47.13 |
| 129 | 2000 | 30.65 | 39.67 | 61.13 | 60.50 | 4600000000 | 3700000000 | 2400000 | 21043.60 | 35.57 | 0.00 | 4.08 | 25.26 |
| 129 | 2001 | 31.16 | 39.00 | 61.38 | 61.96 | 2900000000 | 3900000000 | 1200000 | 20306.90 | 31.30 | 2.00 | 4.21 | 55.05 |
| 129 | 2002 | 33.17 | 39.71 | 63.08 | 63.54 | 2400000000 | 3300000000 | 861900 | 18435.90 | 32.89 | 2.00 | 4.74 | 22.17 |
| 129 | 2003 | 30.37 | 34.92 | 61.21 | 64.63 | 2500000000 | 3300000000 | 1100000 | 18979.30 | 34.63 | 3.00 | 4.55 | 81.25 |
| 129 | 2004 | 28.25 | 35.33 | 60.83 | 60.33 | 2900000000 | 3700000000 | 1500000 | 19896.80 | 39.12 | 3.00 | 4.48 | 64.80 |
| 129 | 2005 | 27.75 | 33.92 | 61.33 | 60.25 | 3800000000 | 4100000000 | 1900000 | 20566.60 | 40.77 | 5.00 | 4.49 | 24.03 |
| 129 | 2006 | 28.48 | 37.54 | 60.38 | 59.04 | 3700000000 | 4300000000 | 1800000 | 21837.40 | 40.78 | 5.00 | 4.46 | 65.77 |
| 129 | 2007 | 27.21 | 37.58 | 59.71 | 57.13 | 4300000000 | 4900000000 | 2100000 | 24924.30 | 40.46 | 5.00 | 4.11 | 42.64 |
| 129 | 2008 | 28.15 | 38.00 | 60.79 | 57.50 | 5400000000 | 5000000000 | 2600000 | 29567.80 | 38.47 | 6.00 | 3.59 | 58.33 |
| 129 | 2009 | 31.06 | 36.83 | 61.79 | 63.50 | 4900000000 | 4500000000 | 2300000 | 27715.60 | 33.19 | 6.00 | 3.93 | 31.15 |
| 129 | 2010 | 26.83 | 35.21 | 58.71 | 59.75 | 5600000000 | 4700000000 | 2800000 | 30693.60 | 34.72 | 6.00 | 3.74 | 5.25 |
| 129 | 2011 | 26.25 | 35.13 | 58.13 | 59.25 | 5900000000 | 4900000000 | 2800000 | 33669.20 | 35.33 | 6.00 | 3.58 | 20.51 |
| 129 | 2012 | 26.87 | 33.00 | 60.00 | 60.75 | 6200000000 | 5000000000 | 2900000 | 32511.20 | 35.95 | 7.00 | 3.86 | 96.49 |
| 129 | 2013 | 26.31 | 32.96 | 59.46 | 60.21 | 6600000000 | 5800000000 | 3000000 | 36309.50 | 33.67 | 7.00 | 3.61 | 74.66 |
| 129 | 2014 | 25.69 | 33.96 | 58.29 | 59.13 | 6600000000 | 6500000000 | 2900000 | 37678.90 | 32.16 | 8.00 | 3.58 | 91.28 |
| 129 | 2015 | 24.31 | 33.00 | 57.75 | 57.88 | 6600000000 | 7500000000 | 2800000 | 35776.80 | 31.21 | 9.00 | 3.89 | 91.33 |
| 129 | 2016 | 22.37 | 32.04 | 55.29 | 57.42 | 6600000000 | 8200000000 | 2900000 | 37321.60 | 29.69 | 9.00 | 3.84 | 89.41 |
| 129 | 2017 | 21.9 | 32.17 | 54.33 | 57.29 | 7600000000 | 9000000000 | 3600000 | 40541.90 | 28.68 | 9.00 | 3.60 | 31.41 |
| 129 | 2018 | 22.06 | 32.71 | 54.42 | 57.00 | 8000000000 | 10000000000 | 4100000 | 41719.70 | 29.45 | 9.00 | 3.59 | 73.36 |
| 129 | 2019 | 22.69 | 33.08 | 54.29 | 58.00 | 8400000000 | 10000000000 | 9100000 | 43592.10 | 29.32 | 9.00 | 48502.40 | 14.70 |
| 130 | 2000 | 21.6 | 23.58 | 61.54 | 58.08 | 29000000000 | 18000000000 | 41000000 | 20087.60 | 25.63 | 36.00 | 0.66 | 33.62 |
| 130 | 2001 | 18.17 | 17.50 | 59.83 | 59.00 | 27000000000 | 17000000000 | 40000000 | 20483.20 | 25.65 | 37.00 | 0.69 | 84.43 |
| 130 | 2002 | 19.6 | 18.96 | 59.79 | 60.46 | 28000000000 | 20000000000 | 40000000 | 22270.10 | 24.41 | 38.00 | 0.67 | 93.31 |
| 130 | 2003 | 20.17 | 21.42 | 57.92 | 61.00 | 33000000000 | 24000000000 | 40000000 | 27465.70 | 23.30 | 40.00 | 0.61 | 51.83 |
| 130 | 2004 | 20.65 | 22.54 | 57.33 | 61.42 | 38000000000 | 24000000000 | 37000000 | 31259.70 | 24.01 | 42.00 | 0.55 | 80.45 |
| 130 | 2005 | 22.46 | 22.17 | 60.50 | 62.25 | 38000000000 | 27000000000 | 37000000 | 32043.10 | 24.60 | 43.00 | 0.55 | 79.75 |
| 130 | 2006 | 23.65 | 22.08 | 62.96 | 62.25 | 42000000000 | 27000000000 | 41000000 | 33501.70 | 26.17 | 44.00 | 0.54 | 47.64 |
| 130 | 2007 | 22.6 | 20.96 | 63.08 | 61.17 | 46000000000 | 33000000000 | 44000000 | 37822.70 | 27.36 | 45.00 | 0.50 | 52.49 |
| 130 | 2008 | 23.33 | 21.17 | 63.00 | 62.50 | 46000000000 | 30000000000 | 43000000 | 40778.30 | 26.86 | 47.00 | 0.54 | 56.82 |
| 130 | 2009 | 25.27 | 20.54 | 63.58 | 66.42 | 45000000000 | 31000000000 | 43000000 | 37079.80 | 22.40 | 48.00 | 0.64 | 18.50 |
| 130 | 2010 | 25.87 | 22.50 | 63.46 | 65.79 | 44000000000 | 31000000000 | 44000000 | 36000.50 | 25.07 | 49.00 | 0.65 | 81.36 |
| 130 | 2011 | 27 | 25.79 | 64.08 | 64.13 | 44000000000 | 31000000000 | 46000000 | 38599.10 | 26.87 | 53.00 | 0.62 | 64.19 |
| 130 | 2012 | 29.12 | 26.46 | 65.79 | 66.00 | 43000000000 | 31000000000 | 46000000 | 35053.50 | 28.38 | 53.00 | 0.63 | 71.04 |
| 130 | 2013 | 29.31 | 28.71 | 65.08 | 64.83 | 43000000000 | 31000000000 | 48000000 | 35550.00 | 28.63 | 55.00 | 0.64 | 46.90 |
| 130 | 2014 | 27.83 | 27.75 | 64.96 | 62.96 | 42000000000 | 31000000000 | 49000000 | 35518.40 | 29.11 | 56.00 | 0.61 | 68.63 |
| 130 | 2015 | 27.17 | 25.63 | 67.38 | 61.33 | 41000000000 | 30000000000 | 51000000 | 30230.20 | 29.72 | 57.00 | 0.65 | 95.00 |
| 130 | 2016 | 24.75 | 24.42 | 64.54 | 60.54 | 42000000000 | 31000000000 | 52000000 | 30939.70 | 29.33 | 57.00 | 0.74 | 25.54 |
| 130 | 2017 | 24.46 | 24.17 | 64.92 | 59.83 | 47000000000 | 35000000000 | 58000000 | 32406.70 | 30.73 | 59.00 | 0.78 | 62.55 |
| 130 | 2018 | 23.87 | 23.21 | 64.63 | 59.92 | 52000000000 | 38000000000 | 62000000 | 34520.10 | 31.45 | 60.00 | 0.75 | 74.38 |
| 130 | 2019 | 24.02 | 22.29 | 65.25 | 60.50 | 52000000000 | 38000000000 | 4900000 | 33228.20 | 31.50 | 60.00 | 3.58 | 86.11 |
| 131 | 2000 | 36.19 | 45.08 | 60.88 | 66.42 | 3600000000 | 3700000000 | 2600000 | 443.31 | 13.00 | 22.00 | 44.94 | 72.24 |
| 131 | 2001 | 35 | 43.67 | 59.88 | 66.46 | 3300000000 | 4400000000 | 2500000 | 451.57 | 12.56 | 22.00 | 47.19 | 91.74 |
| 131 | 2002 | 34.29 | 44.08 | 59.00 | 65.50 | 3300000000 | 4400000000 | 2400000 | 470.99 | 14.26 | 23.00 | 48.61 | 88.17 |
| 131 | 2003 | 32.1 | 41.54 | 57.17 | 65.50 | 4600000000 | 4400000000 | 2700000 | 546.73 | 14.95 | 24.00 | 46.58 | 86.01 |
| 131 | 2004 | 28.4 | 36.83 | 55.63 | 64.33 | 6300000000 | 5800000000 | 3500000 | 627.77 | 17.86 | 26.00 | 45.32 | 93.15 |
| 131 | 2005 | 28.27 | 36.29 | 55.75 | 64.50 | 7700000000 | 8300000000 | 3900000 | 714.86 | 19.61 | 28.00 | 44.10 | 76.07 |
| 131 | 2006 | 28.77 | 36.46 | 56.63 | 64.46 | 8900000000 | 8700000000 | 4400000 | 806.75 | 21.27 | 28.00 | 45.31 | 39.02 |
| 131 | 2007 | 29.33 | 37.54 | 56.63 | 64.50 | 11000000000 | 11000000000 | 5100000 | 1028.33 | 20.80 | 29.00 | 41.35 | 65.78 |
| 131 | 2008 | 32.15 | 39.17 | 57.71 | 67.42 | 12000000000 | 12000000000 | 5300000 | 998.52 | 24.10 | 30.00 | 43.51 | 68.25 |
| 131 | 2009 | 30.29 | 36.83 | 57.25 | 66.50 | 14000000000 | 13000000000 | 5200000 | 1101.96 | 20.40 | 30.00 | 48.41 | 28.86 |
| 131 | 2010 | 30.25 | 37.96 | 55.46 | 67.08 | 15000000000 | 15000000000 | 5800000 | 1357.56 | 22.40 | 31.00 | 45.73 | 75.00 |
| 131 | 2011 | 32.35 | 41.67 | 56.42 | 66.63 | 16000000000 | 16000000000 | 6300000 | 1458.10 | 24.54 | 31.00 | 46.67 | 70.00 |
| 131 | 2012 | 34.27 | 42.38 | 59.17 | 67.00 | 18000000000 | 14000000000 | 6600000 | 1443.88 | 24.53 | 32.00 | 53.44 | 83.73 |
| 131 | 2013 | 33.79 | 41.17 | 58.96 | 67.46 | 19000000000 | 14000000000 | 7000000 | 1449.61 | 25.43 | 33.00 | 58.60 | 74.39 |
| 131 | 2014 | 32.15 | 39.54 | 57.79 | 66.96 | 21000000000 | 17000000000 | 13000000 | 1573.88 | 22.97 | 35.00 | 61.03 | 20.08 |
| 131 | 2015 | 30.48 | 38.38 | 57.75 | 64.83 | 21000000000 | 18000000000 | 13000000 | 1605.61 | 19.81 | 35.00 | 64.15 | 39.90 |
| 131 | 2016 | 29.31 | 37.13 | 57.00 | 64.50 | 23000000000 | 19000000000 | 15000000 | 1732.56 | 19.16 | 38.00 | 67.20 | 90.69 |
| 131 | 2017 | 29.33 | 39.17 | 56.00 | 63.50 | 28000000000 | 22000000000 | 16000000 | 1981.65 | 18.78 | 40.00 | 65.12 | 71.28 |
| 131 | 2018 | 29.75 | 38.71 | 56.71 | 64.08 | 29000000000 | 26000000000 | 17000000 | 2005.86 | 19.85 | 41.00 | 68.39 | 69.85 |
| 131 | 2019 | 29 | 38.00 | 56.42 | 63.58 | 32000000000 | 29000000000 | 95000000 | 2099.60 | 18.41 | 41.00 | 0.72 | 14.30 |
| 132 | 2000 | 43.42 | 54.92 | 66.88 | 65.04 | 5500000000 | 4400000000 | 5100000 | 780.19 | 40.98 | 6.00 | 8421.78 | 81.10 |
| 132 | 2001 | 44.7 | 55.54 | 68.67 | 65.21 | 5100000000 | 4700000000 | 5200000 | 748.26 | 39.03 | 6.00 | 10260.80 | 55.40 |
| 132 | 2002 | 41.77 | 51.88 | 66.04 | 65.63 | 5800000000 | 5000000000 | 5000000 | 900.18 | 32.69 | 6.00 | 9311.19 | 82.81 |
| 132 | 2003 | 39.48 | 49.58 | 65.13 | 64.25 | 4500000000 | 4400000000 | 4500000 | 1065.65 | 30.48 | 6.00 | 8577.13 | 50.04 |
| 132 | 2004 | 37.85 | 48.54 | 64.46 | 62.71 | 5200000000 | 4600000000 | 5300000 | 1150.26 | 32.22 | 7.00 | 8938.85 | 80.95 |
| 132 | 2005 | 36.04 | 46.50 | 62.63 | 62.96 | 5100000000 | 4700000000 | 5000000 | 1263.29 | 34.07 | 7.00 | 9704.74 | 99.45 |
| 132 | 2006 | 32.79 | 40.46 | 61.17 | 63.96 | 4900000000 | 5500000000 | 4900000 | 1589.80 | 31.03 | 7.00 | 9159.32 | 86.87 |
| 132 | 2007 | 30.56 | 38.75 | 59.88 | 62.50 | 5800000000 | 6600000000 | 5500000 | 1860.00 | 29.44 | 7.00 | 9141.00 | 79.80 |
| 132 | 2008 | 31 | 40.04 | 59.96 | 62.00 | 8200000000 | 8800000000 | 6200000 | 2166.85 | 29.81 | 7.00 | 9698.96 | 46.92 |
| 132 | 2009 | 32.83 | 38.21 | 62.71 | 64.75 | 6100000000 | 6900000000 | 6300000 | 2261.25 | 24.16 | 7.00 | 10389.90 | 25.12 |
| 132 | 2010 | 31.9 | 39.67 | 60.96 | 63.17 | 7600000000 | 8400000000 | 7000000 | 3122.36 | 24.30 | 7.00 | 9090.43 | 84.52 |
| 132 | 2011 | 31.87 | 41.00 | 60.29 | 62.46 | 9000000000 | 8700000000 | 7700000 | 3643.04 | 26.33 | 7.00 | 8770.43 | 89.36 |
| 132 | 2012 | 33.31 | 43.92 | 59.88 | 62.83 | 9500000000 | 9100000000 | 8000000 | 3694.35 | 24.59 | 8.00 | 9386.63 | 17.02 |
| 132 | 2013 | 33.71 | 44.38 | 59.25 | 63.79 | 10000000000 | 10000000000 | 8800000 | 3623.91 | 23.92 | 8.00 | 10461.20 | 88.48 |
| 132 | 2014 | 34.27 | 43.04 | 60.67 | 64.83 | 12000000000 | 10000000000 | 9400000 | 3491.62 | 23.67 | 8.00 | 11865.20 | 86.56 |
| 132 | 2015 | 35.31 | 45.21 | 60.88 | 64.54 | 12000000000 | 9800000000 | 10000000 | 3331.70 | 21.16 | 8.00 | 13389.40 | 14.42 |
| 132 | 2016 | 34.25 | 44.71 | 59.29 | 64.50 | 13000000000 | 9900000000 | 12000000 | 3562.85 | 19.09 | 8.00 | 13308.30 | 95.00 |
| 132 | 2017 | 31.46 | 40.83 | 58.50 | 63.58 | 15000000000 | 11000000000 | 14000000 | 3837.65 | 20.18 | 8.00 | 13380.80 | 26.54 |
| 132 | 2018 | 31.73 | 40.21 | 59.04 | 64.21 | 18000000000 | 13000000000 | 16000000 | 3893.85 | 20.97 | 8.00 | 14236.90 | 64.99 |
| 132 | 2019 | 30.79 | 39.21 | 58.88 | 63.50 | 18000000000 | 14000000000 | 18000000 | 4135.57 | 18.41 | 8.00 | 71.66 | 98.64 |
| 133 | 2000 | 15.62 | 9.75 | 63.58 | 57.92 | 34000000000 | 52000000000 | 23000000 | 28149.90 | 25.20 | 9.00 | 0.66 | 61.81 |
| 133 | 2001 | 16.29 | 9.71 | 63.71 | 59.17 | 35000000000 | 58000000000 | 21000000 | 27744.50 | 25.04 | 9.00 | 0.69 | 70.43 |
| 133 | 2002 | 16.92 | 11.17 | 63.08 | 59.58 | 36000000000 | 63000000000 | 22000000 | 30056.60 | 24.11 | 9.00 | 0.67 | 79.13 |
| 133 | 2003 | 15.85 | 13.83 | 58.13 | 59.75 | 38000000000 | 69000000000 | 23000000 | 34419.10 | 23.86 | 9.00 | 0.61 | 64.78 |
| 133 | 2004 | 16.96 | 14.08 | 59.46 | 60.38 | 39000000000 | 74000000000 | 26000000 | 40290.30 | 23.71 | 9.00 | 0.55 | 88.66 |
| 133 | 2005 | 19.15 | 16.42 | 60.83 | 61.04 | 40000000000 | 80000000000 | 28000000 | 42030.30 | 24.97 | 9.00 | 0.55 | 99.01 |
| 133 | 2006 | 20.02 | 18.25 | 60.29 | 61.50 | 41000000000 | 85000000000 | 31000000 | 44599.70 | 27.10 | 9.00 | 0.54 | 77.54 |
| 133 | 2007 | 19.37 | 18.17 | 60.08 | 60.50 | 42000000000 | 91000000000 | 31000000 | 50566.80 | 25.24 | 9.00 | 0.50 | 70.12 |
| 133 | 2008 | 21.48 | 20.13 | 60.96 | 61.88 | 43000000000 | 96000000000 | 30000000 | 47287.00 | 26.99 | 9.00 | 0.54 | 45.52 |
| 133 | 2009 | 26.48 | 23.58 | 62.29 | 67.08 | 45000000000 | 1E+11 | 28000000 | 38713.10 | 26.37 | 9.00 | 0.64 | 19.71 |
| 133 | 2010 | 24.4 | 20.33 | 60.50 | 67.96 | 46000000000 | 1.1E+11 | 28000000 | 39435.80 | 28.28 | 9.00 | 0.65 | 97.32 |
| 133 | 2011 | 23.73 | 20.21 | 61.08 | 66.17 | 47000000000 | 1.1E+11 | 29000000 | 42038.60 | 30.70 | 10.00 | 0.62 | 87.04 |
| 133 | 2012 | 25.58 | 23.08 | 62.08 | 66.00 | 48000000000 | 1.2E+11 | 29000000 | 42462.80 | 29.98 | 11.00 | 0.63 | 14.46 |
| 133 | 2013 | 25.79 | 22.54 | 63.08 | 65.96 | 49000000000 | 1.2E+11 | 31000000 | 43444.50 | 29.95 | 11.00 | 0.64 | 74.82 |
| 133 | 2014 | 22.56 | 19.00 | 63.29 | 62.83 | 50000000000 | 1.3E+11 | 33000000 | 47425.60 | 28.50 | 13.00 | 0.61 | 80.86 |
| 133 | 2015 | 19.08 | 14.58 | 63.29 | 60.29 | 52000000000 | 1.4E+11 | 34000000 | 44974.80 | 27.65 | 15.00 | 0.65 | 69.43 |
| 133 | 2016 | 21.6 | 16.08 | 64.71 | 62.42 | 53000000000 | 1.4E+11 | 36000000 | 41064.10 | 28.44 | 16.00 | 0.74 | 83.04 |
| 133 | 2017 | 22.27 | 18.04 | 64.08 | 62.42 | 54000000000 | 1.5E+11 | 38000000 | 40361.40 | 30.37 | 17.00 | 0.78 | 69.16 |
| 133 | 2018 | 22.33 | 19.00 | 63.29 | 62.38 | 55000000000 | 1.5E+11 | 36000000 | 43043.20 | 30.62 | 18.00 | 0.75 | 89.89 |
| 133 | 2019 | 22.1 | 18.67 | 63.17 | 62.38 | 56000000000 | 1.6E+11 | 16000000 | 42330.10 | 31.60 | 18.00 | 15093.00 | 22.51 |
| 134 | 2000 | 29.08 | 30.67 | 63.00 | 64.50 | 940000000 | 390000000 | 1600000 | 1674.83 | 41.25 | 2.00 | 0.71 | 9.71 |
| 134 | 2001 | 28.18 | 29.21 | 63.17 | 64.00 | 880000000 | 420000000 | 1700000 | 1744.46 | 41.55 | 2.00 | 0.71 | 13.44 |
| 134 | 2002 | 29.29 | 31.42 | 63.33 | 63.83 | 1300000000 | 500000000 | 2400000 | 1827.34 | 46.77 | 2.00 | 0.71 | 64.13 |
| 134 | 2003 | 29.4 | 31.29 | 63.50 | 64.00 | 1300000000 | 500000000 | 2400000 | 1902.59 | 46.72 | 2.00 | 0.71 | 73.48 |
| 134 | 2004 | 27.69 | 27.92 | 63.50 | 63.96 | 1600000000 | 590000000 | 2900000 | 2073.64 | 51.47 | 3.00 | 0.71 | 62.68 |
| 134 | 2005 | 24.56 | 26.71 | 61.00 | 61.42 | 1800000000 | 650000000 | 3000000 | 2214.02 | 51.98 | 3.00 | 0.71 | 40.66 |
| 134 | 2006 | 24.92 | 27.33 | 61.00 | 61.50 | 2400000000 | 960000000 | 3200000 | 2548.27 | 53.13 | 3.00 | 0.71 | 78.90 |
| 134 | 2007 | 24.85 | 27.21 | 61.00 | 61.50 | 2800000000 | 1000000000 | 3400000 | 2773.74 | 53.48 | 3.00 | 0.71 | 32.47 |
| 134 | 2008 | 28.17 | 28.67 | 61.50 | 66.17 | 3500000000 | 1100000000 | 3700000 | 3398.15 | 55.72 | 3.00 | 0.71 | 96.02 |
| 134 | 2009 | 33.04 | 28.71 | 62.38 | 75.00 | 3500000000 | 1200000000 | 3800000 | 3504.00 | 45.79 | 3.00 | 0.71 | 92.57 |
| 134 | 2010 | 30.19 | 30.71 | 59.25 | 70.42 | 4400000000 | 1700000000 | 4200000 | 3690.11 | 47.59 | 3.00 | 0.71 | 73.48 |
| 134 | 2011 | 32.58 | 37.00 | 57.33 | 70.83 | 4400000000 | 1300000000 | 4000000 | 3816.28 | 47.00 | 4.00 | 0.71 | 37.41 |
| 134 | 2012 | 31.98 | 37.46 | 56.00 | 70.50 | 5100000000 | 1300000000 | 4200000 | 3877.34 | 45.61 | 4.00 | 0.71 | 21.83 |
| 134 | 2013 | 33.02 | 37.50 | 58.33 | 70.21 | 5100000000 | 1200000000 | 3900000 | 3998.03 | 41.77 | 4.00 | 0.71 | 4.27 |
| 134 | 2014 | 34.52 | 38.63 | 61.04 | 69.38 | 5500000000 | 1300000000 | 4000000 | 4072.77 | 42.73 | 4.00 | 0.71 | 95.78 |
| 134 | 2015 | 32.94 | 38.96 | 60.17 | 66.75 | 5000000000 | 1300000000 | 3800000 | 4105.45 | 37.07 | 5.00 | 0.71 | 44.32 |
| 134 | 2016 | 33.58 | 39.17 | 60.75 | 67.25 | 4900000000 | 1400000000 | 3600000 | 4103.73 | 34.66 | 5.00 | 0.71 | 54.86 |
| 134 | 2017 | 35.98 | 40.54 | 63.00 | 68.42 | 5500000000 | 1500000000 | 3800000 | 4162.82 | 35.12 | 5.00 | 0.71 | 81.69 |
| 134 | 2018 | 36.17 | 41.33 | 63.00 | 68.00 | 6200000000 | 1500000000 | 4200000 | 4241.79 | 35.64 | 5.00 | 0.71 | 34.93 |
| 134 | 2019 | 35.5 | 41.17 | 62.71 | 67.13 | 6900000000 | 1600000000 | 5400000 | 2715.28 | 106.80 | 5.00 | 0.71 | 23.20 |
| 135 | 2000 | 31.67 | 31.67 | 67.33 | 64.33 | 1400000000 | 830000000 | 2100000 | 390.09 | 53.92 | 5.00 | 14167.80 | 99.65 |
| 135 | 2001 | 30.7 | 34.21 | 64.08 | 63.13 | 1300000000 | 860000000 | 2300000 | 404.81 | 55.06 | 5.00 | 14725.20 | 37.55 |
| 135 | 2002 | 30.56 | 33.67 | 64.00 | 63.46 | 1600000000 | 900000000 | 2600000 | 430.05 | 54.74 | 5.00 | 15279.50 | 99.60 |
| 135 | 2003 | 29.81 | 33.46 | 62.21 | 63.96 | 1400000000 | 940000000 | 2400000 | 480.58 | 56.67 | 6.00 | 15509.60 | 75.29 |
| 135 | 2004 | 30.44 | 34.58 | 61.25 | 65.04 | 1700000000 | 980000000 | 2900000 | 546.91 | 59.73 | 6.00 | 15746.00 | 20.28 |
| 135 | 2005 | 29.94 | 33.50 | 60.58 | 65.79 | 2300000000 | 900000000 | 3500000 | 687.48 | 63.70 | 6.00 | 15858.90 | 84.58 |
| 135 | 2006 | 27.6 | 31.67 | 60.63 | 62.92 | 2900000000 | 1100000000 | 3600000 | 784.37 | 67.72 | 6.00 | 15994.30 | 82.32 |
| 135 | 2007 | 28.02 | 31.04 | 60.46 | 64.54 | 3800000000 | 1200000000 | 4200000 | 906.28 | 70.52 | 6.00 | 16105.10 | 80.72 |
| 135 | 2008 | 30.37 | 32.54 | 60.17 | 68.04 | 3900000000 | 1300000000 | 4200000 | 1149.42 | 70.34 | 6.00 | 16302.30 | 24.73 |
| 135 | 2009 | 31.79 | 33.50 | 59.92 | 70.17 | 3100000000 | 1100000000 | 3700000 | 1217.27 | 62.61 | 6.00 | 17065.10 | 97.06 |
| 135 | 2010 | 31.69 | 34.25 | 59.25 | 69.88 | 4500000000 | 1500000000 | 5100000 | 1317.89 | 72.00 | 7.00 | 18612.90 | 70.68 |
| 135 | 2011 | 33.48 | 37.04 | 60.42 | 69.50 | 5700000000 | 1700000000 | 6000000 | 1525.12 | 79.39 | 8.00 | 20509.80 | 6.33 |
| 135 | 2012 | 33.62 | 38.08 | 60.67 | 68.50 | 6900000000 | 1900000000 | 6800000 | 1735.14 | 80.03 | 8.00 | 20828.00 | 82.66 |
| 135 | 2013 | 32.48 | 39.75 | 60.63 | 64.58 | 7300000000 | 2100000000 | 7600000 | 1886.67 | 83.63 | 8.00 | 20933.40 | 18.40 |
| 135 | 2014 | 31.12 | 39.21 | 59.71 | 63.33 | 7400000000 | 2700000000 | 7900000 | 2030.26 | 86.40 | 9.00 | 21148.00 | 82.20 |
| 135 | 2015 | 29.02 | 35.67 | 59.71 | 62.67 | 7400000000 | 3600000000 | 7900000 | 2085.10 | 89.78 | 10.00 | 21697.60 | 15.94 |
| 135 | 2016 | 28.77 | 35.75 | 59.54 | 62.25 | 8500000000 | 4500000000 | 10000000 | 2192.21 | 93.62 | 10.00 | 21935.00 | 88.50 |
| 135 | 2017 | 31.44 | 37.79 | 60.88 | 64.21 | 8900000000 | 5000000000 | 13000000 | 2365.62 | 101.59 | 10.00 | 22370.10 | 47.13 |
| 135 | 2018 | 30.87 | 37.04 | 60.13 | 64.58 | 10000000000 | 5900000000 | 15000000 | 2566.60 | 105.83 | 10.00 | 22602.10 | 28.88 |
| 135 | 2019 | 29.69 | 36.25 | 59.96 | 63.17 | 12000000000 | 6200000000 | 18000000 | 1305.06 | 34.64 | 10.00 | 22834.00 | 56.05 |
| 136 | 2000 | 41.08 | 32.42 | 77.00 | 72.75 | 820000000 | 100000000 | 457000 | 345.69 | 23.92 | 1.00 | 3.11 | 20.72 |
| 136 | 2001 | 42.7 | 38.21 | 74.29 | 72.92 | 820000000 | 100000000 | 492000 | 382.94 | 25.11 | 1.00 | 3.61 | 86.37 |
| 136 | 2002 | 49.92 | 44.79 | 79.00 | 76.04 | 820000000 | 110000000 | 565000 | 382.24 | 27.13 | 1.00 | 4.40 | 65.80 |
| 136 | 2003 | 48.12 | 43.50 | 76.25 | 76.50 | 820000000 | 120000000 | 413000 | 435.46 | 25.68 | 1.00 | 4.73 | 27.28 |
| 136 | 2004 | 40.08 | 36.92 | 72.83 | 70.42 | 820000000 | 86000000 | 515000 | 538.59 | 33.54 | 1.00 | 4.78 | 66.77 |
| 136 | 2005 | 39 | 35.96 | 72.04 | 70.00 | 820000000 | 87000000 | 669000 | 702.74 | 30.61 | 1.00 | 4.47 | 48.45 |
| 136 | 2006 | 38.58 | 35.88 | 71.63 | 69.67 | 820000000 | 92000000 | 757000 | 1047.92 | 32.59 | 1.00 | 3.60 | 60.50 |
| 136 | 2007 | 37.04 | 36.96 | 69.21 | 67.92 | 820000000 | 120000000 | 897000 | 1124.29 | 33.59 | 1.00 | 4.00 | 34.45 |
| 136 | 2008 | 34.37 | 36.83 | 64.17 | 67.75 | 820000000 | 160000000 | 812000 | 1394.00 | 28.92 | 1.00 | 3.75 | 6.25 |
| 136 | 2009 | 34.21 | 36.54 | 64.38 | 67.50 | 820000000 | 140000000 | 710000 | 1159.91 | 29.25 | 1.00 | 5.05 | 17.92 |
| 136 | 2010 | 32.87 | 37.00 | 60.08 | 68.67 | 820000000 | 170000000 | 815000 | 1489.46 | 37.03 | 1.00 | 4.80 | 96.49 |
| 136 | 2011 | 29.42 | 37.33 | 56.46 | 65.04 | 820000000 | 220000000 | 920000 | 1672.91 | 40.47 | 1.00 | 4.86 | 74.66 |
| 136 | 2012 | 29.58 | 37.00 | 57.42 | 64.75 | 820000000 | 300000000 | 859000 | 1763.07 | 40.08 | 1.00 | 5.15 | 91.28 |
| 136 | 2013 | 28.67 | 38.00 | 56.83 | 62.50 | 820000000 | 360000000 | 915000 | 1878.91 | 40.48 | 1.00 | 5.40 | 92.17 |
| 136 | 2014 | 32.96 | 39.46 | 60.46 | 66.00 | 820000000 | 380000000 | 947000 | 1763.06 | 38.82 | 1.00 | 6.15 | 89.55 |
| 136 | 2015 | 32.92 | 37.63 | 63.13 | 65.08 | 820000000 | 400000000 | 932000 | 1337.80 | 37.14 | 1.00 | 8.63 | 32.61 |
| 136 | 2016 | 34.71 | 36.92 | 64.04 | 68.46 | 820000000 | 420000000 | 956000 | 1280.58 | 35.32 | 1.00 | 10.31 | 73.36 |
| 136 | 2017 | 34.79 | 37.00 | 63.33 | 69.25 | 820000000 | 410000000 | 1100000 | 1534.87 | 34.99 | 1.00 | 9.52 | 16.16 |
| 136 | 2018 | 34.25 | 38.71 | 63.92 | 65.88 | 820000000 | 480000000 | 1100000 | 1556.33 | 36.98 | 1.00 | 10.46 | 37.60 |
| 136 | 2019 | 37.73 | 39.71 | 67.42 | 68.33 | 820000000 | 510000000 | 1300000 | 14896.50 | 28.20 | 1.00 | 11.40 | 84.43 |
| 137 | 2000 | 25.85 | 28.00 | 62.33 | 61.38 | 1200000000 | 900000000 | 1700000 | 5074.90 | 30.53 | 2.00 | 539.59 | 93.31 |
| 137 | 2001 | 24.08 | 21.71 | 64.50 | 61.96 | 1200000000 | 940000000 | 1700000 | 4574.59 | 32.40 | 2.00 | 634.94 | 69.54 |
| 137 | 2002 | 23.85 | 22.71 | 63.00 | 62.00 | 1200000000 | 930000000 | 1400000 | 4446.25 | 32.80 | 2.00 | 688.94 | 80.45 |
| 137 | 2003 | 23.62 | 23.21 | 62.92 | 61.13 | 1300000000 | 1100000000 | 1600000 | 4772.56 | 35.66 | 3.00 | 691.40 | 79.75 |
| 137 | 2004 | 21.29 | 21.38 | 61.79 | 59.42 | 1600000000 | 1300000000 | 1800000 | 6194.85 | 39.80 | 3.00 | 609.53 | 64.41 |
| 137 | 2005 | 18.56 | 18.46 | 60.42 | 58.25 | 1600000000 | 1100000000 | 2000000 | 7598.52 | 40.16 | 4.00 | 559.77 | 56.04 |
| 137 | 2006 | 17.79 | 18.25 | 59.75 | 57.58 | 1900000000 | 1200000000 | 2300000 | 9464.55 | 43.80 | 5.00 | 530.28 | 57.82 |
| 137 | 2007 | 17.62 | 19.79 | 59.50 | 55.96 | 2200000000 | 1400000000 | 2500000 | 10502.40 | 45.07 | 5.00 | 522.46 | 20.99 |
| 137 | 2008 | 19.81 | 21.54 | 61.00 | 57.08 | 2500000000 | 1400000000 | 2700000 | 10751.50 | 41.42 | 5.00 | 522.46 | 85.38 |
| 137 | 2009 | 23.75 | 21.21 | 61.96 | 64.33 | 2400000000 | 1500000000 | 2800000 | 10208.90 | 37.04 | 5.00 | 560.86 | 65.19 |
| 137 | 2010 | 21.73 | 22.42 | 60.29 | 60.75 | 2400000000 | 1700000000 | 2800000 | 12808.00 | 37.75 | 5.00 | 510.25 | 71.04 |
| 137 | 2011 | 22.69 | 25.25 | 61.21 | 58.92 | 2700000000 | 2100000000 | 3100000 | 14637.20 | 37.76 | 5.00 | 483.67 | 53.09 |
| 137 | 2012 | 23.06 | 25.54 | 60.33 | 60.25 | 3200000000 | 2400000000 | 3600000 | 15351.60 | 34.12 | 5.00 | 486.47 | 72.94 |
| 137 | 2013 | 22.25 | 24.67 | 60.08 | 59.75 | 3200000000 | 2500000000 | 3600000 | 15842.90 | 32.20 | 5.00 | 495.27 | 96.00 |
| 137 | 2014 | 23.85 | 23.63 | 61.58 | 62.50 | 3200000000 | 2700000000 | 3700000 | 14671.00 | 33.11 | 6.00 | 570.35 | 27.37 |
| 137 | 2015 | 24.42 | 24.83 | 61.21 | 62.79 | 3400000000 | 2500000000 | 4500000 | 13574.20 | 29.38 | 6.00 | 654.12 | 62.55 |
| 137 | 2016 | 25.62 | 26.54 | 61.00 | 63.71 | 3700000000 | 2700000000 | 5600000 | 13753.60 | 28.15 | 6.00 | 676.96 | 74.38 |
| 137 | 2017 | 25.37 | 27.42 | 60.50 | 62.83 | 4100000000 | 3100000000 | 6500000 | 14999.40 | 28.44 | 6.00 | 648.83 | 86.11 |
| 137 | 2018 | 23.44 | 25.04 | 60.46 | 61.38 | 3900000000 | 3100000000 | 5700000 | 15924.80 | 28.59 | 6.00 | 641.28 | 72.24 |
| 137 | 2019 | 24.19 | 24.88 | 61.54 | 61.96 | 3300000000 | 3100000000 | 5400000 | 10261.70 | 18.42 | 6.00 | 633.72 | 92.98 |
| 138 | 2000 | 26.92 | 37.58 | 55.58 | 60.67 | 17000000000 | 14000000000 | 31000000 | 959.37 | 20.89 | 29.00 | 8.28 | 88.17 |
| 138 | 2001 | 27.02 | 37.75 | 54.71 | 61.58 | 19000000000 | 15000000000 | 33000000 | 1053.11 | 20.31 | 31.00 | 8.28 | 90.01 |
| 138 | 2002 | 25.06 | 33.71 | 55.00 | 61.42 | 22000000000 | 17000000000 | 37000000 | 1148.51 | 22.64 | 31.00 | 8.28 | 95.49 |
| 138 | 2003 | 23.35 | 30.50 | 54.79 | 61.42 | 19000000000 | 17000000000 | 33000000 | 1288.64 | 26.98 | 33.00 | 8.28 | 77.07 |
| 138 | 2004 | 23.29 | 29.83 | 55.33 | 61.42 | 28000000000 | 21000000000 | 42000000 | 1508.67 | 31.06 | 36.00 | 8.28 | 41.40 |
| 138 | 2005 | 22.6 | 30.75 | 53.92 | 60.54 | 37000000000 | 26000000000 | 47000000 | 1753.42 | 33.83 | 37.00 | 8.19 | 76.48 |
| 138 | 2006 | 21.75 | 31.67 | 52.67 | 59.17 | 46000000000 | 31000000000 | 50000000 | 2099.23 | 36.04 | 39.00 | 7.97 | 69.25 |
| 138 | 2007 | 20.62 | 30.88 | 52.08 | 58.29 | 55000000000 | 35000000000 | 55000000 | 2693.97 | 35.43 | 41.00 | 7.61 | 31.00 |
| 138 | 2008 | 21.25 | 31.92 | 52.08 | 58.50 | 64000000000 | 40000000000 | 53000000 | 3468.30 | 32.60 | 43.00 | 6.95 | 75.00 |
| 138 | 2009 | 22.21 | 33.00 | 52.21 | 59.21 | 73000000000 | 45000000000 | 51000000 | 3832.24 | 24.75 | 44.00 | 6.83 | 70.00 |
| 138 | 2010 | 23.6 | 35.54 | 52.00 | 59.67 | 82000000000 | 49000000000 | 56000000 | 4550.45 | 27.19 | 46.00 | 6.77 | 83.73 |
| 138 | 2011 | 25.73 | 39.08 | 52.04 | 60.33 | 91000000000 | 54000000000 | 58000000 | 5618.13 | 26.57 | 47.00 | 6.46 | 74.39 |
| 138 | 2012 | 25.79 | 38.88 | 52.50 | 60.21 | 100000000000 | 59000000000 | 58000000 | 6316.92 | 25.49 | 49.00 | 6.31 | 20.08 |
| 138 | 2013 | 25.69 | 38.71 | 52.50 | 60.17 | 110000000000 | 63000000000 | 56000000 | 7050.65 | 24.60 | 51.00 | 6.20 | 39.90 |
| 138 | 2014 | 27.85 | 42.96 | 52.54 | 60.21 | 120000000000 | 68000000000 | 56000000 | 7678.60 | 23.51 | 54.00 | 6.14 | 90.69 |
| 138 | 2015 | 27.58 | 43.63 | 52.29 | 59.25 | 130000000000 | 72000000000 | 57000000 | 8066.94 | 21.35 | 55.00 | 6.23 | 75.77 |
| 138 | 2016 | 28.77 | 45.00 | 53.54 | 59.00 | 140000000000 | 77000000000 | 59000000 | 8147.94 | 19.58 | 57.00 | 6.64 | 69.85 |
| 138 | 2017 | 27.25 | 42.00 | 52.83 | 59.67 | 150000000000 | 82000000000 | 61000000 | 8879.44 | 19.69 | 59.00 | 6.76 | 14.30 |
| 138 | 2018 | 26.9 | 40.50 | 53.29 | 60.00 | 150000000000 | 86000000000 | 63000000 | 9976.68 | 19.11 | 60.00 | 6.62 | 79.87 |
| 138 | 2019 | 27.46 | 40.17 | 54.13 | 60.63 | 160000000000 | 91000000000 | 160000000 | 11073.90 | 18.42 | 60.00 | 6.47 | 56.50 |
